# Retracing the path of planar cell polarity

**DOI:** 10.1186/s12862-016-0641-0

**Published:** 2016-04-02

**Authors:** Quentin Schenkelaars, Laura Fierro-Constain, Emmanuelle Renard, Carole Borchiellini

**Affiliations:** Institut Méditerranéen de Biodiversité et d’Ecologie marine et continentale (IMBE UMR 7263), Aix Marseille Université, CNRS, IRD, Avignon Université, Station marine d’Endoume, Batterie des Lions, 13007 Marseille, France; Department of Genetics and Evolution, Institute of Genetics and Genomics in Geneva (IGe3), Faculty of Sciences, University of Geneva, Geneva, Switzerland

**Keywords:** Planar cell polarity, Van Gogh, Multigene families, Porifera, Placozoa, Ctenophora, Wnt signaling, Metazoan phylogeny

## Abstract

**Background:**

The Planar Cell Polarity pathway (PCP) has been described as the main feature involved in patterning cell orientation in bilaterian tissues. Recently, a similar phenomenon was revealed in cnidarians, in which the inhibition of this pathway results in the absence of cilia orientation in larvae, consequently proving the functional conservation of PCP signaling between Cnidaria and Bilateria. Nevertheless, despite the growing accumulation of databases concerning basal lineages of metazoans, very few information concerning the existence of PCP components have been gathered outside of Bilateria and Cnidaria. Thus, the origin of this module or its prevalence in early emerging metazoans has yet to be elucidated.

**Results:**

The present study addresses this question by investigating the genomes and transcriptomes from all poriferan lineages in addition to *Trichoplax* (Placozoa) and *Mnemiopsis* (Ctenophora) genomes for the presence of the core components of this pathway. Our results confirm that several PCP components are metazoan innovations. In addition, we show that all members of the PCP pathway, including a *bona fide* Strabismus ortholog (Van gogh), are retrieved only in one sponge lineage (Homoscleromorpha) out of four. This highly suggests that the full PCP pathway dates back at least to the emergence of homoscleromorph sponges. Consequently, several secondary gene losses would have occurred in the three other poriferan lineages including *Amphimedon queenslandica* (Demospongiae). Several proteins were not retrieved either in placozoans or ctenophores leading us to discuss the difficulties to predict orthologous proteins in basally branching animals. Finally, we reveal how the study of multigene families may be helpful to unravel the relationships at the base of the metazoan tree.

**Conclusion:**

The PCP pathway antedates the radiation of Porifera and may have arisen in the last common ancestor of animals. *Oscarella* species now appear as key organisms to understand the ancestral function of PCP signaling and its potential links with Wnt pathways.

**Electronic supplementary material:**

The online version of this article (doi:10.1186/s12862-016-0641-0) contains supplementary material, which is available to authorized users.

## Background

**Fig. 1:**
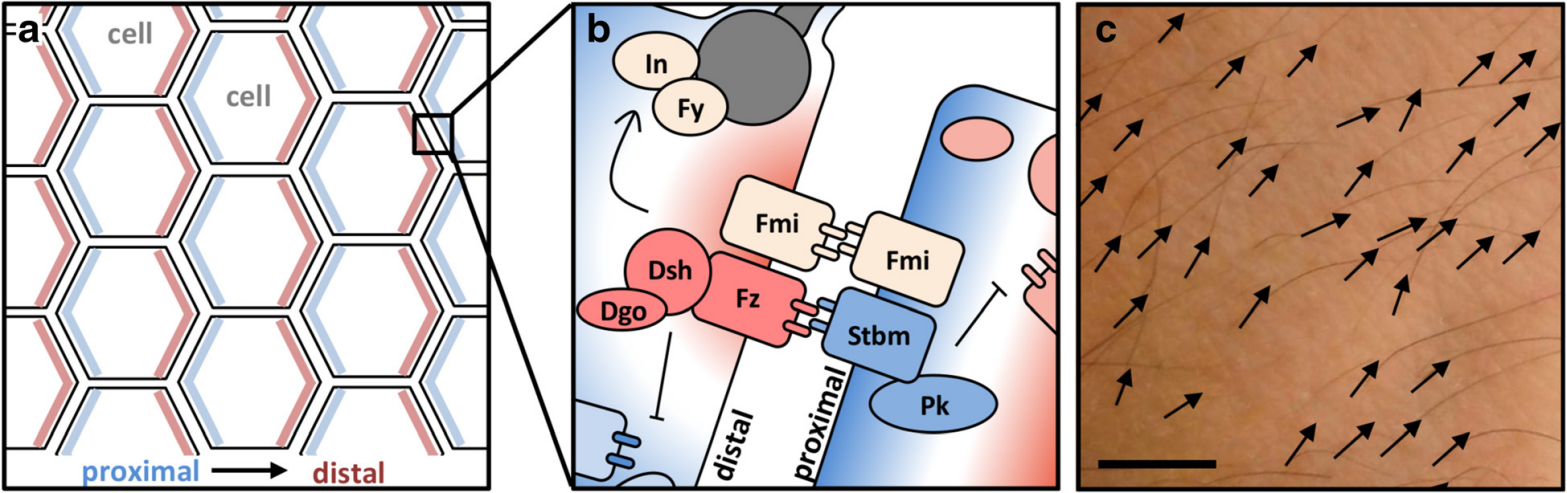
The PCP pathway: two antagonist complexes at each cell pole. Schemas showing the location (**a**) and the composition (**b**) of the two antagonist complexes. Picture of the arm-hair in *Homo sapiens* showing the cells orientation, scale: 1 cm (**c**). Abbreviations. *Dsh* Dishevelled; *Fmi* Flamingo; *Fy* Fuzzy; *Fz* Frizzled; *In* Inturned; *Pk* Prickle; *Stbm* Strabismus

Planar Cell Polarity (PCP) consists in the unique directional orientation of epithelial cells within the plane of the cell layer (Fig. 1a). The study of planar polarity phenomenon has started by the discovery of a collection of *Drosophila* mutants showing defect in patterning the orientation of wing trichomes, raising the question of the underlying molecular mechanisms for such cell organization [1–3]. Extensive investigation of *Drosophila* as a model for studying planar polarity led to the well characterization of six main proteins, now usually referred as the core pathway: Flamingo (Fmi), Frizzled (Fz), Dishevelled (Dsh), Strabismus (Stbm), Prickle (Pk) and Diego (Dgo) (See Table 1 for alternative names). Briefly, the PCP pathway is involved in patterning two different clusters of proteins within bilaterian cells (Fig. 1a, and b). Indeed, the polarized distribution of the two antagonist complexes composed by Fmi/Fz/Dsh/Dgo and Fmi/Stbm/Pk proteins (localized at the distal and proximal poles, respectively, in neighboring cells) is required to induce polarized events (e.g. asymmetric placement of hair or cilium, orientation of the mitotic spindle, etc.) that lead to the morphological orientation of cells in a plan perpendicular to the apico-basal cellular axis.

**Table 1:** Alternative names of PCP core components retrieved in metazoans and choanoflagellates. “No” means that the protein was not found. The asterisk indicates that even if no Fz was retrieved in *Salpingoeca rosetta*, the Fz family has been described in Fungi and Amoebozoa

*Drosophila*	Vertebrates	*Clytia*	*Trichoplax*	Porifera	*Mnemiopsis*	*Salpingoeca*
Van Gogh (Vang) or Strabismus (Stbm)	VangL1, 2	Stbm	Vang	Vang	No	No
Prickle (Pk) & Espinas (Esn)	Pkl1, 2, 3	Pk	Pk	PkTesL	PkTesL	PkTesL
Testin (Tes)	Tes	Tes	No			
Frizzled	Fzd3, 6, 7	Fz1	FzdA	FzdA	FzdA	No^*^
Dishvelled (Dsh)	Dvl 1–3	Dsh	Dsh	Dvl or DvlA, B	Dsh	No
Flamingo (Fmi) or Starry night (Stan)	Celsr1–3	Fmi	Celsr	Fmi or Celsr3L	No	No
Diego (Dgo)	Ankrd6, Invs	Dgo	No	Invs, 2	No	Invs
Fuzzy (Fy)	Fuz	Fy	Fy	Fuz	Fy	Fy
Inturned (In)	Intu	In	In	Intu	No	Inl?

Several evidences support that Wnt signaling regulates PCP in vertebrates [4] and Drosophila [1] even if precise mechanisms involved are so far misunderstood. In addition, various proteins have been identified as downstream effectors of the core pathway. For instance, Fuzzy (Fy) and Inturned (In) (Table 1) are potential additional effectors for the regulation of actin assembly at the basal body of cilia and are thus responsible for flagellum orientation (Fig. 1b) [5–8]. Various modules involving Rho GTPases, such as the Rho/Rock pathway, have been also described downstream of the core pathway [9, 10]. The Rho associated kinase (Rock) regulates acto-myosin dynamics (thought Myosin II phosphorylation) in order to induce changes in cell behavior (i.e. cell shape and cell adhesion). This explains why the down-regulation of the PCP core is often associated with defects in morphogenesis processes such as gastrulation or invagination (reviewed in [1]). Whereas they undoubtedly act in an intermingled way during animal development, the PCP core and the Rho/Rock modules now tends to be considered as two distinct modules [3, 11, 12]. For this reason, and because the presence and function of the Rho/Rock pathway has been recently investigated in sponges [13], the present study focuses on the origin of main PCP components (in addition to Fy and In) and only discusses the data in the light of what is known concerning the coordination of cell orientation in concerned organisms.

As recently reviewed by Hale and Strutt (2015) [12], the function of the PCP core proteins in epithelial cell orientation is well documented in various developmental processes across planulozoans (bilaterians + cnidarians) [14, 15]. Among those commonly used to study the planar polarized tissues in *Drosophila* are the alignment of actin hairs on the abdomen, the alignment of wing sensory bristles, and the arrangement of eye facet systems [16–18]. A further example of reliance on the PCP pathway can be found in the orientation of inner ear sensory hair cells and of hair follicles of vertebrate skin (Fig. 1c) [18–21]. Although the study of the planar polarity genes have been mainly focused on *Drosophila* and mammalians, some lines of evidence strongly support as well the involvement of the core PCP in cilia orientation in less conventional models. For instance, *stbm* knockdown experiments led to the dramatic disorganization of cilia in *Planaria* [22] and in the planula larva of the cnidarian *Clytia hemisphaerica* [23]. Interestingly, these results imply that the PCP pathway was certainly already present in the last common ancestor of Bilateria and Cnidaria for patterning cell polarity processes.

**Fig. 2:**
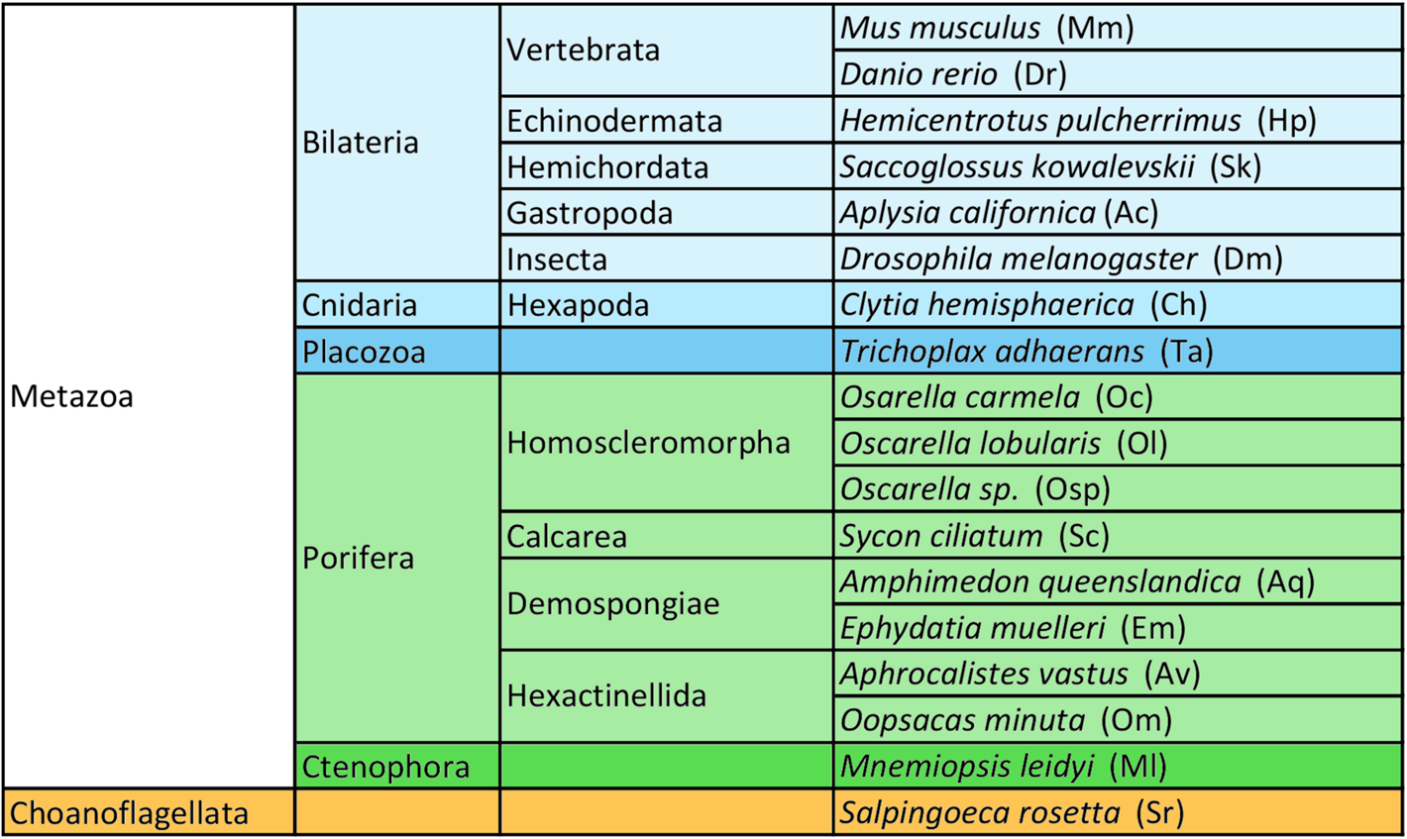
List of the studied species, representing the five metazoan clades and choanoflagellates. Species abbreviations used in domain analyses and phylogenies are provided (see Additional file 1 for more information concerning databases)

Previous studies have suggested that most components of the PCP pathway such as Dsh, Stbm, Fmi, and In arose during animal evolution [24, 25]. In contrast, other proteins, Pk, Dgo and Fy, have more ancient origins and can be retrieved in Choanoflagellata [11, 26–28]. In addition, some authors have recently reported sequences that may belong to the Fz family in Fungi and Amoebozoa [29, 30], suggesting that this protein family was already present in the last common ancestor of unikonts. In order to retrace the full evolution of these genes (emergence, loss and duplication), we performed an exhaustive research of all the components of the PCP core in transcriptomes and genomes of non-planulozoan lineages poorly or un-explored so far (Fig. 2) including i) the four poriferan classes: Demospongiae (*Amphimedon queenslandica* and *Ephydatia muelleri*), Hexactinellida (*Aphrocalistes vastus* and *Oopsacas minuta*), Calcarea (*Sycon ciliatum*) and Homoscleromorpha (*Oscarella lobularis*, *Oscarella carmella* and *Oscarella sp*.); ii) the ctenophores *Mnemiopsis leidyi*; iii) the placozoan *Trichoplax adhaerens* and iv) a non-metazoan species, the choanoflagellates *Salpingoeca rosetta* (Fig. 2). Our findings are carefully discussed and interpreted in the light of the most recent phylogenetic hypotheses and of available functional data. This paper thus fulfill the major sampling gap of previous studies that so far prevented to fully explore the earliest evolutionary steps of the PCP toolkit.

## Results and Discussion

### Fz, Fy, Dgo and the PET family arose in the last common ancestor of Metazoa and Choanoflagellata

As previously reported [11], few members of the core PCP pathway were identified in the genome of the choanoflagellate *Salpingoeca rosetta* (Table 1 and Additional file 1). Indeed, our researches by TBLASN and domain analyses confirm that Dgo (Ankrd6 or Invs) and Fy proteins and the PET (Prickle/Espinas/Testin) family antedate the emergence of metazoans (Table 1). In contrast, these genes have not been retrieved so far in other unikonts such as *Capsaspora owczarzaki* (Filasterea), *Spizellomyces punctatus*, *Allomyces macrogynus* (Fungi) and *Dictyostelium discoïdeum* (Amoebozoa) [11, 31]. This strongly suggests that *fy*, *dgo* and the PET families were inherited from the last common ancestor of choanoflagellates and metazoans. We show here that several of these ancient genes may have been secondarily lost during animal evolution: no *dgo* orthologous gene was found in the genomes of *Mnemiopsis leidyi* and *Trichoplax adhaerens*, and no *fy* was identified in either of the glass sponge transcriptomes (*Oopsacas minuta* and *Aphrocalistes vastus*), whereas it is present in all other sponges studied here, including the *Amphimedon queenslandica* genome in which it had previously been considered as missing (Table 1 and Additional file 1) [24]. Concerning the Fz family, even if no orthologous protein was retrieved in *S. rosetta*, some authors have recently reported sequences that may belong to the Fz family in Fungi and Amoebozoa [29, 30], suggesting that this protein family was already present in the last common ancestor of unikonts. Furthermore our recent study supports that the last common ancestor of metazoans certainly already possessed two ancestral paralogs (FzdA and FzdB) while two duplication events would have occurred just before the radiation of Cnidarians [32].

### Evolution of the Prickle/Espinas/Testin (PET) family

**Fig. 3:**
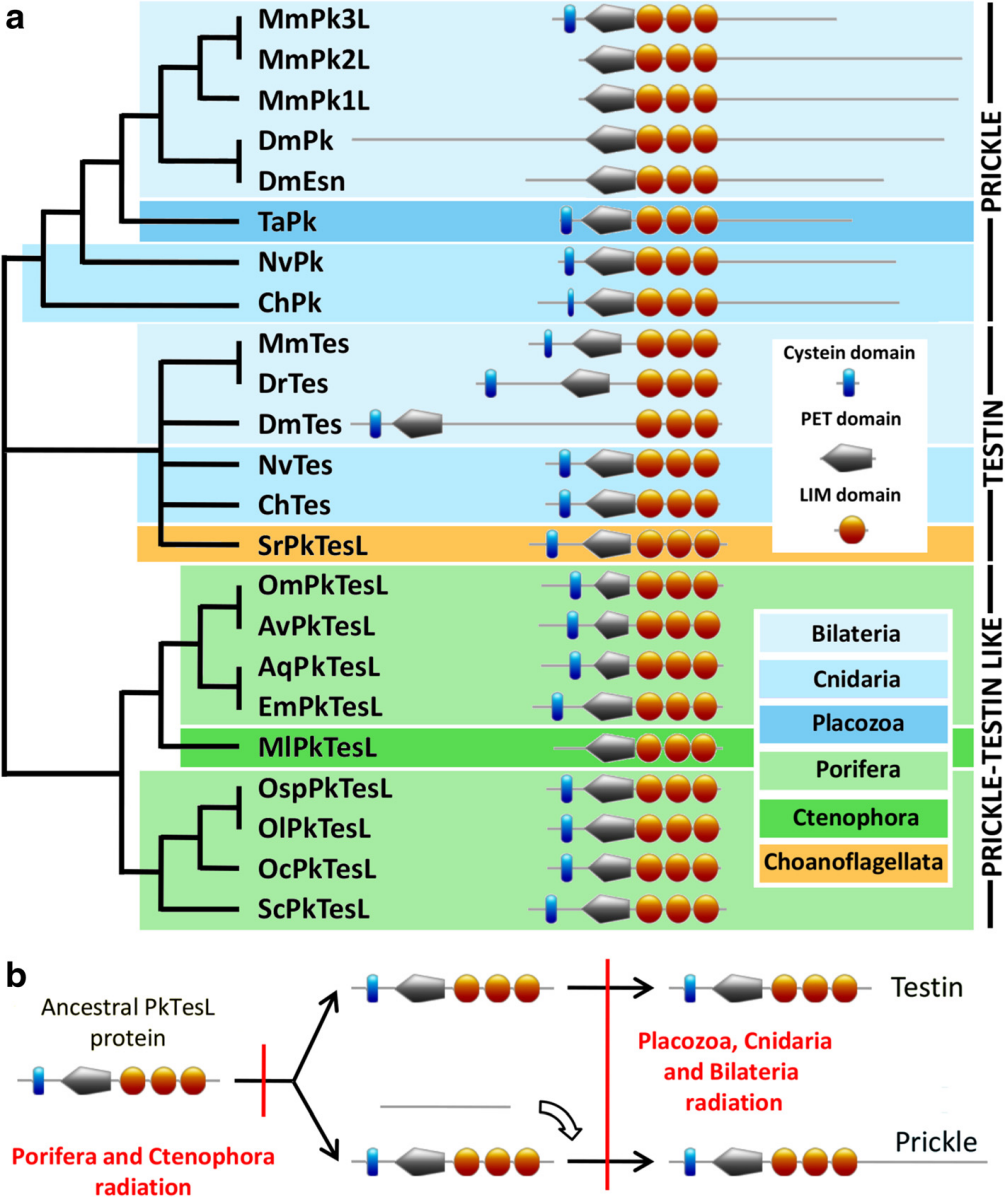
Evolution of Prickle and Testin from an ancestral PkTesL protein without a long C-terminal tail. Functional domain analyses and phylogenetic trees of Prickle (Pk) and Testin (Tes) show the long C-terminal tail of Pk compared to Tes (**a**). The position of each protein in the tree has been determined by ML and Bayesian phylogenetic analyses (Additional file 2). A unique and ancestral Prickle/Testin-like protein (retrieved in Porifera and Ctenophora: PkTesL) is presumed to have given birth to Pk and Tes proteins (retrieved in Bilateria, Cnidaria and Placozoa) (**b**). The tail was added at the C-terminal end of Pk before the split between ctenophores and (cnidarians + bilaterians). Tes was then secondarily lost in placozoans (not represented). Abbreviations. Bilateria: Dm, *Drosophila melanogaster* and Mm, *Mus musculus*. Cnidaria: Ch, *Clytia hemisphaerica* and Nv, *Nematostella vectensis*. Placozoa: *Trichoplax adhaerens*. Porifera: Aq, *Amphimedon queenslandica*; Av, *Aphrocalistes vastus*; Em, *Ephydatia muelleri*; *Oc, Oscarella carmela*; *Ol, Oscarella lobularis*; *Om, Oopsacas minuta*; *Osp, Oscarella sp*. and Sc, *Sycon ciliatum*. Ctenophora: *Mnemiopsis leidyi*. Choanoflagellata: Sr, *Salpingoeca rosetta*. Scale: 200 amino-acids

Two different groups of proteins belonging to the PET family (Pk and Tes) were characterized in bilaterians and cnidarians. Consistently, our present phylogenetic analyses well support the two paralogous clusters and suggest that the third group described in *Drosophila* (Espinas) arose from the specific duplication of the *pk* gene in this lineage (Additional file 2). In addition, our study reveals that Pk and Tes can be easily discriminated due to the fact that Pk possesses an additional long C-terminal tail following the last LIM domain (Fig. 3a and Additional file 2). Nevertheless, there is no particular conserved motif or domain in this Pk tail, preventing speculation as to the functional significance of this difference. According to recent findings, Tes protein can interact with the C-terminal cytoplasmic domain of VangL2 (one of the two mammalian orthologous proteins of Stbm) in yeast two-hybrid assay and the co-expression of VangL2-RFP and Tes-GFP led to an enrichment of Tes at the cellular contact [21], suggesting that the Pk C-tail may not be needed for the protein interaction with Stbm/Vang. Moreover, the functional importance of this region may be open to question, considering its significant divergence between taxa (average sequence identity = 10 % and see alignment in Additional file 2).

Interestingly, only one sequence belonging to the PET family was retrieved in Porifera, Ctenophora and Choanoflagellata (Fig. 3a). We named this single protein PkTesL and it is devoid of the long C-term tail. This strongly suggests that a unique ancestral *prickle/testin-like* copy was already present in the last common ancestor of metazoans and choanoflagellates. A duplication event and the addition of the tail may then have occurred in the last common ancestor of cnidarians, bilaterians and placozoans, resulting in the coexistence of the two paralogous genes, *pk* and *tes* (Fig. 3b). Nevertheless, only *pk* paralog has been retrieved in Placozoa, which implies that *tes* may subsequently have been lost in this lineage.

As mentioned above, it has been suggested that Tes interacts with VangL2 to regulate inner ear sensory cell orientation in mice [21], which suggests that Pk and Tes may have retained partial functional redundancy, both being involved in planar cell polarity mechanism. Thus, even if the functional role of PkTesL proteins remains to be tested in Porifera and Ctenophora through functional studies, the involvement of both Pk and Tes in cell orientation allows us to propose that it is an ancestral role of the PET family.

### Evolution of the inversin family

**Fig. 4:**
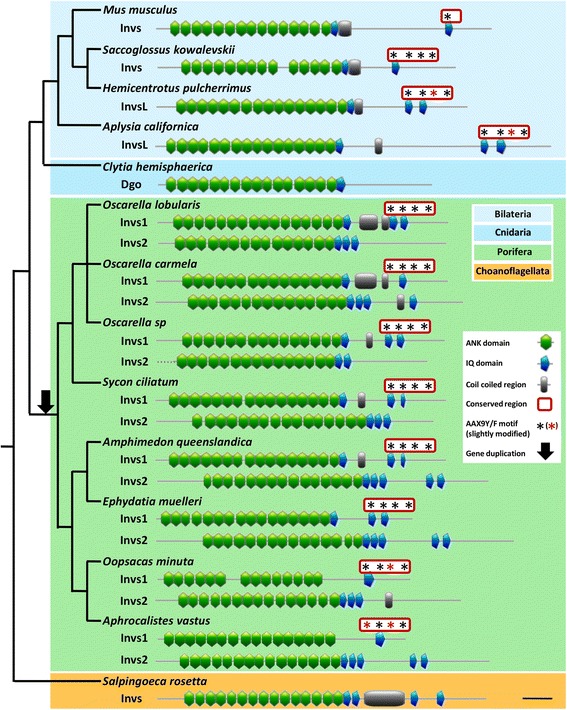
Domain analyses of Inversin proteins revealing their conservation. A duplication event  has occured in Porifera and four AAX9Y/F motifs at the C-terminal end is shared by Ambulacraria (*Hemicentrotus pulcherrimus* and *Saccoglossus kowalevskii* ), Mollusca (*Aplysia californica*) and poriferan Invs1. Scale: 100 amino-acids

No clear orthologous protein of Dgo has been yet reported outside Drosophila. However, Ankrd6 (Diversin) and Inversin proteins found in vertebrates shared homology with the *Drosophila* core PCP gene dgo (repeats of ANK domains). Even if the homology of the two proteins cannot be confidently demonstrated, it has been shown that Ankrd6 can functionally substitute the loss of function of Dgo in Drosophila suggesting that Dgo and Ankrd6 are functional equivalents for planar polarity phenomenon. However, no sequence related to Dgo or Ankrd6 was retrieved in non-bilaterians species. In contrast, the Inversin protein (called Diego in *Clytia*) has been retrieved in different metazoan lineages (Bilateria, Cnidaria and Porifera) and from choanoflagellates, suggesting that it was already present in the last common ancestor of choanoflagellates and metazoans (Table 1, Additional file 1)*.* Nevertheless, no Invs have been found in Placozoa or in Ctenophora. Interestingly, while only one sequence has been described in Bilateria and Cnidaria [23], two copies have been described in the *A. queenslandica* genome [24]. In this study, we retrieved these two copies in all other poriferan species and named them Invs1 and Invs2 so as to avoid phylogenetic confusion with Dgo, which has been only described in *Drosophila* (Table 1 and Additional file 1). The presence of these two copies in Porifera suggests that a duplication event have occurred in the last common ancestor of this lineage (Fig. 4).

**Fig. 5:**
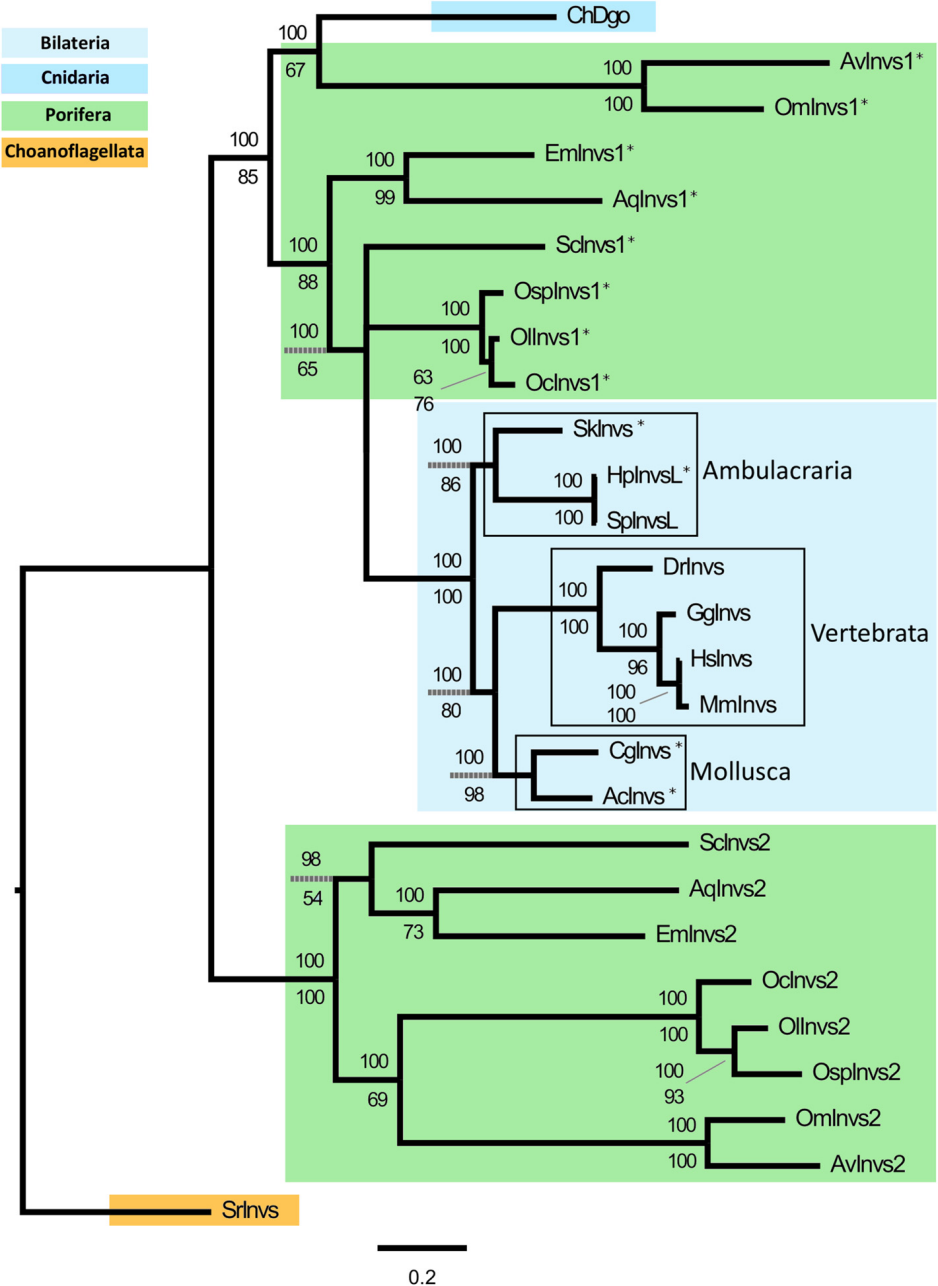
Bayesian-ML consensus tree of Inversin proteins rooted using *Salpingoeca rosetta*. Posterior probability obtained in Bayesian analysis and percentage bootstrap supports obtained in maximum likelihood analysis are indicated above and below each node, respectively. Asterisks correspond to proteins containing the four AAX9/F motifs. Abbreviations. Vertebrate: Dr, *Danio rerio*; Gg, *Gallus gallus*; Hs, *Homo sapiens* and Mm, *Mus musculus.* Mollusca: Ac, *Aplysia californica* and Cg, *Crassotrea gigas*. Ambulacraria: Hp, *Hemicentrotus pulcherrimus*; Sk, *Saccoglossus kowalevskii* and Sp, *Strongylocentrotus purpuratus*. Cnidaria: Ch, *Clytia hemisphaerica.* Porifera: Aq, *Amphimedon queenslandica*; Av, *Aphrocalistes vastus*; Em, *Ephydatia muelleri*; *Oc, Oscarella carmela*; *Ol, Oscarella lobularis*; *Om, Oopsacas minuta*; *Osp, Oscarella sp*.; Sc, *Sycon ciliatum*. Choanoflagellata: Sr, *Salpingoeca rosetta*

According to phylogenic analyses (Fig. 5), it appears that Invs1 is more closely related to Invs proteins found in other animals, whereas Invs2 proteins are much more divergent leading to their basal position (long branch attraction effect). As in bilaterians and cnidarians, all newly identified sequences in sponges and choanoflagellates encode proteins consisting of 14 to 16 ankyrin repeats (ANK) followed by a different number of IQ domains (from 1 to 5) and sometimes a coiled-coil region (Fig. 4). Surprisingly, analyses of these proteins revealed an additional highly conserved region shared by Ambulacraria (e.g. the sea urchin *Hemicentrotus pulcherrimus* and the hemichordate *Saccoglossus kowalevskii*), Mollusca (*Aplysia californica*) and Porifera Invs1 (Fig. 4). A more detailed analysis showed that this conserved C-terminal part (from amino-acid 1148 to 1328 of the full alignment provided in the Additional file 2) encompasses four AAX_9_Y/F motifs. Some of them correspond to predicted IQ domain sites (InterProScan consensus: AX_3_IQFRX_4_KK) (Fig. 4). Nevertheless, no IQ residues could be found within the third and fourth AAX9Y/F motifs (Additional file 2). The ancestrality of this region (containing the four motifs) remains open to question, as the region can be retrieved in these three distant animal lineages but not in vertebrates, cnidarians or poriferan Invs2 paralogs. Therefore, the discovery of this region raises fundamental questions concerning its involvement in protein interaction in concerned taxa. Finally, even if the existence of all functional domains allows us to reliably consider a conserved function of Invs in sponges, further experimental studies are needed to explore the function of both proteins and whether or not Invs acts as a PCP component.

### Potential convergence of In-like protein with metazoan Inturned

We retrieved the In protein in almost all metazoan databases explored here (Table 1 and Additional file 1). Nevertheless, no In was detected in *M. leidyi* genome, suggesting that it is absent from Ctenophores and implying that it may have been secondarily lost by this lineage (depending on the phylogenetic scenario considered).

**Fig. 6:**
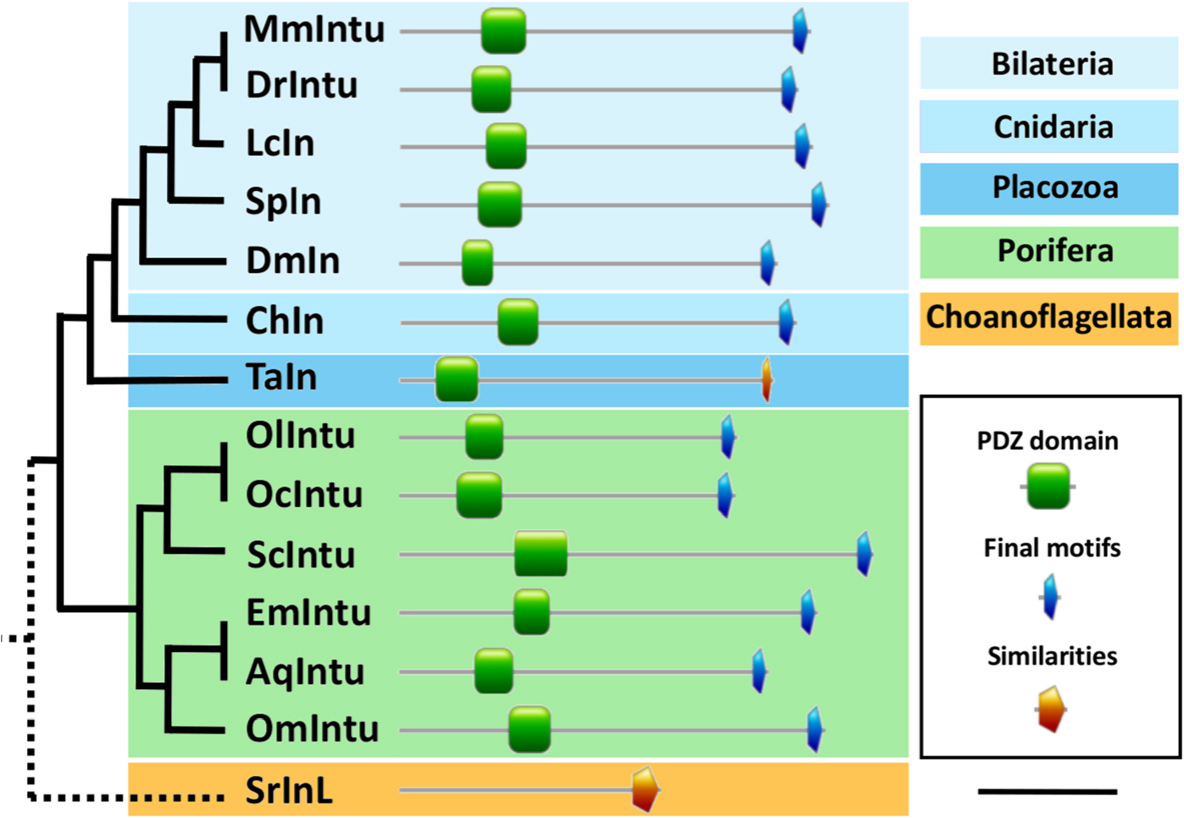
Domain analyses of Inturned proteins highlighting similar motifs in metazoans and choanoflagellates. C-terminal motifs correspond to (Y/F)WX(V/I)GR, EXY(V/I)C(F/Y) and EXAF(R/K)(L/F)XF motifs. C-terminal ends of TaIntu and SrIntu show similarities with other Intu proteins. Final motifs of TaIntu are YWVIG~, ~ ~ ~ ~ Y and EVAHRIGF, where ~ corresponds to one gap. The two final motifs of SrIntuL are ELYVCF and EMAFKFS. Abbreviations. Bilaterians: Dm, *Drosophila melanogaster*; Dr, *Danio rerio*; Lc, *Latimeria chalumnae*; Mm, *Mus musculus* and Sp, *Strongylocentrotus purpuratus*. Cnidaria: Ch, *Clytia hemisphaerica.* Placozoa: Ta, *Trichoplax adhaerens*. Porifera: Aq, *Amphimedon queenslandica*; Em, *Ephydatia muelleri*; *Oc, Oscarella carmela*; *Ol, Oscarella lobularis*; *Om, Oopsacas minuta*; Sc, *Sycon ciliatum*. Choanoflagellata: Sr, *Salpingoeca rosetta*. Scale: 300 amino-acids

Analysis of *in* genes across metazoans (from sponges to vertebrates) confirmed that they encode proteins containing only a PDZ domain (Fig. 6). Moreover, we noticed that the region following the PDZ domain contains well conserved residues, in particular at its C-terminal end (Fig. 6). Indeed, the C-terminal end of In consists in three conserved motifs, (Y/F)WX(V/I)GR, EXY(V/I)C(F/Y) and EXAF(R/K)(L/IF)XF (Additional file 2), calling for functional studies to highlight the role of this region conserved across metazoans.

Although *in* has been considered as a metazoan innovation [11], we obtained conflicting results which made the data difficult to interpret in the present study. Indeed, TBLASTN against the *S. rosetta* genome and reverse TBLASTN against the NCBI database weakly supports (e-value: 0.014) the assignation of PTSG_12265.1 to the In family (Additional file 1). This sequence, called here InL, contains residues which are quite similar (ELYVCF and EMAFKLFS) to those of the two final motifs of In proteins (Additional file 2), suggesting that metazoan In and InL of choanoflagellates may have arisen from an ancestral protein. This suggests that the emergence of *in* dates back to the last common ancestor of choanoflagellates and metazoans. Nevertheless, no PDZ domain was predicted for this sequence and no similarity to the In family was found among the 566 remaining residues of the protein (7.02 % sequence identity) (Additional file 2). In addition, no match for the choanoflagellate InL (cutoff 1.0) was found in the genome of *M. brevicollis*, suggesting that the protein found in *S. rosetta* may represent a convergent feature or that it was lost in *M. brevicollis.*

### Dsh*,* Fmi and Stbm emerged during metazoan evolution

In contrast to other genes, no obvious evidence of *dsh, fmi* or *stbm* has thus far been provided outside metazoans [11]. These genes appear, therefore, to be innovations of the metazoan lineage. The question remains as to when they actually appeared during metazoan evolution. Previous studies have shown that *dsh* belongs to the metazoan ancestral molecular toolkit, being present in all metazoan lineages studied so far (Bilateria, Cnidaria, Placozoa, Porifera and Ctenophora) [26–28, 31]. Nevertheless, no *dsh ortholog* was found in the transcriptomic data of the glass sponge *O. minuta*. Interestingly, a previous study on *Aphrocalistes vastus* (belonging to the same sponge lineage, Hexactinellida) led to the same observation [28], suggesting loss in this lineage.

**Fig. 7:**
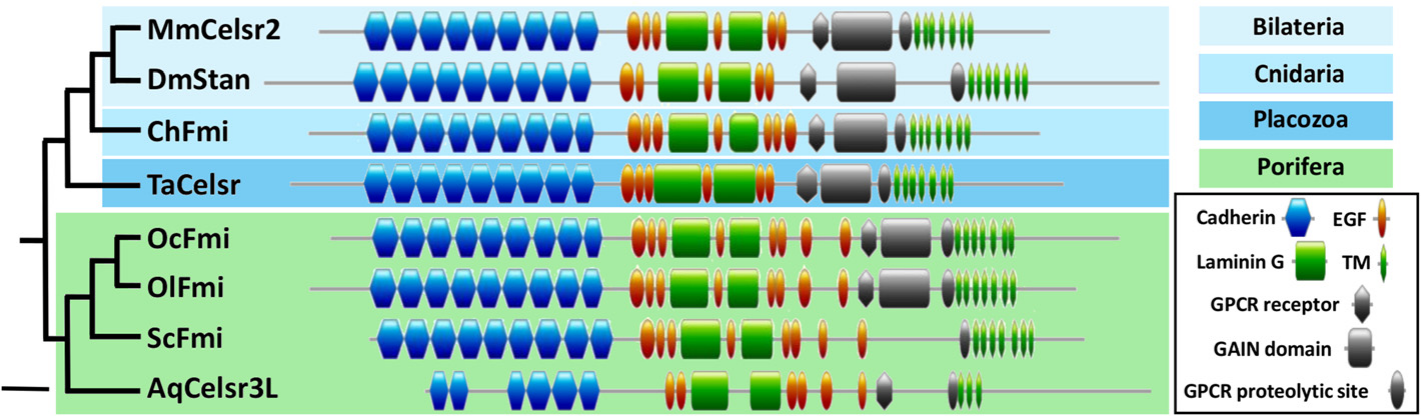
*Bona fide* Flamingo proteins discovered in the *Oscarella* genus (Homoscleromorpha) and in *Trichoplax adhaerens* (Placozoa). Orthologous protein abbreviations. Celsr, Cadherin EGF LAG seven-pass G-type receptor; Stan, Starry night. Species abbreviations. Bilateria: Dm, *Drosophila melanogaster* and Mm, *Mus musculus*. Cnidaria: Ch, *Clytia hemisphaerica*. Placozoa: *Trichoplax adhaerens*. Porifera: *Aq*, *Amphimedon queenslandica*; *Oc, Oscarella carmela*; *Ol, Oscarella lobularis* and *S*c, *Sycon ciliatum*. Scale: 200 amino-acids

In addition, we failed to detect *fmi* in ctenophores (*M. leidyi*), likewise we did not find *fmi* in *A. vastus*, *O. minuta* (Porifera, Hexactinellida) and *Ephydatia muelleri* (Porifera, Demospongiae). In contrast, our identification of an orthologous gene in the two remaining poriferan lineages (Additional file 1), Calcarea and Homoscleromorpha, confirms the results obtained for *A. queenslandica* [33]. However, whereas the Fmi ortholog (AqCelsr3) previously discribed in *A. queenslandica* appears to lack several domains (i.e. the GPCR proteolytic site, three Cadherin domains and four transmembrane domains), the proteins retrieved in Placozoa [34] and Homoscleromorpha (*Oscarella *species) do possess all the characteristic domains of the Flamingo sub-family (including nine Cadherin domains, at least six Epidermal Growth Factor (EGF)-like domains, two Laminin G domains, the family 2 extracellular hormone receptor domain (GPRC), a GAIN domain, a GPCR proteolytic site and the final seven transmembrane domains) (Fig. 7) [34]. Concerning *Sycon ciliatum* Fmi (ScFmi), it is devoid of the GPCR proteolytic site and the GAIN domain, both considered to be involved in autoproteolytic functions (Fig. 7). However, the exact biological function of these autoproteolytic domains remains unknown [35].

**Fig 8:**
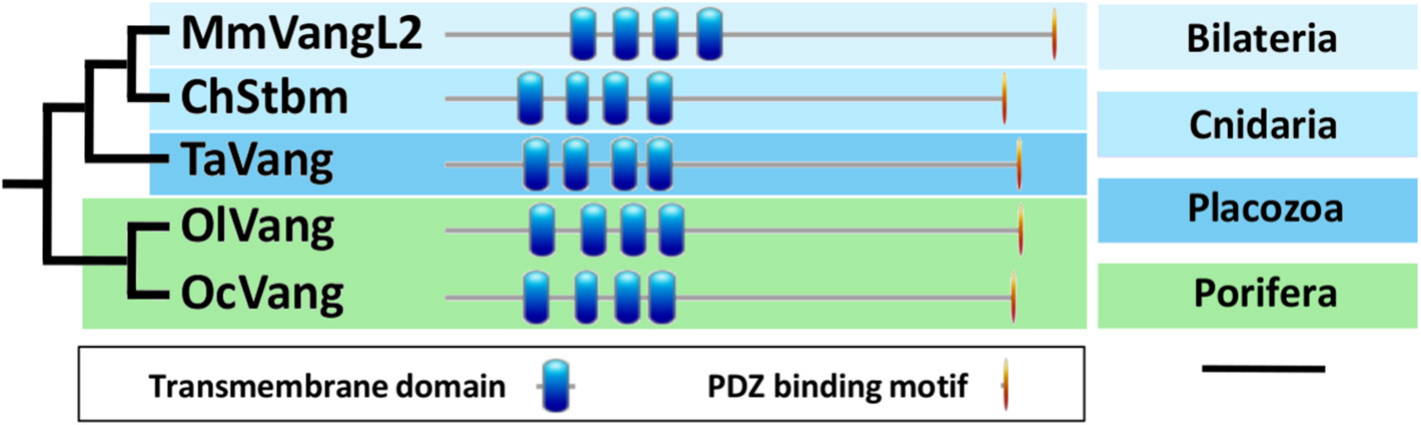
*Bona fide* Van Gogh proteins discovered in *Oscarella* (Porifera) and in *Trichoplax adhaerens* (Placozoa). Orthologous protein abbreviations. Stbm, Strabismus. Species abbreviations. Bilateria: Mm, *Mus musculus*. Cnidaria: Ch, *Clytia hemisphaerica*. Placozoa: Ta, *Trichoplax adhaerens*. Porifera: *Oc, Oscarella carmela*; *Ol, Oscarella lobularis*. Scale: 100 amino-acids

Previous studies have failed to detect *stbm* in any of the basally branching metazoans considered (*A. queenslandica* and *M. leidyi*) [24, 25]. Notably, our study reveals the first poriferan Strabismus genes (called *vang*). Indeed, we evidence a *bona fidestbm* in homoscleromorph sponges (*Oscarella* genus: *O. carmela* genome and transcriptomes of both *O. lobularis* and *O. sp*) (Additional file 1). It encodes a structurally well conserved protein containing the four diagnostic transmembrane domains associated with the C-terminal PDZ binding ETTV motif (Fig. 8 and Additional file 2). In addition, we also confirm that *stbm* is present in the genome of the placozoan *T. adhaerens,* whereas the absence of *stbm* orthologous gene is confirmed in demosponges (*A. queenslandica* and *E. muelleri*) and in ctenophores (*M. leidyi* and *P. pileus*). Finally, we complete previous data by showing that *stbm* is also absent in the hexactinellid sponges (*A. vastus* and *O. minuta*) and in the Calcarea *Sycon ciliatum* (Additional file 1). This gene is thus present in Bilateria, Cnidaria, Placozoa and, unexpectedly, in the *Oscarella* genus (*O. carmela*, *O. lobularis* and *O. sp*).

In summary, the present study shows that *fmi* and *stbm* appear to be absent in ctenophores [25] and hexactinellids whereas sponges belonging to Calcarea and Demospongiae possess only *fmi*. Thus, the homoscleromorph is the only poriferan class that possesses all the genes previously described as the PCP core. This means that both *fmi* and *stbm* were already present in the last common ancestor of Bilateria, Cnidaria, Placozoa and Homoscleromorpha. According to the most recent phylogenetic relationships, showing the monophyly of Porifera and Homoscleromorpha as the sister group of calcareous sponges [25, 36, 37], our findings suggest that the whole set of PCP components was present in the poriferan ancestor and were subsequently lost. The interpretation of the absence of both genes in ctenophores (ancestral absence or losses) depends on the position of this lineage (sister group of all other animals or not).

### The search of the ancestral function of the PCP core

The concept of core PCP module describes a group of *Drosophila* genes that when mutated yield polarity defects. However, it appears that all these genes do not behave in the same way. In fact, one may choose to split them into three or four groups or maybe as many as components of the core. Briefly, the 1) Fmi protein, is totally indispensable, its absence stops any planar signaling (and consequently no polarity can be propagated); 2) Fzd is not indispensable for PCP signaling between two adjacent cells but it is needed for propagation of PCP signaling through several cells; 3) Stbm is necessary for strong intercellular signaling as well as for strong propagation of polarity, and finally 4) a fourth group could correspond to genes which are perfectly dispensable for both signaling and propagation [38, 39]. According to this possible functional interpretation, the keeping of a quintessential core composed of Fzd and Fmi in all poriferan lineages (except in Hexactinellida devoid of Fmi) may reflect a functional constraint. This observation calls for functional experiments on the coordinated beating of oriented cilia in the larva of sponges in order to highlight if whether or not Fzd and Fmi are sufficient to induce planar cell orientation. Nevertheless, the differences of gene content (whole set of genes in Homoscleromorpha, no Vang but the Fzd/Fmi couple in Calcarea and Demospongiae and no Fmi and no Vang in glass sponges) is hardly explained by the presence of oriented cilia in all sponge larvae. Furthermore, observing a coordinated beating of ciliated comb rows in ctenophores while no PCP pathway was identified (no Stbm and no Fmi), also supports that other mechanisms are certainly responsible for the planar orientation of tissues in basally branching animals (e.g. water flow or the Ft-Ds-Fj pathway).

### Limits in gene prediction

Several proteins of the PCP pathway, especially Fmi and Inv, were not retrieved in some databases studied here, implying that they may have been subsequently lost in various lineages. However, it is possible that we were simply not able to identify them due to i) sequence divergence, ii) incomplete databases or iii) because of their composition in highly recurrent domains. For instance, Ankyrin repeats are one of the most common protein-protein interaction motifs in eukaryotes [40–42], resulting in bias in TBLASTN analyses to detect Invs. In addition, if we admit that *diego* is the *Drosophila* functional equivalent of *ankrd6* or *invs* (as proposed in *Clytia*) [18], this means that the encoded protein would have a highly divergent domain composition (only 6 ANK repeats and no IQ domain in Dgo) in addition to a low amino acid conservation, without affecting its function [43].

In addition, the absence of *fmi* in various species will have to be verified against additional databases. *fmi* are long genes split by numerous introns, making more difficult their genomic assembling and identification. For instance, *Scfmi* is a 20 kb genomic sequence consisting of 22 exons for more than 8 kb of coding sequences (Additional file 2). In addition, the numerous Cadherin domains and the high level of divergence of corresponding sequences may still prevent their detection, even when transcriptomic libraries are used.

In contrast, Stbm/Vang is a well-conserved protein that does not contain highly recurrent domains. Furthermore, they are encoded by genes without introns (e.g. *O. carmela* and *T. adharens*). It thus seems very unlikely that the non-prediction of this gene is due to software and algorithmic limitations. In addition, retrieving *vang* in all *Oscarella* databases (including transcriptomes obtained independently by different teams) but not in other sponges (including the genomes of *A. queenslandica* and *S. ciliatum*) or in the *M. leidyi* genome suggests that our results, like previous findings concerning this gene, [24, 25], are not due to the low coverage of databases, and are thus reliable.

### Multigene families as possible characters to support phylogenetic scenarios

**Fig. 9:**
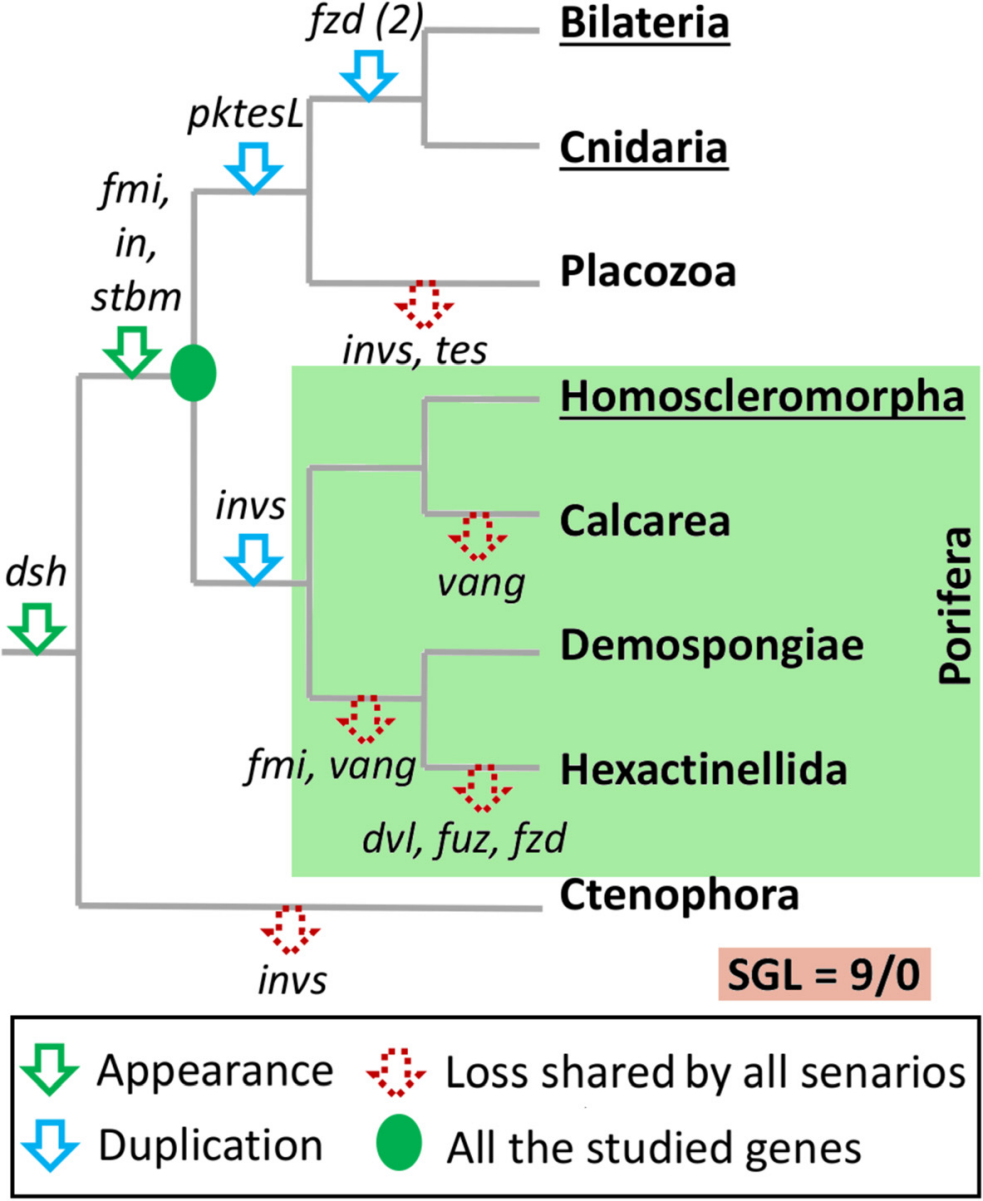
Scenario of emergence/losses of the PCP members during animal evolution. The emergences (green arrows), duplications (blue arrows) and secondary losses (red arrows) of PCP genes support Ctenophora as the sister group of all other metazoans (9 secondary gene losses, SGL) whereas other scenarios are less parsimonious (Additional file 2). None of these nine potential gene losses can be avoid in the other scenario proposed in the literature. Abbreviations. *dsh*/*dvl*, *dishevelled*; *fmi*, *flamingo*; *fuz*, *fuzzy*; *fzd*, *frizzled*; *in*, *inturned*; *invs*, *inversin*; *pktesL*, *prickle-testin like*; *stbm, strabismus; tes*, *testin* and *vang*, *van gogh*

Despite the difficulties involved in accurately evidencing the absence of genes, we have attempted to reconstruct all events concerning the evolution of the PCP pathway on the four currently proposed phylogenies of animals (Fig. 9 and Additional file 2). In doing so, it appears that the most parsimonious scenario (nine gene losses) favors the most recent topology, placing ctenophores as the sister group of all other metazoans (Fig. 9) [25, 36, 44]. Alternative scenarios [36, 37, 45] need a higher number of secondary gene losses (14 to 16) compared to the most recent topology (Additional file 1). Nevertheless, many cases showing that the most parsimonious scenario is not always the most reliable are already known. Indeed, among the best-known examples, we can note the status of Urochordata within Chordata [46]. Hence, at this stage of research where so many additional genomic data are needed, this evolutionary exercise remains speculative. However, recent work has shown how the study of multigene families may unravel the evolution of complex relationships when phylogenetic analyses are not congruent [47]. Applying similar reasoning to multigene families of the PCP pathway seems to confirm that i) Porifera is a monophyletic group (since they all share two paralogous genes of *invs*) (Figs. 4 and 9); ii) Cnidaria is the sister group of Bilateria (according to Fzd duplications, [32]) and iii) bilaterians, cnidarians and placozoans share a common ancestor (according to the presence of *pk* paralogs) (Figs. 3 and 9). The question of whether the most basal lineage should be attributed to ctenophores or to sponges remains unresolved. It is to be hoped that the accumulation of such data concerning the evolution of multigene families will help to decipher animal relationships.

## Conclusion

By examining a large dataset of non bilaterian metazoans, the present study challenges previous statements concerning the origin of the Planar Cell Polarity pathway. Although previous studies of the genomes of *M. leidyi* and *A. queenslandica* suggested that the PCP pathway arose in the last common ancestor of Bilateria, Cnidaria and Placozoa, our results allow us to state that this pathway dates back at least to the emergence of Porifera. More specifically, we describe here *bona fide* Stbm/Vang and Fmi proteins in sponges from the *Oscarella* genus (Homoscleromorpha class), whereas other lineages lack few players of the PCP pathway. This discovery spotlights the retrieval of a unique, complete PCP pathway - in Homoscleromorph sponges - and thus calls for functional studies in this particular sponge lineage to investigate the mechanisms involved in the coordination of cell orientation in a non planulozoan lineage (Bilateria + Cnidaria). Nevertheless, whether or not the absence of key proteins means that no PCP mechanisms occurs in other sponge lineages also requires investigation. The present study provides yet another strong affirmation that each sponge lineage is of high interest to gain a better understanding of the evolution of key molecular toolkits in metazoans. Finally, this study highlights how the accumulation of data concerning the evolution of multigene families may help us to trace early metazoan evolution when no phylogenetic consensus is currently accepted.

## Methods

The main components of the Planar Cell Polarity pathway were researched in different transcriptomic or genomic databases: Demospongiae (*Amphimedon queenslandica* and *Ephydatia muelleri*), Hexactinellida (*Aphrocalistes vastus* and *Oopsacas minuta*), Calcarea (*Sycon ciliatum*), Homoscleromorpha (*Oscarella lobularis*, *Oscarella carmela* and *Oscarella sp*.), *Mnemiopsis leidyi* (Ctenophora), *Trichoplax adhaerens* (Placozoa) and *Salpingoeca rosetta* (Choanoflagellata). Most of these databases are available online (Additional file 1). When genes were absent from *Mnemiopsis leidyi* or *Salpingoeca rosetta*, we investigated the closely related species *Pleurobrachia pileus* and *Monosiga brevicollis,* respectively*.*

We searched proteins of interest by local TBALSTN analyses using BioEdit software and *Mus musculus* sequences [48]. In order to retrieve highly divergent sequences, a 1.0 threshold e-value was fixed. The relevance of our results was then tested using a reciprocal best hit approach [49] against the NCBI database (nr/nt) (with no restriction to a specific taxa) and via protein domain analyses using InterProScan5 (http://www.ebi.ac.uk/Tools/pfa/iprscan5/). When no orthologous sequences were obtained using *Mus musculus* sequences, similar methodology was applied using *Clytia hemisphaerica* (Cnidarian) or *Amphimedon queenslandica* (Porifera) sequences [23, 24].

Since the Prickle/Testin and Inversin/Diego families are multigene families, phylogenetic analyses were performed for sequence assignation to each orthologous group. Protein alignments were carried out using the ClustalW function of BioEdit [50]. Alignments were then controlled visually and slightly improved before being cut to conserve only functional domains (i.e. LIM domains for the Prickle/Testin family and ANK domains for Invs proteins) (full alignments are available in Additional file 1). Maximum likelihood (ML) analyses were performed using the online PhyML software (www.atgc-montpellier.fr) [51] and branch supports were estimated using 1000 bootstrap re-sampling (all other default parameters were conserved). Bayesian analyses were performed using MrBayes v3.2.2 [52] with one cold chain and three heated chains until the average deviation of split frequencies was below 0.01 (100 000 generations). As recommended by ProtTest 3.2 [53], both ML and Bayesian analyses were performed using the WAG substitution model.

## Additional files

Additional file 1: BLAST analyses. (PDF 497 kb) Additional file 2: Sequence analyses. (PDF 2200 kb)
