# Supplementary material for: Retracing the path of planar cell polarity
Source: BMC Evol Biol. 2016 Apr 2;16:69. doi: 10.1186/s12862-016-0641-0 (PMC4818920; doi:10.1186/s12862-016-0641-0)
Supplement: Additional file 1: — BLAST analyses. (PDF 497 kb) [file 12862_2016_641_MOESM1_ESM.pdf]

ADDITIONAL FILE 1: BLAST analyses

Table S1.1: Database information…………………………………………………………………………………………p2

Table S1.2: Proteins found in *Amphimedon queenslandica* (Porifera, Demospongiae)…………..p2

Table S1.3: Proteins found in *Aphrocalistes vastus* (Porifera, Hexactinellida)…………….………..p2

Table S1.4: Proteins found in *Ephydatia muelleri* (Porifera, Demospongiae)…………….…………p3

Table S1.5: Proteins found in *Mnemiopsis leidyi* (Ctenophora)……………………………………..……p3

Table S1.6: Proteins found in *Oopsacas minuta* (Porifera, Hexactinellida)……………….…………..p4

Table S1.7: Proteins found in *Oscarella carmela* (Porifera, Homoscleromorpha)……….…….……p4

Table S1.8: Proteins found in *Oscarella lobularis* (Porifera, Homoscleromorpha)……….…….……p5

Table S1.9: Proteins found in *Oscarella sp.* (Porifera, Homoscleromorpha)…………….….…………p6

Table S1.10: Proteins found in *Salpingoeca rosetta* (Choanoflagellata)………………….…….…………p7

Table S1.11: Proteins found in *Sycon ciliatum* (Porifera, Calcarea)……………………….………………p7

Table S1.12: Proteins found in *Tricoplax adhaerens* (Placozoa)……………………………….…….…..p8

Table S1.1: Database information.

Lineage	Species	Genome	Transcroptome
Choanoflagellata	*Salpingoeca rosetta*	Broad institute	Not available
Ctenophora	*Mnemiopsis leidyi*	NHGRI	Compagen
	Pleurobrachia pileu	Not available	Compagen
Placozoa	*Trichoplax adhaerens*	Ensembl	Compagen
Porifera	*Amphimedon queenslandica*	Ensembl	Not available
	Aphrocalistes vastus	Not available	Ualberta
	Ephydatia muelleri	Not available	Compagen
	Oopsacas minuta	Not available	Personal database
	Oscarella carmela	Compagen	Compagen
	Oscarella lobularis	Not available	Personal database
	Oscarella sp.	Not available	Compagen
	Sycon ciliatum	Compagen	Compagen

Websites: Broad institute, http://www.broadinstitute.org/; Compagen, http://www.compagen.org/; Ensembl, http://metazoa.ensembl.org/index.html; NHGRI, http://research.nhgri.nih.gov/mnemiopsis/ and Ualberta, https://era.library.ualberta.ca/public/home.

Table S1.2: Proteins found in *Amphimedon queenslandica* (Porifera, Demospongiae).

Gene	Accession or Contig	Tblastn top hit
Celsr3L	Aqu1.224307	Fahey and Degnan, 2010
Dvl	NP_001266242.1 (NCBI)	Adamska et al., 2010
Fuz	contig13228	Xenopus tropicalis Fuz<br>(2e-35; NM_001040017.2)
Fzd	NP_001266206.1 (NCBI)<br>NP_001266202.1 (NCBI)	Adamska et al., 2010
Intu	Aqu1.217322<br>Aqu1.214741<br>Aqu1.208411	Adamska et al., 2010
Invs1 (Dgo)	Aqu1.221107 (completed)	Adamska et al., 2010
Invs2 (Invs)	Aqu1.227557	Adamska et al., 2010
PkTesL	Aqu1.217773	Adamska et al., 2010
Vang	Not found	Adamska et al., 2010

Table S1.3: Proteins found in *Aphrocalistes vastus* (Porifera, Hexactinellida).

Gene	Accession or Contig	Tblastn top hit
Dvl	Not found	
Fmi	Not found	
Fuz	No hit	
Fzd	No accession	Riesgo et al., 2014
Intu	comp54332_c0_seq1<br>comp64270_c0_seq1<br>comp2131_c0_seq1	Echinops telfairi Intu<br>(4e-16; XM_004711858.1)
Invs1	comp15586_c0_seq1	Saccoglossus kowalevskii Invs<br>(1e-101; XM_006819037.1)
Invs2	comp34481_c0_seq1	Amphimedon queenslandica InvsL<br>(8e-73; XM_011411529.1)
PkTesL	comp27734_c0_seq1	Saccoglossus kowalevskii Pk2L<br>(2e-96; GU224218.1)
Vang	No hit	

Table S1.4: Proteins found in *Ephydatia muelleri* (Porifera, Demospongiae).

Gene	Accession or Contig	Tblastn top hit
Dvl	comp70341_c0_seq1	Amphimedon queenslandica Dsh<br>(0.0; NM_001279313.1)
Fmi	Not found	
Fuz	comp58048_c0	Saccoglossus kowalevskii Fuz<br>(1e-86; XM_006815707.1)
Fzd	AJE25513.1<br>AJE25514.1	Schenkelaars et al., 2015
Intu	comp68133_c0	Sorex araneus Intu<br>(8e-60; XM_004606283.1)
Invs1	comp54435_c0	Saccoglossus kowalevskii Invs<br>(0.0; XM_006819037.1)
Invs2	comp66288_c0	Saccoglossus kowalevskii Invs<br>(2e-109; GU211213.1)
PkTesL	comp22862_c0	Amphimedon queenslandica TesL<br>(2e-139; XM_003387784.1)
Vang	Not found	

Table S1.5: Proteins found in *Mnemiopsis leidyi* (Ctenophora).

Gene	Accession or Contig	Tblastn top hit
Dsh	HM448818 (NCBI)	Pang et al., 2010
Fmi	Not found	Ryan et al., 2013
Fy	ML0181	Poecilia reticulata Fuz<br>(6e-10; XM_008434537.1)
Fzd	HM448819 (NCBI)<br>HM448820 (NCBI)	Pang et al., 2010
In	No hit	
Invs	Not found	
PkTesL	ML2189	Latimeria chalumnae Pk1<br>(2e-71; XM_005993176.1)
Vang	Not found	Ryan et al., 2013

Table S1.6: Proteins found in *Oopsacas minuta* (Porifera, Hexactinellida).

Gene	Accession or Contig	Tblastn top hit
Dvl	Not found	
Fmi	Not found	
Fuz	No hit	
Fzd	AJE25510.1 (NCBI)	Schenkelaars et al., 2015
Intu	KT898945	Sorex araneus Intu<br>(5e-53; XM_004606283.1)
Invs1	KT898946	Saccoglossus kowalevskii Invs<br>(1e-124; XM_006819037.1)
Invs2	KT898947	Amphimedon queenslandica InvsL<br>(5e-65; XM_011411529.1)
PkTesL	KT898948	Saccoglossus kowalevskii Pk2L<br>(6e-92; GU224218.1)
Vang	No hit	

Table S1.7: Proteins found in *Oscarella carmela* (Porifera, Homoscleromorpha).

Gene	Accession or Contig	Tblastn top hit
Dsh	comp44599_c0_seq1	Lytechinus variegatus Dsh (6e-134; AY624074.1)
Fmi	comp27089_c0_seq1	Xenopus tropicalis Celsr3 (0.0; XM_002933077.2)
Fuz	comp7117_c0_seq1	Saccoglossus kowalevskii Fuz (5e-72; XM_006815707.1)
Fzd	AJE25507.1 (NCBI)<br>AJE25508.1 (NCBI)<br>AJE25509.1 (NCBI)	Schenkelaars et al., 2015
Intu	comp39366_c0_seq9	Egretta garzetta Intu (2e-81; XM_009642227.1)
Invs1	comp36587_c0_seq1	Saccoglossus kowalevskii Invs (0.0; GU211213.1)
Invs2	comp33916_c0_seq2	Saccoglossus kowalevskii Invs (7e-80; GU211213.1)
PkTesL	comp26691_c0_seq1	Saccoglossus kowalevskii Pk2L (3e-112; GU224218.1)
Vang	comp31109_c0_seq5<br>scaffold12158	Saccoglossus kowalevskii Stbm (2e-61; GU076129.1)

Table S1.8: Proteins found in *Oscarella lobularis* (Porifera, Homoscleromorpha).

Gene	Accession or Contig	Tblastn top hit
Dvl	KT898949	Lytechinus variegatus Dsh (1e-92; AY62407.1)
Fmi	KT898950	Xenopus tropicalis Celsr3 (0.0; XM_002933077.2)
Fuz	KT898951	Saccoglossus kowalevskii Fuz (1e-45; XM_006815707.1)
Fzd	AJE25504.1 (NCBI)<br>AJE25505.1 (NCBI)<br>AJE25506.1 (NCBI)	Schenkelaars et al., 2015
Intu	KT898952	Callorhinchus milii Intu (3e-61; XM_007910682.1)
Invs1	KT898953	Saccoglossus kowalevskii Invs (0.0; GU211213.1)
Invs2	KT898954	Saccoglossus kowalevskii Invs (1e-85; GU211213.1)
PkTesL	KT898955	Saccoglossus kowalevskii Pk2L (6e-117; GU224218.1)
Vang	KT898956	Hydra magnipapillata VangL1 (7e-51; XM_002160130.2)

Table S1.9: Proteins found in *Oscarella sp.* (Porifera, Homoscleromorpha).

Gene	Accession or Contig	Tblastn top hit
Dvl	comp18085_c0_seq1<br>comp27893_c0_seq1<br>comp18203_c0_seq1<br>comp30186_c0_seq1<br>comp30334_c0_seq1<br>comp75723_c0_seq1	Tribolium castaneum Dvl3<br>(1e-118; XM_009068758.1)
Fmi	Not found	
Fuz	comp156056_c0_seq1<br>comp76968_c0_seq1<br>comp61620_c0_seq1	Saccoglossus kowalevskii Fuz<br>(1e-07; XM_006815707.1)
Fzd	comp13598_c0_seq1<br>comp20726_c0_seq1	Oscarella lobularis FzdB<br>(0.0; KM365031.1)
	comp16628_c0_seq1	Oscarella lobularis FzdA2<br>(4e-156; KM365030.1)
	comp4918_c0_seq1	Oscarella carmela FzdA1<br>(0.0; KM365032.1)
Intu	comp29063_c0_seq1<br>comp77813_c0_seq1<br>comp114481_c0_seq1<br>comp103590_c0_seq1<br>comp37720_c0_seq1<br>comp74551_c0_seq1<br>comp91853_c0_seq1<br>comp82399_c0_seq1<br>comp90976_c0_seq1<br>comp180374_c0_seq1<br>comp75621_c0_seq1<br>comp61285_c0_seq1<br>comp133579_c0_seq1<br>comp38913_c0_seq1	Nannospalax galili Intu<br>(2e-16; XM_008823687.1)
Invs1	comp5479_c0_seq1	Saccoglossus kowalevskii Invs<br>(0.0; GU211213.1)
Invs2	comp14868_c0_seq1	Crassotrea gigas InvsL<br>(1e-85; XM_011416660.1)
PkTesL	comp4801_c0_seq1	Saccoglossus kowalevskii Pk2L<br>(1e-119; GU224218.1)
Vang	comp32065_c0_seq1<br>comp70883_c0_seq1	Musca domestica VangL2<br>(2e-59; XM_005190441.1)

Table S1.10: Proteins found in *Salpingoeca rosetta* (Choanoflagellata).

Gene	Accession or Contig	Tblastn top hit
Fuz	PTSG_02764.1	Anolis carolinensis Fuz (4e-41; XM_003222723.1)
InL	PTSG_12265.1	Capra hircus Intu (0.014; XM_005691271,1)
Invs	PTSG_12654.1	Saccoglossus kowalevskii Invs (2e-102; GU211213.1)
PkTesL	PTSG_10035.1	Saccoglossus kowalevskii Pk2L (4e-86; GU224218.1)

Table S1.11: Proteins found in *Sycon ciliatum* (Porifera, Calcarea).

Gene	Accession or Contig	Tblastn top hit
Dvl	CDO67914.1 (NCBI)<br>CDO67915.1 (NCBI)	Leininger et al., 2014
Fmi	sctid1773	Chrysemys picta bellii Celsr2 (0.0; XM_008176079.1)
Fuz	sctid38004	Saccoglossus kowalevskii Fuz (2e-101; XM_006815707.1)
Fzd	CDO67909.1 (NCBI)<br>CDO67910.1 (NCBI)<br>CDO67911.1 (NCBI)<br>CDO67912.1 (NCBI)	Leininger et al., 2014
Intu	sctid17446	Sarcophilus harrisii Intu (1e-86; XM_003772754.1)
Invs1	sctid33564	Saccoglossus kowalevskii Invs (2e-81; GU211213.1)
Invs2	sctid23651	Saccoglossus kowalevskii Invs (0.0; XM_006819037.1)
PkTesL	sctid62610	Saccoglossus kowalevskii Pk2L (4e-86; GU224218.1)
Vang	Not found	

Table S1.12: Proteins found in *Tricoplax adhaerens* (Placozoa).

Gene	Accession or Contig	Tblastn top hit
Celsr	XM_002118173.1 (NCBI)	Hulpiau and van Roy, 2011
Dsh	No accession	Scrivastava et al., 2008
Fy	Scaffold 1	Saccoglossus kowalevskii Fuz (6e-64; XM_002108011.1)
Fzd	AJE25511.1 (NCBI)<br>AJE25512.1 (NCBI)	Schenkelaars et al., 2015
In	Scaffold 5	Taeniopygia guttata Intu (1e-64, XM_004176709.1)
Invs	Not found	
Pk	Scaffold 5	Saccoglossus kowalevskii Pk2L (2e-126; GU224218.1)
Vang	Scaffold 1	Apis dorsata VangL2 (5e-81; XM_001630370.1)
