# Supplementary material for: Retracing the path of planar cell polarity
Source: BMC Evol Biol. 2016 Apr 2;16:69. doi: 10.1186/s12862-016-0641-0 (PMC4818920; doi:10.1186/s12862-016-0641-0)
Supplement: Additional file 2: — Sequence analyses. (PDF 2200 kb) [file 12862_2016_641_MOESM2_ESM.pdf]

ADDITIONAL FILE 2: Sequence analyses

Table S2.1: Flamingo/Celsr/Stan sequence identity matrix……………………………………………………...p2

Table S2.2: Fuzzy sequence identity matrix ………………………………………………………………………..p3

Table S2.3: Inturned sequence identity matrix……………………………………………………………………….p4

Table S2.4: Inversin sequence identity matrix………………………………………………………………………p6

Table S2.5: PET family sequence identity matrix……………………………………………………………………p10

Table S2.6: Strabismus/Van Gogh Stan sequence identity matrix …………………………………………p12

Figure S2.1: Flamingo/Celsr/Stan alignment………………………………………………………………………..p13

Figure S2.2: Fuzzy alignment……………………………………………………………………………………………...p32

Figure S2.3: Inturned alignment………………………………………………………………………………………….p34

Figure S2.4: Inversin alignment…………………………………………………………………………………………….p40

Figure S2.5: PET family alignement……………………………………………………………………………………..p52

Figure S2.6: Strabismus/Van Gogh alignement……………………………………………………………………p66

Figure S2.7: Maximum likelihood unrooted tree of PET family………………………………………………p68

Figure S2.8: Bayesian unrooteed tree of PET family……………………………………………………………..p69

Figure S2.9: Intro/exon composition of *flamingo* in *Sycon ciliatum*…………………………………………p70

Figure S2.10: Alternative scenario 1 of the PCP players during animal evolution……………………p71

Figure S2.11: Alternative scenario 2 of the PCP players during animal evolution……………………p71

Figure S2.12: Alternative scenario 3 of the PCP players during animal evolution……………………p72

Table S2.1: Flamingo/Celsr/Stan sequence identity matrix. Obtained using bioedit software.

Seq->	MmCelsr1	DrCelsr1b	DrCelsr1a	MmCelsr2	DrCelsr2	MmCelsr3	DrCelsr3
MmCelsr1	ID	0,596	0,609	0,539	0,52	0,431	0,262
DrCelsr1b	0,596	ID	0,702	0,516	0,528	0,427	0,267
DrCelsr1a	0,609	0,702	ID	0,522	0,535	0,431	0,264
MmCelsr2	0,539	0,516	0,522	ID	0,605	0,442	0,269
DrCelsr2	0,52	0,528	0,535	0,605	ID	0,43	0,257
MmCelsr3	0,431	0,427	0,431	0,442	0,43	ID	0,351
DrCelsr3	0,262	0,267	0,264	0,269	0,257	0,351	ID
DmStan	0,29	0,287	0,285	0,279	0,286	0,284	0,161
ChFmi	0,235	0,241	0,248	0,234	0,25	0,207	0,127
TaCelsr	0,264	0,269	0,271	0,257	0,255	0,25	0,137
OcFmi	0,217	0,215	0,213	0,212	0,213	0,203	0,116
OlFmi	0,227	0,219	0,225	0,216	0,222	0,198	0,102
ScFmi	0,184	0,181	0,178	0,194	0,179	0,173	0,099
AqCelsr3L	0,127	0,129	0,136	0,131	0,128	0,122	0,092

Seq->	DmStan	ChFmi	TaCelsr	OcFmi	OlFmi	ScFmi	AqCelsr3L
MmCelsr1	0,29	0,235	0,264	0,217	0,227	0,184	0,127
DrCelsr1b	0,287	0,241	0,269	0,215	0,219	0,181	0,129
DrCelsr1a	0,285	0,248	0,271	0,213	0,225	0,178	0,136
MmCelsr2	0,279	0,234	0,257	0,212	0,216	0,194	0,131
DrCelsr2	0,286	0,25	0,255	0,213	0,222	0,179	0,128
MmCelsr3	0,284	0,207	0,25	0,203	0,198	0,173	0,122
DrCelsr3	0,161	0,127	0,137	0,116	0,102	0,099	0,092
DmStan	ID	0,192	0,225	0,181	0,184	0,151	0,113
ChFmi	0,192	ID	0,214	0,185	0,197	0,159	0,117
TaCelsr	0,225	0,214	ID	0,202	0,199	0,17	0,126
OcFmi	0,181	0,185	0,202	ID	0,545	0,197	0,132
OlFmi	0,184	0,197	0,199	0,545	ID	0,196	0,126
ScFmi	0,151	0,159	0,17	0,197	0,196	ID	0,127
AqCelsr3L	0,113	0,117	0,126	0,132	0,126	0,127	ID

Abbreviations. Vertebrate: Dr, *Danio rerio* and Mm, *Mus musculus.* Hexapoda: Dm, *Drosophila melanogaster.* Cnidaria: Ch, *Clytia hemisphaerica.* Placozoa: Ta, *Trichoplax adhaerens*. Porifera: Aq, *Amphimedon queenslandica*; *Oc, Oscarella carmela*; *Ol, Oscarella lobularis*; Sc, *Sycon ciliatum*.

Table S2.2: Fuzzy sequence identity matrix. Obtained using bioedit software.

Seq->	DrFuz	MmFuz	DmFy	ChFy	TaFy	OcFuz	OlFuz
DrFuz	ID	0,436	0,219	0,245	0,23	0,272	0,248
MmFuz	0,436	ID	0,216	0,244	0,201	0,247	0,224
DmFy	0,219	0,216	ID	0,166	0,168	0,214	0,22
ChFy	0,245	0,244	0,166	ID	0,162	0,18	0,161
TaFy	0,23	0,201	0,168	0,162	ID	0,185	0,181
OcFuz	0,272	0,247	0,214	0,18	0,185	ID	0,587
OlFuz	0,248	0,224	0,22	0,161	0,181	0,587	ID
OspFuz	0,082	0,075	0,071	0,097	0,065	0,144	0,174
ScFuz	0,262	0,273	0,195	0,173	0,228	0,309	0,259
AqFuz	0,211	0,219	0,168	0,182	0,162	0,222	0,199
EmFuz	0,275	0,275	0,209	0,18	0,262	0,291	0,263
MlFy	0,153	0,14	0,132	0,116	0,146	0,168	0,182
SrFy	0,195	0,215	0,158	0,146	0,144	0,196	0,19

Seq->	OspFuz	ScFuz	AqFuz	EmFuz	MlFy	SrFy
DrFuz	0,082	0,262	0,211	0,275	0,153	0,195
MmFuz	0,075	0,273	0,219	0,275	0,14	0,215
DmFy	0,071	0,195	0,168	0,209	0,132	0,158
ChFy	0,097	0,173	0,182	0,18	0,116	0,146
TaFy	0,065	0,228	0,162	0,262	0,146	0,144
OcFuz	0,144	0,309	0,222	0,291	0,168	0,196
OlFuz	0,174	0,259	0,199	0,263	0,182	0,19
OspFuz	ID	0,09	0,116	0,077	0,055	0,084
ScFuz	0,09	ID	0,248	0,341	0,171	0,224
AqFuz	0,116	0,248	ID	0,308	0,11	0,194
EmFuz	0,077	0,341	0,308	ID	0,152	0,193
MlFy	0,055	0,171	0,11	0,152	ID	0,099
SrFy	0,084	0,224	0,194	0,193	0,099	ID

Abbreviations. Vertebrate: Dr, *Danio rerio* and Mm, *Mus musculus.* Hexapoda: Dm, *Drosophila melanogaster.* Cnidaria: Ch, *Clytia hemisphaerica.* Placozoa: Ta, *Trichoplax adhaerens*. Porifera: Aq, *Amphimedon queenslandica*; Em, *Ephydatia muelleri*; *Oc, Oscarella carmela*; *Ol, Oscarella lobularis*; *Osp, Oscarella sp.*; Sc, *Sycon ciliatum*. Ctenophora: Ml, *Mnemiopsis leidyi*. Choanoflagellata: Sr, *Salpingoeca rosetta*.

Table S2.3: Inturned sequence identity matrix. Obtained using bioedit software.

Seq->	MmIntu	DrIntu	DmIn	SpIn	LcIn	SmIn	ChIn
MmIntu	ID	0,477	0,161	0,328	0,572	0,183	0,19
DrIntu	0,477	ID	0,161	0,321	0,512	0,184	0,196
DmIn	0,161	0,161	ID	0,157	0,146	0,119	0,129
SpIn	0,328	0,321	0,157	ID	0,312	0,178	0,183
LcIn	0,572	0,512	0,146	0,312	ID	0,173	0,176
SmIn	0,183	0,184	0,119	0,178	0,173	ID	0,117
ChIn	0,19	0,196	0,129	0,183	0,176	0,117	ID
TaIn	0,172	0,185	0,135	0,174	0,169	0,129	0,152
OspIntu	0,142	0,139	0,081	0,134	0,126	0,109	0,128
OcIntu	0,222	0,233	0,145	0,208	0,21	0,14	0,19
OlIntu	0,211	0,216	0,137	0,185	0,186	0,129	0,18
ScIntu	0,199	0,185	0,123	0,193	0,197	0,125	0,169
EmIntu	0,2	0,174	0,133	0,178	0,17	0,112	0,173
AqIntu	0,179	0,17	0,126	0,163	0,161	0,1	0,152
AvIntu	0,085	0,079	0,057	0,078	0,086	0,069	0,075
OmIntu	0,172	0,167	0,12	0,157	0,171	0,121	0,153
SrInL	0,081	0,096	0,087	0,076	0,07	0,062	0,078

Seq->	TaIn	OspIntu	OcIntu	OlIntu	ScIntu	EmIntu	AqIntu
MmIntu	0,172	0,142	0,222	0,211	0,199	0,2	0,179
DrIntu	0,185	0,139	0,233	0,216	0,185	0,174	0,17
DmIn	0,135	0,081	0,145	0,137	0,123	0,133	0,126
SpIn	0,174	0,134	0,208	0,185	0,193	0,178	0,163
LcIn	0,169	0,126	0,21	0,186	0,197	0,17	0,161
SmIn	0,129	0,109	0,14	0,129	0,125	0,112	0,1
ChIn	0,152	0,128	0,19	0,18	0,169	0,173	0,152
TaIn	ID	0,122	0,163	0,152	0,149	0,145	0,135
OspIntu	0,122	ID	0,384	0,44	0,148	0,124	0,111
OcIntu	0,163	0,384	ID	0,632	0,222	0,183	0,186
OlIntu	0,152	0,44	0,632	ID	0,197	0,177	0,175
ScIntu	0,149	0,148	0,222	0,197	ID	0,178	0,144
EmIntu	0,145	0,124	0,183	0,177	0,178	ID	0,224
AqIntu	0,135	0,111	0,186	0,175	0,144	0,224	ID
AvIntu	0,05	0,232	0,073	0,074	0,072	0,066	0,06
OmIntu	0,12	0,117	0,173	0,159	0,172	0,153	0,146
SrInL	0,073	0,078	0,096	0,092	0,068	0,079	0,083

Seq->	AvIntu	OmIntu	SrInL
MmIntu	0,085	0,172	0,081
DrIntu	0,079	0,167	0,096
DmIn	0,057	0,12	0,087
SpIn	0,078	0,157	0,076
LcIn	0,086	0,171	0,07
SmIn	0,069	0,121	0,062
ChIn	0,075	0,153	0,078
TaIn	0,05	0,12	0,073
OspIntu	0,232	0,117	0,078
OcIntu	0,073	0,173	0,096
OlIntu	0,074	0,159	0,092
ScIntu	0,072	0,172	0,068
EmIntu	0,066	0,153	0,079
AqIntu	0,06	0,146	0,083
AvIntu	ID	0,245	0,037
OmIntu	0,245	ID	0,076
SrInL	0,037	0,076	ID

Abbreviations. Vertebrate: Dr, *Danio rerio;* Lc, Latimeria chalumnae and Mm, *Mus musculus.* Hexapoda: Dm, *Drosophila melanogaster.* Ambulacraria. Sp, *Strongylocentrotus purpuratus*. Cnidaria: Ch, *Clytia hemisphaerica;* Nv, *Nematostella vectensis.* Placozoa: Ta, *Trichoplax adhaerens*. Porifera: Aq, *Amphimedon queenslandica*; Av, *Aphrocalistes vastus*; Em, *Ephydatia muelleri*; *Oc, Oscarella carmela*; *Ol, Oscarella lobularis*; *Om, Oopsacas minuta*; *Osp, Oscarella sp.*; Sc, *Sycon ciliatum*. Ctenophora: Ml, *Mnemiopsis leidyi*. Choanoflagellata: Sr, *Salpingoeca rosetta*.

Table S2.4: Inversin sequence identity matrix. Obtained using bioedit software.

Seq->	MmInvs	DrInvs	HpInvsL	SkInvs	AcInvs	ChDgo	OcInvs1
MmInvs	ID	0,514	0,341	0,359	0,308	0,211	0,281
DrInvs	0,514	ID	0,346	0,356	0,313	0,21	0,291
HpInvsL	0,341	0,346	ID	0,52	0,364	0,211	0,362
SkInvs	0,359	0,356	0,52	ID	0,388	0,228	0,408
AcInvs	0,308	0,313	0,364	0,388	ID	0,182	0,305
ChDgo	0,211	0,21	0,211	0,228	0,182	ID	0,223
OcInvs1	0,281	0,291	0,362	0,408	0,305	0,223	ID
OlInvs1	0,289	0,293	0,364	0,411	0,306	0,221	0,863
OspInvs1	0,283	0,286	0,361	0,408	0,304	0,223	0,808
ScInvs1	0,257	0,277	0,346	0,367	0,287	0,229	0,472
AqInvs1	0,227	0,239	0,29	0,322	0,244	0,217	0,392
EmInvs1	0,242	0,257	0,338	0,354	0,268	0,218	0,425
AvInvs1	0,182	0,175	0,215	0,251	0,176	0,171	0,26
OmInvs1	0,178	0,186	0,224	0,255	0,197	0,182	0,283
OcInvs2	0,165	0,177	0,197	0,208	0,162	0,146	0,212
OlInvs2	0,175	0,191	0,185	0,194	0,158	0,145	0,215
OspInvs2	0,183	0,191	0,188	0,194	0,154	0,138	0,215
ScInvs2	0,158	0,166	0,166	0,183	0,138	0,148	0,183
AqInvs2	0,173	0,179	0,192	0,195	0,16	0,152	0,206
EmInvs2	0,197	0,199	0,188	0,203	0,16	0,175	0,212
AvInvs2	0,154	0,162	0,156	0,171	0,132	0,151	0,177
OmInvs2	0,157	0,168	0,166	0,185	0,143	0,154	0,185
SrInL	0,196	0,196	0,21	0,222	0,177	0,187	0,22

Seq->	OlInvs1	OspInvs1	ScInvs1	AqInvs1	EmInvs1	AvInvs1	OmInvs1
MmInvs	0,289	0,283	0,257	0,227	0,242	0,182	0,178
DrInvs	0,293	0,286	0,277	0,239	0,257	0,175	0,186
HpInvsL	0,364	0,361	0,346	0,29	0,338	0,215	0,224
SkInvs	0,411	0,408	0,367	0,322	0,354	0,251	0,255
AcInvs	0,306	0,304	0,287	0,244	0,268	0,176	0,197
ChDgo	0,221	0,223	0,229	0,217	0,218	0,171	0,182
OcInvs1	0,863	0,808	0,472	0,392	0,425	0,26	0,283
OlInvs1	ID	0,863	0,466	0,391	0,438	0,263	0,286
OspInvs1	0,863	ID	0,466	0,389	0,427	0,26	0,288
ScInvs1	0,466	0,466	ID	0,359	0,393	0,25	0,283
AqInvs1	0,391	0,389	0,359	ID	0,483	0,282	0,304
EmInvs1	0,438	0,427	0,393	0,483	ID	0,297	0,295
AvInvs1	0,263	0,26	0,25	0,282	0,297	ID	0,481
OmInvs1	0,286	0,288	0,283	0,304	0,295	0,481	ID
OcInvs2	0,219	0,209	0,206	0,197	0,188	0,175	0,177
OlInvs2	0,222	0,212	0,198	0,189	0,2	0,173	0,175
OspInvs2	0,224	0,208	0,198	0,195	0,218	0,172	0,185
ScInvs2	0,182	0,186	0,188	0,194	0,199	0,16	0,165
AqInvs2	0,205	0,2	0,201	0,184	0,205	0,17	0,166
EmInvs2	0,217	0,211	0,215	0,196	0,197	0,159	0,153
AvInvs2	0,174	0,174	0,178	0,161	0,162	0,152	0,15
OmInvs2	0,187	0,183	0,202	0,178	0,191	0,167	0,166
SrInL	0,226	0,221	0,225	0,202	0,227	0,168	0,169

Seq->	OcInvs2	OlInvs2	OspInvs2	ScInvs2	AqInvs2	EmInvs2	AvInvs2
MmInvs	0,165	0,175	0,183	0,158	0,173	0,197	0,154
DrInvs	0,177	0,191	0,191	0,166	0,179	0,199	0,162
HpInvsL	0,197	0,185	0,188	0,166	0,192	0,188	0,156
SkInvs	0,208	0,194	0,194	0,183	0,195	0,203	0,171
AcInvs	0,162	0,158	0,154	0,138	0,16	0,16	0,132
ChDgo	0,146	0,145	0,138	0,148	0,152	0,175	0,151
OcInvs1	0,212	0,215	0,215	0,183	0,206	0,212	0,177
OlInvs1	0,219	0,222	0,224	0,182	0,205	0,217	0,174
OspInvs1	0,209	0,212	0,208	0,186	0,2	0,211	0,174
ScInvs1	0,206	0,198	0,198	0,188	0,201	0,215	0,178
AqInvs1	0,197	0,189	0,195	0,194	0,184	0,196	0,161
EmInvs1	0,188	0,2	0,218	0,199	0,205	0,197	0,162
AvInvs1	0,175	0,173	0,172	0,16	0,17	0,159	0,152
OmInvs1	0,177	0,175	0,185	0,165	0,166	0,153	0,15
OcInvs2	ID	0,656	0,561	0,228	0,253	0,237	0,223
OlInvs2	0,656	ID	0,653	0,211	0,263	0,224	0,214
OspInvs2	0,561	0,653	ID	0,216	0,236	0,206	0,203
ScInvs2	0,228	0,211	0,216	ID	0,232	0,242	0,183
AqInvs2	0,253	0,263	0,236	0,232	ID	0,316	0,254
EmInvs2	0,237	0,224	0,206	0,242	0,316	ID	0,23
AvInvs2	0,223	0,214	0,203	0,183	0,254	0,23	ID
OmInvs2	0,243	0,244	0,225	0,203	0,254	0,248	0,559
SrInL	0,196	0,2	0,184	0,178	0,191	0,201	0,179

Seq->	OmInvs2	SrInL
MmInvs	0,157	0,196
DrInvs	0,168	0,196
HpInvsL	0,166	0,21
SkInvs	0,185	0,222
AcInvs	0,143	0,177
ChDgo	0,154	0,187
OcInvs1	0,185	0,22
OlInvs1	0,187	0,226
OspInvs1	0,183	0,221
ScInvs1	0,202	0,225
AqInvs1	0,178	0,202
EmInvs1	0,191	0,227
AvInvs1	0,167	0,168
OmInvs1	0,166	0,169
OcInvs2	0,243	0,196
OlInvs2	0,244	0,2
OspInvs2	0,225	0,184
ScInvs2	0,203	0,178
AqInvs2	0,254	0,191
EmInvs2	0,248	0,201
AvInvs2	0,559	0,179
OmInvs2	ID	0,182
SrInL	0,182	ID

Abbreviations. Vertebrate: Dr, *Danio rerio* and Mm, *Mus musculus.* Hexapoda: Dm, *Drosophila melanogaster.* Mollusca: Ac, *Aplysia californica* and Cg, *Crassotrea gigantea*. Ambulacraria: Hp, *Hemicentrotus pulcherrimus*; Sk and *Saccoglossus kowalevskii*. Cnidaria: Ch, *Clytia hemisphaerica.* Porifera: Aq, *Amphimedon queenslandica*; Av, *Aphrocalistes vastus*; Em, *Ephydatia muelleri*; *Oc, Oscarella carmela*; *Ol, Oscarella lobularis*; *Om, Oopsacas minuta*; *Osp, Oscarella sp.*; Sc, *Sycon ciliatum*. Choanoflagellata: Sr, *Salpingoeca rosetta*.

Table S2.4: PET family sequence identity matrix. Obtained using bioedit software.

Seq->	MmPkL1	MmPkL2	MmPkL3	DmPk	DmEsn	ChPk	TaPk
MmPkL1	ID	0,484	0,292	0,198	0,249	0,24	0,215
MmPkL2	0,484	ID	0,323	0,193	0,247	0,225	0,19
MmPkL3	0,292	0,323	ID	0,174	0,259	0,255	0,269
DmPk	0,198	0,193	0,174	ID	0,303	0,162	0,127
DmEsn	0,249	0,247	0,259	0,303	ID	0,226	0,211
ChPk	0,24	0,225	0,255	0,162	0,226	ID	0,215
TaPk	0,215	0,19	0,269	0,127	0,211	0,215	ID
MmTes	0,117	0,115	0,188	0,081	0,13	0,144	0,169
DmTes	0,078	0,075	0,118	0,06	0,082	0,099	0,103
ChTes	0,136	0,129	0,196	0,086	0,139	0,152	0,185
OcPkTesL	0,138	0,141	0,237	0,098	0,161	0,178	0,204
OlPkTesL	0,147	0,148	0,246	0,104	0,172	0,187	0,207
OspPkTesL	0,153	0,151	0,247	0,104	0,172	0,19	0,202
ScPkTesL	0,126	0,126	0,199	0,092	0,143	0,156	0,183
AqPkTesL	0,13	0,13	0,198	0,093	0,138	0,159	0,167
EmPkTesL	0,133	0,13	0,212	0,09	0,139	0,163	0,171
AvPkTesL	0,128	0,127	0,21	0,092	0,148	0,16	0,193
OmPkTesL	0,129	0,127	0,209	0,1	0,15	0,16	0,189
MlPkTesL	0,142	0,14	0,196	0,092	0,151	0,156	0,162
SrPkTesL	0,083	0,079	0,124	0,064	0,092	0,097	0,109

Seq->	MmTes	DmTes	ChTes	OcPkTesL	OlPkTesL	OspPkTesL	ScPkTesL
MmPkL1	0,117	0,078	0,136	0,138	0,147	0,153	0,126
MmPkL2	0,115	0,075	0,129	0,141	0,148	0,151	0,126
MmPkL3	0,188	0,118	0,196	0,237	0,246	0,247	0,199
DmPk	0,081	0,06	0,086	0,098	0,104	0,104	0,092
DmEsn	0,13	0,082	0,139	0,161	0,172	0,172	0,143
ChPk	0,144	0,099	0,152	0,178	0,187	0,19	0,156
TaPk	0,169	0,103	0,185	0,204	0,207	0,202	0,183
MmTes	ID	0,173	0,345	0,337	0,329	0,335	0,25
DmTes	0,173	ID	0,167	0,165	0,171	0,169	0,142
ChTes	0,345	0,167	ID	0,38	0,396	0,41	0,301
OcPkTesL	0,337	0,165	0,38	ID	0,763	0,787	0,392
OlPkTesL	0,329	0,171	0,396	0,763	ID	0,835	0,377
OspPkTesL	0,335	0,169	0,41	0,787	0,835	ID	0,38
ScPkTesL	0,25	0,142	0,301	0,392	0,377	0,38	ID
AqPkTesL	0,282	0,134	0,301	0,382	0,391	0,387	0,322
EmPkTesL	0,289	0,146	0,344	0,427	0,428	0,438	0,332
AvPkTesL	0,255	0,141	0,314	0,399	0,419	0,424	0,307
OmPkTesL	0,244	0,149	0,319	0,406	0,419	0,413	0,302
MlPkTesL	0,243	0,109	0,247	0,3	0,297	0,304	0,257
SrPkTesL	0,15	0,111	0,154	0,19	0,182	0,183	0,205

Seq->	AqPkTesL	EmPkTesL	AvPkTesL	OmPkTesL	MlPkTesL	SrPkTesL
MmPkL1	0,13	0,133	0,128	0,129	0,142	0,083
MmPkL2	0,13	0,13	0,127	0,127	0,14	0,079
MmPkL3	0,198	0,212	0,21	0,209	0,196	0,124
DmPk	0,093	0,09	0,092	0,1	0,092	0,064
DmEsn	0,138	0,139	0,148	0,15	0,151	0,092
ChPk	0,159	0,163	0,16	0,16	0,156	0,097
TaPk	0,167	0,171	0,193	0,189	0,162	0,109
MmTes	0,282	0,289	0,255	0,244	0,243	0,15
DmTes	0,134	0,146	0,141	0,149	0,109	0,111
ChTes	0,301	0,344	0,314	0,319	0,247	0,154
OcPkTesL	0,382	0,427	0,399	0,406	0,3	0,19
OlPkTesL	0,391	0,428	0,419	0,419	0,297	0,182
OspPkTesL	0,387	0,438	0,424	0,413	0,304	0,183
ScPkTesL	0,322	0,332	0,307	0,302	0,257	0,205
AqPkTesL	ID	0,465	0,327	0,337	0,243	0,165
EmPkTesL	0,465	ID	0,376	0,377	0,277	0,175
AvPkTesL	0,327	0,376	ID	0,665	0,251	0,173
OmPkTesL	0,337	0,377	0,665	ID	0,254	0,171
MlPkTesL	0,243	0,277	0,251	0,254	ID	0,142
SrPkTesL	0,165	0,175	0,173	0,171	0,142	ID

Abbreviations. Vertebrate: Mm, *Mus musculus.* Hexapoda: Dm, *Drosophila melanogaster.* Cnidaria: Ch, *Clytia hemisphaerica.* Placozoa: Ta, *Trichoplax adhaerens*. Porifera: Aq, *Amphimedon queenslandica*; Av, *Aphrocalistes vastus*; Em, *Ephydatia muelleri*; *Oc, Oscarella carmela*; *Ol, Oscarella lobularis*; *Om, Oopsacas minuta*; *Osp, Oscarella sp.*; Sc, *Sycon ciliatum*. Ctenophora: Ml, *Mnemiopsis leidyi*. Choanoflagellata: Sr, *Salpingoeca rosetta*.

Table S2.6: Strabismus/Van Gogh/Stan sequence identity matrix. Obtained using bioedit software.

Seq->	MmVangL1	DrVangL1	MmVangL2	DrVangL2	DmVang	ChStbm	TaVang
MmVangL1	ID	0,648	0,708	0,644	0,422	0,366	0,321
DrVangL1	0,648	ID	0,628	0,608	0,397	0,365	0,305
MmVangL2	0,708	0,628	ID	0,768	0,414	0,373	0,309
DrVangL2	0,644	0,608	0,768	ID	0,413	0,363	0,31
DmVang	0,422	0,397	0,414	0,413	ID	0,287	0,267
ChStbm	0,366	0,365	0,373	0,363	0,287	ID	0,332
TaVang	0,321	0,305	0,309	0,31	0,267	0,332	ID
OcVang	0,272	0,271	0,252	0,259	0,251	0,277	0,294
OlVang	0,27	0,276	0,253	0,255	0,245	0,274	0,286
OspVang	0,244	0,251	0,23	0,228	0,219	0,238	0,212

Seq->	OcVang	OlVang	OspVang
MmVangL1	0,272	0,27	0,244
DrVangL1	0,271	0,276	0,251
MmVangL2	0,252	0,253	0,23
DrVangL2	0,259	0,255	0,228
DmVang	0,251	0,245	0,219
ChStbm	0,277	0,274	0,238
TaVang	0,294	0,286	0,212
OcVang	ID	0,75	0,564
OlVang	0,75	ID	0,605
OspVang	0,564	0,605	ID

Abbreviations. Vertebrate: Dr, *Danio rerio* and Mm, *Mus musculus.* Hexapoda: Dm, *Drosophila melanogaster.* Cnidaria: Ch, *Clytia hemisphaerica.* Placozoa: Ta, *Trichoplax adhaerens*. Porifera: *Oc, Oscarella carmela*; *Ol, Oscarella lobularis*; *Om, Oopsacas minuta*; *Osp, Oscarella sp*.

Figure S2.1: Flamingo/Celsr/Stan alignment. Threshold for shading has been fixed at 80% for both identity (black) and similarity (grey).

```
                      10        20        30        40        50        60        70
             ....|....|....|....|....|....|....|....|....|....|....|....|....|....|
MmCelsr1     --------------MAPSSPRVLPALVLLAAAALPALELGAAAWELRVPGGARAFALGPGWSYRLDTTRT
DrCelsr1b    --------------MELPVIWMCFG-LLLHLSPFGCLDL-HLSETLKPGANLANVSIGPEWTYRIDRSLT
DrCelsr1a    --------------MDLVMCWLLWVHLLLCAPLMGCFDL-HLSETLHSGAVLANLSLGPGWTYKIDWTLT
MmCelsr2     ----------------------------------------------------MRTRAA---SAPLPTPLL
DrCelsr2     ----------------MKGDLLLFVFGCFELAGLVDCFSVHVSEDAGPGTAIAELQRG---SCALDQLLL
MmCelsr3     MARRPLWWGLPGPSTPVLLLLLLSLFPFSREELGGGGDQDWDPGVATTTGPRAQIGSGAVALCPESPGVW
DrCelsr3     ----------------------------------------------------------------------
DmStan       -----MQTREFPQRPLGLLLVLLVVLLQSSLIKSYLIIVHEDTPPGTVIFNASVYKLGSERHYKINAHKS
ChFmi        ----------------MKLRWLEEEVFKLQRYFMILFLFSTVKDVITTDTQFHNPVQDLDQKCKLLDLSQ
TaCelsr      --------MSSLNFIYRLMCYIIAISQLCASCQGIIVHIQDNLKHDTVIVDLNRQLNSVQADYQLLPLGN
OcFmi        ----------------------------------------------------------------------
OlFmi        --------------------MRILRGARDETTLRSRNPIHLCSAVESLIFNAQHSPNGCRYPVKSERSRV
ScFmi        ----------------------------------------------------------------------
AqCelsr3L    ----------------------------------------------------------------------

                      80        90       100       110       120       130       140
             ....|....|....|....|....|....|....|....|....|....|....|....|....|....|
MmCelsr1     PRELLDVSREGPAAGRRLGLGAGTLG------------------CARLAGRLLPLQVRLVARGAPTAPSL
DrCelsr1b    DKSFRHFLKIGRSDG--LLLLARKFD------------------CARLPRNPVPMYLRIKPQLSENFILL
DrCelsr1a    SKYIGNFIKVDWRDG--VVRLNGEVD------------------CARAPHSALPLYFKIKSLTTEGLVKT
MmCelsr2     PLLLLLLLLPP--SP--LLGDQVGP-------------------CRSLGS---------GGRSSSGACAP
DrCelsr2     PRFAETFLRPD--AG--VLTLSNAPN------------------CDSVLSNPFSVYAVVDCKDASGFRHL
MmCelsr3     EDGDPGLGVREPVFMRLRVGRQNARN------------------GRGAPEQPNAEVVVQALGSREQEAGQ
DrCelsr3     ----------------------------------------------------------------------
DmStan       ANFVHHLVSVNHKDGQIQLRKALKCDGIYYPNLFTFYVDSTSNRLRSIDYYSLPVRIFVSGHSCNEDRRI
ChFmi        LFVGWDYYTLDQEANNENNELTLEHG------------------------SLFSSDCLNKKSDRTICFKV
TaCelsr      NHAENNLFTIHAKTGKMITRKDIRCS------------------DLKSNPLSYLIRLRPTGLSSQKLTHL
OcFmi        --------------------------------------------MHGTLAFII--LLLSVFSSWAAPCAG
OlFmi        GRDSLGLALLGFCAAGDQGDVAQRSW-----------------IMHRTIGIICGVLFASILSSSAEQCAS
ScFmi        ----------------------------------------------------------------------
AqCelsr3L    ----------------------------------------------------------------------

                     150       160       170       180       190       200       210
             ....|....|....|....|....|....|....|....|....|....|....|....|....|....|
MmCelsr1     VLRARAYGARCGVRLLRRSARGAELRSPAVRSVPGLGDALCFPAAGGGAASLTSVLEAITNFPACSCPPV
DrCelsr1b    HFNTFVHGQNCYIKYKRKNLTP----DAYMYVVTGYDSCLSLDSLPINIYESLPVSMRNSQMFRGRCVEK
DrCelsr1a    HFNVFLHGQNCVFKFKRKRETKPDI-NVNYIYNSGLTSCLSPGDLLIHFEDYLPVFTRNSAVFASSCTSD
MmCelsr2     VG--WLCPASASNLWLYTSRCRESGIELTGHLVPHHDGLRVWCPESG---AHIPLPPSSEGCPWSCRLLG
DrCelsr2     VT--SRYDVHVHGKNCASTRKRENKLDVEIISLTESSECREAHSAVFRVFDVLPGNPARCNVTRTSAFYM
MmCelsr3     GPGYLLCWHPEISSCGRTGPLRRGSLPLDALSPGDSDLRNSSPHPSELLAQPDGSRPVAFQRNARRSIRK
DrCelsr3     ----------------------------------------------------------------------
DmStan       EQELHHHHYEEEDNTGYSKRRRRRSTQEMIQLNGNQLEEVFRQNSTEFRAGDLIFGDSFDNEMRHRILSR
ChFmi        VKNLVNKYSFIDGKICLTTLPGSEDHNKITTSSKTFFNLFIPFPWRDQYVNIKQIISNNRTSVECINVPE
TaCelsr      NKRLIPITIYTHGSRCRLQDARKKIINTNFATLNHQGEAYLFLAEIFPDVALKRIVGQVEPVERSAQRHI
OcFmi        VVRLNTS-AWLSSDWRFIGSVRMEDIQRPSQPNTYRFKAMRRRFFAKEQGTLLPCFRVKSAAPGRVASAL
OlFmi        VLRLNAAISRPSSDWHLIGAIRTADALKGLRIAAADRNATRIRLFAVEKQSALTCFRVLPAGLGQIR-LF
ScFmi        ----------------------------------------------------------------------
AqCelsr3L    ----------------------------------------------------------------------
```

```
                220       230       240       250       260       280       280
           ....|....|....|....|....|....|....|....|....|....|....|....|....|....|
MmCelsr1   AGTGCRRGPICLRPGGSAELRLVCALGRAAGAVWVELVIEATSGTPSESPSVSPSLLNLSQPRAGVVRRS
DrCelsr1b  TQ----RRVSPLTN-----RCLPHNQ-RDEANLICLMPNSVNS-SLYVDVKLHLNRKYTGSDFDPWAKRQ
DrCelsr1a  QLNVFVRNGTLLVKDA---YCLETNLGRFKSTIRCSFLDSKGE-PFSVCLKLRFTSDQTSLSYVTNPRKQ
MmCelsr2   IGGHLSPQGTLTLPEEHP--CLKAPRLRCQSCKLAQAPGLRAGEG---------------SPEESLGGRR
DrCelsr2   SDGSLFTSARLCFTRDS----LLEFQMRCGGRAGAAHAHWRVGRGPFTQTHLSKVLRLAARSNSGLLSRR
MmCelsr3   RVETSRCCGKLWEPGHKGQGERSATSTVDRGPFRRDCLPGSLGSGLGEDSAPRAVRTAPTPGSAPRESRT
DrCelsr3   ----------------------------------------------------------------------
DmStan     KRRAVGSPDPLHLQPALHRRISDAKQWISETYASYAIHTTDKWNQICLRRSQFINSLNAFLPRSVCQHCK
ChFmi      NTVRLQKDGTLYINKNVLHEPIKSIKCYLKRNGSNSKFKLIIQFENDFEVLVR-----------------
TaCelsr    VLTPISKRIVKIYCKALTDPSKTASLCSRPFHLQVVTRYQRSLNVNFAKIHLMTDYQINTNHDRQNQKVI
OcFmi      RKQSLLYVESRCNSTAV----TAEDNTRRFTLRMVEISTGGRRRETCVDVVVTFESVGRPRTYGGGSRPL
OlFmi      GTESLLYVETRCSN-------QTTAKRREFELRVAATGRTGRR-QACVRVDVDYTHHSAVSHYDAR-AIS
ScFmi      ---------------------MAGVERIWFSVLVVVLFTFSPR---------------------------
AqCelsr3L  ----------------------------------------------------------------------

                290       300       310       320       330       340       350
           ....|....|....|....|....|....|....|....|....|....|....|....|....|....|
MmCelsr1   RR--------------------------------------------------------------------
DrCelsr1b  ----------------------------------------------------------------------
DrCelsr1a  RI--------------------------------------------------------------------
MmCelsr2   ----------------------------------------------------------------------
DrCelsr2   ----------------------------------------------------------------------
MmCelsr3   APGRMRSRGLFRRRFLFERPGPRPPGFPTGPEAKQILST-------------------------------N
DrCelsr3   ----------------------------------------------------------------------
DmStan     VSFLDVNDERFAIEHQSRDLVASRDVCIAESMWKVSITFNIRCDRRDIVDSDHRLKIVYHHQEFNDTDIA
ChFmi      ----------------------------------------------------------------------
TaCelsr    NHHQRHQLVEWYNRLPSKNNPILEHHRN-----------------------------------------R
OcFmi      RR--------------------------------------------------------------------
OlFmi      RL--------------------------------------------------------------------
ScFmi      ----------------------------------------------------------------------
AqCelsr3L  ----------------------------------------------------------------------

                360       370       380       390       400       410       420
           ....|....|....|....|....|....|....|....|....|....|....|....|....|....|
MmCelsr1   ----GTGSSTSPQFPLPSYQVSVPENEPAGTAVIELRAHDP--DEGDAGRLSYQMEALFDERSNGYFLID
DrCelsr1b  ----KRNVNSAPQFQLPNYQVSVPENEPSGTRVITLKAFDA--DDGDAGVVVYDMEALFDSRSNNLFQIN
DrCelsr1a  ----KRAANSVPQFQLSNYQVSVPENEPAGTRVITLKATVS--DPGETGKIEYGMEALFDSRSNDFFRID
MmCelsr2   ----KRNVNTAPQFQPPSYQATVPENQPAGTSVASLRAIDP--DEGEAGRLEYTMDALFDSRSNHFFSLD
DrCelsr2   ----RRNVNNNPQFHPPMYQVSVPENQPAGTFVVVLKAVDP--DEGEAGRLEYVLEALFDSRSNNLFAVD
MmCelsr3   QARPRRAANRHPQFPQYNYQTLVPENEAAGTSVLRVVAQDP--DPGEAGRLIYSLAALMNSRSLELFSID
DrCelsr3   ----------------------------------------------------------------------
DmStan     RRVRRELRNQSPYFEQALYVASVLEEQPAGAAVTTVRARDP--EDS---PVVYSMVSLLDSRSQSLFKVD
ChFmi      -----RRRNIPIYFEKSLYSVDIFEDTKRDSVIRKLKVNGG--------SGKYSFSKTEDPKTDILFKVK
TaCelsr    RSIRSKRDVIAPVFTQSSYSSSIFENEPSGTTVLTVSVRD-----KSAPGLTYSMAAVGDLRSNSLFGIN
OcFmi      ---RRAVNQNAPKFTKTTYNFAVSENQSPGMFVGEISANDP--DAGDAGVVVYSMKFLNDVRTEGKFEID
OlFmi      ---KRAVNRNAPKFTKQSYDFQVTENLNAGTFVGKVSADDA--DTGSAGAVVYSMRFLHDANTAGKFVID
ScFmi      ------PVSCENRFSQLTYTFTVSEDTPVKTSIGQVVLTDASGKELAVSAARYSISARGVLRSVGLFTVR
AqCelsr3L  ----------------------------------------------------------------------
```

```
                   430       440       450       460       470       480       490
            ....|....|....|....|....|....|....|....|....|....|....|....|....|....|
MmCelsr1    AATGAVTTARSLDRET-KDTHVLKVSAVD---HGSPRRSAATYLTVTVSDTNDHSPVFEQSEYRERIREN
DrCelsr1b   PETGGITTLQPLDREV-KDTHVFKVTATD---KGIPKRSAIAYLTITVSDTNDHSPVFEQNEYRVNVREN
DrCelsr1a   PQTGSIETAQPLDREV-KDTHVFKVTASV---AGFAKRAATSYLTITVSDTNDHGPVFEQNEYRVRIREN
MmCelsr2    PITGVVTTAEELDRET-KSTHVFRVTAQD---HGMPRRSALATLTILVTDTNDHDPVFEQQEYKESLREN
DrCelsr2    PASGVVSTVEILDRET-KDTHVFRVTAVD---HGTPRRTAMATLTVTVSDTNDHDPAFEQQDYKESIREN
MmCelsr3    PQSGLIRTAAALDRES-MERHYLRVTAQD---HGSPRLSATTMVAVTVADRNDHAPVFEQAQYRETLREN
DrCelsr3    ----------------------------------------------------------------------
DmStan      SRTGVVTTSASLDREL-MDVHYFRVVATD---DSFPPRSGTTTLQVNVLDCNDHSPTFEAEQFEASIREG
ChFmi       SDTGDVILLQELNREDGAERFSIDIRAID---LNDPSNSATTTIRVKVLDVNDNAPTFAESSYSITVDEG
TaCelsr     SVSGAIYTVNYLDREE-IAKHQFTVTATVN--DGTTTLFSRVSVTIDVLDRNDNAPRFESPSYQVSIPED
OcFmi       ATTGKITTRVALNREK-MDKHEFEVKAVD---KGSPKFEGKASLVIRVLDENDNNPVFDRLSYTKSIYEN
OlFmi       ASNGEIRTKVPLDREK-MNRHEFELTARD---NGSPQLEGKASLVIRVLDANDNSPVFDDASYSETIRED
ScFmi       -EDGHVVIAAALDRES-LEQHEYSLVGKYITRAHNVWQRADCTIVVNLLDVNDSPPKFENDVRTVGILEG
AqCelsr3L   -------------------------------------MLAGERIGMLIYIDTYTLLIGQEILQLSVVDNT

                   500       510       520       530       540       550       560
            ....|....|....|....|....|....|....|....|....|....|....|....|....|....|
MmCelsr1    LEVGYEVLTIRATDGDAPSNANMRYRLLEG--AG--------GVFEIDARSGVVRTRAVVDREEAAEYQL
DrCelsr1b   VEVGFEVMTIRATDGDAPSNANMIYKIVNDDEVN--------SCFEIDPRNGLVRTKVRPDREVKSQYKL
DrCelsr1a   VEVGFEVITVRATDGDAPSNANMIYRIMNEEGVN--------SGFEIDSWNGLVKIKVRPDRETMAQYQL
MmCelsr2    LEVGYEVLTVRATDGDAPPNANILYRLLEGAGDSPS------DAFEIDPRSGVIRTRGPVDREEVESYKL
DrCelsr2    LEIGYEVLTVRATDGDAPINGNILYHLLNSNGTN--------DVFEIDSRSGVIRTRGLVDRETVDSYML
MmCelsr3    VEEGYPILQLRATDGDAPPNANLRYRFVGSPAVRTAAA----AAFEIDPRSGLISTSGRVDREHMESYEL
DrCelsr3    ----------------------------------------------------------------------
DmStan      ATVGSTVITLRATDQDIGKNAEIEYGIEAVTDGAGLAQDQEMPIFRIDSRSGVISTRSSLDRETSDSYHL
ChFmi       QVANKYILTVRATDADEGSNQEIRYSIVNKANVQVP--------FQIDPISGAIKSTDVLDRETHPYYNF
TaCelsr     IPIGTTALQVRAQDNDASSNARVTYSIVNSAGIN--------SAFRVGVTSGAIIIDRSLDREITDRYHL
OcFmi       VAVGHQAEEVHATDIDFGVNGSLVYSLLSTGNINGA--------FTIVSSSGQIKTAKSLDRETVPRYVL
OlFmi       VDVGHQAGEVHATDLDTGSNADVIYSLVG--NTDDA--------FVVTSTTGRILTTKSLDRETIAVYSF
ScFmi       EQVDRAVADMVVSDADLGTNGDISFAILQASVPN---------TFYMDSSEGLVLTSAALDRETIAQYNI
AqCelsr3L   SHVLYEILSTQSSDDDFYLSP-----------------------------NGFLYTARMLNKSQTPLYNL

                   570       580       590       600       610       620       630
            ....|....|....|....|....|....|....|....|....|....|....|....|....|....|
MmCelsr1    LVEANDQGRNPGPL-SASATVHIVVEDENDNYPQFSEKRYVVQVPED---VAVNTAVLRVQATDRDQGQN
DrCelsr1b   FVEANDQGREPGPR-TATATVHIFIEDENDNYPQFSEKRYVVQVSEN---IAVNTQVAVVKATDKDAGNN
DrCelsr1a   IVEASDQGKEPGPR-SATATVHITVEDENDNYPQFSEKRYVVQVPEN---VAVNTKVAQVEATDRDEGNN
MmCelsr2    TVEASDQGRDPGPR-SSTAIVFLSVEDDNDNAPQFSEKRYVVQVRED---VTPGAPVLRVTASDRDKGSN
DrCelsr2    MVEANDQGRDPGPR-SATATVHIVVEDDNDNAPQFSEKRYIVQVPED---LTPNTEILQVTATDQDRGNN
MmCelsr3    VVEASDQGQEPGPR-SATVRVHITVLDENDNAPQFSEKRYVAQVRED---VRPHTVVLRVTATDKDKDAN
DrCelsr3    ----------------------------------------------------------------------
DmStan      LVTAADLASAQSERRTATASVQVKVLDDNDNYPQFSERTYTVQVPEDQWGGTEDNTVAHIRATDADQGNN
ChFmi       TVRAEDQGKVKLHT---DVSVSITLRDKNDNSPKFSKDVYKLSIPEN---TTVGSVVFKLDAVDDDIGQN
TaCelsr     EIQATDSGTTPKFA---RSNVTIIVTDVNDNPPQFTQKNYKVTIAED---AKINTLVTTVRATDPDNDDN
OcFmi       TAVATDEG--PQPR-SATVPVTIILNDVNDSPPKFTKLTYQENVDED---IAVSRTVVTVTANSDDLGQN
OlFmi       RAVATDRAGASGAL-SASVPVTITLSDVNDSPPRFDKSTYHEDVDED---VGVPRTIVTVEAKSDDLGDN
ScFmi       TIRATDHGTPALHS---DLLLIVDVLDINDHAPEFNQTTYNASVHED---IASGTAILQIFATDLDTGTN
AqCelsr3L   AVSARNTQPLSTPS-------YTFVTVVITNDSVFSESEILVPLHGN---ESVGQNLYTVEATRADS--Y
```

```
                     640       650       660       670       680       690       700
           ....|....|....|....|....|....|....|....|....|....|....|....|....|....|
MmCelsr1   AAIHYSIVSGNLKGQFYLHSLSGSLDVINPLDFEAIREYTLRIKAQDGGRPPLINSSGLVSVQVLDVNDN
DrCelsr1b  AKVHYSIINGNIKGQFYIHSPTGVIDVVSPLDYEMIREYTLRVKAQDGGRPPLINGTGMVVIQVVDVNDN
DrCelsr1a  ARVHYSIISGNVKGQFFIHTPTGAIDVINPLDYETIREYNLRIKAQDGGRPPLINGTGVVVVQVVDVNDN
MmCelsr2   ALVHYSIMSGNARGQFYLDAQTGALDVVSPLDYETTKEYTLRIRAQDGGRPPLSNVSGLVTVQVLDINDI
DrCelsr2   AVVHFSIMSGNTRGQFYIDAQTGKLDLVSQLDYEMNKEYTLRIRAQDGGRPPLSNISGLVTVQVLDINDN
MmCelsr3   GLVHYNIISGNSRGHFAIDSLTGEIQVMAPLDFEAEREYALRIRAQDAGRPPLSNNTGLASIQVVDINDH
DrCelsr3   ----------------------------------------------------------------------
DmStan     AAIRYAIIGGNTQSQFSIDSMSGDVSLVKPLDYESVRSYRLVIRAQDGGSPSRSN-TTQLLVNVIDANDN
ChFmi      GVIEFRPLDSNLR--FRINTSTGEIILKEAVDFDKYQRFSRFTVVAFDKGSPPKYANTEVEITIEDVNDN
TaCelsr    ADIRYSLAGQN---KFTINPTNGRITLIASLDYEQQQSYTLDVTAKDRGRPSLSN-TTTVVVSVTDVNDN
OcFmi      GRVVYSITAGNDDGTFGIGHVSGSIYVAKSLDYESHTLYTLTIKAQDGG-TVPLSATASVDIQVNNVNDN
OlFmi      ARIFYNIDSGNDDGTFGIGQTTGKIFVAKPLDYETTSFYSLTVKARDGG-TVPLSAFATVEIQINNVNDN
ScFmi      SKLAYRFASGNYDDALKIDPQTGMITVAKSLDYDTLQDGDAYQVQVQAVDGGGLSAMATVIVTVMNANDH
AqCelsr3L  PFQYTIIGGSNAFQFFTLNSMSGQLSLSSTVNYFGFLDQYRIVVEGRSLSDPLVFGNVVIIFSLSPKNSS

                     710       720       730       740       750       760       770
           ....|....|....|....|....|....|....|....|....|....|....|....|....|....|
MmCelsr1   -APIFVSSPFQAA----VLENVPLGHSVLHIQAVDADAGENARLQYRLVDTASTIVGGSSVDSENPASAP
DrCelsr1b  -APMFVSTPFQAS----VLENVPIGYSVIHIQAIDADSGDNAHLEYKLTDTS-----------------P
DrCelsr1a  -APMFVSTPFQAT----VLENVPIGYSVIHIQAIDSDSGENARLDYSLTDTT-----------------P
MmCelsr2   RPPIFVSTPFQAT----VLESVPLGYLVLHVQAIDADAGDNARLEYSLAGVG-----------------H
DrCelsr2   -APIFVSTPFQAT----VLENVPVGYSVIHIQAVDADAGDNSRLEYRLTETT-----------------A
MmCelsr3   -APIFVSTPFQVS----VLENAPLGHSVIHIQAVDADHGENSRLEYSLTGVA-----------------S
DrCelsr3   ----------------------------------------------------------------------
DmStan     -APRFYTSQFQES----VLENVPVGYNIIRVQAYDSDEGANAEITYSISERD-----------------D
ChFmi      -APRFAKRIFERTSD--IMEDIKVGSFVYKLSARDRDSGPNGDITFKLEDGLP----------------Q
TaCelsr    -QPRFSSNVYQAS----VKEDIPVGSSVLPVHAFDADSDQNKQVRYSIQRGS-----------------E
OcFmi      -APHFEHDPYRKS----IREGDTPQKSFLTVLAVDDDKGTLGDVTYRISAPA----------------DI
OlFmi      -APRFKKPRYRET----VRENDTPKNGFATVSAVDDDKGVLGDVTYRIDASS----------------DV
ScFmi      -SPVFDQDGYTFF----VTETDTPLAPVGKVYATDLDRGLFGSVVYDLPNVR-----------------D
AqCelsr3L  -TGGFTKEEYLFTVSNETAIGEVFGYVYLSVCNISSDSMNSSPLAVNYSITN------------------

                     780       790       800       810       820       830       840
           ....|....|....|....|....|....|....|....|....|....|....|....|....|....|
MmCelsr1   DFPFQIHNSSGWITVCAELDREEVEHYSFGVEAVDHGSPAMSSSASVSITVLDVNDNDPMFTQPVYELRL
DrCelsr1b  GFPFVINNSTGWVTVSAELDRETTEFYSFGVEARDHGVPTMSSSASVSVTILDVNDNVPTFTQHLYNLKV
DrCelsr1a  GFPFSINNSTGWITVSDELDRESTEFYTFGVEARDNGVPVMSSSASVSITVLDVNDNIPTFTEKMYSLKI
MmCelsr2   DFPFTINNGTGWISVAAELDREEVDFYSFGVEARDHGTPALTASASVSVTILDVNDNNPTFTQPEYTVRL
DrCelsr2   NFPFTINNSTGWIVVAAELDRETVDFYNFGVEARDHGAPAMSSSASISMTILDVNDNNPEFTQKAYYMRL
MmCelsr3   DTPFVINSATGWVSVSGPLDRESVEHYFFGVEARDHGSPPLSASASVTVTVLDVNDNRPEFTMKEYHLRL
DrCelsr3   ----------------------------------------------------------------------
DmStan     NFPLAVDPRTGWVQTIKPLDREEQGRFAFQVVAKDGGVPPKSASSSVVITVQDVNDNDPAFNPKYYEANV
ChFmi      DFPFELDPKSGEITVSKGLDYETTKSYKFGVIAVDNGHPPKKDTTQVVFNIGNVNDNNPKFKQSSYYKEI
TaCelsr    EIPFNVDSVSGVISTARPLNYESKSQYSFFIIATDGGTPPLSSSAQVTVTVVDVNDNAPKFPKSIYNADV
OcFmi      QAKFSIGSTSGNIDIVTHLDFESKSSYQFTVIAEDGGQSPASGQTTIRVGVTDVNDEVPQFTKTLYTEKI
OlFmi      EKKFDIGRTSGDIDIIAHLDYEDKTSYQFNVIAEDGGNPPSTGQTTVTVNVIDVNDEVPQFTKTLYAEKI
ScFmi      HAYFQRTSSDGILRAAAPLDHELRPRHTMTVRALDKGSPPRTALTNVTIIVNDINDVVPAFQQRSYEASI
AqCelsr3L  SDDVQINPVTGGLSFVSLPRNGSITNLTVIAVTRSGSSFSASVTVELEPTRYVMEIEKRSYMYVVYIDDG
```

```
                 850       860       870       880       890       900       910
           ....|....|....|....|....|....|....|....|....|....|....|....|....|....|
MmCelsr1   NEDAAVGSSVLTLRARDRDA--NSVITYQLTGGNTRNRFALSSQSGGGLITLALPLDYKQERQYVLAVTA
DrCelsr1b  NEDAVVGTSVLTVSAVDRDV--NSVVTYQISSGNTRNRFAISSQSGGGLITLALPLDYKQERQYLLTITA
DrCelsr1a  NEDAVVGTSVLTVTALDRDV--NSVVTYQISSGNTRNRFAITSQSGGGLITLALPLDYKQERQYVLTITA
MmCelsr2   NEDAAVGTSVVTVSAVDRHA--HSVITYQITSGNTRNRFSITSQSGGGLVSLALPLDYKLERQYVLAVTA
DrCelsr2   NEDAAVGTSVVTVSAVDQDI--NSVVTYQISSGNTRNRFSITSQSGGGLITLALPLDYKLERQYVLTVTA
MmCelsr3   NEDAAVGTSVVSVTAVDRDA--NSAISYQITGGNTRNRFAISTQGGVGLVTLALPLDYKQERYFKLVLTA
DrCelsr3   ----------------------------------------------------------------------
DmStan     GEDQPPGTPVTTVTATDPDE--DSRLHYEITTGNTRGRFAITSQNGRGLITIAQSLDYKQEKRFLLTVAA
ChFmi      LEDTRRGEAIEKVEAFDPDQLSNEDFEYAIEEGNSDDCFGIDP--FQGVLFLKCDLDYKRKNMYNLKLRV
TaCelsr    REDEKVGRQIVQVTATDVDS---KTIYYSIVSGNVRSRFGIDPQ---GYIYLAAPIDYLREKEYTLVVQA
OcFmi      SEAASKRTPVLQVSANDKDLPSNGQLLYKIGATTVGNPFRINS--LDGIIEVAALDRERKDVYVFVVEA
OlFmi      SELAGRGTPVVKVSANDADLPLNGYLGYQIGPGPIGNPFEIDS--GSGIVSVAAALDREKVDVYVFVVEA
ScFmi      SEDTARNRLVTTVEAVDVDG---LPLRYVIASGNIGSVFSINSADGSIRVAKSLDADRPGGDQYNLVVTA
AqCelsr3L  PTD-GRSTVLTRIDGGGCNN-------YTILAGDCYGLFSIDPVSGNLTIHPSP----LSPSAYSLVVGV

                 920       930       940       950       960       970       980
           ....|....|....|....|....|....|....|....|....|....|....|....|....|....|
MmCelsr1   SDG-TRSHTAQVFINVTDANTHRPVFQSSHYTVSVSEDRPVGTSIATISATDEDTGENARITYVLEDP--
DrCelsr1b  SDG-TRHDTTQVFINVTDANTHRPVFQSANYQVLVSEDRPVGSTVVVISATDEDTGENARITYVMEDN--
DrCelsr1a  SDG-TRFDTAQVFINVTDANTHRPVFQNANYHQTFSEDQPIGSTVVVISATDEDTGENARITYIMEDN--
MmCelsr2   SDG-TRQDTAQIVVNVTDANTHRPVFQSSHYTVNGNEDRPAGTTVVLISATDEDTGENARITYFMEDS--
DrCelsr2   SDG-TRFDTAKVYVNVTDANTHRPVFQSSHYTVNINEDRPVGTTVVMISATDEDTGENARITYIMDDS--
MmCelsr3   SDR-ALHDHCYVHINITDANTHRPVFQSAHYSVSMNEDRPVGSTVVVISASDDDVGENARITYLLEDN--
DrCelsr3   ----------------------------------------------------------------------
DmStan     TDSGGRSDTATVHINITDANNFAPIFENAPYSASVFEDAPVGTTVLVVSATDSDVGVNAQITYSLNEESI
ChFmi      TDADKKPGYAYFKIEVTDANNNSPRFGQDVYQWNVLENADIGTVIKSVLATDADTGENARITYSLQPGS-
TaCelsr    SDL-LLNSTATVKMRVIDTNNHAPTFDR-AYTADIYEDTAIGTVILTVTAKDADANENARLTYRIVTP--
OcFmi      TDG-ELTNTTQVKVTIEDENDSSPTFKGMPYIASIEENRPFNTTVYTVSAEDADIGDNGAITFSIQQT--
OlFmi      TDG-ELTNTTQVRVTITDENDSSPVFLDTPYTTSLDENVDAGTLVYTVSADDADIGRNGNVTYSLQQT--
ScFmi      HDG-QHASNVNVTVAVQDINDNSPSFQPTYYEAKVREGDRVGTPVLTVTARDLDSADNGRVTYSLQST--
AqCelsr3L  YCG-KFLSYISVEVRVLDVNN-PPVFDQRIYKVTARSSTPRNTRLLTLTYSDLDDPGSSNGTTHLSSTS-

                 990      1000      1010      1020      1030      1040      1050
           ....|....|....|....|....|....|....|....|....|....|....|....|....|....|
MmCelsr1   -----VPQFRIDPDTGTIYTMTELDYED----QAAYTLAITAQDNGIPQKSD-TTSLEILILDANDNAPR
DrCelsr1b  -----VPQFRIDPDTGAITTQIEIDYED----QASYTLAIIARDNGIPQKSD-TTYVEIIVLDANDNVPQ
DrCelsr1a  -----VPQFKIDPDSGAITTQMEIDYED----QASYTLAIIARDNGIPQKSD-TTYVEIIILDANDNSPR
MmCelsr2   -----IPQFRIDGDTGAVTTQAELDYED----QVSYTLAITARDNGIPQKSD-TTYLEILVNDVNDNAPQ
DrCelsr2   -----IPQFNIDADSGAVTTQMELDYED----QVSYTLAITARDNGIPQKSD-TTYLEILVNDVNDNSPV
MmCelsr3   -----LPQFRIDADSGAITLQAPLDYED----QVTYTLAITARDNGIPQKAD-TTYVEVMVNDVNDNAPQ
DrCelsr3   ----------------------------------------------------------------------
DmStan     NGLGSPDPFSINPQTGAIVTNAPLDRET----TSGYLLTVTAKDGGNPSLSD-TTDVEIGVTDVNDNAPA
ChFmi      ----PIDTFGIVNDTGAITTKKKLDRET----TKSYTLVVKASDHGKPFKKFGFATVVITVTDVNDNKPA
TaCelsr    -----VREFKIDSKSGAISIAAALDRET----RSTFSLEVRASDNGVPSLSG-RTDVVISILDSNDNAPV
OcFmi      -----SNLFSIDPKSGVVRTAAIIDFET----TEFVLLKVLATDHGTSPKQS-SAELSVTVRDENDNKPE
OlFmi      -----INLFRIDPKSGVIRTMTVIDYET----TTYTLVRVLATDDGLSPRQT-STELSVTVLNENDNKPK
ScFmi      -----DTTLQVDPDTGQVYIAAPADCDRG--KGIIQYYTLWAHDNGVSSRND-SARLQLTITDINDNAPV
AqCelsr3L  -----LPSFLRLLPNGELLLINALPSIAKPWETSSYNFSVVVSDGGSPSLSSTAEVVVIVINDIMPQSIS
```

```
                   1060      1070      1080      1090      1100      1110      1120
             ....|....|....|....|....|....|....|....|....|....|....|....|....|....|
MmCelsr1     FLRD--FYQGSVFEDAPPSTSVLQVSATDRD-SGPNGRLLYTFQGGDDGDGDFYIEPTSGVIRTQRRLDR
DrCelsr1b    FLRD--IYQGTVFEDAPVYTSVLQVSASDRD-SGSNGRLSYTFQGGDDGEGDFFIEPYSGIIRTARKLDR
DrCelsr1a    FGRD--KYQGTVFEDAPIYTSVLQISASDRD-SGSNGRVSYTFQGGDDGEGDFMIEQYSGIIRTHRKLDR
MmCelsr2     FLRD--SYQGTVYEDVPPFTSVLQILATDRD-SGLNGRVFYTFQGGDDGDGDFIVESTSGIVRTLRRLDR
DrCelsr2     FQRD--RYLGSVMEDIPVFTSVLQVSATDRD-SGLNGRVFYTFQGGEDGDGDFIIESTSGIVRTLRRLDR
MmCelsr3     FVAS--HYTGLVSEDAPPFTSVLQISATDRD-AHANGRVQYTFQNGEDGDGDFTIEPTSGIVRTVRRLDR
DrCelsr3     ----------------------------------------------------------------------
DmStan       FKSP--LYQASILEDALVGTSVIQVAASDPD-VGLNGRIKYLLSDRDIEDGSFVIDPTSGTIRTNKGLDR
ChFmi        FTKN--LYTFTIEEDAKIGAVVGHLQATDND-EGRNKEVRYQFKERK--HEYFSINAESGSIRTIKLLDR
TaCelsr      FSQP--SYNASIAEDVEIGTRVLQVAATDKD-EGVNQQIHFELAENENGNGTFAIDGSTGVIRTAKALDR
OcFmi        FMASPLYTSGSVSEEAIFGTLVLFVSATDKDELNS-NQLKYSFVEN---VTDFSISELSGAIRVAASLDA
OlFmi        FDRSPLHTIGRVKETDRIGTLALTVSASDADESGSGDQLKYSFVQN---VTDFSISELSGKIRIAASLDR
ScFmi        FVGLP--YNAQVFENLVRRTRVFTVLTSDDDYSINNRGVRFSLNQS--AANSFRIEHLTGVVRTTRTLDR
AqCelsr3L    FSRS--FYSGNITENSPPGTPILSLSLKNPQ-VPRHLEITYKLIGSPDVLAYLTISP-LGLLVSSLPIDR

                   1130      1140      1150      1160      1170      1180      1190
             ....|....|....|....|....|....|....|....|....|....|....|....|....|....|
MmCelsr1     ENVAVYNLWALAVDRGSPNPLSASVGIQVSVLDINDNPPVFEKDE-----------LELFVEENSPVGSV
DrCelsr1b    ENVALYTLKAFAVDKGVP-PLKAAVDIQVSVLDINDNAPVFEKDE-----------LYIYVEENSAVGST
DrCelsr1a    ENVPVYNLRAYAVDRGVP-PLKAAVEIQVSVLDINDNAPVFEKDE-----------LYIDVKENSPVDSV
MmCelsr2     ENVAQYVLRAYAVDKGMP-PARTPMEVTVTVLDGNDNPPVFEQDE-----------FDVFVEENSPIGLA
DrCelsr2     ENTAIYNLQAFAVDKGVP-ALKTAVDMQVTILDVNDNPPVFEKDE-----------FDIFVEENSPIGLV
MmCelsr3     EAVPVYELTAYAVDRGVP-PLRTPVSIQVTVQDVNDNAPVFPAEE-----------FEVRVKENSIVGSV
DrCelsr3     ----------------------------------------------------------------------
DmStan       ESVAVFHLTAIAVDKGSP-PLSSTVEVQIRLEDVNDSPPTFASDK-----------ITLYVPENSPVGSV
ChFmi        ETIERFTFEVLAIDQGTP-ALQTSTSVIIDIEDVQDSNPVFTEKV-----------YNFTIPENS--LVE
TaCelsr      ETVPEYTIVVTAIDKGRP-PKHSFATVKITITDVKDSPPEFYPKE-----------YDAYLPENSPAGTT
OcFmi        ETEPVYHFQVQAVDSGIP-PQTGVVNVTVTIVDVVDNPPQFSKLL-----------YEASVFENSDLRSF
OlFmi        ETVSVYHFKVQAVDSGIP-PQTGVVNATIHVIDVGDNPPEFEKSV-----------YDASVNEGSDVGTS
ScFmi        ETRDRYRILVYARDTGTP-ALTSELYVDIEVLDVPDNSPRFTAIASARPPANSSACYQTYIVENMPAGTH
AqCelsr3L    E---RYPSLTARVVAEYNKSIHTEATVSITVLDQPDTTPVFLQEY-----------YTVSVQAPLPVSKG

                   1200      1210      1220      1230      1240      1250      1260
             ....|....|....|....|....|....|....|....|....|....|....|....|....|....|
MmCelsr1     VARIRANDPDEGP-NAQIMYQIVEGNVPEVFQLDLLSG----DLRALVELDFE-VRRDYMLVVQATSAPL
DrCelsr1b    LARVSATDPDEGT-NAQILYQIVEGNFPEVFQLDIFSG----DLIALTDLDYE-TKMEYVIVVQATSAPL
DrCelsr1a    VARITAMDPDEGT-NAQILYQIVEGNIPEVFDLDIFTG----DLKALVDLDYE-TQKEYVIVVQATSAPL
MmCelsr2     VARVTATDPDEGT-NAQIMYQIVEGNIPEVFQLDIFSG----ELTALVDLDYE-DRPEYVLVIQATSAPL
DrCelsr2     VAHISASDPDEGS-NAQIMYQIVEGNIPEVFQLDIFSG----ELTALTDLDYE-TRAEYVIVVQATSAPL
MmCelsr3     VAQITAVDPDDGP-NAHIMYQIVEGNIPELFQMDIFSG----ELTALIDLDYE-ARQEYVIVVQATSAPL
DrCelsr3     ----------------------------------------------------------------------
DmStan       VGEIHAHDPDEGV-NAVVHYSIIGGDDSNAFSLVTRPGSERAQLLTMTELDYESTRKRFELVVRAASPPL
ChFmi        VGRIRATLADDAFRHSMVYSFT---KYQTTFRIASLQG----IIRANSPLDYEKQHHYQLHVRVVSDSGR
TaCelsr      VVQLNATSKDEGT-NAFITYEATSGFEPRIFHVNSSNG----VVTTLRPLDFE-IRRTYQIRVRAQSPPF
OcFmi        VTQVKATSRDSSVPDSDIRFSIRGGNDPKTFEIDPQSG----VITLIAEVDAE-ILGDFFLLVRAELQHQ
OlFmi        IVRVKATSRDSSVPDSAIRYSITSGNVPPTFAIDERTG----DIALAVEVDAE-TTPSFFLQVRAQVESR
ScFmi        IMTVHATVPDISVEQSSIRYRLMSVSPDELFHLNSSTG----ELTSGAVLDRDKGQLFYTARVDAYTDFV
AqCelsr3L    LLVLNAITQDSSP----ITYSLVG--SSDIFTLNSATG----SLDTLTELSAP---NSYTLLAKATSSSG
```

```
                   1270      1280      1290      1300      1310      1320      1330
            ....|....|....|....|....|....|....|....|....|....|....|....|....|....|
MmCelsr1    VSRATVHIRLLDQNDNPPELPDFQILF----NNYVTNKSNSFPSGVIGRIPAHDPDLSDSLNYTFLQGNE
DrCelsr1b   VSRATVHVLLVDVNDNDPVLQDFEIIF----NNYITNKSSSFPNGVIGKVPARDPDVSDKLIYTFVEGNE
DrCelsr1a   VSRATVHVRLIDVNDNDPVLQNFEIIF----NNYVTNKSNSFPSGIIGKVPAHDPDVSDKLKYSFIEGNE
MmCelsr2    VSRATVHVRLLDRNDNPPVLGNFEILF----NNYVTNRSSSFPGGAIGRVPAHDPDISDSLTYSFERGNE
DrCelsr2    VSRATVHIKLIDKNDNVPVLKNFQIIF----NNYVTDKTNSFPSGVIGRIPAQDPDVSDQLHYSFEAGNE
MmCelsr3    VSRATVHVRLVDQNDNSPVLNNFQILF----NNYVSNRSDTFPSGIIGRIPAYDPDVSDHLFYSFERGNE
DrCelsr3    ----------------------------------------------------------------------
DmStan      RNDAHIEILVTDVNDNAPVLRDFQVIF----NNFR----DHFPSGEIGRIPAFDADVSDKLHYRILSGNN
ChFmi       EDTAVVNVHVEDVNDHPPILEDFYIFLN----------ALNGKYDPMFRVPAEDPDVSSVLRYGIITGNE
TaCelsr     FTETTVTIHIIDVNDNPPTIQNYTFLLNYFP--------DYYPTASVGKLNATDPDISDTLTFAIIQGNN
OcFmi       FSDVRVNVQVLDENDNPPQVLPLTMHL----IVIEG----FFRRTTIGKVRAIDPDPTAILIQDLVGTLP
OlFmi       FGDVLVNVRVLDLNDNPPEILPLTIHL----IIIEG----FFRGTSIGRVRAVDRDSTAVLRQSLVGEMP
ScFmi       SGSCEIIIRVRDVNDNPPQVRDTDVVAN--------LLRSSFQGGVIGQVRIIDPDISLVVQTPLSSPGF
AqCelsr3L   LYNTARVHVRVFVGPSRPQLRPSSLFLS----TFSYLLPSVSLLGDLG--TFVNGQRRNGFGLSLNPSSC

                   1340      1350      1360      1370      1380      1390      1400
            ....|....|....|....|....|....|....|....|....|....|....|....|....|....|
MmCelsr1    LSLLLLDPATGELQLSRDLDNNRPLEALMEVSVSDGIHSVTALCTLRVTIITDDMLTNSITVRLENMS-Q
DrCelsr1b   LGLLILNQDTGELKLSKDLDNNRPLEALMRVTVSDGLHQVSALCTLRVTIITDDMLTNSITVRLENMS-Q
DrCelsr1a   LSLLILNPDTGELKLSKDLDNNRPLEATMKVSVTDGIHQVTAFCTLRVTIITDEMLTNSITVRLENMS-Q
MmCelsr2    LSLVLLNASTGELRLSRALDNNRPLEAIMSVLVSDGVHSVTAQCSLRVTIITDEMLTHSITLRLEDMS-P
DrCelsr2    LKLVILNQSTGEIRLSRALDNNRPLEASMKISVSDGVHSVSAQCLLQVTVITDEMLSNSITLRLANAS-Q
MmCelsr3    LQLLVVNRTSGELRLSRKLDNNRPLVASMLVTVTDGLHSVTAQCVLRVVIITEELLANSLTVRLENMW-Q
DrCelsr3    ------------------------------------------------------MLSSSITVRLQNMS-Q
DmStan      ANLLRLNSSSGGLVLSPQLNTNVPKFATMEVSVSDGINEAKAIMQLSVRLITEDMLFNSVTVRLNEMT-E
ChFmi       HDFISLNDETGELFLKKSIINT-AAEIEILFEVSDGKYADRAYGHILVSEVTDVMVKNSMFIVLNNATRN
TaCelsr     DRLLQLNRTSGEIYLSPSAGNVQE-SRQMTISVSDGIHVARSTLSLKVIAVTSDTLKNIAVLRFTNIL-P
OcFmi       SDFIEFDNATGSVIALPGISQG---EYVINTTVSDGQAEAWGLTHIDVTMVSNATFDNAIVLRIDSFT-R
OlFmi       STFLEFENASGTIFASPGLSSG---SYVINTTATDGVTTASGLTRVDVTIVSNATFVNAVTLRIVDFT-R
ScFmi       DSGFFTLARDGSIAATRSVLPGVYKLRSSADDQNAAHSRAEGTTTVHVRWISDTSLQHSVSMQVSGSRDL
AqCelsr3L   SADKYFRITEGSLYVLNTITSG---SHQLNISVTDGRVIWSETITVYANLLTNDSLDHTLSLALPNLS-L

                   1410      1420      1430      1440      1450      1460      1470
            ....|....|....|....|....|....|....|....|....|....|....|....|....|....|
MmCelsr1    EKFLSPLLSLFVEGVATVLSTT------KDDIFVFNIQNDTDVSSN-ILNVTFSALLPG----------G
DrCelsr1b   ERFLSPLLSLFAEGVAAVLSTS------PDGIFIFNVQNDTDVSGS-ILNVTFSALLPG----------G
DrCelsr1a   ERFLSPLLSLFVKGVAAVLSTS------REGVFIFNVQNDTDVSGN-ILNVTFSALLPG----------G
MmCelsr2    ERFLSPLLGLFIQAVAATLATP------PDHVVVFNVQRDTDAPGGHILNVSLSVGQP--------PGPG
DrCelsr2    ERFLSLLLAQFLEGVASVLSAS------REDVVVFNIQDDTDVSAG-ILNVSLSVALPGQGSRGRGVSQG
MmCelsr3    ERFLSPLLGHFLEGVAAVLATP------TEDVFIFNIQNDTDVGG-TVLNVSFSALAPR------GAGAG
DrCelsr3    ELFLSPLLTHFLDGVSAVLSVS------PDDVFVFNVQPDADAG--KVLNVSFSAALP------------
DmStan      EAFLSPLLNFFLDGLAAIIPCP------KEHIFVFSIQDDTDVSSR-ILNVSFSARRPD----------V
ChFmi       DFLKATVLRKFKDSLAQIFDVDGSKIVDASKIFILSIEAFRDPRKGNKNAEMLEIAIAVKS--------K
TaCelsr     ETFITNMTSLLTNTIAGIMRCQ------SRDIYILNVQQDDVAGQN-ILNVTISGWDRTN----------
OcFmi       ERIVKDHLDKLRASLAKWIPCR------QEDIQIFSVESFG---SLVDVLFAVKKADGS-----------
OlFmi       RRIVKDHLNKLRLSLAKWIPAR------SENIQIFSVEIVEDSSSLVDVLFAVKNDDG------------
ScFmi       ADFVQHYLLRTSAAIERLLTTM------PGSVEIFTVRETTGALGDGAVDLIFAVRSRS-----------
AqCelsr3L   DHFLGRFLSPFRSALSHMLSCM------HECLHIISVDELPGARGVKVVIAAKEKDLVT-----------
```

```
                   1480      1490      1500      1510      1520      1530      1540
           ....|....|....|....|....|....|....|....|....|....|....|....|....|....|
MmCelsr1   TRGRFFPSEDLQEQIYLNRTLLT--TISAQRVLPFDDNICLREPCENYMKCVSVLR-FDSSAPFISSTTV
DrCelsr1b  APGRYFPSEELQEQLYLNRTLLM--LISTQRVLPFDDNICLREPCENYMKCVAVLK-FDSTAPFVASNTV
DrCelsr1a  VPDRYFPSEELQEQIYLNRTLLQ--EISSQNVLPFDDNICLREPCENYMKCVSVLK-FDSSPPFIASDTV
MmCelsr2   GGPPFLPSEDLQERLYLNRSLLT--AISAKRVLPFDRQHLLREPCENYMRCVSVLR-FDSSAPFIASSSV
DrCelsr2   SEVEFFGSEELQERLYLNRSLLA--QISSQEVLPFDDNICLREPCENYMKCVSVLK-FDSLAPFVASDTI
MmCelsr3   AAGPWFSSEELQEQLYVRRAALA--ARSLLDVLPFDDNVCLREPCENYMKCVSVLR-FDSSAPFLASTST
DrCelsr3   -GGQFFPSEALEEQLYLNRPRLN--ALAHMEVLPFDDNVCLREPCQNYMKCISVLR-FNSSAPFIASPST
DmStan     SHEEFYTPQYLQERVYLNRAILA--RLATVEVLPFDDNLCVREPCLNFEECLTVLK-FGNASEFIHSDTV
ChFmi      MADTYMTADFLKDKFYLNATAFTRSGFDLVSFDFWGEYFCGSESCKNLERCSIQWSNTNQISSTIKSKYV
TaCelsr    --NRYYLQNYFRDSIYIYREQLL--AKG-FNLLPFDGGYCLPQPCSPNYDCVVNFG-ASLDNAVVSTPKI
OcFmi      --GGFISRNDVVLQMDLNSSPIE--KESGLLFDEVNVDLCLREPCKNFQACTNQLA-VQTETNAIFKDDV
OlFmi      ---GFVSRGDVIGQIDLHSSAIE--KESGVSFEDVNLDECLREPCKNFQVCTNHLA-VQTATNPVVKDDV
ScFmi      --GAYMGPGIVIESIDVQQVMFY--QHSGLRINFINLDTCRTEPCNYFQDCLDSVR-FTGENTTIVAGNT
AqCelsr3L  ----YKSSLELRRTIEAEIGHIS---SALSWTVAVMLDSCSSSPCPNPLRQCSPYVSLALPHKTITSQSL

                   1550      1560      1570      1580      1590      1600      1610
           ....|....|....|....|....|....|....|....|....|....|....|....|....|....|
MmCelsr1   LFRPIHPITGLRCRCPPGFTG----DYCETEIDLCYSNPCGANGRCRSREGGYTCECFEDFTGEHCQVNV
DrCelsr1b  LFRPIHPVNGLRCRCPDGFTG----DYCETEVDLCYSGPCQNNGKCRSKEGGYTCECPQDFTGERCEVNA
DrCelsr1a  LFRPIHPINGLRCRCPAGFTG----DYCETEIDLCYSGPCRNNGRCRSREGGYTCECLEDFTGENCEVDS
MmCelsr2   LFRPIHLVGGLRCRCPPGLTG----DYCETEVDLCYSRTCGPHGRCRSREGGYTCLCRGCYTGEHCEAST
DrCelsr2   LFRPIHPIAGLRCRCPLGFTG----DYCETEIDLCYSRPCGAHGVCRSHEGGYTCHCMQDYTGERCEISS
MmCelsr3   LFRPIQPIAGLRCRCPPGFTG----DFCETELDLCYSNPCRNGGACARREGGYTCVCRPRFT--DCELDT
DrCelsr3   LFRPIHPITGLRCRCPAGFTG----DYCEIEINLCYSNPCMNGGVCARREGGYTCICREDYTGERCEFDR
DmStan     LFRPIYPVNTFACSCPEGFTGSKEHYLCDTEVDLCYSDPCQNGGTCVRREGGYTCVCPSTHTGQNCETGV
ChFmi      IFRGIYFKPTLKCECPHRFHG----MKCDKPFNMCFMERCNSNGKCISTDDGFSCKCDHGYTGDFCAFDR
TaCelsr    IVRNIRQAKRYACECPVGYTG----LRCNRMRNLCRSNPCGNNGKCVNIENGYTCICNSGYAGINCEIGS
OcFmi      LFLSLQRRISYTCTCPSGFVHSWNPNSCLEQINSCLSEPCLFGGTCVNILDGFECKCRHGTIGNRCEIPC
OlFmi      LFLSLQRRISHTCTCPSGYVHSWNPNSCSERIDSCLSQPCLFGGTCVNTLDGFECKCAAGIVGKTCDVPC
ScFmi      SFTSLLSKRSFSCTCPLGYQHTWHSNSCQREVDECASSPCKYGGSCIDMLSGYKCLCLANTTGPACETFC
AqCelsr3L  LVKSLSSASAHKCVCPRGYDS-----KCTSELNECEPSPCDYDAVCTDLINDYHCSCPPFTFGKNCSTPC

                   1620      1630      1640      1650      1660      1670      1680
           ....|....|....|....|....|....|....|....|....|....|....|....|....|....|
MmCelsr1   R--SGR----CASGVCKNGGTCVNLLIG-GFHCVCPPG-EYEHPYCEVSTRSFPPQSFVTFRGLRQR-FH
DrCelsr1b  R--SGR----CVPGVCKNGGRCLDLLVG-GFMCQCPDG-EYEKPYCQMSTRSFPGQSFITFRGLRQR-FH
DrCelsr1a  R--SGR----CVPGVCKNGGECVNLLVG-GFTCNCPSG-EYEKPFCEMTTRSFPGQSFITFRGLRQR-FH
MmCelsr2   H--SGR----CTPGVCKNGGTCVNLLVG-GIKCDCPSG-HFEKPFCQVTTRSFPARPFITFRGLHQR-FH
DrCelsr2   R--SGR----CAEGVCKNGGTCLNLLVG-GFRCECPSG-GFEKPFCLLSSRNFPPQTFITFKGLRQR-FH
MmCelsr3   E--AGR----CVPGVCRNGGTCTNAPNG-GFRCQCPAGGAFEGPRCEVAARSFPPSSFVMFRGLRQR-FH
DrCelsr3   R--GGR----CVAGVCRNGGTCRELSGG-GFRCECPAG-GYEKPYCSVTTRSFPPKSFMMFRGLRQR-FH
DmStan     G--HLRP---CPSETCEGGLSCLSNYPS-SQPPPYTAT-------CELRARAFGRNSFLTFESLKQR-HR
ChFmi      QTSSCPIQLNLAKNPCGLRGACSAKSGG-GFDCTCPAK-GTDTEYCKLSTRYFPKGSYIAMPGLKKQQRN
TaCelsr    C----------ASQPCKNGASCVENANGQGFYCTCPKY--YGGPLCDAIERSFPGNSYVTVGGIKQR-WR
OcFmi      PSLSCAL---CEPNPCLNGGVCSVDSNGMAFSCRCPSG--FDGPLCEQTIAHFDYGSLLAFSPPFGR-WK
OlFmi      PSLSCKL---CDPNPCRNGGRCVVDENGSAFSCTCPSG--YDGPLCEQATAHFDYGSLLAFSPRLGR-WG
ScFmi      PSASCDF---CEPNPCMNGGTCTTTSRG-EVQCVCKDG--FDGPQCQMTLASFLEGDYVAFPSLTSR-WQ
AqCelsr3L  RFSAPCS--ACSPNPCLNGGSCSIQSEGTISCTSCPAG--YTGPLCELTVARVSGAGGASLEPLGRQ-RE
```

```
                  1690      1700      1710      1720      1730      1740      1750
              ....|....|....|....|....|....|....|....|....|....|....|....|....|....|
MmCelsr1      FTVSLAFATQDRNALLLYNGRFNEKHDFIALEIVEEQLQLTFSAGETT------TTVTPQVPGGVSDGRW
DrCelsr1b     FTLSFMFATRERNALLLYNGRFNEKHDFIAIEIVKEQIQLTFSAGESK------TTVTPFVAGGVSDGQW
DrCelsr1a     FTVSFMFATRERNALLLYNGRFNEKHDFIAVEIIEEQIQLTFSAGESK------TTVAPFVPGGVSDGQW
MmCelsr2      FTLALSFATKERNGLLLYNGRFNEKHDFVALEVIQEQVQLTFSAGEST------TTVSPFVPGGVSDGQW
DrCelsr2      FTLSLSFATREPNGLLLYNGRFNEKHDFIAMEIVNQQIQLTFSAGGTK------TTVLPFIEGGVSDGHW
MmCelsr3      LTLSLSFATVQPSGLLFYNGRLNEKHDFLALELVAGQVRLTYSTGESN------TVVSPTVPGGLSDGQW
DrCelsr3      LSISLSFATLESNGLLFYNGRFNEKHDFLALEILDGQMVLKYSTGESS------TQVSPYLPGGVSDGNW
DmStan        FNLKLRFATVQENGLLLYNGRYNELHDFIALEIHEGHVSFSFSLGDHS------ERISVIQEAKVSDGKW
ChFmi         FDISFQFRTFQANAVLLYNGRYSNQNDFIAVEIVDGQVVFSVNFGKSKDNKEQVTTVQSFNAGGVNDGKW
TaCelsr       LEISVQFATIHDNGLLLYNGRYNHRGDYLALELINGKVRLSFSTGTDK------YSATANIEGNVNDGKW
OcFmi         LSFSLEFSTVDPNGLIMYNSRLGEQYDFVALELVGGQLYFLFSFGSST------AKAVAKSQTSLADGQW
OlFmi         FDIRLDFATVDPNGLMMHNNRLGEQYDFIALELDAGQITCSLSFGSLT------ETVVARSQVPLSDGEW
ScFmi         LKLSLSFVTARSNGLLLYNGRLGTQYDFLALELINGRLQCQISLGNGP------VKFTTLNTRVLSDAKW
AqCelsr3L     VEFSFDFIGVQPNAMLLHAGQLNNGEDYIAIGLVLGNLQVSVRWGNWS-----VRTTLSSKNGSLNDGQW

                  1760      1770      1780      1790      1800      1810      1820
              ....|....|....|....|....|....|....|....|....|....|....|....|....|....|
MmCelsr1      HSVLVQYYNKPNIGHLGLPHGPSGEKVAVVTVDDCDAAVAVHFGSYVGNYSCAAQGTQSGSKKSLDLTGP
DrCelsr1b     HTIHLHYYNKPNIGRLGVPHGPSQEKVAVVALDDCDVAMALRFGGQIGNYSCAARGTQTGQKKSLDLTGP
DrCelsr1a     HSVQLHYYNKPKISHLGLPQGPSGEKVAVVAVDDCDIAMAVRFGTQIGNYSCAAQGTQTGQKKSLDLTGP
MmCelsr2      HTVQLKYYNKPLLGQTGLPQGPSEQKVAVVSVDGCDTGVALRFGAMLGNYSCAAQGTQGGSKKSLDLTGP
DrCelsr2      HTAHVHYYNKPVLNRAGLPQGPSDQKVVVVTVDDCDSSVALRFGHVVGNYSCSAQGSQLGSKKSLDLTGP
MmCelsr3      HTVHLRYYNKPRTDALGGAQGPSKDKVAVLSVDDCNVAVALQFGAEIGNYSCAAAGVQTSSKKSLDLTGP
DrCelsr3      HTVHIHYYNKPKRSVSGEVQGPSDEKVAVLSVDDCDTAVSLRFGAQLGNYSCAAQGRQTSSKKSLDLTGP
DmStan        HQVEVVYLNRSVTLVLDNCDTAIALSGQLGDRWSCANRTTLKLDKRCS-------LLTETCHRFLDLTGP
ChFmi         HTVKVQLKNKVISIAVGESCDIETASVLNSQGTHR---------------YCAASTRVQGEMKTLDLTAP
TaCelsr       HTVSVKYLN----------------QVASVSIDYCDTDLAIRYGSTIGNYSCAATTLSQDQGKSLDVTAP
OcFmi         HTVTVNLANQVADLSVTNCQNPDTSNDALIYEDD---------------FICSGSKKPEGETKSLDLDGP
OlFmi         HSVTIRLVDKTLHLTVDNCQNPDSSNDARIYEDD---------------FICSASGTPKGNARSLDVDGP
ScFmi         HTVTLELKDLAVKMRVDGCYHEHKSASFLDDFICQEIRN--------ITEEVNIDRKRFRMGRSLDLGGP
AqCelsr3L     HSVEFRLNDMTLDIVLGNCIESEVILERD-------------------IVNCSVSIPVQTSSKIINLDSS

                  1830      1840      1850      1860      1870      1880      1890
              ....|....|....|....|....|....|....|....|....|....|....|....|....|....|
MmCelsr1      LLLGG-VPNLPEDFPVHSR--QFVGCMRNLSIDG----RIVDMAAFIANNG--TRAGCASQR-NFCDG--
DrCelsr1b     LLVGG-VPNLPEDFPVQNR--DFVGCIKNLTIDS----KPIDMANFISDNG--TAAGCAAKR-DFCSQ--
DrCelsr1a     LLLGG-VPNLPEDFPIRNR--DFVGCMRNLTIDS----KSVDMASYIANNG--TTEGCPAKK-NFCYE--
MmCelsr2      LLLGG-VPDLPESFPVRMR--HFVGCMKDLQVDS----RHIDMADFIANNG--TVPGCPTKK-IVCDS--
DrCelsr2      LLLGGGVPKLPEEFPVQNR--QFIGCMKDLRIDE----RHVDMAGFIANNG--TLPGCSAKR-NFCNN--
MmCelsr3      LLLGG-VPNLPENFPVSHK--DFIGCMRDLHIDG----RRMDMAAFVANNG--TMAGCQAKS-HFCAS--
DrCelsr3      LFLGG-VPNLPENFPFSTR--EFIGCMKDLHIDN----RPVDMAGFIANNG--TLPGCSAKL-PFCKS--
DmStan        LQVGG-LPRIPAHFPVTNR--DFVGCISDLRIDD----RFVDLNSYVADNG--TLAGCPQKA-PLCQS--
ChFmi         ALIGG-LPDMQRTFISPVK--DFIGCMRNIVFDH----NSLDLSNYLHNFG--SAANCPAPE-PSCNDAY
TaCelsr       LQIGG-VPTIRDTYPMAFKKNDFVGCIRNLYINS----KLIPLDNFYVNNG--SVIGCRQTD-QRCQGGS
OcFmi         LYFAG-VENIDFRYPTSSL--NFSGCMRNIEVNG----KLLDLASARKSIN--TLNSCPPKS-RFCDS--
OlFmi         LYFGG-IENTFFDHPTNNV--NFSGCIRNVEIDG----HLLDFSTATKNVK--TVNGCPPKL-SYCDS--
ScFmi         LYIGG----LRSAFNSELSNQHFVGCIRDIVVNE----QYLDFSSSIDQSAGVTLSACPPLSSQFCDG--
AqCelsr3L     LYLFNSLGESTNQIPSLAQ---FTGCLSNVKIGGERNGKLVDFSSVPTHPGASLLPGCPQSS-SSCQS--
```

```
                1900      1910      1920      1930      1940      1950      1960
           ....|....|....|....|....|....|....|....|....|....|....|....|....|....|
MmCelsr1   -TSCQNGGTCVNRWNTYLCECPLRFGGKNCEQAMPHPQRFTGESVVLWSDL--DITISVPWYLGLMFRTR
DrCelsr1b  -AVCQNGGVCVNRWNTHTCNCPLGYGGKNCEHVMPAPLHFDGHALVSWSDT--DITIAIPWYMGLMFRTR
DrCelsr1a  -GLCQHGAQCENKWNTHFCECPEGRGGKNCDQDMPSRQHFDGHAMMLWNDP--DMTIAVPWYIALMFRTR
MmCelsr2   -SICHNGGTCVNQWNTFSCECPLGFGGKSCAQEMANPQRFLGSSLVAWHGL--YLPISQPWHLNLMFRTR
DrCelsr2   -NPCLNGGSCVSLWGSFRCDCALGFGGRNCEKVMSNPLRLQGKGLLWWADL--ASVMTPPWHMEFMFRTR
MmCelsr3   -GPCKNNGFCSERWGGFSCDCPVGFGGKDCRLTMAHPYHFQGNGTLSWDFGN-DMAVSVPWYLGLSFRTR
DrCelsr3   -NPCQNGGTCRVSWETFSCDCPVGFGGKDCSLVMSHPHRFLGSSALWWDLKN-EVTITTPWYMGLVFRTR
DmStan     -EPCFNGGTCREGWGTYSCECPEGYAGNSCQDNIPAPWRFSGDGSLSFNPL--LRPIQLPWTTSFSLRTR
ChFmi      CTKYVNG-DCHETFDGPKCSCRSRFVGQRCEKAA-EPIRLNANSVLVVDDSQASGSGFSAWRYSMVVKTT
TaCelsr    ISPCSSGAECVDVYDGFRCRCPANIGGKTCQDTIVRTWRFTDQSIIIYPLPPLQNTITAPWSLSLIFRTR
OcFmi      -NPCLEGGACADVWNGFYCSCPTGNDGPQCSVVS--AFQFDGQAYTHHHLS----KPTFLESFSVTFRTS
OlFmi      -DPCKNGGTCSSVWKGYYCSCTPGHGGQQCDVSS--AFQFDGQSYRHYHFS----KAVFLRTFSVSFRTR
ScFmi      -AVCGNNGVCQNVWNTSYCGCMPDFGGKQCEHAKVYKAKFTGQSFIQYYLS----QQFFRGRRGLTFRTR
AqCelsr3L  --SCGGDGKCIEFWNGSRCHSDSNDATEAISFDGSSSSSYVVSSISAGTSMS-YSSPFNVMNISFDMRTL

                1970      1980      1990      2000      2010      2020      2030
           ....|....|....|....|....|....|....|....|....|....|....|....|....|....|
MmCelsr1   KE--DGVLMEATAGTSSRLHLQILNSYIRFEVSYGPSDVASMQLSKSRITDGGWHHLLIELRSAKEGKDI
DrCelsr1b  KS--TGVILQATAGEFSKINLMVTNRHLRFQVFLGNRRVALLDFPQVYVDNGEWHHVLVELKSGKDGKDI
DrCelsr1a  QTSQTATLMQVNAGDTSQINLLIRDKYVQFEVLLGEQKVAVLDFTDVRVNDGEWHHLLVELRSSKDGKDT
MmCelsr2   QA--DGVLLQAVTRGRSTITLQLRAGHVRLSMEGTGLQASSLHLEPGRANDGDWHHAQLALG-ASRGP--
DrCelsr2   QP--TATVMHMSSGQQHNFTIQLREGFVVTSVQ-RGEDSVVSRVDDVTVSDGQWHHVSLELKGPATGG--
MmCelsr3   AT--KGILMQVQLGPHSVLLCKLDRGLLSVTLNRASGHTVHLLLDQMTVSDGRWHDLRLELQEEPGGRRG
DrCelsr3   SK--EGVLLQAQAGQYTNLIFQVVGGQLVFSVARGSTRPVRLRLDQVQVCDGRWHDLQLELRDVRSGRET
DmStan     QK--EAFLLQIQIGQNSSAAVCLRQGVLYYIFDGEPMYLAGAFLS-----DGEWHRVEIRWQQGSEIH--
ChFmi      QKD---AVILEETINGVDGRLMLKNGYLQYHHKS----KLILVLDSFFMANGKWNQIQIKWTANR-----
TaCelsr    LRN---AYLSYMTDSQRDIVLMIENGLLKYKFG-----NYETVVKYIRVNDGEWHEARIEWEKSG-----
OcFmi      NLD-----ATLFSTATTLLQLRNGHVQLIFNVTSLS--EAIFMTNVIAVNDGLWHDITVTFKEGTELS--
OlFmi      QAD-----ATLLSTGRTQLQIRNGHIQLLFNVTSSSQTEGIFMNDVVAVNDGRWHSIAVTIQSGTELS--
ScFmi      QAN-------STLWYTDHSTLDVLHGQLAYSFLAGS-KSYHMRLGDIQVDDGLWHQAVIDLGDGRSTRLS
AqCelsr3L  QDG----YTHVLAIGDKGDLELFYGSLIFNYRPSSPMLRPLSLTADARVNDGYWHGIKLSFS--------

                2040      2050      2060      2070      2080      2090      2100
           ....|....|....|....|....|....|....|....|....|....|....|....|....|....|
MmCelsr1   KYLAVMTLDYGMDQSTVQIGNQLPGLKMRTIVIGGVT--EDK-VSVRHG-FRGCMQGVRMGETSTNIATL
DrCelsr1b  KYMALVSLDYGMFQRTVEIGNELPGLKLRNLFIGGLL--KKD-DTVQGG-FNGCMQGVRMGETSTNIANI
DrCelsr1a  KYMAQVLLDYDMFKKSVEIGNELPGLKLKSFFIGGLQ--GQR-DIVQQG-FKGCMQGLRMGETATSTANI
MmCelsr2   -GHAILSFNYGQQTAEGNLGPRLHGLHLSNITVGGVP--GPA-SGVARG-FRGCLQGVRVSETPEGVHSL
DrCelsr2   -VTATLSLDYGLYTSSMDLDSKLKGVRLKTLSVGGVS--DGR-NRVIGG-FRGCIQGLRIGGDSSTPAFP
MmCelsr3   HHIFMVSLDFTLFQDTMAMGGELQGLKVKQLHVGGLP--PSSKEEGHQG-LVGCIQGVWIGFTPFGSSAL
DrCelsr3   RYIATVRLDFGLYQGTVIVGNEINGVKVKHLHVGGVL--GSG--EVQNG-IRGCIQGVRLGVRPD-SPAL
DmStan     -----FSVDYGQRSGSVPMSQKVQGLYVGKIVMGSPD--GSIGAVPEASPFEGCIQDVRIG----AGQSV
ChFmi      -----LYLSSLYDQHSVSTPLPLTTLSLKMTSFGGTG---------------NNKLLACIKAFTYNDNDI
TaCelsr    ---VFLKLDYNSYQASFQLDLSRP-VQINQIMFGGNR--VIN-SSTSIATIQNGFDGCMQGIAINDRQIA
OcFmi      LDYGRFTATTSYVETATVTNVYVGGRHIGSKNVIDEF--SGCLDAVQIN-GRALPFVNGISALTSRGGNL
OlFmi      LDYGRFRVIVSDVETAPVTNVYVGGKHVGPKNVVDRF--SGCVDRTEIN-GEVVPSASGVVARKFAGVDV
ScFmi      LDYGKYSAMLPVTESQTTFNFVLGAAISSAAATGVKSNFKGCMHSAFLGSDMLSLDPSGATTDSAVTASA
AqCelsr3L  ---SSLTRLSIDDERIVADSNQTITIATSRVELGGRE-------GSHSRSYDGCIRNLRINSETVDDDNY
```

```
                      2110      2120      2130      2140      2150      2160      2170
                ....|....|....|....|....|....|....|....|....|....|....|....|....|....|
MmCelsr1        NMNDALKVRVKDGCDVEDPCAS-----SPCPPHSHCRDTWDSYSCICDRG----YFGKKCVDACLLNP-C
DrCelsr1b       NIRHAKRIHAKDGCNVPDACLA-----NLCPSHSHCTDNWTSHTCVCQPG----YFGRDCVDACLLNP-C
DrCelsr1a       NMHHAQKIRVEDGCDMSENCDA-----INCPENSQCTD---EHTCTCDPG----FFGRDCVDACHLNP-C
MmCelsr2        DPSRGESINVEPGCSLPDPCDS-----NPCPTNSYYSNDWNSYSCSCVLG----YYGDNCTNVCDLNP-C
DrCelsr2        SMPQARVLNVESGCSLPNTCGS-----NPCPAHSDCKDDWDAHSCRCHTG----YYGNSCTDACALNP-C
MmCelsr3        L-PPSHRVNVEPGCTVTNPCAS-----GPCPPHADCKDLWQTFSCTCRPG----YYGPGCVDACLLNP-C
DrCelsr3        P-RPSRTIKVETGCNVGNPCNS-----SPCPTHSQCTDEWDRHTCVCEPG----FYGKSCMDACQLNP-C
DmStan          LSRPTIRENVEDGCESRAQCP------DHCPNHSSCQSSWDLSTCECDSG----YVGTDCAPICTVRP-C
ChFmi           NLLHTSNKHVQKGCLESSLCDT-----LDCGTGTCQITSGVAQCHCNGAG----YKGPQCVDICKDSP-C
TaCelsr         VTSFTVQQNVGANCNIGNPCAS-----NPCPSQSSCVAEWNRYTCKCSTG----YVGPKCISACSLNP-C
OcFmi           TVKTTSSPSIKTGCDSQVMCAS-----LQCPAHSRCMEP---ASCECSLG----RKGRQCVDVCLVNP-C
OlFmi           GVTTTVVG-VTPGCPN--LCDS-----TRCPANSVCVEP---ASCRCTSG----HKGRQCVDVCASQP-C
ScFmi           LQISSSPQNIGRGCLSAAVCAA-----YPCSRHATCIDEWEQYRCECPPF----FSGRKCLDVCYQDR-C
AqCelsr3L       N---VTLIGTVTGCSNKSPCDSSSSSYTGCPANSYCIEGWRASSCMCDSSRSYFHNGGTCVQPCNQPVNC

                      2180      2190      2200      2210      2220      2230      2240
                ....|....|....|....|....|....|....|....|....|....|....|....|....|....|
MmCelsr1        KHVAACVRSPNTPRGYSCECGPG---------HYGQYCENKVDLPCPKGWWGNP-VCGPCHCAVSQGFDP
DrCelsr1b       EHTSSCVRKPSTKRGYSCDCGHN---------YYGQYCEHKGDQPCAHGWWGNP-TCGPCNCDLSKGFKR
DrCelsr1a       EHLSTCVRKPSSSHGYTCECSQD---------YYGQYCENKVEKPCPRGWWGNP-MCGPCNCDVSKGFNP
MmCelsr2        EHQSVCTRKPNTPHGYICECLPN---------YLGPYCETRIDQPCPRGWWGHP-TCGPCNCDVSKGFDP
DrCelsr2        EHQSACSRRSSSAHGYTCSCPSN---------YFGQYCEKKTDLPCPRGWWGEK-SCGPCNCDTTKGFDA
MmCelsr3        QNQGSCRHLQGAPHGYTCDCVSG---------YFGQHCEHRVDQQCPRGWWGSP-TCGPCNCDVHKGFDP
DrCelsr3        ENQAKCHRKPSSSHGYICDCEDS---------HYGQYCQHRIEQQCPRGWWGSP-TCGPCHCDASKGFDT
DmStan          AS-GVCRANTSLPRGYDCECNSSS--------RHGDYCEKELQQPCPGGWWGER-VCGPCRCDLAQGYHP
ChFmi           NT-GTCKHSSSSLSGYVCDCPIG---------FTGEHCEIAKQKKCPPKTFGKD-YCGPCDCKLHENFNE
TaCelsr         KNNGRCVMNVNVHYGYECQCNKG---------FQGRFCETQFGDKCQPGEFGTPGFCEPCACDRNKNFSH
OcFmi           KHDGLCSHTSENRLGFACKCNSL---------YIGTLCETTNTQWCLSGFYGFP-NCRPCVCNVEGTNSE
OlFmi           QHGGQCAPSDDSERGFVCECASL---------YAGAFCETITLGKCPKGFFDYP-HCRPCVCSVVGATSA
ScFmi           LHNGKCHHSAHDLDNITCDCSGSFPYSRGPSGLCDRIFPDSVKLSCPTGFYDYP-NCVPCRCDWTGAAGA
AqCelsr3L       LHGGTCNFNFYEDDGFTCVCAAP---------YVGLDCQINLGSSCRIGFFRAPEECVPCACDREGTTFD

                      2250      2260      2270      2280      2290      2300      2310
                ....|....|....|....|....|....|....|....|....|....|....|....|....|....|
MmCelsr1        DCNKTN---------GQCQCKENYYKPPAQDACLPCDCFPHGSHSRACDMDTGQCACKPGVIGRQCNRCDN
DrCelsr1b       DCNKTT---------GECSCKDNYFRPLGGDTCYPCDCFHLGSHSRVCDPVTGQCSCKTGVVGRQCNRCDN
DrCelsr1a       DCNKTT---------GECRCKDNYYQPKDSDTCFPCDCFHLGANSRTCDPETGQCPCKAGVIGRQCNRCDN
MmCelsr2        DCNKTS---------GECHCKEKHYRPPGSPTCLLCDCYPTGSLSRVCDPEDGQCPCKPGVIGRQCDRCDN
DrCelsr2        DCNKTN---------GACRCKDNHFRPPSSDTCVLCDCYAVGSLSRVCDRAVGQCQCKPGVIGRQCDRCDN
MmCelsr3        NCNKTN---------GQCHCKEFHYRPRGSDSCLPCDCYPVGSTSRSCAPHSGQCPCRPGALGRQCNSCDS
DrCelsr3        DCNKTT---------GHCHCKEFHYRRRGSDICLPCDCYPVGSFSRSCDPESGQCQCRPGVIGRQCNMCDN
DmStan          DCNKTT---------GQCYCKTNHYQPPNETACLSCDCYSIGSFSGACNPLTGQCECREGVIGRRCDSCSN
ChFmi           TCDPDGQMEPYSYPGKCFCNDDYYLKGG--TCVKCNCLLEGSESSVCDKKTGQCKCKANVIGMKCDKCPG
TaCelsr         ICNITS---------GECICEDKYWKDSNLDSCRPCDCYQYGSTSPICDKKTGSCTCRPGVTGDKCNLCVN
OcFmi           ICDSSS---------GGCLCKVDTYSPKEDVGCQLCEC-SVGATSTTCDQLTGKCQCQPFVVGRRCDQCAD
OlFmi           ICTES----------GSCVCEKDTYSPIDDLGCRPCECNALGAVSSTCDRATGNCECKPFVVGRACDRCAD
ScFmi           VCTDT----------GICLCKDGTFSPANDKGCQPCNCHVHGSVASECDRSTGQCRCKPFTTGRRCDQCMD
AqCelsr3L       ICNRDG-----------VCQCNPDIATYGSDCKPCNCNRTGSNSTECD-DNGKCPCKPGVTGDHCDSCQT
```

```
                   2320      2330      2340      2350      2360      2370      2380
             ....|....|....|....|....|....|....|....|....|....|....|....|....|....|
MmCelsr1     PFAEVT---------------------------------------------SLGCEVIYNGCPRAFEAGI
DrCelsr1b    PFAEVT---------------------------------------------VTGCVVVYDGCPKAFEEGI
DrCelsr1a    PFSEVT---------------------------------------------STGCEVGYEGCPKAFDSGI
MmCelsr2     PFAEVT---------------------------------------------TNGCEVNYDSCPRAIEAGI
DrCelsr2     PFAEVS---------------------------------------------SSGCEVIYDSCPQAIEAGI
MmCelsr3     PFAEVT---------------------------------------------ASGCRVLYDACPKSLRSGV
DrCelsr3     PFAEVT---------------------------------------------QTGCEVIYDGCPKTITSAI
DmStan       PYAEVT---------------------------------------------LSGCEVVYDACPRSFAGGV
ChFmi        AWQEIT----------------------------------------------NGCGEINNSCPRTYVDGI
TaCelsr      KTSEISTDG------------------------------------------GYGCRVVRGACPRSFSGGL
OcFmi        GYAGLD-----EKGCREVTQVEGSLLLTNEVYWSLLGDRTSSQFMILAGALSRAMETLYVGLAGTQEVKV
OlFmi        GYAGLDDDGGAETGCKKVTRVEGSVILTSEIYWSSLADVSSSQFQSLSAALSREMETLYVGLHGTQKVGI
ScFmi        DYTGLD---------------------------------------------ADGCHVLTRVVAEVSLLGP
AqCelsr3L    GFVGFGPEGCREG--------------------VLVSGSFVIDNLYYANGDDTSNQFIKEEILRELESFV

                   2390      2400      2410      2420      2430      2440      2450
             ....|....|....|....|....|....|....|....|....|....|....|....|....|....|
MmCelsr1     WWPQTKFGQPAAVPCPKGSVGN------------AVRHCSGEKG-WLPPELFN-----------------
DrCelsr1b    WWPRTMFGGPAATNCPKGSSGT------------AIRHCSDDKG-WFPSELFN-----------------
DrCelsr1a    WWPRTNFGFPVAMNCPKGSIGT------------AVRHCNDEKG-WLPPELFN-----------------
MmCelsr2     WWPRTRFGLPAAAPCPKGSFGT------------AVRHCDEHRG-WLPPNLFN-----------------
DrCelsr2     WWPRTKFGLPAAVACPKGSIGT------------AIRHCDEHKG-WLSANLFN-----------------
MmCelsr3     WWPQTKFGVLATVPCPRGALGA------------AVRLCDEDQG-WLEPDLFN-----------------
DrCelsr3     WWPRTKFNLPAAVPCPKGSVGT------------AIRHCDGERG-WLDPDLYN-----------------
DmStan       WWPRTPLGGVAIEGCPPPARGK------------GQRSCDVQSGSWNTPDMYN-----------------
ChFmi        WWRRAARGVTQRERCPFNAVGN------------ATRHCDPRKG-WQPPDFFD-----------------
TaCelsr      WWNRSRFNATITMDCPNGASGK------------ASRVCNESTS-WQDPDTSA-----------------
OcFmi        TAFQRAAGERVLAFFTVISYTD------------DIGGASVNELLWVLDVAMSHRQYGSFLVESVELKAD
OlFmi        SSFRRAHGEQVFVSFSIISYSD------------GIDEATVNELLWVLYAAS--EDYGSFRVRDLRLDVD
ScFmi        STSQALRTESQQTRTEIAYLIERFFVGFPGSQHVTVTSLSIVPG--------------------------
AqCelsr3L    LPTTLELNLSLSNNYNVTAVMYN---------LVSDSKAGLISTHFLGSVMQTVMG--------------
                   2460      2470      2480      2490      2500      2510      2520
             ....|....|....|....|....|....|....|....|....|....|....|....|....|....|
MmCelsr1     ------------------------------CTSGSFVDLKALNEKLNRNETRMDGNRSLRLAKALRNATQ
DrCelsr1b    ------------------------------CTSLSFSKLKKASEDLYANASRMDGERSRSLASLLQSATG
DrCelsr1a    ------------------------------CTTITFSHLKKMNDDLHRNESSMDGQKSRDIARMLHNATD
MmCelsr2     ------------------------------CTSVTFSELKGFAERLQRNESGLDSGRSQRLALLLRNATQ
DrCelsr2     ------------------------------CTSVSFSKLKTLSERLVRNASLMDSTNVQQTAALLFNATK
MmCelsr3     ------------------------------CTSPAFRELSLLLDGLELNKTALDTVEAKKLAQRLREVTG
DrCelsr3     ------------------------------CTSPPFVELNTALESLERNETELNTIIEKKLAHQLRDVTE
DmStan       ------------------------------CTSEPFVELRRQLSQLEKLELELNSFVAIKMAEQLRKACE
ChFmi        ------------------------------CTSQLFSNIQTLLKPVLTSGKRMTKDLVTSISYKLDFAVS
TaCelsr      ------------------------------CTSRVYLRLQSQFDG-YKQNPTFTGLQAVLNDLNQQLKTS
OcFmi        CSSDPCFPGSTCSPVPMTAAGRVQSFTCSSCPGPGFAGDGIVCISQQHLCSDKSDLTDVDNDGIGNICDN
OlFmi        CSSSPCFLTSTCS------DSASRNFSCSSCPGPSFSGNGIVCIPRERLCTD-DNSIDTDNDGIGDNCDN
ScFmi        ----------------------------RTAKEGATPRLQVNFAVRSHAYTGGHEASINDIMAALTVGLS
AqCelsr3L    ------------------------------RDCIGRYEIRKNSHDFNADCSLCDPMADCTNSSICEDCPS
```

```
                     2530      2540      2550      2560      2570      2580      2590
                 ....|....|....|....|....|....|....|....|....|....|....|....|....|....|
MmCelsr1         GNS-----------------------------------------------------TLFGNDVRTAYQLL
DrCelsr1b        HTY-----------------------------------------------------RLFGNDVRTAYHLL
DrCelsr1a        QTS-----------------------------------------------------SFYGNDIKTTYHLL
MmCelsr2         HTS-----------------------------------------------------GYFGSDVKVAYQLA
DrCelsr2         NTR-----------------------------------------------------EFYGSDVKIAFHLL
MmCelsr3         QTD-----------------------------------------------------HYFSQDVRVTARLL
DrCelsr3         ATS-----------------------------------------------------RLYGNDLQIAERLL
DmStan           AVDRRGASKDQKISGNGRPNRRYKMESSFLLSNGGNVWSHELEMDYLSDELKFTHDRLYGADLLVTEGLL
ChFmi            APKLTFTP-----------------------------------------------HAFYGKDIMIGYDAL
TaCelsr          GLS-------------------------------------------------------FEHDINICLDVV
OcFmi            CVYEFNPQQSSCQ--SGSNYCQATAFEGVYWPRAPDGAEKSRPCPAG-------LVGTASRRCKGAGWAE
OlFmi            CPYVFNPQQDQCRQDDDSASCPTEMSDDVYWPRTLSGRKSSRPCPAG-------LVGVATRSCRNGEWQK
ScFmi            HSNMLEQYRIVPG---------------------------------------FLQFQANCSDHPCYPDVQ
AqCelsr3L        GSYLDDP----------------------------------------------IICRSSLPQGCSGLDTD

                     2600      2610      2620      2630      2640      2650      2660
                 ....|....|....|....|....|....|....|....|....|....|....|....|....|....|
MmCelsr1         ARILQHESRQQGFDLAATREANFHEDVVHTGSALLAPATEASWEQIQ----RSEAGAAQLLRHFEAYFSN
DrCelsr1b        ASVLEHESLQQGFELTATHDTDFNRNIIKAGSAILDPLNQEHWEKIQ----RSDGGTANLLHQFEEYANT
DrCelsr1a        SSILRYESQQQGFNLAAMRDAKFNENLVKAGSAILDASNKEHWDQIQ----RTEGGTANLLKHFEEYANT
MmCelsr2         TRLLAHESAQRGFGLSATQDVHFTENLLRVGSALLDAANKRHWELIQ----QTEGGTAWLLQHYEAYASA
DrCelsr2         RAILRHESQQQGFNLTATQDVLFNEHLMHVGSAILRAETRGHWQTIQ----QTESGTDTLLRQFEEYTHT
MmCelsr3         AYLLAFESHQQGFGLTATQDAHFNENLLWAGSALLAPETGHLWAALGQRAPGGSPGSAGLVQHLEEYAAT
DrCelsr3         SRLLTFESLQSGFGLTATQDAHFNENVLRGCSVLLGPASASLWRAQ----SSGQSGAAGLADLLEQYTRI
DmStan           QELINYELMQSGLNLSHSQDKYFIKNLVDAASVILDRKYEAEWRRAT---ELIQRGPDDLVDAFNKYLVV
ChFmi            RLMISYEGNQTADHLVSADEEQFVGNLLKSAGKLFQTSLSPFWDSIQKKYSGTAGLMKEMERFVNILSKN
TaCelsr          NNALSYGRQLLGRSNQRANTNEVNTITEKSFNAISELLSRNNLDKWS----KKSNRSSTLLSSVNDYTND
OcFmi            PSLVDCVSDEVAQLLALYEELEQGKVFLESDAIAVVEQLKEITSSPSAENSNIESAIRIVMGVIQYETDA
OlFmi            TSLVECVSDDVERLLSLYEELERGKVLLESEAIAMVEQLRDITADSSSDNSNIEAAIRVAMGVIQYETDT
ScFmi            CQQINATVVSCGSCPVGFIGDGIQCRKDMAGCSSIKNVSEMQYSSIDKDYDGVLDSCDNCPWVYNPDQSA
AqCelsr3L        QDGFPDACDSCPYWFNPRQDYTLPCSETSTESCPREKAGDVLWSQTDKGRVDSRECPTPLIGIATRQCSS

                     2670      2680      2690      2700      2710      2720      2730
                 ....|....|....|....|....|....|....|....|....|....|....|....|....|....|
MmCelsr1         VARNVKRTYLRPFVIVTANMILAVDIFDKLNFTG-------AQVPRFED--IQEELPRELESSVSFPADT
DrCelsr1b        LVQNVRKTYLRPFTIITDNIIVALDFLDSTASQ--------EKIPRFQE--VKEVFSKEMECSVLFPNL-
DrCelsr1a        LAQNMRKTYLKPFIIVTENMVFAVDYLVTYPDA--------TKVPNFQT--TDQECPKDLKSSVLFPEFS
MmCelsr2         LAQNMRHTYLSPFTIVTPNIVISVVRLDKGNFAG-------TKLPRYEA--LRGERPPDVETTVILPESV
DrCelsr2         LAQNMRRTYLNPFTIVTPSIVISIDRLEKMNFAG-------AKLPRYQS--LRGPRPLDLETAVTLPDSV
MmCelsr3         LARNMELTYLNPVGLVTPNIMLSIDRMEHPSSTQGA-----RRYPRYHSNLFRGQDAWDPHTHVLLPSQA
DrCelsr3         LAQNMKQTYLNPVALVAPNIVMTLDRVDNHTHVR-------RRFPRYHSPLFRGQALWDAHTHVVLPPAA
DmStan           LARSQHDTYTSPFEIVQPNMALGLDIVTTESLFGYE----PEQLSEYHRSKYLKPNAFTTESVVLPDTSG
ChFmi            LAYIKRNRNRRSVTLNTYSQATSNIYFEIKPMVR--------------------GNSVDEEKFITLPDSL
TaCelsr          LGKAYIKDKSINNAITYPNVMMRIEEIIVSQYNG-----------------------------LTLPRPS
OcFmi            VSQVVNSQYEGQEKLFKSLLEVISNVLMEDNAYSWERIRKNGSLPGAASLMQKIESMAAAASATDLVSSA
OlFmi            VLQIENSRYATQEKLLTSLLEVISNVLMESSAYAWRRIQENGSLPGAASMMKSVEAMAVAASGTELVSSL
ScFmi            LLCSNAKSARCPAGLETLPPHTVVWRTGRPDHTLQQLCPGTSVGNMTRTCTEHGWQASNRSACQSLPIAA
AqCelsr3L        DGMWLEPNFNNCMTFTGVQLNSILQDLTGEPPSDQNETIAVSIKLESVLSSSPRLYCRDLDSAVFILAKI
```

```
                  2740      2750      2760      2770      2780      2790      2800
            ....|....|....|....|....|....|....|....|....|....|....|....|....|....|
MmCelsr1    FKPPEKKEGPVVRLTNRRTTPLT-----------------AQP--EPRAERETSSSRRRRHPDEPGQFAV
DrCelsr1b   LMSKKSKDALSTDPPSQINSTVS-----------------VMSMDGSDVVDLIPSIKKRSHAEQPDPPAA
DrCelsr1a   FKSSEHKVSPTDDPEFQIN---------------------------SDEEEKQATNKRKRHVETIPPFPV
MmCelsr2    FREMP---SMVRSAGPGEA-----------------------------QETEELARRQRRHPELSQGEAV
DrCelsr2    FSPAPEGKGHRHHSEPIIE-----------------------------PGRNHTANRKRRHPDERQEDAI
MmCelsr3    SQPSPSEVLPTSSN-AENAT---------------------------ASSVVSPPAPLEPE--SEPGISI
DrCelsr3    LVPQRNVFSAPSPAGPVNLL---------------------------ANQTLEATAARRSLSLQEAPVSI
DmStan      FLQHSARQRPVISFPKYNNYILDRRKF-----DQHTKVLVPLEMLGITPPESDEISQSGRRGSSHDHRAI
ChFmi       SPRHIDYYMWKDPSVSVRIS---------------------------------DAIFLDHDELFRINEVN
TaCelsr     DSGSVSNITDRISLPNDLVK-----------------------------------------KGSSDESVP
OcFmi       RGNQITWAASHIALHMVSFSDRPPSSD-----VIFPVNDFAFRPIGPFQFKEGTAVSLPADIFPSSGTNG
OlFmi       PGGEITWAASHIALHLVSMNDRRRS-------FVFPSAIVALQPLG--LLKRGTGVSLSADNFP--LAKG
ScFmi       LMNEFESLEAGHIFSAAEASQWMLR----------------------LQTAVRKATHSSSGSTPVVSDHT
AqCelsr3L   LNLDTLLPLTSSGLKPLAEVLGLLGSSKLAEVWEETGCNAGIILNLTEHLGVLSASSLSIGESLTVEGDG

                  2810      2820      2830      2840      2850      2860      2870
            ....|....|....|....|....|....|....|....|....|....|....|....|....|....|
MmCelsr1    ALVVIYRTLGQLLP-EHYDP------------------------------DHRSLRLPNRPVINTPVVSA
DrCelsr1b   VMVMIYRSLGHLLP-ESYDP------------------------------DRRSLRLPNRPVINTPIVSV
DrCelsr1a   ASVIIYKTLGQFLP-EHYDP------------------------------DRRSLRVPNRPVINTPIVSA
MmCelsr2    ASVIIYHTLAGLLP-HNYDP------------------------------DKRSLRVPKRPVINTPAVSI
DrCelsr2    ASIIIFHTLATLLP-ERYDT------------------------------DKRSLRVPKRPVINTPVVSI
MmCelsr3    VILLVYRALGGLLP-AQFQA------------------------------ERRGARLPQNPVMNSPVVSV
DrCelsr3    VIILIYRSLGAALP-PKYHT------------------------------DRRGVRLPRHPVLNSPVVSI
DmStan      VAYAQYKDVGQLLP-DLYDETITRRWGVDVELATPILSLQILVPSMEREQETQRLEIPSRKIFSSSSPSS
ChFmi       IGLIIYKTLGHLLP-TNFDQ------------------------------DNREEGRKYALNVYSDVVTL
TaCelsr     IVYLKFDTIGQLLP-GKSDVVAP------------------------------YDIMVTSTVLSCKVVGV
OcFmi       VAFVVYNTLHKILS-NSFEA--------------------------------LGNNSGSKWSIASHIISV
OlFmi       VAFVAYNTLHKVLP-SLFDS--------------------------------LGNTYGSGWFVNSPIISV
ScFmi       AATLPYPGDVKVVY-HILNLMLQYG---------------------------TSLMMSNLETLSEDFIST
AqCelsr3L   LSMSVSAYNGRVYSGYEFNVNGSTATGVSVPNGVIPLADNDNFSISLTHYSDLSLSLPYTDLSPDAIVGS

                  2880      2890      2900      2910      2920      2930      2940
            ....|....|....|....|....|....|....|....|....|....|....|....|....|....|
MmCelsr1    MVYSE-----------------------------------------------------------------
DrCelsr1b   VVHKD-----------------------------------------------------------------
DrCelsr1a   TVYSE-----------------------------------------------------------------
MmCelsr2    SVHDD-----------------------------------------------------------------
DrCelsr2    TVHDN-----------------------------------------------------------------
MmCelsr3    AVFHG-----------------------------------------------------------------
DrCelsr3    SVYNN-----------------------------------------------------------------
DmStan      SSSSGSTEQQFVEVFDVPKAPTSSSEQQIEDIRITAHEIPPPVSSVEQQEASSDEDGEEREPHIRLNLDD
ChFmi       TIP-------------------------------------------------------------------
TaCelsr     D---------------------------------------------------------------------
OcFmi       GMSDS-----------------------------------------------------------------
OlFmi       GVSGS-----------------------------------------------------------------
ScFmi       SLDVAD----------------------------------------------------------------
AqCelsr3L   PVWSIQLLMN------------------------------------------------------------
```

```
                    2950      2960      2970      2980      2990      3000      3010
                ....|....|....|....|....|....|....|....|....|....|....|....|....|....|
MmCelsr1        ----------------------------------------------------------------------
DrCelsr1b       ----------------------------------------------------------------------
DrCelsr1a       ----------------------------------------------------------------------
MmCelsr2        ----------------------------------------------------------------------
DrCelsr2        ----------------------------------------------------------------------
MmCelsr3        ----------------------------------------------------------------------
DrCelsr3        ----------------------------------------------------------------------
DmStan          IEFHGNSGEEVISPDSPEMLNPNYEGVSSTGSDEQPKGENEAVYRDRRLVKRQVEITYPSEQMQQTEQVV
ChFmi           ----------------------------------------------------------------------
TaCelsr         ----------------------------------------------------------------------
OcFmi           ----------------------------------------------------------------------
OlFmi           ----------------------------------------------------------------------
ScFmi           ----------------------------------------------------------------------
AqCelsr3L       ----------------------------------------------------------------------

                    3020      3030      3040      3050      3060      3070      3080
                ....|....|....|....|....|....|....|....|....|....|....|....|....|....|
MmCelsr1        -----GTPLPSSLQRPILVEFSLLETEE-------------------RSKPVCVFWNHSLDTGGTGGWSA
DrCelsr1b       -----GEPLLSPLQRPILLNFRLLETQE-------------------RTKPVCVYWNHSIMVAGGGAWSS
DrCelsr1a       -----GQPLHIPLERPITLDYNLLETEE-------------------RTKPVCVFWNHSITVGGAGAWSS
MmCelsr2        -----EELLPRALDKPVTVQFRLLETEE-------------------RTKPICVFWNHSILVSGTGGWSA
DrCelsr2        -----EELLQHTLDKPIIVQFRLVTTEE-------------------RSKPICVFWNHSIVAASPGGWSS
MmCelsr3        -----RNFLRGVLVSPINLEFRLLQTAN-------------------RSKAICVQWDPPGPTDQHGMWTA
DrCelsr3        -----QTFVEGFLESPVLLEFRLLETSN-------------------RSKPLCVQWNHSSIVSGGGCWTV
DmStan          YRSLGSPHLAQPIKLQMWLDVDSARFGP-------------------RSNPQCVRWNSFTNQWTRLGCQT
ChFmi           ------SIKSLHPLTEIQITYKNVSQLNS-----------------TSTSLVCAFWNYSLSGTVSGGWSP
TaCelsr         ---------TSSLSSPITLTLSNTKKVSN------------------VQAHECVYWKKDLKLTEGGRWST
OcFmi           ------NINTSFLAAPVNLTFSHNQPLN-------------------ESSTECSFWKFQDKGTLGGSWST
OlFmi           ------NLNTTKLAFPVNLTFVHTQVLHRVRITYSNILAVFLRKELNRSSIECGFWNFKDKQNPEGSWST
ScFmi           -----VLFSGKQKAAWKLMDVSHQSTVAG-----------------LMVQLESFSMAHALSVAGDDAGVA
AqCelsr3L       ------GGRVTQLATPINITFPHPIEVNS------------------SLTSLCVTWN-----ATRNKWVE

                    3090      3100      3110      3120      3130      3140      3150
                ....|....|....|....|....|....|....|....|....|....|....|....|....|....|
MmCelsr1        KG------CELLSRNRTHVTCQCSHSASCAVLMDISRRE--HGEVLPLKIITYAA---------LSLSLV
DrCelsr1b       KG------CELVFRNSTHISCQCSHMSSFAILMDISKRE--HGDVLPLKVVTYTT---------VSASLV
DrCelsr1a       KG------CDIISRNHTHISCQCNHMTSFAVLMDISKRE--HGDVLPLKIVTYTT---------VSASLL
MmCelsr2        RG------CEVVFRNESHVSCQCNHMTSFAVLMDMSRRE--NGEILPLKTLTYVA---------LGVTLA
DrCelsr2        KG------CEVVFRNSTHISCQCYHMTSFAVLMDISRRE--NGEILPVKLVTWSS---------VGATLF
MmCelsr3        RD------CELVHRNGSHARCRCSRTGTFGVLMDASPRERLEGDLELLAVFTHVV---------VAVSVT
DrCelsr3        RD------CSVLYRNTSHVRCQCHRLGTFGVLMDSSQREQLEGDLETLAIVTYSS---------LSVSMA
DmStan          EIPDFDGDFNPAAQQAILVNCSCTHISSYAVIVDVIDPEDIPEPSLLVQITSYSA---------FLVSLP
ChFmi           NG-----CVTKMEVNSSDVTCVCNRMASFALVGFQYTEI--IPRVYQPAFLVYIA---------LAVAML
TaCelsr         DG------CSLISRDSSKTVCQCNHLTDFGVLSRYRSKPLPSVTPIKLELVTYIG---------IGISLL
OcFmi           EG------CTTVFSNSTQTTCQCHHLTSFGVAADD------KSGLIDLNLFVIIS---------CCFTIG
OlFmi           SG------CSAISTTSTSTTCQCDHLTSFAVLSAD------KSGSIDLDLFVIIG---------CCFTIG
ScFmi           RQS---HVLENMYVEYGMMSNGSSTFPTSSMSSNLSEFSISSNVPSGYAAFLQFD---------DAADLM
AqCelsr3L       SG------CQATVNNPNSTSCSCPHLSDFTVLIVPYEEVFVCLVLYTILHLIRASKDQSHIQNRVNATLF
```

```
                     3160      3170      3180      3190      3200      3210      3220
                ....|....|....|....|....|....|....|....|....|....|....|....|....|....|
MmCelsr1        ALLVAFVLLSLVRTLRSNLHSIHKNLITALFFSQLIFMVGIN----QTENPFLCTVVAILLHYVSMGTFA
DrCelsr1b       ALLITFLLLAILRKLRSNLHSIHKNLVASIFLSEFIFLTGIN----QTDSPFVCTVVAILLHYSYMCAFA
DrCelsr1a       ALFITFILLAILHKLRSNLHSIHKNLVAALFFSELVFLIGIN----QTDNPFVCTVIAILLHYFYMCTFA
MmCelsr2        ALMLTFLFLTLLRALRSNQHGIRRNLTAALGLAQLVFLLGIN----QADLPFACTVIAILLHFLYLCTFS
DrCelsr2        FLLLTIIFLSCLRAANSNKTSIAKNGSSALFIALLVFILGIS----QADNPFVCTLMAILLHFFWLCVFS
MmCelsr3        ALVLTAAVLLSLRSLKSNVRGIHANVAAALGVAELLFLLGIH----RTHNQLLCTAVAILLHYFFLSTFA
DrCelsr3        ALLLTVLVLSCLRGLKSNTRSIHSNMAAATLLSHLTYLLGIN----QTEQQFLCTVVAILLHYFFMSAFA
DmStan          LLLGVLLALALLRGQQTNSNTIHQNIVLCVFCAELLFFVGMQSRRQLLESEFPCKLTAICLHYFWLAAFA
ChFmi           LFLIVFLVFLCLSQLKSNANSIHKNLAFVLLLGWIVFTFAIN---RPDVGKANCRIIAILIHYCLTCAFS
TaCelsr         LLVSVFLCLTVMRSVNGSTNTIHCHILINLILVESLFLIGIT----QTASTMICIAISTLLHFFLLVVFS
OcFmi           CLVILTLASCCSRKASSDRMLVLLNLDLAIVMSELFFVIGIG-----QENSDTCTAMAVILHFFCAAIFS
OlFmi           CLTVLLLVLYCSRKSNQDRIVVFANFDLAMLFTELFFVIGIG-----QEKAGVCVAMAVVLHFFCTAVFA
ScFmi           NARVVKSAGEIASEWYLNSAVVMARFQATGSVPTSGFNGTVS---FQLRRRNTAASGEAICAFWNPQLQQ
AqCelsr3L       LVFLFIFIVINLSFTMLTLHSIQWSPPFSTVFSLIVILSVVTS--AVLIFTVLCCTKRPKIFKLNIESGS

                     3230      3240      3250      3260      3270      3280      3290
                ....|....|....|....|....|....|....|....|....|....|....|....|....|....|
MmCelsr1        WTLVENLHVYRMLTEVRNIDTGPMRFYHVVGWGIPAIVTGLAVG------LDPQGYGNPDFCWLSLQD-T
DrCelsr1b       WMFVEGLHIYRMLTEMRNINQGHMRFYYAIGWGIPAIITGLAVG------LDPQGYGNPDFCWLSVYD-T
DrCelsr1a       WTFVEGLHIYRMLTEVRNINHGHMRFYYAIGWGIPAIITGLAVG------LDPQGYGNPDFCWLSVHD-T
MmCelsr2        WALLEALHLYRALTEVRDVNASPMRFYYMLGWGVPAFITGLAVG------LDPEGYGNPDFCWLSVYD-T
DrCelsr2        WLFLEGLHVYRMLSELRDINYGPMRFYYLIGWGVPAFITGLAVG------LDPEGYGNPDFCWLSLYD-T
MmCelsr3        WLLVQGLHLYRMQVEPRNVDRGAMRFYHALGWGVPAVLLGLAVG------LDPEGYGNPDFCWISIHE-P
DrCelsr3        WLFVEALHIYRMQTEARNINYGAMRFYYAIGWGVPAIITGLAVG------LDPEGYGNPDFCWISMYD-K
DmStan          WTTVDCVHLYRMLTEMRDINHGPMGFYFAMGYGAPAIVVGLSVG------VRAHEYGNSLFCWLSVYE-P
ChFmi           WLMVEALHMYRMILEPRDINYGQMMFYYFIGWGAPVIVVGVTAG------LKPDGYGTPEFCWISAKVND
TaCelsr         WICIESIYMYRKLKETKKTTKCHLLFCLLPGYGIPAAIATATTA------ISLSYYHNKRYCWLSVEH-G
OcFmi           WVVVEILGIYYLAA-VKNAEKTKIPYYLGVGWGLPALITVITVG------IRLDAYKGNSYCFLTLKE-S
OlFmi           WVVVEILNVYYIVS-AKDSEKTKIPYYLGVGWGFPALIVAITVG------IRLNAYEGNLYCFLTLDD-D
ScFmi           WSDSGCSMTAATVTHITCVCNHMTAFGVLERAALPVTRLTMHVL------ATASCAVSAVLFLVILAL-V
AqCelsr3L       SEAAKEFQQYSNNSSCIEDENTQPIYHTIGNRTEELYQEPVPVPPINQLNPGFQASSIPEYHELEQQGDG

                     3300      3310      3320      3330      3340      3350      3360
                ....|....|....|....|....|....|....|....|....|....|....|....|....|....|
MmCelsr1        LIWSFAGPVGTVIIINTVIFVLSAKVSCQRKHHYYERK-----GVVSMLRTAFLLLLLVTATWLLGLLAV
DrCelsr1b       LIWSITGPISIVVLINIVLIVLAAKASCGRRQR-TEKS-----GAISALRVAFLLLLLISATWLLGLMAV
DrCelsr1a       LIWSFAGPIAVVVLVNIVIFVMAAKASCGRRQRSYEKS-----GVTPALRMAFLLLLLISATWLLGLMAV
MmCelsr2        LIWSFAGPVAFAVSMSVFLYILSARASCAAQRQGFEKK-----GPVSGLRSSFTVLLLLSATWLLALLSV
DrCelsr2        LIWSFAGPIAFVVSMNLFLYIMASRASCSLRQKSCEKRD----VPAAGLRTACTVLFLVTVTCLLALLSV
MmCelsr3        LIWSFAGPIVLVIVMNGTMFLLAARTSCSTGQREAKKT-----SVL-TLRSSFLLLLLVSASWLFGLLAV
DrCelsr3        LMWSFAGPVSVVILMNGGMFLMVLRMTCNPTQKEIKKL-----PVISTIRSAFFLLLLSTCVWLFGLMAV
DmStan          VVWWLVGPIAGMSVVNLLILFVSVKAAFTLKDHVLGFG-----NLRTLLWLSVVSLPLMGVMWVLAVLAA
ChFmi           SVWTYVAPIFAIIAGTFIIIILALASSCEKANIKGKKAK--LARIRYRLAISFFFLFIMMVTVFGGLLEV
TaCelsr         LIWSLGCPAACIIGISLVFFCLTYHKIRFRSNSMTRWNRKEIDSDKFDLRSGMLILLFITAAWVFGVIAV
OcFmi           DVWAFSGLAVLSIAAFFIVFAMIFDSRKKLKKKLSPDDDQ-LPSVKMPVVCSCLILLFLVTLFVFGRFYL
OlFmi           VIWAFGVVAVLAIAAFFIIFCMIFDGQKKLRKRSDADDEK-LPSIKLPLVGSVLTLITLIVLFAIGYFYL
ScFmi           IAYRKRSPAMLIVSYFGIAAGMFLLEVVFAVGLFSWDLS----SVTCTSVAAILQYLLLAVACNVFLLFL
AqCelsr3L       SNSDSDSENEIDVDVEALQQQTREEREIRERRESNRRISARLEEEKRRRSAGDENISSITDPQQQEAWFN
```

```
                   3370      3380      3390      3400      3410      3420      3430
             ....|....|....|....|....|....|....|....|....|....|....|....|....|....|
MmCelsr1     NSDTLSFHYLFAAFSCLQGIFVLLFHCVAHREVRKHLRAVLAGKKLQLDDSATTRATLLTRSLNCNNTYS
DrCelsr1b    NSDVLSFHYLFAILSCLQGICIFFFHCILNKDVRRNLKSVFTGKNIPAEDPSVTRATLLTRSLNG-DVYM
DrCelsr1a    NSDVMTFHYLFAIFSCLQGVFIFFFHIVFNKEVRKNLKNVFTGKKQLPDESSTTRTTLLTRTLNCNNTYM
MmCelsr2     NSDTLLFHYLFAACNCVQGPFIFLSYVVLSKEVRKALKFACSRKP--SPDPALTTKYTLTSSYNCPSPYA
DrCelsr2     NSDIILFHYLFAGFSCVQGVFIFFLRVVFNQEARDAMRYCCSGK---RPDHMIKSK---PAAFLCSSNYV
MmCelsr3     NHSILAFHYLHAGLCGLQGLAVLLLFCVLNADARAAWTPACLGKKAAPEET-RPAPGPGSGAYNNTALFE
DrCelsr3     NNSVLAFHYLFIILCCIQGLAVLLVFTVLNSEVQEAWKLACLGKKSPGEEPPRPPQITGQNPYNSASLLE
DmStan       SEHSQLLSLLLSGVVLLHALFCLIGYCIINKRVRENLQRTCLRCMGRKVPLLDSSMVVSNSSHNVNAAAR
ChFmi        SFDMTLLTYIFAGTCALEGVYMFLFYVCFNRKVRREAYNAYKRYSTGNKSYGLKPPPKRRGHRFGEKEAL
TaCelsr      QNETVYFQYIFTMVNFIKSACILICYCISNESVRSELSARLCGREGTYYTGTIDKGQLNGHRSNGLNGHF
OcFmi        INSTSAWSYGFGIINLLLGLVLFITQLLQISKLRKWYMGESSKSRGQYHVSSAQPTRSILSGSFGGDKTN
OlFmi        PRSSSSLKYAFGTFNILLGFVFLACQILQVSKEVRYIR----------HMSVFL-------------IVD
ScFmi        RVTVCGACPNEKSRLSLYSPVSLVLFTVVFGYIAPTAVPVLCNALLYSHDFGNTYFCWISRHASGDSLWL
AqCelsr3L    TLDPSSLCAPAAPVTYANGTQLHADSPISTDAHELADLHNEIDNLYQMKSAVLKETVLTADTSSGDYVDL

                   3440      3450      3460      3470      3480      3490      3500
             ....|....|....|....|....|....|....|....|....|....|....|....|....|....|
MmCelsr1     EGPDMLRTALGESTASLDSTTRDEGVQ-----------------------KLSVSSGPARGNHGEPDASF
DrCelsr1b    EDGGLYRTTIGESSVSLQSSVRSGKSHGSS-----------YLASTFREKKRSISSNGGHVGHDEVDSTL
DrCelsr1a    EDGALFRSAIGESTVSLESTVRDELGQ-----------------------KPSVSGN-AKAGLTDIDGSL
MmCelsr2     DG-RLY-QPYGDSAGSLHSASRSGKSQPS-------------YIPFLLREESTLNPGQVPPGLGDPSG-L
DrCelsr2     DG-GLYHLPFAESSVSLN-GTHSSKTQQN-------------YMPFSLRG-NDMNSSHLS--LSEQNT-L
MmCelsr3     ES-GLIRITLGASTVSSVSSARSGRAQDQDSQRGRSYLRDNVLVRHGSTAEHTERSLQAHAGPTDLDVAM
DrCelsr3     QS-GLHRITLGTSTISSVSSAR-----------------ENLLARQ--TLEHTL----AHTGATDLDVAM
DmStan       PSNFLASGYDTTTRRNIGISASSTTSRSTAKTSS-----SPYSDGQLRQTSTSTSNYNSASDAPSFLRGF
ChFmi        IGVNKQYAHVFEAFHLDSTSSNESSDTTDGR----------------RRARAKFAMSDTSTDFQTHDSSD
TaCelsr      NGRIDTSRGHGFAGPNAYPVARSYDHVDGSNVLG----VPDNEAWREFTDAYEKSAIAADGDSVGSSSEE
OcFmi        LSVSFTGTYKSHPYTGNPMFSSGPQWDRNVP-----------------DQSYDAMEMDREEDWDEEGIAF
OlFmi        LAYKSHGTVQAYP-VANPMY--GHHWDHGE-------------------QSYDEIDNDREEDWDED--AL
ScFmi        FYVPPAALCTVTFILAMGLAGASRNATHQAR-------------------WNTVWMIVFCLGFGCAFLLL
AqCelsr3L    KDDTPAVNNNWYEDGSSSSNHRPRDVTDNMYAN--------ATNTSPFDDAQYCTVSEARQYTPDVPRRF

                   3510      3520      3530      3540      3550      3560      3570
             ....|....|....|....|....|....|....|....|....|....|....|....|....|....|
MmCelsr1     IPRNSKKAHG------PDSDSDSELS--LDEHSSSYASSHTSDSE---DDGGEAEDKWNPAGGPAHSTPK
DrCelsr1b    FFHKSKKKE--------DSDSDSELS--ADEHSSSYASSHSSDSE---DEERHSKTKWNNERTPIHSTPK
DrCelsr1a    FRN-GTKAD--------DSDSDSELS--VDEHSSSYASSHSSDSE---DEDIDMQPKWNNERQPLHSTPK
MmCelsr2     FLEGQAQQHD------PDTDSDSDLS-LEDDQSGSYASTHSSDSE---EEEEEAAFPGEQGWDSLLGPGA
DrCelsr2     FKD-KEQMDD------PDSDSDSDLSELEDDQSGSYASTHSSDSE---EEEE-GAFQ-EECWENLATS--
MmCelsr3     FHRDAG----------ADSDSDSDLS-LEEERSLSIPSSESEDNGRT-RGRFQRPLRRAAQSERLLAHP-
DrCelsr3     FHRNGGTLTHNNLHACEDSDSDSDLS-LEEERSLSIPSSESEDNVRL-RGRIQRRFKRTGHGERLLTEAT
DmStan       ESSTTGRSRGGEEKPSRRQRKDSDSGSETDGRSLELASSHSSDDDESRTARSSGTHRSTAVSSTPAYLPN
ChFmi        SSDDDSTTTG-----PRKRPADSDSDSSDDNSSGMDEFSTPTTTTGNGSTPMGGVEWKQATSKKLALSRT
TaCelsr      EDVREVLEQNYASTSSDDEKGNWKAPSRQASVPPNQDGSRSATPSGSQVDKSQESVSVHSSKESLHSHEP
OcFmi        HGFPMSQLHR-------HRGDGADCVDVAQSESLDLGLRGDESVS--EMAPVVYSQVKAHVERDWFEEED
OlFmi        HQFPISQLQR-------HQGDGADCVDVMHQDGSEEMQLGNESISDDKASYPMADDAGVTYDKDWFEDED
ScFmi        PYFMAEYPQS--------RAPVVLFAITNVIWSFVLILLLVRMHRQNRRYHVKNDFDARPAVQKYTSEPK
AqCelsr3L    SLQNQAQQYSSIDQLPPQPPPMQQFADHTYQAIRGQNSPNASPRRTSLNLQGRRQSNDGRPVARRLSNQT
```

```
                      3580      3590      3600      3610      3620      3630      3640
              ....|....|....|....|....|....|....|....|....|....|....|....|....|....|
MmCelsr1      ----ADALANHVPAGWPDESLAGSDSEELDTEPHLKVETKVSVELHRQAQGNHCGDRPSDPESGVLAKPV
DrCelsr1b     ----VDGVSGHGKPYWPEEMPTASESEDMVRPEKLRVETKVNVELHQGNKLNHNGDLSQS--EGPPS---
DrCelsr1a     GTKEVDTVSNHVKPYWPTEAMTASDSEDPGGAERLRVETKVNVELHQENKLNHIGESTQD--KEQPTPSI
MmCelsr2      ----ERLPLHSTPKDGGPGSGKVPWLGDFGTTTKENSGSGALEERPRENGDALTREGSLG----------
DrCelsr2      ------------------GSGKP-----LGVCEGEIRATDETVDKAEDHG----TENLLL----------
MmCelsr3      ----KDVDGNDLLSYWPALGECEAAPCALQAWG---SERRLGLDSNKDAANNNQPELALTS-------GD
DrCelsr3      HNGGKDLDGNDLLSYWPALEGCEAH--SLQKWG---SERRLGGDYNKDAANNNQPDAALTS-------GD
DmStan        ITEHVQATTPPELNVVQSPQLFPSVNKPVYAPRWSSQLPDAYLQSPPNIGRWSQDTGSDNEHVHGQAKMT
ChFmi         YASDGPMHSTPSESEASERLKWKTGVTPKKSDLSAINSESDISRSEKRPVRKGPKGPTRIS---------
TaCelsr       QVLLRQNQLIPARSSSHVYSTIDETMLEPTSPVSPVSPKNKVIAKGSEKVVVRNNVPTSFSFQEDRINPP
OcFmi         ---VDDIDKPPLDDVVEEPEDVSSDEIDKRGTR-FQHHRDSAMDT-LRTKGTYLQSPTHKS-----SEEK
OlFmi         ---FDN-DKPPLEDVAEEPEEFDLEEIHERSNRGRARYPLSAVDSPARTKGTYLQSPVHKS-----SEEK
ScFmi         ---QSILNPPSALPAAAPLSEQHFVPMPHTTNSGSSHSRRSHSSHRTPPPVWYLVEGTSGG--ITASKHS
AqCelsr3L     RNPGVSASVQLMNGGSLAPRSVLPSIMTTTARVAIRIAIGVLIPLLYLNEATVLARDTTYSSSLTNGTVR

                      3650      3660      3670      3680      3690      3700      3710
              ....|....|....|....|....|....|....|....|....|....|....|....|....|....|
MmCelsr1      AVLSSQPQEQRKGILKNKVTYPPPLPEQPLKSRLREKLADCEQSPTSSRTSSLGSGDGVHAT--------
DrCelsr1b     QPNSNQLP--RRGILKNKITYPPPLTDKNMKNLLREKLSDYNPPQISRKTPSPGSNEGLHSGP-------
DrCelsr1a     QANSNHQPEQRKGILKNKITYPPPLTDKNMKNKLREKLSDYNPPTISSRAPSITSNDGGNG---------
MmCelsr2      PLP-GPSTQPHKGILKKKC--LPTISEKSSLLRLP--LEQGTG---SSRGSSISEGSRHGPP--------
DrCelsr2      LLPSLPHTQPHKGILKKKQ--LSPIAERNAINRIHNQLSGDSA---SSRGPSSNEDRGGG----------
MmCelsr3      ETSLGRAQRQRKGILKNRLQYPLVPQ-----SRGTPELSWCRAATLGHRAVPAASYGRIYAGGGTGSLSQ
DrCelsr3      EN--GLTHRHRKGILKNRLGCPPALQGLSAVGRAPSEMSWYRTSTLGHRGVPAASYGRMYSG--TGSLSQ
DmStan        ISPNPLPNPDLTDTSYLQQHHNKINMPPSILENIRDAREGYEDSLYGRRGEYPDKYGSYKPPSHYGSEKD
ChFmi         SSNTDSDTQSQSKAPVSILKKKSQYDSKGHRLRAVRVKDSQRKPLMSMNETADLNDSQV-----------
TaCelsr       NCRSNPPANKRPGSSFSGMQPPLAIEEDEDDDMEVTSFPNKSYDKQQHAQPPRSIYAKSSVAHQNEASMM
OcFmi         TTVSP----MSSGIEG--VMVRRAVESSSRESSIPQQKINHWKSQGGDDDSSSLSSSSTDSLSFGKGRGQ
OlFmi         TTVSPMTVEMSPGIEAGVVVVRQAIETSSRESSIPHQKPLWSMPKRRDVNDDDDDDDS------------
ScFmi         AHTTTGSAASSALAAAAEYVSPYSLPPPPARVPTPPRISDFPTNPQDKQRPKQQQPRRNSAP--------
AqCelsr3L     PTSCKEIYQSDAESKTGEYTLYDAVTGEAYSATCIMGTFKYCNKEAGLTQIFTFENGNKCPRGFVQTSSK

                      3720      3730      3740      3750      3760      3770      3780
              ....|....|....|....|....|....|....|....|....|....|....|....|....|....|
MmCelsr1      ----------------------------------------------------------------------
DrCelsr1b     ----------------------------------------------------------------------
DrCelsr1a     ----------------------------------------------------------------------
MmCelsr2      ----------------------------------------------------------------------
DrCelsr2      ----------------------------------------------------------------------
MmCelsr3      PASRYSSREQLDLLLRRQLSKERLEEVPVPAPVLHPLSRPGSQERLDTAPARLEARDRGSTLPRRQPPRD
DrCelsr3      PASRYSSREHLDSLARRQRSRDNLDLLPR-RRELGAEHLGGSRERLGPVHSIHASREDLVGRGGLAGLMG
DmStan        YPGGGSGSQTIGHMRSFHPDAAYLSDNIYDKQRTLGSGYLGAKSESPYLSKDRITPDIYGSRDGHYSLKR
ChFmi         ----------------------------------------------------------------------
TaCelsr       PGGPRHLVPKPPMRKGHPQPPPRSNPVKP-----------------------------------------
OcFmi         VKGKGRHGKAQQRRRHLAGVEAGQHFSSASSLHSDFNSSVTSSQRNHRRG--------------------
OlFmi         ----------------------------------------------------------------------
ScFmi         ----------------------------------------------------------------------
AqCelsr3L     DSCVRSRRKYGGCTSIPVAGHGILYNKVCVMVYGYQVGSPDGVTGTDRIRFVEGPYVDGVSITHGIPRKH
```

```
                 3790      3800      3810      3820      3830      3840      3850
             ....|....|....|....|....|....|....|....|....|....|....|....|....|....|
MmCelsr1     ----------------------------------------------------------------------
DrCelsr1b    ----------------------------------------------------------------------
DrCelsr1a    ----------------------------------------------------------------------
MmCelsr2     ----------------------------------------------------------------------
DrCelsr2     ----------------------------------------------------------------------
MmCelsr3     YPGTMAG-RFGSRDALDLGAPREWLSTLPPPRRNRD-------LDPQHPPLPLSPQRQLSR-DPLLPSRP
DrCelsr3     AEGSLSGSRTQLNTLTRQQASREHLGGALMSSRSREQLESNGGAQPCREWLRTLPPRQLSHPDPHPPSSP
DmStan       QPAYATDSLHSVHSLLKNDYHQQQQQQQQHHLQDRLS---------------------------------
ChFmi        ----------------------------------------------------------------------
TaCelsr      ----------------------------------------------------------------------
OcFmi        ----------------------------------------------------------------------
OlFmi        ----------------------------------------------------------------------
ScFmi        ----------------------------------------------------------------------
AqCelsr3L    IWTFMASSSEKKPICPCAPG--------------------------------------------------

                 3860      3870      3880      3890      3900      3910      3920
             ....|....|....|....|....|....|....|....|....|....|....|....|....|....|
MmCelsr1     ----------------------------------DCVITIKTPR-REPGREHLNGVA-------------
DrCelsr1b    ---------------------------------ETPSIIIKPP--PVPPKPALNGLT-------------
DrCelsr1a    -----------------------------------HNVLIKPPRPPVPPREHLNGMA-------------
MmCelsr2     -------------------------------------PRPPPRQSLQEQLNGVMPVA-------------
DrCelsr2     -----------------------------------------------ARRNHVNGAS-------------
MmCelsr3     LDSLSRISNSREGLDQVPSRHPSREALGPAPQLLRAREDPASGPSHGPSTEQLDILSSILASFNSSALSS
DrCelsr3     PPPIT-----EEPHESLPSRRGRLDSAPPCRYPSSSAAAPGAPSSRPPSSEHLDILSSILASFSSSVLTP
DmStan       ------------------EGSDKNGYHFPYTAEEDHLPARKLSHTQPPSLHGSQLMQPPGVGLVNDVNNP
ChFmi        ----------------------------------------------------------------------
TaCelsr      --------------------------MRQQASLSTFDAESAVSSQGRPSQRSRHGNGPVAVQHSVSDAAI
OcFmi        -----------------------------LGSTDRHSGRRKYGRRASPGRGGSHSGGNLSRSHSMEGAPR
OlFmi        ----------------------------------------------------------------------
ScFmi        -------------------------------------RRRQEGRTVFTTLAVLDASTR------------
AqCelsr3L    ----------------------------STVKVPDFIGNDYFCESGNPEETAIPGKIYKEDMIWDMEQCE

                 3930      3940      3950      3960      3970
             ....|....|....|....|....|....|....|....|....|....|..
MmCelsr1     ------------------------MNVRTGSAQANGSDSEKP----------
DrCelsr1b    ------------------------LDLTTRPVTLADDDFHSDGSNETSI---
DrCelsr1a    ------------------------MNLKSATVN-GGNQSDSDGSNETSI---
MmCelsr2     ------------------------MSINAGTVDEDSSGSEFLFFNFLH----
DrCelsr2     ------------------------VSGLMKTLDNDSSESEC-----------
MmCelsr3     VQSSSTPSGPHTTATASALGPSTPRSATSHSISELSPDSEVPRSEGHS----
DrCelsr3     PPNPAGPSP----------SPPPPLSATSQSVSEVSPDSEVNRSDGQS----
DmStan       GLMGRHTLNGGSRHSSRASSPPSTMVAPMQPLGPLTSITDTERNIDDDETTV
ChFmi        ----------------------------------------------------
TaCelsr      SFHNNLSSSGELRNSKQNDLHRSSHSLTSSNGSDAIAIAVRRGSSSTK*---
OcFmi        RYKVPRFDKPVGQIVSPTRRGTAEAYLTRGHQHQEEKHEIATKSDVSTAV--
OlFmi        ----------------------------------------------------
ScFmi        -----------------------SSPAPTMSDMPKVALVVVLCRFSICG---
AqCelsr3L    GVEGSCCKSTHKYAYVELPHYTTDDLEIRICSDEDSAEEDFALSMAAISVY-
```

Abbreviations. Vertebrate: Dr, *Danio rerio* and Mm, *Mus musculus.* Hexapoda: Dm, *Drosophila melanogaster.* Cnidaria: Ch, *Clytia hemisphaerica.* Placozoa: Ta, *Trichoplax adhaerens*. Porifera: Aq, *Amphimedon queenslandica*; *Oc, Oscarella carmela*; *Ol, Oscarella lobularis*; Sc, *Sycon ciliatum*.

Figure S2.2: Fuzzy alignment. Threshold for shading has been fixed at 80% for both identity (black) and similarity (grey).

```
                10        20        30        40        50        60        70
        ....|....|....|....|....|....|....|....|....|....|....|....|....|....|
DrFuz   ---MLQAE----------TLQLLCLTASSGVPLFSRGSS------KQLPFSVIGSLNGVHMFGAGHGAQL
MmFuz   MGDEGPGS----------PVHLLCLAASSGVPLFCRSSSGGAPSRQQLPFSVIGSLNGVHMFGQNLDVQL
DmFy    -----------------MSIYLLCLTTNGGLPVFTRKKG----ECENLPFSTVASLNGFHMFFKSLGIQL
ChFy    MKSNEPREGGIKSTDSLIKFSLHCLTVN-GIPLFSKNIG----HSSTVPFPVIGSLNGVQMFAKSKGVES
TaFy    ------------------MAYVIALTSGGGVPLFTRTIG-DYEAVCTLPFPTVGSLNGVHMFTETHGAKL
OcFuz   ------------------MSYLVAIAAESGLPLFVRAAK----GSKPLPFSSIGSLNGVYTFAEGRGTKL
OlFuz   ------------------MSYLVAIAAESGLPLFVRADE----GSKSLPFSTIGSLNGVYTFAEGRGARF
OspFuz  ---------------------------------------------------XIG---GVGAWTPRGGG--
ScFuz   ------------------MAYLAVLRSDTGVPLFSRTKG----ELSQLPFPLIGTLNGVHSFASQHATTL
AqFuz   -----------------------------------------------LSFPVVGSLNGVHWFTENAKASL
EmFuz   ------------------MAYLAAITAETGVPLFVRTTG----NLPPLSFPVVGSLNGVHLFASNLKTSL
MlFy    ----------------MSINSVFGILHQSGVPFLNRQFG-----SKTLPFSTIGSLGGLFTFANSCEGTE
SrFy    -----------------------------------------MFAVMRLSRAHRQLFYGIYKYVQRNEHEL

                80        90       100       110       120       130       140
        ....|....|....|....|....|....|....|....|....|....|....|....|....|....|
DrFuz   SSCETDRGSRVV---------WGVFQDSLMLIAVSGSGGSSISQLQLRRLLENVWNCMVLVLGQDELSS-
MmFuz   NSARTED-TTVV---------WKNFHDSITLIVLSSEEGT--SELRLERMLHMVFGAMVLIVGLEELTN-
DmFy    EATTTSKQQPIQNQAPADAEEWTYIWRDRESITLIVCGCGVGSEQLLQTLADLVFGAIGMFITRAAEMAH
ChFy    GLTRTET-GVVF---------WKEYESVKFILLVFENKCN---ERFLETLIENLYKSLIMSIGETELTK-
TaFy    LSTRT-QNAKIV---------WQVFNDSITIIIVTVDDGAS--DVHLKTLLQFIYNAMVLFLGENELKS-
OcFuz   ESTIA-GSRQIA---------WKTFHDSVTLILIEEKGNRT-RFQERHDFLELIFHSLVLLVGVAPLT--
OlFuz   ESTIASGSRQIV---------WRTYRDSITFVLIDEKRMRR-R-ANVDELLATIFDALVLLVGVPSLIGR
OspFuz  ----------------------------------------------------------------------
ScFuz   ASALS-EDAKIV---------WKSYHDKVTLILVAGEEGST-D-IHFKNMLDYMYNGLVSIIGLDTLVE-
AqFuz   LSCTS-KNVRIV---------WKNFHDSLSLLMTAQLILTA-T-TYRHSFLMLWYILLVLLIGLPDLLR-
EmFuz   LSCTN-DGVKVV---------WKCFYQSITLILVMTDDSPV-E-THLLHLLDMVFNALVLLVGLQELES-
MlFy    LSQFSKGSLKVR---------YKLYNDLVLLVHVTTDQMIT--DSYVSHALDHVQLFIVSEFGWDNLIS-
SrFy    TSTVT-EGARIT---------WRSFEPGIFFIFVHPNEHVE--DTTVDSFVQLLFNSLTFLLSKESLSA-

               150       160       170       180       190       200       210
        ....|....|....|....|....|....|....|....|....|....|....|....|....|....|
DrFuz   -----IRNVERLKRELRSCYRLIDMLLERVNDEEGFMGDLTQCTDCLLPSHSGLLLQEAVDSFAQATESE
MmFuz   -----IRNVERLKKELRASYCLIDSFLG--NSE--LIGDLTQCVDCVIPPE-GSAMQETLSGFAEATGTA
DmFy    -----ATLLDRLKKDAKKYVPIVDAILEAACAG-TQLLGYTDCLLAAENAQ----LLQCLNEFSGHCGSL
ChFy    -----SNNVERLKKKMKLCSPLLDAMLG--PSK--ITATLTQTTETLHISN-SSVLINYLTSFTEDLDSE
TaFy    -----LRNVERLKKELKSIYFLIDKLLEE-SLLIGHITGCLDTTAFTDIDVLQVHSYDCLEAFVGWCNTE
OcFuz   ----KAAKDDRLKRDLRCCFSLLDGLFSPPRNLFGSATGLVDVTWTSEASA----LQEYLDAFAERLDTN
OlFuz   DGGGAPTRDDRLKRQLRTCFPLVDSLWKPPRLLIGSLTGIVDVTWTTEAAT----FQDHVDEFAARLDTN
OspFuz  -----AARDDRLKRQLRACFPLLDSLLWPPRNLIGSLXXXXXXXXXTEAAG----IQDHVNSFAERLDTN
ScFuz   -----VRSTERLKRETRLSFGLIDTLLFG-TDLLGPLTHSVDVIATTETP----ILESLLNAFSEAAETQ
AqFuz   -----IEDPENLKRDLRCCYPLID-LLLGNQFTMSPLTQTVEVLVDHEHKS--LMVSXXXXXLSEAIESN
EmFuz   -----VQNVERLKKDLKCCYPLIDSLLLQGTHLMYPLTQSVDVLAVAVEDF--STLSQCMDAFSEAVHSG
MlFy    -----CQNADKLKSSLSSTLNIMSAALFEEFACISLHLLAYPVFIFNRGPK---LWLSKLEKFLDNVETA
SrFy    -------DATSLRLSLRACFGFIDHLLQEHNNLALQLGCMEVALSGSPAEL-----QEVLNTFCEHAESN

               220       230       240       250       260       270       280
        ....|....|....|....|....|....|....|....|....|....|....|....|....|....|
DrFuz   FGCLLLNGRVARATEKWWSRLTPQEVVVLSVLVRSLADAS--SCDYPVFLPKGSPTVAIRLLSFQLLPG-
MmFuz   FVSLLVSGRVVAATEGWW-RLGMPEAVLLPWLVGSLPPQA--ARDYPVYLPHGSPTVPHRLLTLTLLRG-
DmFy    FCCLVVGHRIAVATEGWW-DLDTRDRELLLFLLNSSSTMQ---HDVPVYLPVKSPHIAYRFVSIPVASN-
ChFy    FGCITVDGRVVVATEKFW-QLQTIETSLLSYVVYNLRNAT--ASDTPVYLPNGSPVVPHRLITVTLIDH-
TaFy    FGCVLINGKLAASTTRWW-NLPSTDSILLSLLISAMVHSAS--KDIAIYLPATFPDVPHRLMTFQILDG-
OcFuz   YACLLTDGKVCVATESWW-QLDDRELVLLSFLVHSLSSSPPTSADIPVFLPIASPEVAHRLLVVHVMG--
OlFuz   YACLCVDGKVCVATASWW-QLDRRELVLLLFLVRTLTGSS-----VPVFLPVESPDVAHRLVVYRLAD--
OspFuz  YACLLVGDKVCVATASWX-XXXXXXXXXXXXXXXXXXXXX-----XXXXXXXXXXXXAHQLVVFRVVDDD
ScFuz   FACAFVRGRVVAATAGWW-DLSPFDSVMLSLLCTHHPKST--SQDLPVYLPKASPNTPHRLLSLKVVED-
AqFuz   YSCLIVNHQIIHATESWW-QLDVIDSNLLTLLPSSLPPTQ--CTDVPVYLPVASNTSPHRLLVFRLYDS-
EmFuz   YGCVSVNGKIIVATESWW-KLSSLEIGLLAHLIASLPPSR--CQDIPVYLPRASNKVAHRMLVFSLLEG-
```

```
MlFy    YGCVFHEQNVHVVSGEWQKFPFVECLNLLLTCSKIKLDAEKVLKKKLVYLPVSSPNNQFLLVCCNYRP--
SrFy    YGALLVGGRCVAATEPFW-ELSSTDTCLVCAYAAYLPASD--AFDGPVFLPTLSPDVPHRFVRLRLLHN-
               290       300       310       320       330       340       350
        ....|....|....|....|....|....|....|....|....|....|....|....|....|....|
DrFuz   --VHVCVLCGPKPSLHNAS-ELISRFWSTLVENLRT---------------------CLEQAKRSSLPPS
MmFuz   --LELCLLCGPRPPLGQLDPQLMERWWQPLLEPLRA---------------------CLPLGPR-ALPEG
DmFy    --SALCVLCGAENASFRELHAHAMSAYRNEGNLLAA----------------------AERCVPRSLPEH
ChFy    --VQIAIVCGPEPSLHEIQNKLIVHYWSPLVPYLRE---------------------CLR-----SLPRS
TaFy    --VEVCVLCGSTPSHNEVLQNVCNSIKDRSITYGTYCCRIFAANFRFILEKLYRYFKKYACTKSSSTFHN
OcFuz   GAA-VCVLCDLATSLEHVERDVVPQCWTS-ALDLLK---------------------SSSYMLARGLPIG
OlFuz   GAALLCALCDLNASLDHVERDVVSQCWTSSALDVLKRL-----------------TASPSSPKCRGLPSE
OspFuz  DGALLCALCDLD----------------------------------------------------------
ScFuz   --FTVALLCEAVPTLEDVQEQILPSIWTGHLDTVHK----------------------CIQTFPRNVPAQ
AqFuz   --VSLLVLCDTEPSLDVAQKIVV-QIWLPLRDKIIS----------------------LPLLVPRAFPLS
EmFuz   --VHVCVLCDAEPSLRRAQDEVG-VYWSGLTERLVR----------------------CARNTS-CVPAG
MlFy    --FRVFALCPPTIQDSEILSAFNRVFPQKDSSTLVK----------------------------EDMFDP
SrFy    --VYVVLLCGARPALHTLVQQLVPSIWKGILPLLQD---------------------TARLEETGLPPSS

               360       370       380       390       400       410       420
        ....|....|....|....|....|....|....|....|....|....|....|....|....|....|
DrFuz   VSLRWDVQALLLINCDSRRALTVCPRVRVGAPAEDTPPLISTARRLELLRLFYVVFAVT-----------
MmFuz   FPLHSDILGLLLLHLELRRCLFTME------PSKDKEP--SPEQRRRLLRNFYTLVAT------------
DmFy    LEIDGNVLAIILINRHTRKSLFTRNLNQSASVKRIIVG------DLQRLDILKKFFD-------------
ChFy    F---------------------------------------------------------------------
TaFy    SRFWNTWVGLSPENSKKVKFCKIIIFCYTLKLMLWVLFNDAFRFILLNQNSRKCLATFVRALSNFTGIDD
OcFuz   VRLDPGVNAYVFLSQTPSMCLSSFGLGDDKSSRLGRESQQEKLVDFYWMVVGEMFPS-------------
OlFuz   ISLDPGIMAYIYVTRCPPMCMSDFNLDSSSSSSSGR-TWQEEILDFYWTVAGEAFPS-------------
OspFuz  ----------------------------------------------------------------------
ScFuz   VSLENSLLAFILVNDETHRCLSSYDLGPAQPPDAPTN-----ADFLSPGQRRLVLTS-------------
AqFuz   VTMDSSVIGFCFVNLLTRRSLLSVLS-------------------YETQERNPALLS-------------
EmFuz   VELSSSLLAFCLVNLKSRRCVSSLKPSTFSGDFSNNTLSAGFQGELSTEERHCLVEIYKTVADNMLPSQ
MlFy    IKFDPSILALTAVKNSESIVYVSDNFASEEHKMRKTLSYTLTRGLISGDVVDEHGTLG------------
SrFy    STVHESVLGVLAVDPVYHRCLVTTPSPDVQLQHTRCPTR-----RILRAWYKQILDAR------------

               430       440       450       460       470       480       490
        ....|....|....|....|....|....|....|....|....|....|....|....|....|....|
DrFuz   ---------------RYFTSQESTASDFAQGFT--HVPVQCYLVTDECKCFGLQSPQH---QLFLLMDPS
MmFuz   ---------------THFPPEPGPAEKQEDTVYPAQMPRACYLVLGPGMGWQLVAVQLGLRLLLLLLSPH
DmFy    --------------------QTMDAVHQFETQSSSNKTTLVDQYSCSDYHKCYAHMDELGNVIFLLFVAA
ChFy    ----------------------------------------------------------------------
TaFy    SAAIGKYNLIRLFSAGHNEQKHIILEDILIPLTLVYYFTYLAIQLQQNYSVGILWSLFINLSIRYFPLIN
OcFuz   --LAAASGKVTEQDKVGGGGDTGDTGDSGEVKNWLAHSCLETYICTEQYKCYALYC--EQKQLYLLLSAK
OlFuz   --LTTTT--------------MGDDGDEERHGRWLTHPCSETYVCTEHYKCYALYESHERKQLYLLLSAD
OspFuz  ----------------------------------------------------------------------
ScFuz   -------FYKSTVGSMFPHALQDDNGADTDLRNIIPHQPVETYLCAEEYKCYALHFNFHQLFTLFTP--S
AqFuz   ---------KGTIYIVP------------IIRN--------------------VGIDCSKISVL------
EmFuz   VKDGLRQETAGTDSVQPRPPEEVSPVNGRLLRASVPHRIAESYICTKKYKAYTLHQDGYNLFVLLDP--N
MlFy    ---------------------------NVDSFFFVNKNMNCHLISNQDILISALFKPNISQILSTALIAD
SrFy    -----------------------------HQLDTDTQQLHETYSCHETYSTYAILRPTCHVLVVLAP--S

               500       510       520
        ....|....|....|....|....|....|....|...
DrFuz   VPTFALRTVATQTLSAITSATGF---------------
MmFuz   TPTHGLRSLATRTLQALTPLL-----------------
DmFy    VPSHAMRFLAQRIHANILQEKSVCW-------------
ChFy    --------------------------------------
TaFy    AIQQVWNYLVTSLCNNLFCVIELVMVVAYFGFICYIVM
OcFuz   VPTYALRDVSHRTLKLLARSMDIVPGTSK---------
OlFuz   VPTYALRDVTYRTFKVFGQSADVVANTGK---------
OspFuz  --------------------------------------
ScFuz   TPTYSMRSLSYSTLDSLTTDQFISV-------------
AqFuz   --------------------------------------
EmFuz   TPTFALRKATMDVYKTLTGNTEVGEAMGGALVN-----
MlFy    IVDYFLERKKK---------------------------
SrFy    LPTYALKAVAQETAATLSQLGLG---------------
```

Abbreviations. Vertebrate: Dr, *Danio rerio* and Mm, *Mus musculus.* Hexapoda: Dm, *Drosophila melanogaster.* Cnidaria: Ch, *Clytia hemisphaerica.* Placozoa: Ta, *Trichoplax adhaerens*. Porifera: Aq, *Amphimedon queenslandica*; Em, *Ephydatia muelleri*; *Oc, Oscarella carmela*; *Ol, Oscarella lobularis*; *Osp, Oscarella sp.*; Sc, *Sycon ciliatum*. Ctenophora: Ml, *Mnemiopsis leidyi*. Choanoflagellata: Sr, *Salpingoeca rosetta*.

Figure S2.3: Inturned alignment. Threshold for shading has been fixed at 80% for both identity (black) and similarity (grey). Red squares correspond to the three C-terminal motifs described in the present study.

```
                     10        20        30        40        50        60        70
            ....|....|....|....|....|....|....|....|....|....|....|....|....|....|
MmIntu      ----------------------------------------------------------------------
DrIntu      ----------------------------------------------------------------------
DmIn        ----------------------------------------------------------------------
SpIn        ----------------------------------------------------------------------
LcIn        ----------------------------------------------------------------------
SmIn        ----------------------------------------------------------------------
ChIn        --------------------------------------------------MSVGTGNRYLWRENSQSYDG
TaIn        ----------------------------------------------------------------------
OspIntu     ----------------------------------------------------------------------
OcIntu      ----------------------------------------------------------------------
OlIntu      ----------------------------------------------------------------------
ScIntu      MVVMAAVAASSEATNPEQDDVDDQESYNPQWASEVDDRGNVFFIEPSTPGSGVVSGSALRAGGLYKTPVG
EmIntu      -----------------------------MAGTNSSRNWKQGKLEIFSSRPNVSIQDELPPQGVLLKPAV
AqIntu      ----------------------------------------------------------------------
AvIntu      ----------------------------XXXXXXXXXXXXXXXXXXXXXXXXXXXXXXXXXXXXXXXXXXXXX
OmIntu      ----------------------------MAQEAHKPRLPSQSKIKSKFVPLKATHHKSFDALSNDPSITS
SrInL       ----------------------------------------------------------------------
```

```
                     80        90       100       110       120       130       140
            ....|....|....|....|....|....|....|....|....|....|....|....|....|....|
MmIntu      ------MAGLARGDSRGRPPELPGDLSSQEEEEEEGDSDAGASSLGSYSSASSDTDVEPEWLDSVQKN-G
DrIntu      ------------------------MFSTCSDGRPFQSFSTNLEDFDSIRSVLLYSDLEPEWLDDVQKN-G
DmIn        -----------------------------MRKSPASLPSEAFSSSNSSLSNSYSDGSDLANWEEYVSTDG
SpIn        -----MDGSRRKANVRVKYSGESVPKPKMPTPEIVVDHEPEDDTASDAEYSDDSDALEPAWSSSVHKDSG
LcIn        -----MMSSLEEGGGDG-LYETPRTWDSYTEPGQQGSGSQSAASSDQEDESCYSDDLEPEWLDDIQKN-G
SmIn        ----------------------------------------------------------------------
ChIn        SYYSDNTSLQSQEHYYDGSYNSENNFFYSGERRHSNFGDMIERSDSYCSTCPSEDYHTDSEDEDAAQKWP
TaIn        ----------------------------------------------------------------------
OspIntu     ----------------------------------------------------------------------
OcIntu      ---------------------MSVRKLQLSGLSSSDSALFDPKSDEPESAFPNRASLDFEHLDSEPDIGG
OlIntu      ------------------------MLSASSGLSSSDSALFDPQGDKPDPSFANRASLNFDRFD-DRDILG
ScIntu      NGGPRRLRRSPLSPSQSLSYGLSLSAIGGAQRGDTVAAAAGHASGSSSGAAAAGTSAKTGQAADGEKRSK
EmIntu      KYNIDDGAWSDKVDPSTGSLLYVDPVLRSEHRLTSSGVFDSTSSSNSSLSSIASPEMSPSRQPSCQPSAS
AqIntu      ---------------------MAEKVQGRKRSKESFKRERSYSYSNDSQSPDSSAGVPDIEITQVEDKSH
AvIntu      XXXXXXXXXXXXXXXXXXXXXXXXXXXXXXXXXXXXXXXXXXXXXXXXXXXXXXNISSDAWVNEIDPSTG
OmIntu      QKPPQSPFPSIPSFIKQKLPGRHSRTGSTSSVQSLDMLQTSGSNFSFETPPPISQFSDDIWVTQIHPVTG
SrInL       ----------------------------------------------------------------------
```

```
                    150       160       170       180       190       200       210
            ....|....|....|....|....|....|....|....|....|....|....|....|....|....|
MmIntu      ELFYLELSE---DEEESLLPETQTANHV--NHVRFSDKEVIIE---EDDSRERK----KSEPKLRRFTKI
DrIntu      QLFYLELSD---GEEEALLAQVSANHSAATNHVRFSEKEAEII---TDNAKRQANSSNKSEAKLKKLTKI
DmIn        SLFYMEYVRCEKTGVSAERRVEFMRRSLRLGKKSSRRQP-------HSNNQNQSQNQNQSHGPSRLDSQT
SpIn        ELFYLEAGKPVLSPRETNKGSTTPKDTTPGKTNRKGIAQLQKV---LKVSKSRDSTPQSSPNGSRFFSRT
LcIn        ELFYLELSE---GEEEALLQASSAHISAG-NHVRFSDKEAEVI---PDNRLKER----KCEPRLKRFAHI
SmIn        ----------------------------------------------------------------------
ChIn        WISHIQDNGDLIYVHTHHQHKHSHPRNHNHNHSNSNHHKPRTKSFLHKDNKEKGLQGKLKNVIKKSRSFT
TaIn        -------------XMATEEKEVKIGRHRSFHSHRKPSNLDVTPPSSARRSHSFKIKWLKNVIPIKFRPFA
OspIntu     ---------------------------------------AAAA---AAPSKTGAEKKGRKSRWKRLLPSA
OcIntu      LSDAFNPQGNVFFVEESRRVLSPIRSS-PD---SHEKPRIRPA---SASSAAGTEKKGKKHKWKKLLPSA
OlIntu      GADTIHPQGGVFFIEESRRALSPIRSASPDGVPTSTAPRIRPS---SAAAASTSEKKERKSRWKRLLPSA
ScIntu      LKAMWKNLTGQRGRGGAAHSTSGPGVDNEFHTSSSKSMPAAQSHQANHGNSLNTASGMHATGGDPVQQHG
EmIntu      SLSNRRTHSPSVSEYLSSSPASEHHYSPSMAAKASKKDERRSVERNSPTKSPGGGGGKSATLESRNLDSI
AqIntu      KLGSPRRSVPVLSVIPSPSRAPEAPPRGGVKEKKKNQTRVRSA---SPKRYGYSLDRKKAAYSQRLLQSY
AvIntu      NLFYVELQPVLRRKKHHDSSLSLSSAISDISISGATAPKSNATPGTSPSLSRKRKLKLSKLFPNRVLGRK
OmIntu      NIFYIDIQPIFRRKKHYQSTISLNSVISDISAASAPIKPIETPG-TSPSLSSKRKLKLSQLFPHRTLGRS
SrInL       --------------------MCDAVLMRRPNEKRAKREKDATYCYKAAVAAAYNASEAARLAASLLGEDE
```

```
                     220       230       240       250       260       270       280
             ....|....|....|....|....|....|....|....|....|....|....|....|....|....|
MmIntu       LKSKSLLPRRHHKKSSSNN--------GPVSILKHQ---SSQKTGVTVQQ-RYKDVTVYINPRKLTAIKA
DrIntu       LRRKRRPSQRKAEGKDSSQ--------RPASILKNQ---AGQRPGVVVQQQRLKDVCVYLNPKRLSSVSS
DmIn         EPQEFRFSQFKTAAPDPKK-----------------LDLVITAADRFRFGRRSTAVESILGFRVLPFPDQ
SpIn         PPGSNHKLRRSSPGSQSNG-----------GTPVKE---VILKVGTSPPGSPKDTLSIRLQGWLGIVPGK
LcIn         LKKKNLSRKPSKRNSPCNANDAGSQPSGPTSILKHQ---PSQKTGVIVQQ-RYKDVYVYVNPKKLTHSGV
SmIn         ----------------------------------------------------------------------
ChIn         KKDVIDKNIDISHFKNSEK--------KKQQNPLTDTTTEIQTITTDGVHLLCKNVLIHLNDKVQESFAN
TaIn         HLDDDSPIQTTDDPISPAG---------AADVVFRKITLQLDTPTVITDLEDSLGIKVQFYGELSETIQE
OspIntu      LMRRAGKSKAKESXXXXXX---------XXXXXXXX---XXXXXXXXXXXXSLESLGIVVGRGD------
OcIntu       LMRRAGKG--AKKATMDGR---------TGKTQIVS---VKSPDGRKLRVDSLQSLGIVTGRSK-GEEGR
OlIntu       LMRRAGKSRKTKSDVVDER---------VGKTQTIV---IRVPTGR--RIDSLESLGIVAGRSVRGEE--
ScIntu       PQRKPGLAGLSEHPTVGSSLEHHAHEVQPTMTTSKLKTVVKVTVKMPSDMSAKTDGDMSLRLLGITAECR
EmIntu       SLCSDSLPDTRRSLTTYFRSVPEGEQFGSRGLPLDENAACITRVKIDLAQYHIGTDGNPCSLELLGILPD
AqIntu       SSGLDTPNLIRDNDDTCNN---------SLTDGREE---TLTKVHINLSQYKLGLPHVPCSLEALGIIPT
AvIntu       KQTSTPNPVRLSTFSLDEKLNLKTSTESSKAPSVIYTSNKLITNXXXXXXXXXXXXXXXXXXXXXXXXXX
OmIntu       KKKNQQSPMHKPARNSTISLDEGKLLKTSLPESPKPFPRSKSVTNSIYLNLKLPQSDRDSNPITKLDSLT
SrInL        ADTSDAVDVAASAGGGDGS--------------------RAQREGSESRHGDGGKQGAGG----------

                     290       300       310       320       330       340       350
             ....|....|....|....|....|....|....|....|....|....|....|....|....|....|
MmIntu       R--EQVKLLEVLVGIIHQTKRSWKRSAKQADG---------ERLVVHGLLPGGSAMKSGQVL-----VGD
DrIntu       SSADRGGLLEALLGVVHRPGGN---TGKRGGK-----------LIIHGLIPHSPASKCAEIL-----IGD
DmIn         P----------------------------------------ECLMVDGFVHDVSALQHGIKR------GD
SpIn         G------ATDLLSGSVMGNGREHKNGGPVRVD---------DRLMVRGLLPQGAAMKSAEIE-----IGD
LcIn         G--EGTNLLEALIGILHQSSWNGKRAGKDGGQDTASKGTCEERLLVHGLLPGSSASKCGQIM-----IGD
SmIn         ----------------------------------------------------------------------
ChIn         LENVIGVISGLDLDPVLDDFSDNILMNGNSTRYN------SNCIVVQGIIPNSPAGES-EIN-----IGD
TaIn         GK--------------------------------------LDILQVAGIDSNSPAYRCGQLS-----IGD
OspIntu      ----------------------------------------ERRVRVRRIVPGSVAEIGNQIR-----MGD
OcIntu       HG--------------------------------------KGRLVVKGLVPDSLADKTREIR-----NGD
OlIntu       ----------------------------------------KGRFQVKRLVSDGLAEKRNEIRVGEKRNGD
ScIntu       PRVPSSSGPNGAASGTSPNLPRHTAAARAAVEN-TLLEDTSDVLVITAIAPGSPAASAAASGTARLQTGD
EmIntu       RRSMTRGHAIFASSRLSLRSHRRHLPG-------------HDRVVIKDVTKGSPAAASQQLH-----KYD
AqIntu       R------YHSSGKGSFLSLTRRSLKGASSKKP---------VPVIIRAVLPSSPAALTEELS-----PGD
AvIntu       XXXXXXXXXXXXXXXXXXXXXXXXXXXXXXXXXX------XXXXXXXXXXXXXXXXXXXXXX-----XXX
OmIntu       KFGIIIQQPRIKSSKSFDDLLDGSNAVSREKHTS------LSPVIIQSIIQDSPASIDGSLR-----SMD
SrInL        ---------------------------------------------VDATATAAAAAERGSDS-----RDD

                     360       370       380       390       400       410       420
             ....|....|....|....|....|....|....|....|....|....|....|....|....|....|
MmIntu       VLVAVNDVDVTSENIERVLSCIPGPMQVKLTFEN--AYAVKR-ETAQPQKKKAQSSTQDLVKLLCGSEAD
DrIntu       ALVAVDDVEVTSENIERVLSCIPGPMQVRLTLET-----VCPAGVSPESKVSASPQVSQLVRLLWGEDTI
DmIn         WFRSLNGIEVYASNVDELLKQFVEPTQVCLGFQSCGAASAVSPTDPGYNRNVREEQVCKVENFPMFTEKF
SpIn         VISAINGVPVSYSSAEAVLNSMEPVKQVRLSVER-----APSWNEHNPSPVVCRPPNGTLVKMLNNIAEA
LcIn         VLVAVNDVDVSLENIERVLSCIPGPMQVKLTLEMPVCDPVRSGPSLQHSSKQTLTPVSNLVRLLWGEDNL
SmIn         ----------------------------------------------------------------------
ChIn         IILAINGIKVNLQSVNSIVEKINGTQEIVLTIER--------LATVVEPEKPSELCSIVSNTLDEGAKED
TaIn         KLISINGHEVTTSNLDELLKSLGEVSEIELVVTR----VAFLKDDFKIQPDKITRIDPDLIVSKGDTRQN
OspIntu      CIFALNGMPVTEDTVDRVLRDCANENEXXXXXXX----XXXXXXXXXXXXXHSSTATPALIHLFSGTPSA
OcIntu       HIAAVNGIPVSKETVNQVLRDSIGAKEISLTVYR----MVASQSSGVKLRRHISTATPALIQLFNGIPSA
OlIntu       YILALNGTSVAKDIIDQVLRDNSDSNEISLTVYR----MGMSRTR-VRLRRHASTATPALIHLFNGTPSA
ScIntu       EILQINGVNVSTRNISTILAAVAFYKEIAMRVQRDVSLKASKMSSTSTSSQHLLSLMRGTGAGGGGARRQ
EmIntu       IILAVNGHCVDMSNVDSVLSGVIDEVQLMVQKHS------------LQLPLSPDPGSPSLSKLVAGSKVT
AqIntu       EIARVNGDDVKMRSIEESLSKLTNEVIFDVYKCT-----------PSSPPSHTLSPPPTYFSDTTSPTPP
AvIntu       XXXXXXXXXXXXXXXXXXXXXXXXXXXXXXXXXXXXXXXXXXXXXXXXXXXXXXXXXXXXXXXXXXXXXX
OmIntu       KIVSIDNNNVTQDNIHIILDKLKNKPQLTLEIQKEQYSNFTYTNPDTEDVSGESGIAESFARLVAGKETF
SrInL        TVVTDDTATAEHGPVNREHNQAGTAAAADVNSRTG------SSSNPHQEANKAEEQEEKEEQEEQEEKEK
```

```
                     430       440       450       460       470       480       490
                ....|....|....|....|....|....|....|....|....|....|....|....|....|....|
MmIntu          AVQHSTL-------------------------SIPHISMYLTLQLQS--------EAAREEQEILYHYPV
DrIntu          ELQMSIA-------------------------DVPHIAMFLSLRLDS--------ETQQDEQEIVYQYPQ
DmIn            EQMLITEGN----------------------FGIPGSSVDIRMPFAMLLLPP-ECYQHEQQQDSLYYFPE
SpIn            ASKQQDQQQQGKEEGNPHTS---------PGDDVPCAIMYLTLVRDS--------DSD-DAQEVLYKYPS
LcIn            ELQLGLS-------------------------SSPHIVLYLTLKLDS--------ESSKEEQEILYHYPV
SmIn            ----------------------------------------------------------------------
ChIn            VFHAYHD---------------------------TCMVSLLTLNSNE----------DDVDGDILFWYPD
TaIn            DEEVDLY-------------------------QVPHVALCLTMGSSA---------DEDGDKDIVYKFSL
OspIntu         NWKMTXX-------------------------XXXXXXXXXXXXXXXXX-----XXXXXXXXXXXXXXXX
OcIntu          NWKMTMS-------------------------GIPHCVMYLKLVS---S-----EAED--DKDIVYSYPS
OlIntu          SWKMTMS-------------------------GIPHCVMYLKLTGDDDS-----EGNDGAEKDVLYSYPG
ScIntu          YSPYNARTKDASASGNNDQFGSRAPNIAVKPKVALHGLMYLTLGSGSGAGSSSKNDEALHMEDILYHFPQ
EmIntu          LRSLSHSLR-----------------------SLPHLLLYLTLDTRE---------DDHPDMDILYRFPP
AqIntu          SSSFSPP---------------------------PPLILFLTLDTK---------EDDPPDKDILFCYPS
AvIntu          XXXXXXXXX-----------------------XXXXXXXXXXXXXXXXX-----XXXXXXXXXXXXXXXX
OmIntu          ENIAKTSEK----------------------DIGTYLCLYMTINDKN-Q-----VSKDQGMDDLVFYYPH
SrInL           GNKAKAE-----------------------------------------------DEEEQEEEENEDEQPA

                     500       510       520       530       540       550       560
                ....|....|....|....|....|....|....|....|....|....|....|....|....|....|
MmIntu          S--------------------------------EAS----QKLKSVRGIFLTLCDMLESVTG--TQVTSS
DrIntu          S--------------------------------EAS----AQLKAVRGIFLTLCDMLENVTG--GQIISS
DmIn            IP-------------------------------------DNFLFKARGSFLTLHAVLSELHT---QPLSS
SpIn            S--------------------------------SAS----TKFKDINGLFITLGDMLMNITG--SKITCT
LcIn            S--------------------------------ESS----QKLKAVRGIFLTLCDMLENVTG--GQVIR-
SmIn            ------------------------------------------------MFMTIGQMIPDVDLS-TPLLSC
ChIn            S------------------------------------EKSKILRNTRGIFLTLSDMLTDVMA--EHIQSS
TaIn            S-----------------------------------DTQAKVLTALRGLFLTMSDVLLQVTL--DNVFAT
OspIntu         X--------------------------------XXXXXXXXXXXXXXXXXXXXXXXXXXXXX-XXXXXXX
OcIntu          E--------------------------------DSHWRLSKLILGIRGAFLTLSDMMKEITG-SYAE-SS
OlIntu          E--------------------------------DSHWRLSKILLNVRGAFLTLGHMMEEVAG-SHVERSS
ScIntu          CTAATAAKKNGDDEEGNDGKRDSAGGRHGQTGQSAQWSPIDHLVSIRGVFLTLSDVLQQVTG--TSTSSS
EmIntu          E--------------------------------GAER-----LLQTRGLFLTLSDIVGSIGH--NALSCS
AqIntu          R--------------------------------GCHH-FLDQIVSLRGAFLTLSNLIPTISSSLSYTTSS
AvIntu          X--------------------------------XXXXXXXXXXXXXXXXXXXXXXXXXXXXXXXXXXXXX
OmIntu          QPIPKNIDS-----------------------KDPAASSLHKLVAVRGMFYTLADVVQSISG-LAGDRAT
SrInL           S----------------------------------------ALGVLSGALITMIDSFPEHVT-RDMPIGN

                     570       580       590       600       610       620       630
                ....|....|....|....|....|....|....|....|....|....|....|....|....|....|
MmIntu          SLHLNGKQIHVAYLKESDKLLLIGLPAE--EVPLPQLRNMIEDVAQTLKFMYGSLD-SAFCQVENAPRLD
DrIntu          SLWLQQQLVHVGYWKEESNLLVIAVPAS--RVPLLYLQTVIEGVVRTLKVMYGSLD-RGFSDVENAPRLD
DmIn            RLLVDQVQYHVNYRQLNGFLVLFAYAGG--LCCAAECSLRSDELIGYIRFSLPGLVLESFNQEREPGNSS
SpIn            SLEVGDELVHVGYHKWGSELLVLAFPAC--FASKHTVQCMVQQLARLLNFMFLSLN-RAFSERRNHQRLD
LcIn            -------------------------------VPLPQLRNMMADVVQILKVMYNSLD-SAFYQVENVSRLD
SmIn            NITVQNEVVHVGFGKEENELLVLALPSA--CATVREVQYFTANIVKLLKFQYSTLE-NAFMTEEYRNRIE
ChIn            VLYTNKETINVAYKKCGGNVLVVAAPDH--KCDLNTLISCTDDFSKLLTIFNGDLN--RAFTDENMESTR
TaIn            SLLIDKMITNVVYYQFEQNLFILAVPDQ--RCGLFLLNRTIRNIARLLKVLYGSLH-SAFFSTQNEQNLN
OspIntu         XXXVNDRLIHVGCRQCGKCLLILALPAD--TIHLHSLVQYLDDICKLLAFLFGSVARXXXXXXXXXXXLD
OcIntu          TVSVGEKLVHVGYRQCGKCLFVLALPAD--VVHIHSLSEFLDDVCRLLSFLYGSVS-NAFESGQNTSRLD
OlIntu          TVFVNDQLVHVGYRHYGRCLLLLALPAR--TVHLHSLVEYLDDVCKLFAFLYGSVPRSAFESSGNFKRLD
ScIntu          FLVVDGKPIHVGYLQLGENLMVLMLPAT--SAPLFYLDCVMEDIDRLLTITFGSIH-SAFTKKRHKERLD
EmIntu          SLILDGRLVHLGYIQSGKELVLLALPAT--LASLYHIQLLIKELQQTLELMFGSLA-SAFKKRENHPSLD
AqIntu          VLVVDGELFYISGHLVGKELLLIAQTG----VSNYQVELVLDSLVECLELVYGDLM-SAFKS--DSSTLS
AvIntu          XXXXXXXXXXXXXXXXXXXXXXXXXXXX--XXXXXXXXXXXXXXXXXXXXXXXXXXXXXXXXXXXXXXXX
OmIntu          LETTNGEIVHAAFKLLPNKEIVVIIAPENVVEEKTTLCNHLDTFIDALTFQFGNVS-NAFMQQSNLEELD
SrInL           TQVLSGTLVHVAYVYLEDDYVLAVACDAA-ACPFVHLQTKLVEFVRVLKSLYCDFQ-ALLVSARHHRLLT
```

```
                    640       650       660       670       680       690       700
           ....|....|....|....|....|....|....|....|....|....|....|....|....|....|
MmIntu     HFFSLFFERALRPGKLHLSGSPSAQQYAAASAVLLDNLPGVRWLVLPQELKVELDTALSDLEAADFEELS
DrIntu     HFFCLFFQQLIQPSRLIHSSRTP----DLYGSLFLDGLPAVRWLTLPPDIKVEVDTVLSDFESSDFGDMS
DmIn       RLKLFLRHFCEIQRTRLVARCHG-------HIRFEELLGQSRSLPLPKEAQLRIFDALSEMEAMDYRNWN
SpIn       HLFALLFHQVLVDRSQLEP------RKKHKEDIFLQMLPGVRWLNLPDQDKLNVDCCLSELEAADFADLC
LcIn       HFFNLFFQRAIQPARLSSSSSPSSQQYDSFSAAFLDNLPGVRWLTLPQDIKVEIDTAMSDLEAADFADLS
SmIn       IYCADFFEYFLGSVYSGQK--------GVSCSHTLEILPSVQWVPLPIDLKTEIDDILSELESSDFESSC
ChIn       HETSLFLDLLLQRKP-------------------VVPFGTVRQLQLPTKQMIEISNVLSDVEAADYGECV
TaIn       KIFKYALYRLLTPATDLASMR---FLKAFYPTYLAHLFEAAPSLFLPNVDQALVSGALTKLECGSYFQMP
OspIntu    HSFSLIFERILNPGESGFX------------SRTNDTCHGLLKLYVQPDLLVKVSSVXXXXXXXXXXXXX
OcIntu     HTFSLIFERFVSAGESGFS------------TRTSDTVHGIPKLYIQPDLLVKVSSVLNDLESDDYSDTL
OlIntu     HTFSLIFERLLSPGESGFS------------TRMNDTSHGLLKLYVQPDLLVKASSVLSDLESDDHSETV
ScIntu     QLFSLIFERLLSRDRDGYH-------------HVTDALHGIHRLPIPFEVQSKISSVLSELEADDHSILD
EmIntu     HIFSMFFQKTLAPLPCWGT-------------SLLKGVACAPVLSIPLDHRLKLSAALTELDSVDRSSLP
AqIntu     GVLSSILSFSSSPSP------------------NSFLFPALQQLPLSIKTAAQISSHLNQIDAASYSPPL
AvIntu     XXXXXXXXXXXXXXXXXXX----------XXXXXXXXXXXXXXXXXXXXXXXXXXXXXXXXXXXXXXXXX
OmIntu     HLCCLLFSNIYRTVLFDQS------------DVFISNLMASQTLRINSEINTEINSLLSELNASDLSDQP
SrInL      LHLTRFFAHLLASYQHRLG-------------------HHCSIHNHPSSLRLSIAGVRRRPRVIQKALHL
```

```
                    710       720       730       740       750       760       770
           ....|....|....|....|....|....|....|....|....|....|....|....|....|....|
MmIntu     EDYYDMR-RLYTILGSSLFYKGYMVCSHLPKDDVIEIAAYCRQHCLLPLA---AKQRIGQLIIWREVFPR
DrIntu     EDFYGMR-RLYVILGSCLFYKGYLIANHLPKEDLLDVCLYCQHYCLLPLA---SEQRVAQLVVWREVFPQ
DmIn       DEPLTTH-REFFIYGSALYYDGFLVASLLPPEVRVSVEGFLRCRGIFELLGAAPGIKVREMYVWEEIVLP
SpIn       DRHFDYR-RPYVILGSCLLYKGYILANHLPVDDLMDVHLYVQYYSLSSLS---TQQAVGQLVIWREVFPT
LcIn       EDYYGMR-RLYMILGSSLFYKGYLIGNHLPKDDLLDIALYCQHYCLLTLA---AEQKIGQLVIWREVFPR
SmIn       HGFYANQ-RIYGILGSCIFYKGYLLSNHLPKSDLLDFHLFLHHHHVLKLT---RMQPVSKIIIWHEVFPS
ChIn       DDFFPHQ-RLYGTIGTCLFYKDNLVCSHLTDENLQDVVTFCKYHWLFMLA---KSEKMKQLVLWKEIFLT
TaIn       GQMTHTR-RAYASLGSCLFYKGYLVASRMRDEDFLDTRLLCFYYGLLDAS---KRHIGGNITLWHKMNLQ
OspIntu    XXXXXXX--XXXXXXXXXXXXXXXXXXXXXXXXXXXXGIYCRYHCILSAM---RKQKLGHIVIWTKIFDT
OcIntu     DVLMLRR--RYRIHSSCLFYKGYLIDSHMSSNDLADVGLYCRFHCILGAM---IKQKLGHVVVWTEVFEK
OlIntu     DVLVLRR--KYRIRSSCLFYKGYLVDSHMMDEDLSVVGIYCRYHCILSAM---RKQKLGHVVIWTKVFSG
ScIntu     EVLYMRR--PFLILGSCLFFKGYLIESHVPEEDTQDLALYCRYYCLLQLS---SRQRLGQLVIWHEIFLS
EmIntu     EVMCVPS--PYRVVGSCFFYQGTLVCSHLGNSDLQSVVRFCHAHRLLELT---AAESVNQIVIWRQVWLP
AqIntu     LSPPGSSPSLLSPLGSCLFYKGYLVSSHLTLQLLRAAHLVCVCFVTRQPL---AELTS----VWKQIYLK
AvIntu     XXXXXXXXXXXXXXXXXXXXXXXXXXXXXXXXXXXXXXXFCSFHGLLNLT---STQPAGQIVIWREIFPT
OmIntu     EISAMQR--PYHTLGSCLYYKGYIICSQLPASYQREVNLYCSYHGLLNLT---STQSVGQIVIWKQVFTS
SrInL      PSLETCDPRCMISSG-----RGNAVFHFLAAEPGTGLVLVSDPFAPLNEH-------SLQAKVLDQFYRT
```

```
                    780       790       800       810       820       830       840
           ....|....|....|....|....|....|....|....|....|....|....|....|....|....|
MmIntu     HHLQPPSDSDPE---AFQEPEGRYFLLVVGLRHYLLCVLLEAGGCASKATGNPG-PDCIYVDQVRATLHQ
DrIntu     RRETRNSTAHP----GYCQPHARHFLLIVGLRHFMQCVLLEAGGCASSAVGRPT-PDSVYVDQVKATLLQ
DmIn       SAT------------------GRYFLTICTKNHLSLAVILKIFDAPDMAPDAVVGPSLFYIEEIQETLDH
SpIn       RRVQPIEAPHP----GYTEPQGRWFLLIVGLKHSVLCVLLEAGGCASPCEGVVY-PDEFYVDQSRATLLH
LcIn       RHFQLGKDPGVE---GYREPEARYFLLIVGLRHFLLCVLLEAGGCTSKAIGNPG-PDCIYVDQVKATLLQ
SmIn       RHQEPSIVN--S---DFQETSGRYFMLIVAMNHCVLAVLLEAGGCAMKVEGFPP-PEPFYVKEVENTLII
ChIn       EKSGESPTTKN---IYETPENARWFILVVGQGHSLFCTLLETGGAASIPEGNPG-PHPYYVDTAWSILTY
TaIn       RLQGEKRS------YGVFTEDSYVYLLVVCVNSCVIATLLECIIPSDTISQSE--ADPIYVNSARDTIKY
OspIntu    KDE------------GASSE--QRYLLVVGMANALVCVLLEAGCAAEELSPN---ADPLYIDLAREVVLT
OcIntu     ETG------------ATNGEDYRRFLLIVGMANALVCVLLETGCGTEEPLPHPG-PDPLYVDLAREVILT
OlIntu     EEE------------AAG----QKYLLLVGMANALVCVLMETGWELEELPPHVG-LDPLYIDLAREVILT
ScIntu     RRGPLVSENQPED-PAYVEQTGRTFLLIVGFNHALLCVVLEAGGTTAKVTGHPG-PVPYYVDVAEEALST
EmIntu     STDTPSED---H---TYFLHRGNTYMLVFGVERVIVCMVLESR-HSGVMGGVAA-LDTTLVESANTLVAE
AqIntu     EE---------------SQSDANLFVLAIGMEHTLLCQVVSVG---RELNISSF-PDDRVKENCRKILSD
AvIntu     ISEASIRKNQTQKNLNFKSLGGKHFLLVVGIGQSICAQILLAGPMATRLLDKPX-XXXXXXXXXXXXXXX
OmIntu     NVEPPSTPKTHKN-ISFKCLGGKYFLLLVGLGQALCAQILLAGPMATRLLNLPP-PDPLLVDRIKEMLDE
SrInL      SLR-------------------VRALLHQSRASNTSSSLFTAPPPPPPSSSSPLSSAATPSTPSRRHSAM
```

```
                    850       860       870       880       890       900       910
                ....|....|....|....|....|....|....|....|....|....|....|....|....|
MmIntu          LEG-VDSR--IEEQLATSPGPCLSCADWFLAAP-REKADSLTT------SPILSRLQGPSK-TAASP---
DrIntu          LES-LDAG--IEERLSAPPTPCLSCADWFLPAGGRSQQDTIGS------SPILNRLTAAIKPPSPGG---
DmIn            LVQCGIESLAMFWSVSNKRPEVLDATASESRDQEKEPNNRLESFLKQKLTVLSPSVEEEAQLCSSLGGS-
SpIn            LET-LDVPSACEERLKVPPIPALSCADWLFPSPRRGSFDSSVPPRPHPDSPMLSKLHSQSQTPLKGKKPE
LcIn            LEG-VDSS--IEERLAAPPSPCLSCADWFLPSS-RDKLESFTS------SPIFSKMHGSTKSAVVSP---
SmIn            LHD-CGIASAVDNMLSICLPPVATPDLFFKDCASTWNPLSSIFTSTGTAYSSNSNINNVGNFSGHSG---
ChIn            LIENMDYETSSHSRLEGPSIPAVVHATEAINPKTSASPRAIELIKRKVPIQRRPHHDHLSISHSISN---
TaIn            LEC-DGFFAICDDRINNVSCAELASADTFTTSDSAVKRSSSLSASSRRSTVSTGNTPNHHRHPSGGSIN-
OspIntu         LNV-RGLLEALDMRLTSSRIPAVTHADELAWMS-------------------------------------
OcIntu          LNV-RGILEALDLRLSSSRIPSTTQADALVSAL-------------------------------------
OlIntu          LNV-RGLLEALDMRLTSSRIPAVTQADDLV----------------------------------------
ScIntu          LSS-RGLLQACDSRIQMAPAPAVITASQALSATSAMQPALLKFAQQHG---IKAVKEDGTDSDPGSG---
EmIntu          MRA-KGLLEGLERRCGGGCVGGYGVCTTTPFPSLVLADTAMAT-------MFSSRHHNMSHLVNNQG---
AqIntu          IHS-SGLLSDCERRLIG--LPALTSADVLLASN----------------------LTTAAAAGAGGE---
AvIntu          XXX-XXXXXXXXXXXXXXXXXXXXXXXXXXXXXXXXXXXYFELG--------------------------
OmIntu          LER-YGLFSDLEEKSFNTAHLPPITNPLVDSNKGNDRNTIFERN--------------------------
SrInL           FPGDHPLSMMEEHGLMFTVGTATWSSSSHDQQQ-------------------------------------

                    920       930       940       950       960       970       980
                ....|....|....|....|....|....|....|....|....|....|....|....|....|
MmIntu          ----TCRRTF--FSDYSFKARKPSPSRIGGGREP-TEGEE---------SAGLSPHATPDAVRKQ---RE
DrIntu          ----IGRSLFGEAGTVGIRGRRASPQRSQ--SDSGSEGHA---------------DGTPASVARRDS-LG
DmIn            ---------------------------------------------------SIHSLTPSEDESCRRRLTPIHG
SpIn            SPHMPHKRSSSPTPPVSFLSVSPNPSRKPTSTSSRTESDNESDSTSPHNSRTNSTQSSPSTTRRSDSRKE
LcIn            ----TSRKVL--FGDTSLRTRRPSPPRSSGGSDTGSEGHGEGG----YLNTGLSPHSTPDSIRRHGW-RE
SmIn            --------ALLEKGASSRKGLEGNLAERLDDPEVERQHLISFMGPADGESDVESNYSDDSNDNSQSQDKR
ChIn            ------------------------------------------------------------------ESDL
TaIn            --------FLLRSPNLSLNVSRHKNPPNSPVVDRKVLSANDDGSVSAATLRNGNPEKLSSSHGNDLLEAD
OspIntu         ----------------------------------------------------------------------
OcIntu          ----------------------------------------------------------------------
OlIntu          ----------------------------------------------------------------------
ScIntu          ---------------------------------------------------------------------H
EmIntu          --------------------------------------------------------------------SE
AqIntu          ----------------------------------------------------------------------
AvIntu          ---------------------------------------------------------------------L
OmIntu          ---------------------------------------------------------------------L
SrInL           ----------------------------------------------------------------------

                    990      1000      1010      1020      1030      1040      1050
                ....|....|....|....|....|....|....|....|....|....|....|....|....|
MmIntu          SEGS---DDNVALLKLARKKSTLPNPFHLGTSKKELSEKE-LEVYD--IMKLTSGPENTLFHYVALETVQ
DrIntu          SGGSDGSLGSAGFLKMPRLKH--PNPFYLGSLRKSLSERETEEMQN--VLQVTAGVENTLFHYVLMESVQ
DmIn            TAEDSDSGSDWENFAVQHPLHYGLNLGAESHSQSQMTESMWKEINNVVPVKISAGWKNSVLHYVYIDIAN
SpIn            SDASIGSVGSSEIYRAVRRGRLIPDPYSMGVMQRAREETSSPDYFT--ATKLTSGRDNTMFHYVHLDPAA
LcIn            SQGSGGSEGSGGSGSINKKKHNLPNLFHLGNTKKNLSDKESEELYN--ATKLTSGSESTLFHYISLDTVQ
SmIn            SRSYSTSTGSTNGSQGSSEYVNVQLPKHGTASDTNIGLLGCENLFSENAVSIVAGCENTLFHYLHMDPVE
ChIn            GMRERSQSRTSNLSGNSSNRHYSVHEMAPHFKQLSMASTSEVSTTAALATKLPTGSHNTLFYFTVLSNRN
TaIn            SSQARERASSYGLHRASPIVSRKNAISANTAYSYLDTTVEQSRSEGFSIRNVTSGKINVLFHYIGINSKT
OspIntu         -------------SFSSASARHSIAAINSDEMSRHKGRLVGGKLGKLGTSSLTKCS-GLLFHYVNVNTVS
OcIntu          -------------SLLTTSR-HSLSVGDSEDLARHKKKLA----AKGEISSLTKCNNGLLFHYVNVDSVN
OlIntu          --------------LMSSGS-SSLSVPDSDDLGRHKSRLVNRKFGKHGTSSLTKCA-GLVFHYVNVDTVS
ScIntu          AGSPGLSRAIFMQKAALPPGPASSGFRKSIKRHGGPKAATVSKPLASLAHSLTAGDGNILFHYINVDAVA
EmIntu          SEDESSPLGSRRTASVSLFKFQKKDLSLPSLSHVGGHSKASIVGVPRPADMLNAGAETIVLHYIDLHAER
AqIntu          KGGGGGGGKGHHRMTRSSTGSIYSLSTIVSGSLSDSDDDSSSRLTPNKSSSVLTAGMENHLLYYVSLESGR
AvIntu          RGSRGSLRTSGDSTDSEGSNLTKTQSRHSLNLLDNLDKPHGKAATFDSIAFLTSSPTNLLFHYLVFDDFS
OmIntu          RSSRSSLRTSGESTDSDGSTATKTQSRISLPLLDPQPQPIGKQIPYDSISFLTSSTINLLFHYLVFDELS
SrInL           ---------------RQQQQHQSRGGVSCNPAVSAFQRRRTKRASSRHASAEHGGGSPFPLSYWVVGRLL
```

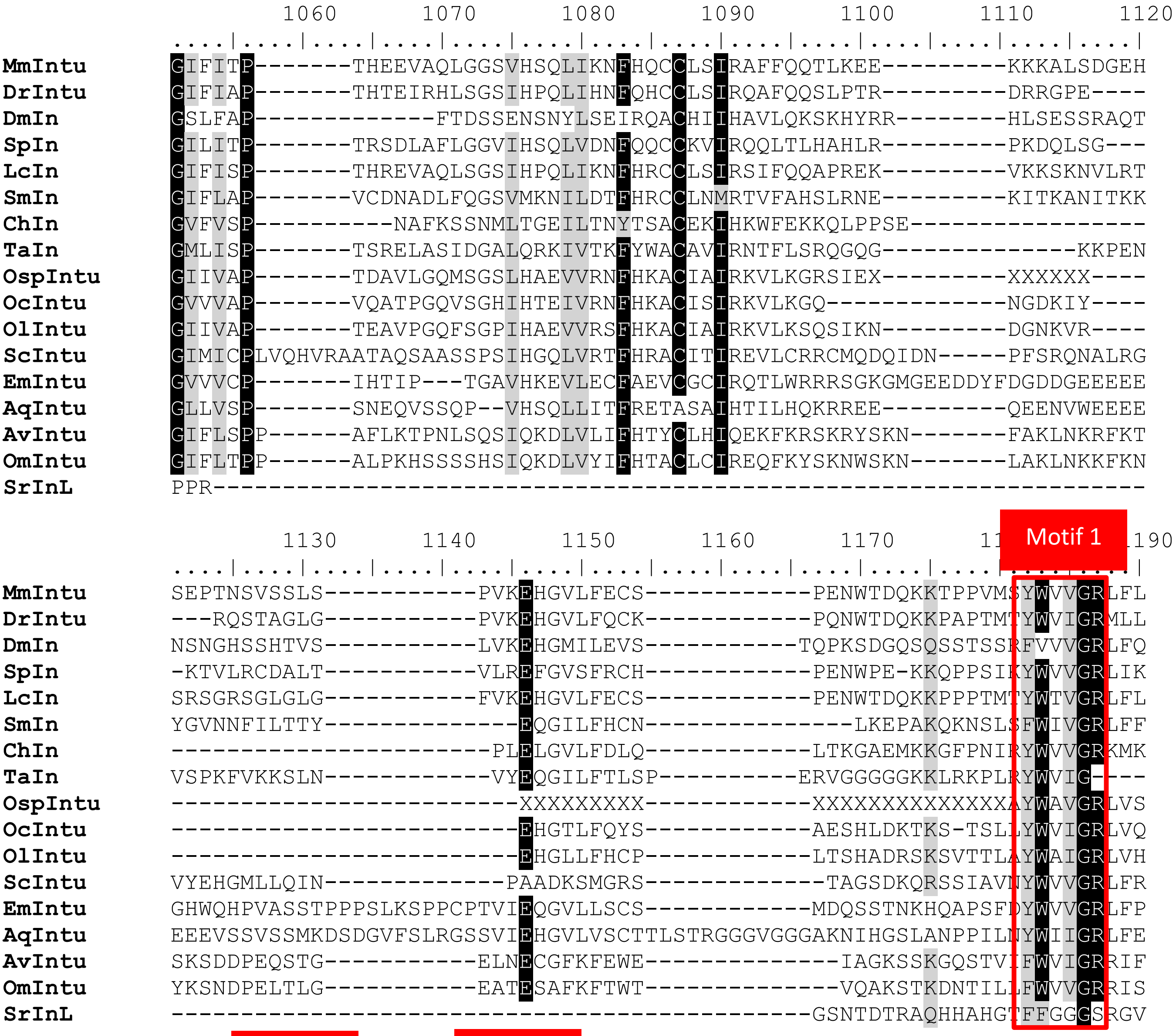

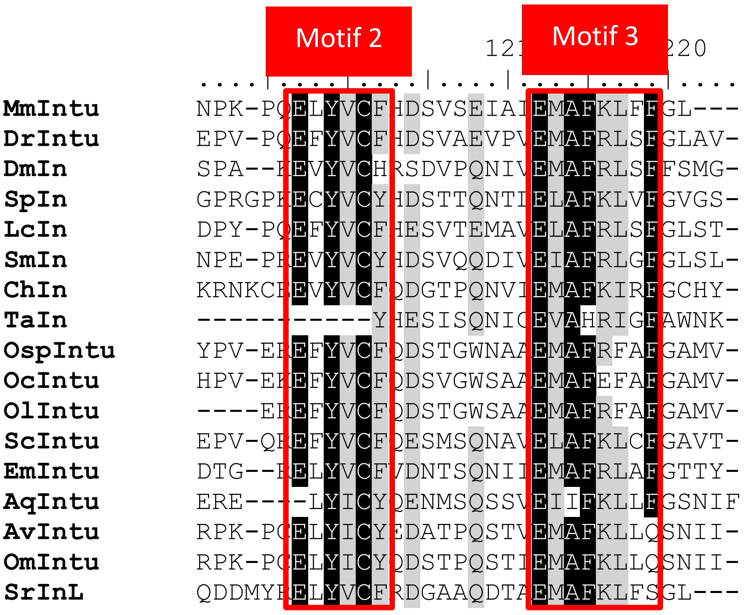

Abbreviations. Vertebrate: Dr, *Danio rerio;* Lc, Latimeria chalumnae and Mm, *Mus musculus.* Hexapoda: Dm, *Drosophila melanogaster.* Ambulacraria. Sp, *Strongylocentrotus purpuratus*. Cnidaria: Ch, *Clytia hemisphaerica;* Nv, *Nematostella vectensis.* Placozoa: Ta, *Trichoplax adhaerens.* Porifera: Aq, *Amphimedon queenslandica*; Av, *Aphrocalistes vastus*; Em, *Ephydatia muelleri*; *Oc, Oscarella carmela*; *Ol, Oscarella lobularis*; *Om, Oopsacas minuta*; *Osp, Oscarella sp.*; Sc, *Sycon ciliatum*. Ctenophora: Ml, *Mnemiopsis leidyi*. Choanoflagellata: Sr, *Salpingoeca rosetta*.

Figure S2.4: Inversin alignment. Threshold for shading has been fixed at 80% for both identity (black) and similarity (grey). The black square indicate the C-terminal sequence shared by Ambulacraria, Mollusca and poriferan Invs 1 while the red squares correspond to the four AAX9Y/F motifs.

```
                    10        20        30        40        50        60        70
           ....|....|....|....|....|....|....|....|....|....|....|....|....|....|
MmInvs     ----------------------------------------------------------------------
DrInvs     ----------------------------------------------------------------------
HpInvsL    ----------------------------------MNFNLCIYFIFFNLQMKRHNAEVAPLQVASGVKSIP
SkInvs     -------------------------------------------------MSKSQREQRKMQTRQASRTIP
AcInvs     -----------------------------------------------------------MAAAAAVASPP
ChDgo      -------------------------------------------------------------------MTV
OcInvs1    ---------------------------------------------MDPRGGLQPPYATSGISFTPSTQPV
OlInvs1    ---------------------------------------------MDPRGGLQPSYATSGISFEPSSQPA
OspInvs1   ---------------------------------------------MDQRG-LQPPYATSGITFEPSSQPT
ScInvs1    ---------------------------------------------------MAAAFTASDVSRAGRMDGS
AqInvs1    -------------------------------------------------------------------MRV
EmInvs1    -------------------------------------------------------------------MED
AvInvs1    --------------------------------------------------------------------MQ
OmInvs1    --------------------------------------------------------------------MS
OcInvs2    -------------------------------MNRESNQSRIASLRPDQFAYRAVDSWPVPGPGLGKRRRM
OlInvs2    ----------------------------------------------------------------------
OspInvs2   ----------------------------------------------------------------------
ScInvs2    ----MASFGKRQKSKSFAVSPAG------HSHNAKPLNRRATSTSLELTLQAQARMRPKTSTSVRLATRN
AqInvs2    MDQQQAQIWSQLSPYQQFQLLQQQ-----SLLSQGAYYDDLAPEDTEWFTEPAPPDPPDTAVTEYMYERL
EmInvs2    MAYQRSVYSTDPTPSVWYNAAEGNSISGDQSFDYKASISRQTSTLPKVVEELSPAISAQFIGGGSSLNRQ
AvInvs2    -----------------------------------------------MSHYVSSRMSARPGGGGQNTQRQ
OmInvs2    -----------------------------------------------MTHYVSSRMSARPGGKSQ--KRN
SrInvs     -------------------------------------------MSLDLAVPEARFDRAPSATSTRLGAAD

                    80        90       100       110       120       130       140
           ....|....|....|....|....|....|....|....|....|....|....|....|....|....|
MmInvs     --------------MNISE---------------------------DVLSTGSSLASQVHAAAVNGDKGA
DrInvs     -----------------MA---------------------------MLLPQNP---SQVHAAAVNGDKNT
HpInvsL    DVKGQVPSHGRGGGVLDSK---------------------------TSLQREASPTTQIHAAAVNGQKAA
SkInvs     SFE--VPG---AEGNIGSD---------------------------TSLKRDVAPTSQIHAAAVNGDKSI
AcInvs     HCTQMPPGSETPRGPFSAR---------------------------SEGQRLEG-VTQVHAAAVNGDRQT
ChDgo      LESQNTTNISNGFHDGFG----------------------------DETFGNQSQSSPLHVHAFNGNKKL
OcInvs1    LPGMADFYVSSRELLPSAS---------------------------FAQPPDTVHGSPLHGVAVNGERDE
OlInvs1    LPGVAEFYVSSRELLPSSS---------------------------FVQPPDTVHASPLHGVAVNGERDE
OspInvs1   LPGVATFYVSSRELLPSSS---------------------------FVHPPDTAHASALHGVAVNGERDE
ScInvs1    LVMQGMTTTTSDTGIRLG-----------------------------MQDVGDAAG-PLHAAAINGIKAQ
AqInvs1    TMATASDYTKWRIRQMLRD---------------------------GEAGEEAIITTELNSAAYSGDKDL
EmInvs1    FNQEAAGYE----------------------------------------GQEWDLQLPIIVSAYNGDIST
AvInvs1    FSSAILSHVSITD-----------------------------------YLSPQIVNSPLHNAVLSDDVIL
OmInvs1    FTATNSSHRSISN-----------------------------------LFSLEQNTSPLHDAVISQDLPS
OcInvs2    TSRTSLAPEKLYNVEEFLE-------------------------------PGKGPLVGLHKATACGDDAA
OlInvs2    -------XEKAYNMDEFLE-------------------------------PDKGKLEGLHKAAAYGDDVA
OspInvs2   ----------------------------------------------------------------------
ScInvs2    ADAALQCWVLPTSEGGAGGTVANLLERAASTISLTGVAEEGADGDFLRRVLKSGQLTGLHKACAQGNINA
AqInvs2    AHTALDSWKLYQNEKEMKR----------INSRLSQSGRGLGETSDLKGLIDTKKLSGLYKAAFKGDAAG
EmInvs2    VDSAVLSWRIGKLPGDVG----------TSSLGISGRNVDLELRELLKQRG-NHPLEGLHKAAAHGDRTA
AvInvs2    IIKSVDTWRAASS-----------------------------------LLQDSSHVMGVYKAALHGDANL
OmInvs2    IILSVETWKPAQT-----------------------------------VTQDPSQVLGLHKAAFHGDANL
SrInvs     SLVNDIAGMTDTDLFKSIS---------------------------DVTYQEQHQVSFMHLTAMTNNLND
```

```
                    150       160       170       180       190       200       210
           ....|....|....|....|....|....|....|....|....|....|....|....|....|....|
MmInvs     LQRLIVGN--------SALRDKEDRFGRTPLMYCVLADRVDCADALLKAGADVNKTDHSRRTALHLAAQK
DrInvs     LHKLIT-E--------SALRDSEDQFGRTPLMYCVLADRLDCAEVLLKAGAGINKTDHSQRTALHLAAQK
HpInvsL    LQKLLKTY--------PKEVDQADQFGRTPLMFAVLADRLDCAEVLIKKGANVDAKDNGGRTALHWATYK
SkInvs     LQKLLSAN--------PHQIDGQDQFGRTPLMFAVLADRLECTEILLKAGANVDAKDSGGRTAIHWAAHK
AcInvs     LSRLLAGD--------YGDLEVGDQFGRSPLMYCVLADRLECAEILLKAGAQVNLQDRGGRTALHWAAHK
ChDgo      LQDALQSK--------EYGVDDIDSTGRTPLIYSVLVEQIDCFNLLLKHGADIDKPDTDGRTCLHWAAYQ
OcInvs1    LLDLIASA--------AYDLDCGDQFGRTPLVYSVLGDRLDCAEILLKAGAQVNRADMDGRTALHWAAYQ
OlInvs1    LLNLISSN--------AYDLDCGDQFGRTPLVYSVLGDRLDCAEILLKAGAQVNQADMDGRTALHWAAYQ
OspInvs1   LLNLIASN--------AYDLDSGDQFGRTPLVYCVLGDRLDCAEILIKAGAQVNQADMDGRTALHWAAYQ
ScInvs1    LAGLLQSG--------AYDIESQNQHGQTPLIFSVLGDRLECAELLVRYGAEIHVTDKEGRTPLHWAAYT
AqInvs1    LLALLKE----------HDIDECDEQGRTPLMYASMVNQYDIVDLLLKRKASSTIQDTLGQTPLHLAASN
EmInvs1    LQQLLKS----------SPVDEPDSSGRTALMFAAMADRADCIDLLLKAGARISAQDINGQCSLHWATLN
AvInvs1    LKELISSK--------LYDVNISDDNGRTPLVYSIFIGSFVCFDILLNNGADLYSLDVQNMNPLHWACQI
OmInvs1    LRQLLTSN--------SYDIDVLDECGRTPLIYSIFVGSFVCFELLLQSGSNIYILDFDSKSPLHWACQL
OcInvs2    VMNIMKIEGERHHHIMYTGLDELDVHGRSPLMYAVLSESLACMEVLLEFGALREQIDGNGRSALHYAAFY
OlInvs2    IMNIMKIEGQRHHHIMYTGLDQLDAYGRSPLMYAVLSESLACMEVLLEFGALREQIDVNGRSALHYAAFY
OspInvs2   -----------XRHIMYTGLDELDAYGRSPLMYAVLSESLTCMEVLLEFGALREQVDVSGRSALHYAAFY
ScInvs2    VVSIVKIDSERQGQLGYAGLSERDVFGRTPLMYAAVADSKPCLDMLLTCGAKREVRDPNGHTAVHYAAYF
AqInvs2    IMNIVKRDGRNAP------LNELDKNGCTPLMYAALSDSDSAIEMLLNLSVRREQVDAVGRTAVHYAAFF
EmInvs2    IIQMLKQIGPGD------HVDILDDNGCTPLMYAAIADSWQAAEMLLNFSARREMTDTEGRTAVHYAAFF
AvInvs2    LMHIVTAEGLHL-------INQQDQLGIPPMIYATLSDNPACIERIQELGGRKEVSDMYGRTSVHYAAYF
OmInvs2    FMHIVSVEGLHM-------INQQDQFGIPPLIYATLSDNPSCIERLQELGAKKEVKDLYGRTSVHYAAFF
SrInvs     LQGLINQHP--------DYVNAVDSLDRTPLYFAILANQPKTCKLLIDAGALVDVQDADGRTPGHWATFH

                    220       230       240       250       260       270       280
           ....|....|....|....|....|....|....|....|....|....|....|....|....|....|
MmInvs     GNYRFMKLLLTRRANWMQKDLEEMTPLHLSTRHRSPKCLALLLKFMAP--GEVDTQDKNKQTALHWSAYY
DrInvs     GNVRFMKLLLSRHADWRLKDLEEMTPLHLASRHSSSKPLSLLLKHMAP--GEVDTQDRNKQTALHWSAFY
HpInvsL    GVFRLCKLLVSKGAYWREKDNESQTCLHFATRHKDTRCLALIMKQLLP--GEVDEQDNSKRTALHWSASY
SkInvs     GHFKCLKLLISKGANCKEKDSEGQTALHLSTRHKNTKCLALLMKQLHVDLGEVDEQDSAKRTALHWSASY
AcInvs     GNLRFMKLLLSRGADCREKDNEGQTGLHLCTRHKLPKCMALLLRQLTP--GEIDDQDKNKRTALHWAASY
ChDgo      GNHKLVKLVLSKCKNRTSRDKEGQTPLHLAISHDNLRVMQIILKHLN--EHEVDATDNRGMTALCWSAHY
OcInvs1    DNYRCLRMLLSKGANWKAKDNEGRTALHMATGHTSCKCLKELLKKMKHGSREVNDSDNEKMTALHWSAYN
OlInvs1    DNYRCLRMLLMKGANWKAKDNEGRTALHMATGHTSCKCLRELLKKMKQGSREVNDSDNEKMTALHWSAYN
OspInvs1   DNYRCLRMLIAKGANWKAKDNEGRTALHMATGHINCKCLRELLKKMKQGSREVNDSDNEKMTALHWSAYN
ScInvs1    NNAKIAKFLLGKGASIREKDLDGRSPLHLATSHKSGKCLKELLRRSK--AADVNEGDNEKMTPMHWAAFH
AqInvs1    NAFECLKLLARSATELNIKDMDGRTPLHLSSAKSNLRCLDFLIRKLR--SSELNEGDNEKMTALHWCAYN
EmInvs1    GHYKSLKLMLAQSLDICIKDHDGRTPLHLSTTQLSSKCLALLVRKLK--SHELNDADNEKMTALHWSAYN
AvInvs1    GLPKIVKALIANNCDLYQKDAQGRIPLHYTTLHHDPKVIHILIKRMNR--SSIDEGDSLGMSALHWAAYH
OmInvs1    GRLRIVKYLISKGCDIYQKDSDCRTALHHATVQHLPKLLSMILKNYDQ--SLIDQPDSLGMSALHWSAYY
OcInvs2    GRHRALQYLLDKATNVMARDKFGRTALHWAAMISNPKCLSVLIQYALAHRLGVDWLDDEHVTPLHVAAQF
OlInvs2    GRSRALQYLLDKAANVTARDAFGRTALHWAAMINNPKCLSLLIQYATGHHLGLDWTDQEQVIPLHVAAQF
OspInvs2   GRCRALQYLLDKAANVMARDVFGRTVLHWAAMISHPKCLSLLVQYSAFHSLGLDWVDKEQVTPLHVAAQF
ScInvs2    GQHRALKFLIEAACDWAPHDIYGRPPLHISISSDSVKCLQLLLKHCSVVGGDVDLVDKEAMTALHVAAHC
AqInvs2    GKAKSLKFLIESGSDWTPRDAYGRTPLHWATASKNTKCLLTLLNYASIVNGNINPQDNEGMTPVHCAAQF
EmInvs2    GKYNTLKFLISSASAWAPRDSYGRTPLHWSCANGSTKCLQVLLNHAKVVYGDINPKDNEGMTPLHCAALF
AvInvs2    SKMKALKYLLQSASDWSSIDLYGRTPLHWACASKSVKSLRFLLEYANSVYGQTEYVDFERTTPVFVAVQF
OmInvs2    NKLKSLKYLLQTASDWSCIDAFGRTPIHWSCANNSVKALKQLLNYANSVLGQTEYIDFEGTTPVFVAVQY
SrInvs     GCPECLDVLLAAGATPDVRDKDGRTMLHWATAQKQIKCLQHLLKQQD---LDLDTRDSEEMTPLHWACFH
```

```
                     290       300       310       320       330       340       350
             ....|....|....|....|....|....|....|....|....|....|....|....|....|....|
MmInvs       NNPEHAKLLIKHDSNIGIPDVEGKIPLHWAANH-KDPSAVHTVRCILDAAPTESLLNWQDYEGRTPLHFA
DrInvs       NHPEHVKLLIKHDSNIGIPDSEGKIPLHWAAHN-KHPNATRTVRCILEAAPTESLLNWQDYEGRTPLHFA
HpInvsL      GNEEAVRMLVKHSSNIGIPDTDGKTPLHWAANAGDSPTAINTVQHILETEP--SVVNWQDYEGRTALHLA
SkInvs       GNEEAVRMLIKQDSNIGIPDTEGKTPLHWAATAGQDSSAVNTVKLLLESAP--SVINWQDYEGRTALHLT
AcInvs       GNLEHVKMLIKQGSNIGIPDTEGKTPLHWAASS-RDPDAVSCVLLILETTP--SVINWQDYEGRTALHLA
ChDgo        ERIEHVELLLRYGSDVHVLDNERRGILHWTSQN----KDPVLIKRLLEKGC--KGLGFPDQKGRTVLHMA
OcInvs1      NNPEAIKLLIKAGADIVLPDVEGKTALHWTAGN----DSGTSVKAILELSP--AVINLQDNEGRAVLHLA
OlInvs1      NNPEAIRFLIKAGADIVLPDVEGKTALHWTAGN----NDGASVRAILEISP--AVINLQDNEGRAALHLA
OspInvs1     NNPEAVKFLIKAGADILLPDVEGKTALHWTAGN----NDGASVKAILELAP--AVINLQDNEGRTALHLA
ScInvs1      GNIEAVKLLFRNEADLTVSDVEGKTPLHWTAAN----EDPTCCKALLEHFP--AIINIADTEGRTTLHLA
AqInvs1      NNSEGVRKLLQSGADIVLTDIDGKTGLHWTASN----EDDSTIKVLLDKAP--TAINLKDKDERTVLHLS
EmInvs1      GAAENVRLLLKAGADIVVTDTDGKTALHWTANN----PDDSTVKVILELAP--AAINLRDNEGRTALHLA
AvInvs1      NNHSACRFLIRNHSNPGNTDNEGRTPLHLCSGN----KNIMSIKVLVELAG-STLLTLQDFQGCTLLHHI
OmInvs1      NNSEACRMLIRNRSIPSSVDNEGRTPIHLCAGN----DDCSSIKVFLEFAG-QTILNLQDSQGCTLLHHI
OcInvs2      NREKHVVMLIKAGARCEIIDSTGQTALHYCLGN----ANVDCIQTLCSAYP--SVLNAKDGSGATLLHWA
OlInvs2      NRERNVALLMKAGARCDCVDMTGQTALHYCLGN----TNVDCLNTICSGHP--AILNVKDGSGATLLHWA
OspInvs2     SRDKHVAMLMKAGACCDCIDVTGQTALHYCLAN----MNADCLNTICSGHP--AILNAKDGSGATLLHWA
ScInvs2      GKAQHLSTLLKAGASPKAVDMEGKTAMHWAVTN----TDATCITTLHQAYP--AIVNMRDIYSETVLHVA
AqInvs2      GSAKHITLMIEANCDFSLVDIERKTVLHWAVEN----KDMSSLHAIVNHSP--YLLNRQDINGQTLLHIS
EmInvs2      GRPKHISLLNEAECSLVAVDIEGKTALHWTANN----SDPSCVLALMEAYP--PLLNRRDVYGCTVLHLM
AvInvs2      CNTQHIEILAKEGHNFLQEDIEGKTAFHWTVDN----PNTNCVESLLKFQP--ELLNKQDGEGKTLLHIA
OmInvs2      SHAQHIELLTREGHNLLQEDIQGKTAFHWTVDN----PSTDCIDALLSSQP--DLLNEQDGEGKTLLHIA
SrInvs       NHAKHVQLLLNAKADITAVDMEGKTALHWTSGN----KDAAVVKVLLASKP--ELANQGDNEGRTPLHLC

                     360       370       380       390       400       410       420
             ....|....|....|....|....|....|....|....|....|....|....|....|....|
MmInvs       VADGNLTVVD------VLTSYESCNITSYDNLFRTPLHWAALLGHAQIVHLLLERNKSGTIPSDSQGATP
DrInvs       VADGNEAVVE------VLTSYEGCSVTAYDNLFRTPLHWAALLGHAKIVHLLLERNKSGMIPSDSQGATP
HpInvsL      VANGNAAIVQRL--VDFQTPLVKCNISVLDNMFRTPLHWAAVLGHTHMVNMLLDKN-ANYSCSDSNGATP
SkInvs       VADGNEPIVG------ALTSLEKCNVTALDNMFRTPLHWAAVLGHTKIVQLLLNRK-ADYASTDSNGATP
AcInvs       VADGNESVVI------MLTSVENCNVSALDNVFRTPLHWAAVLGHSKIVAMLLKSG-ADSSSSDSNGATA
ChDgo        VGQGNQAIIE------YLLTISDIPVNQTDEIQRTPLHWAAVLGHTAIVELLLNSG-ADYSLADNNGVRP
OcInvs1      VAEGNEKTVG----ALTSMPGVKCDVISRDHMFRTPLHWAAVLGHTHIVGLLLERH-ADYASADGNGATA
OlInvs1      VAEGNENTVG----ALTSVPGVKCDVTSRDHMFRTPLHWAAVLGHTHIVGLLLERN-ADFSSTDGNGATA
OspInvs1     VAEGNENTVG----ALTSVPGVKCDVSSRDHMFRTSLHWAAVLGHTHIVGLLLERN-ADYASTDGNGATP
ScInvs1      VAESNYIIVD----TLTSMPNIKCKVSAADRMFRTALHWAAVLGHSDIAGLLLDRG-ADPSAADSTGATP
AqInvs1      VAAGNATVIM----TLLNSSSVRCDVMAVDSDFRTPLHWAAVLGLSQIVGILMENG-ADPTAVDATGATP
EmInvs1      VATGNVAVVE----ALISAQGVKCHLSAADLQFRTPLHWASVLGLPELVRVLMDKG-ADPRCADAVGATP
AvInvs1      VAFENFSLLSALTDSTNFTEIKALKLDSKDLEHRTALHWAFAFGYATIADILLTLG-ANFEIFDGNGYLP
OmInvs1      VAIQNVSLLSTITNPQNSSWLMNLNLDSKDLQHRTPLHWAFALGSSVIAGILISRG-ADWKVIDDNGYLP
OcInvs2      SAHGHAHIVA------VLVSFDGCDNESRDQWSCTPAHYTCLYGNANCLQILLARG-AHDYATDINGLTC
OlInvs2      SGHGQAHIVA------ALVSFDGCDIESRDQWSCTPAHYACLYGNANCLRILLAHD-AHDYATDINGLTC
OspInvs2     SAHGHAHIVA------ALVAYDGCDIESLDKCGCTPAHYACLYGNANCLRLLLAHN-ARDYATDVNGLTC
ScInvs2      SKLGYAHIVD------AVCSMENCNMDTKCRDDQSALHLATIHCQNSCVSTLLRHG-ALDACVDSFGLSP
AqInvs2      SKKGNGPLVE------RLLNIDGIEIDTRDKQDCTPLLLATMEGHSNVVDYLLDFH-ALDSCIDASGLTA
EmInvs2      AANGSLPLMD------TLLRIEGVEVNAQDSTQRTPLHYACLGGHAEVASMILQWG-GLDRMVDIEGATP
AvInvs2      CEYNLSHIAY------NLLINQGILIDLRDSNGRTPLHSTAISGSPNLTEALLSQK-ALDSVFDDANLTA
OmInvs2      CEYKLSHIVF------YLLHQDGLLIDARDLSSRSSLHYAAISGSAVLVEALLSRK-ALDSIFDMEGLTP
SrInvs       VGDASKAIAQ------AILKSAKTDPTLVDHTGRTALHWAVALANDTMIGVLAKHR-DSVRVQDEQGATP
```

```
                  430       440       450       460       470       480       490
              ....|....|....|....|....|....|....|....|....|....|....|....|....|....|
MmInvs        LHYAAQSNFAETVK-VFLQHPSVKDDS--DLEGRTSFMWAAGKGNDDVLRTMLSLK-SDIDINMSDKY-G
DrInvs        LHYGAQSNFADTVA-VFLKHHSVRDEP--DLEGRTAFMWAAGKGSNDVIKIMLDLK-KDIDINMTDKY-G
HpInvsL       LHYAAQNNHTETVE-VFLQREGITDEP--DLEGRSALMWAAGKGADGVIEVMMRYK---QDINATDKT-G
SkInvs        MHYAAQNNYAETVD-AFLSRENVTDEP--DLEGRTALMWAAGKGADDVIRTILKRN---SDINATDKT-G
AcInvs        LHYAAQNNFSDTVS-VFLSN-QCTDEA--DLEGRTAFMWAAGKGADDVIRVFLEQG---VDIHQEDKT-G
ChDgo         LHYAVQNNHRDVVA-TMIRTGRVTDEP--DKDQRTALMWAALKGHMNVLKVLLGGK--NVNINAVGIN-K
OcInvs1       LHYAAQNNFGETVA-TMLSFPHVRDVH--DNDGQTALMWAAGKGSYEVVRTMMER---KLDVHACDKQ-G
OlInvs1       LHYAAQNNFGETVA-TMLSFPQVKDLH--DNDGQTALMWAAGKGSYEVVRTMMER---KLDVHACDNQ-G
OspInvs1      LHYAAQNNFGETVA-TMLSFSQIKDLH--DNDGQTALMWAAGKGSYEVVRTMMER---KLDVHACDNQ-G
ScInvs1       LHYAAQNNFATTVA-TFLTFDHVQDTP--DDEGRTSLMWAAGQGSYDVIRTMLER---KMNVNAQDQT-G
AqInvs1       LRYQAMKDHEDTVE-VLLSFN-SDDIP--DNEGRTALMVAAEKGYYNIVKKMIER---KVEINAQDNQ-G
EmInvs1       LHYSAQKDHVDCVA-ALLSYNGVQDIS--DAEGRTALMWAAQKGNASCIRAMLEMG--TIDLHVRDQL-G
AvInvs1       LHYAVERNFVDCID-CFFSHKNVADLP--DKGNRTSLMLSCQIGHFESTRAILERN--IISIDYSTSVTG
OmInvs1       LHYAIERNFIDCID-CYFTFDFTDDLE--DNNGRTCLMLSCQLGHHESTRAILERR--ISGVNGFSDKNG
OcInvs2       LHYAVQENHLDCIH-LLVSLPLASNLP--DSEGHRPLTWAAALGHHNAIKSLLQNSNFIGTIDQEEEE-G
OlInvs2       LHYAVQENHLDCIH-LLVSSPVASNLP--DSEGHRPLTWAAALGNHNAIKSLLQNSIFVSAIDQEEEE-G
OspInvs2      LHYAVQENHLDCIH-LLTSSPVASNLP--DSEGHRPLTWAAALGNHNAIESLLQNAAFVGAVDHEEEE-G
ScInvs2       LHYAIMSGSVDVVR-LYAQLESLANLP--DSDGRRPLHWAVTEDNAEMLALLLEHPDMQADVNIEDKR-G
AqInvs2       LHYAVQINSVNCVK-LLVENEHATHIPSANEQGLLPLSLAAIFGFSDIIKALLVNPHIANDINRTDSN-R
EmInvs2       LHCAVMSGSASTVRSLLSLLPEPTYIP--DQSGNTPLTLAAQYDSAEVLNVLLSSDELRKHINQIDSN-G
AvInvs2       LHYAVLYKHVQCVN-MFTLQDNMSHLP--DNEFKTPLMYAACISEVPILNALLSRPEICAQINAQDKQDN
OmInvs2       LHYAVQQQHVECVR-VFLLQNSPSHLP--DHDFRTPLMYASVLDNIAIVNALLNHPEVRIHINAIDKQEN
SrInvs        VHYAAQLDSEPCTKALLKSMPKDMVDVE-DREQRTALFWAIAKDHLSTAELLLKAG---ANPNHVDST-G

                  500       510       520       530       540       550       560
              ....|....|....|....|....|....|....|....|....|....|....|....|....|
MmInvs        GTALHAAALSGHVSTVKLLLDNDAQVDATDVMKHTPLFRACEMGHRDVIQTLIKGGARVDLVD--QDGHS
DrInvs        GTALHAAALSGHVSTVRLLLEQGGMVDPLDVMKHTPLFRACEMGHRDVILTLIKGGARVDLVD--IDGHS
HpInvsL       ATALHAAAMSGHASTVEVLLQHGAAVNVVDQTKHTPLFRAAEMGHTEVMKTLAKGGAQVKVVD--QEGRS
SkInvs        GTGKHKNAISSNNKSFSLLLXHGAHVNACDMMKHTPLFRACEMGHLEVVKTLMDGGAKVNIAD--QDGRT
AcInvs        DTALHAAALSGHSTTVQLLLNSGARLEGMDQAKHTPLFRACEMGQMDVVQTLIEYRARVDILD--QDGRS
ChDgo         QTALHMSCQTGNLECVQLLIQHKADVNMMDQQQHTPLFYACASGHGQIVTKLLQQDDQRNLHQCDLEGRT
OcInvs1       GTALHGAAYAGHANIARLLLQHGAPLNALDARNHTPLFRSCEMGHTEVAVTLIQGGAQVDIVD--VDGRT
OlInvs1       GTALHAAAYAGHANIARVLLQHGAPVNTLDSRNHTPLFRACEMGHTEGVVTLIQGGAQVDIVD--VDGRT
OspInvs1      GTALHAASYAGHANIARVLIQHGSPVNSLDSRNHTPIFRACEMGHTEVVVTLIQGGAQVDIVD--VDGRT
ScInvs1       GTALHAASYAGHTNCVRILLQHGAKVNSADQLGHTALFRACETGQLDVVLTLLQAGARVNLAD--VDGRW
AqInvs1       ATALHLAAYSGHSDICQLFIQHNADINATDINGHSPLFRACECGHNPVVLTLIQHKALVDLID--NEGRT
EmInvs1       ATALHAAALNGSAECVQLLLQHGADVNAQDSNSLTPLLRACERGNTEAVVMLLNGGASVELRD--TIERT
AvInvs1       LTALHYAALGGNLLCLKLLLKYGAEINAKDIHGRTPISYASERGYSESVKFLYSSGGAIDISS--NDGRI
OmInvs1       MSALHFASIGGNINCLKLLLQYGALVDMQDNHLRTSLSYACEYGYAFIVEYLINSGGLIDLPS--IDGRL
OcInvs2       RTALHIASINGYSLCVRVLLESGAHIDAQDIYGSTAFHLAATQPHVKCMQELISHGADVLLRD--GNGQT
OlInvs2       RTALHVASLNGHALCVKALLENGSHIDALDAYGCSSLHLAASQSHINCIQELLSYGADVFACD--ANGQS
OspInvs2      RTALHVASINGHALCVKTLLENGAHIDVTDAYGCSSLHLAASQSHDDCIQELIAYGADVFARD--TNGQS
ScInvs2       MTAAHLAVENGSTDCLSELLNANATVNKRSSEVESPVFVACETGKAQCLELIMRSGCNPNLGS--FEGLP
AqInvs2       LTAMHYACEGGFFDCVVTLLQHGSDPNQLNNENITPLDIVCENGFYICIGPLINGGAEAERIG--NDGCS
EmInvs2       RTALYFACYAGAADCVTSLISNGADPNICDAFQQTPLFPACESGHGACIHALAKGGCDINTRD--GDGHA
AvInvs2       TCLLHVVITTQSVDAMRVLLNAGADPNVLNSQGYNALHICCQMNYVESAILLIDSRASVLIPDR-NELLS
OmInvs2       SSLLNTLLATQSIETMRILLKSGADANIINATGYNALHICCQTNNVEIAILLIDSGASILIPDK-NEKLP
SrInvs        RTILHISALSGKLGAVKLLTKYKADKEIQDSAQQTPLFIACENGFADLVSYLLDNKANDAIID--AEGRN
```

```
                    570       580       590       600       610       620       630
           ....|....|....|....|....|....|....|....|....|....|....|....|....|....|
MmInvs     LLHWAALGGNADVCQILIENK-INPNVQDYAGRTPLQCAAYGGYINCMAVLMENN---ADPNIQDKEGRT
DrInvs     ALHWAALGGNAEVCEVLMENG-ISPNLQDQAGRTPLQCAAYAGYINCMALLIQHD---ADPNIQDKEGRT
HpInvsL    PLHWAALGGHTCVCYHLMTHD-ISPNVQDNAGRTPLQCAAYGGFIRCMTLLLEHG---ADPNLQDNEGMT
SkInvs     PLHWAALGGHAVICETLMKHG-ISVDVRDHVGRTPLQCAAYGGYINCMSLLMENG---ADPNLQDHEGMT
AcInvs     PLHWAALGGHAYICQMLIKYG-VHPNIRDNSARTPLHCAAYGGFVNCMSALMEHG---AEPNSQDKEGMT
ChDgo      PLHYSAMVDRREIVNNLLQHG-LDPNAQDNSGCPPLHIAAYGGNVHCMNVLLENN---AQVNMQDNSGST
OcInvs1    PLHWAALGGHAYICLSLIQNG-STVDVKDKEGRSPLECASYSGFVNCMAVLLENG---CDVNTRDNEGIT
OlInvs1    PLHWAALGGHAYICLSLIQNG-STVDVKDKEGRSPLECASYGGFVNCMAVLLENG---CDVNTRDNEGIT
OspInvs1   PLHWAALGGHAYICLSLIQNG-ATVNIKDKEGRSSLECSSYGGFVNCMAVLLENG---CDVNTRDNEGIT
ScInvs1    PIHWSCLGGHDQVCTALIQSG-CDVNSPDTSERTPLQCAAHGGHMTCVSILLESG---ADPNKQDIEGIS
AqInvs1    CLHWAASGGHEFIVTTLIHSG-LPVDILDNENRSSLHCAAYNGSVGCCSELINAN---AFINQTDKDGVT
EmInvs1    SLHWAASGGHTTLCSTLFHQG-LPVDVADQGGRTPLHCAAYGGFSECVSVLLGLG---ADVNLQDNEGIS
AvInvs1    PLHYAALAHNLATNDFIISHS-SVLSPKDDFGRIPFHYAASCDNIIALKLLGIDA-----INFQDKTGYS
OmInvs1    PIHYAALTNSCSTIAFLISKG-NQLNTPDQMGRIPLHYACTLDNVELITMLGFAQ-----INSQDLSGFT
OcInvs2    VLHCAVRSRFVAAVECILQNSSIDVDGIDNISSSPLHYATAEGLTDVVTCLVTGG---ADPTLLGLDGLS
OlInvs2    VLHYAVHSRSVAAVELILQTN-VNVDGVDSINSSPLHYATTENLTDIVTCLVRYG---ADPALLGVDGLS
OspInvs2   LLHYAVHSRSVSMVELVLQAD-IHVDGVDSINSSPLHYATAEGLTDIVACLVKYG---ADPTRLGLDGLS
ScInvs2    CLHAIILGQHTASLKVLLSYS-ADANVYDAEGTSALQAAAFSGYQAAVTELLLN---GARPDDQGSSGLT
AqInvs2    VLHKAILSENSEILKALINAN-CNAEVPDSNGRTPLELSVYIGNKDITALLLQSS--NADPNAMDAYGLT
EmInvs2    PLHKATLNGQLDSVRALVISG-ADLNLYDSGGNTALHMAAFGGMEAILAVLVEN---RVDPNLQDPSGMT
AvInvs2    PLHIAVRHSSKDVLAFLALQT-ECVNLVTQDGLNALHFAAQMGDKVLVEILLNSPLSSTLINAVDSTGLS
OmInvs2    PLHMAVRHSSFDVVALLAVQP-LCVNLVTQDGLNALHFAAQLGEVTLVEILLNSPLKSSLLNAVDAAGLA
SrInvs     IVHAAAISGNKNVLAECLQLRPHDMNSVDERGETPVHYAAYFGQFDCVQELLSSG---AEPNCFDLEGVT

                    640       650       660       670       680       690       700
           ....|....|....|....|....|....|....|....|....|....|....|....|....|....|
MmInvs     ALHWSCNNGYLDAIKLLLDFA-AFPNQMENNEERYTPLDYALLGERHEVIQFMLEHGALSIAAIQDIAAF
DrInvs     ALHWSCNNGYLDAVKLLLGCG-AFPNHMEHTEERYTPLDYALLGEHQELTQFLLEHGALSIAAIQDIAAS
HpInvsL    ALHWACSTGYLDATRLLLDHG-AFPNHMELTEDRFTPLDYTLLNDHHEVSQYMVEQGALSITGIRDMAAT
SkInvs     ALHWACSSGCLDAIKLLFEYK-AFPNHMEFNEDRFTPLDYALLNDHHDVAQYMIEQGALSITGIRDLVAS
AcInvs     CLHWACSKGHLDAVKLLVEYA-AYPNHMEFTEERYTPLDYALMGEHHEVAQYMIEQGALSITGIQDIAAL
ChDgo      ALHLACRSGNLDAVKLLVSRYRANMNIFDGSEEKLTCLDYAILNDHQDVSFFLTENGANTISTLHDLS-L
OcInvs1    SIHWASASGHLEAIRLLFEFG-ANPNFMEVDGDRLTPLDYAIINDHQEAAQYMIEQGALSITGIQELAAT
OlInvs1    SIHWASASGHLEAIRLLFEYG-ANPNFMEVDGDRLTPLDYAIINDHQEAAQCLIEQGALSITGIQDLAAT
OspInvs1   PIHWASGGGHLEAIRLLFEYG-ANPNFMEVDGERLTPLDYAIIKDHQEVAQFMIEQGALSITGIQDLAAT
ScInvs1    ALHWSASQGHIEVVQLLLEFG-AFCNPMEVDGDRLTPLDYAIIGDHQEVAQFLMEQGALSITGIQDMAAT
AqInvs1    PLHWACAGGSSDCVQFLLSKG-ANPNAMDSS-EQLTPLDYAILENHQELAQALIINGALTIASIQELAAI
EmInvs1    GLHWACSAGHLDTVQLLLGAG-AVPNLMEANGDKLTCLDYAIIGNHQEIAQLLIEQGALSASGIHDLAAV
AvInvs1    ALHLAIIHGSENSVDFLISNG-AKLNQMDFSNERRTPFDLAIYFEKHNIISILQNSGCVSGQEIITLAVV
OmInvs1    PLHWAVMHENIHTIKILVEND-AKINVMDYSMQRRTPLDLSLFIEDREIYSFLRYLGCVTGIEIQTLAVI
OcInvs2    SLHLAVKNGMDVALVEALLR--GTPNPSIWNADQETPLDLCFNHNQLQLADSLVSAGAMKFIEIQSLAAT
OlInvs2    SLHIAVKNGSDASLVTALLS--GAPNPSVWNEDGETPLDFCYKFGQLQLVDTLINAGAMKFTDIQSFAAT
OspInvs2   SLHLAVKHGADKALLKFLLR--SAANPNVLNENRETPLDFCYKLNQFHLVNTLVGAGAVTFSHIESLAAT
ScInvs2    PLHLASGSGHDEVVEALLQRG-AAVNVRSFAEEDITALDAAKQNGHDGCAAILQQCGGMTMEEIQSKAAT
AqInvs2    MLHIAAKEGKHEILQLLLVNN-GYINSMDATEDRYTPLDYAIQGNHFECMTTLKSKGGMTVDEIRTIAAV
EmInvs2    ALHLSSIEGHEGCVRQLLSYN-AHPNIMDTTDQRCTPLDYARLHDHTPCADLLVEAGGLVSASIKEMAAI
AvInvs2    PIHHAILKEKIEIVAILCQFG-AYLDLQLRDFDSDTCLDLAINNNQQGIINVLKEYDAKTKFELETGAAV
OmInvs2    PIHHAVLKGNAPVVKVLCAYG-AYLYLQLIEFDFDTCLDLAVYNKQVEIVDVLKRYNALTTFEIRTKAAV
SrInvs     PLHWACSQGFPDVVRELLNYN-AYPNFTDRSEDQLTPLDYALSGGFQECVDLLVEARGATGDELRAFAAN
```

```
                  710       720       730       740       750       760       770
          ....|....|....|....|....|....|....|....|....|....|....|....|....|....|
MmInvs    KIQAVYKGYKVRKAFRDRKNLLMKHEQLRKDAAAKKREEENKRKEAE-----------------------
DrInvs    SIQALYKGYKVRRAFRERKKLLMRHEQLRKDAAKKR--EEERRREAE-----------------------
HpInvsL   RIQCRFRGFCVRKTFVERKKLLMKHEQLRKDAAAKKREEQSKKGDN------------------------
SkInvs    KIQAHYRGYLVRKTFLERKKLLMKHEQLRKDAAKKKEQETKKRQLE------------------------
AcInvs    KIQSAFKGFRVRKAFIERKKLFMKHEKLKKEAARKRAAEEGKKSEDQTVRKQDTMKSSTASIPGTEHQTP
ChDgo     KIQTNWRGYSARKRYAELKRKMNISTSIPKRRLTNLTTTSLGDNTLKVG---------------------
OcInvs1   AIQSAWRGHVIRRKSQARKEKADVRSSAASSRKVISEEE----RREE-----------------------
OlInvs1   AIQSAWRGHVVR-KAIAKKKEKREAPSASSGGRPVSVGS----RGDD-----------------------
OspInvs1  AIQSAWRGHLTR-KALAKKRSIESASSAKT-VRSGRDGA----AADK-----------------------
ScInvs1   AIQSAFRGRTARKRFRSMKSQTEIMRREEEERRLQQESANLLLTETA-----------------------
AqInvs1   MIQKVVRGYLARKRFKLLHAEKVKGEVKVEETGGTETEA-------------------------------
EmInvs1   IIQKWVRGYLSRKKAAILRAQKLQQRSMPQGLPSSRTSS-------------------------------
AvInvs1   EIQKFWRKMNRVCKRRMHANLSSTQN--------------------------------------------
OmInvs1   EIQRFWRHISRQ-RLKKNLRITSPEK--------------------------------------------
OcInvs2   AIQAAFRGWRTKKSLKSKKQRKNAAVIIQSYFRGFLGRKKFKQMVGR-----------------------
OlInvs2   AIQAAYRGWKARRELKGEAKLARAALKIQSYYRGYRGRKRYREVVRR-----------------------
OspInvs2  AIQAAYRGWRVRKELKVEERLANAALKIQSCFRGFQERKRYRNLLCR-----------------------
ScInvs2   IIQAVSRGYLARRSMLWLVERRLAVVAIQKTVRGFLQRRRFQSMLQR-----------------------
AqInvs2   WIQSSYRKYKAMKLLKKLRKERLAAVCLQRICRGFIERRRVKKLQKE-----------------------
EmInvs2   TIQANFRGHMARKLRKKLVTQSRAVTVISAAFRGYCQRKHYKQKKRE-----------------------
AvInvs2   VIQTCVRFFLARIQFLQLKRKAKAVSTISAYFKGFKARNFYFDLKRK-----------------------
OmInvs2   IIQSYLRCHLAKLQFANLKISSRAATVISANFRSFQTRLYYRNLKRQ-----------------------
SrInvs    TIQAAFRVFQAKRILQNLRKRKQSNAAIVIQAAARGFLERNRYKSMR-----------------------

                  780       790       800       810       820       830       840
          ....|....|....|....|....|....|....|....|....|....|....|....|....|....|
MmInvs    ----------------------QQKGQLDTDPPRSHCSSSAPVLPCPPSPQNEGSKQDATPSKQPPASHT
DrInvs    ----------------------QQLSFAEAGQKQRVLLAAVGVEKLSLDEAEQRVKDSVAAKGHKHKKSS
HpInvsL   ----------------------PDSSSTPLNSQSQDRISTSNEEMLPPPKDRTQLLENGWTQENANTNNN
SkInvs    ----------------------YRKREEQRRQRLHHKQGNESQNSTDSEQDK---INNQHRQHPVKETSV
AcInvs    QQQAEQLPTPPLVIQDSQESLPPDSRAESSSQTIKEEVPTERETGLSQQQQHQQKRQQQQKQQHEHKQVQ
ChDgo     ----------------------TLPPVSPTHAVLPHKANSHIGIPTARYRSNTQVSHTSNVSDSRQGPKF
OcInvs1   ----------------------ERRQREESARKKAS--ERRAALEKELADAER-IDDKEKQTELKR----
OlInvs1   ----------------------EKRKREESARKKAF--ERRAALERELADAER-LDDKERQTELKR----
OspInvs1  ----------------------AKESRDESARRKAF--ERRAALERELADAQR-FDDKERQVELKR----
ScInvs1   ----------------------QKRQRQELEQLIAHGKDRIARLEREVDGGQSSLPPIDIAAGNRM----
AqInvs1   -------------------------TDAVSSRPDSS---HRRSLELQVAES-------------------
EmInvs1   -------------------------RTSESTHTPTQ---PQAILKLEAPKSEAVVVKVGHRRELEA----
AvInvs1   ------------------------FVSKKNIEDPIS------QDNILVIKSRDVYIP-LTLPT-------
OmInvs1   ------------------------PVQKSRVQPSLRSIDSSLTNDVHKTNCDEKYFPNLEIHP-------
OcInvs2   ------------------------HKAAQVIQRNYRAFVMEKRRRKELIQQRKKTEMMVLVNDFHQRLLV
OlInvs2   ------------------------DKAARVVQRNYRNFVLRKERKRELLRLRRKTEVTVLISEFHERLLI
OspInvs2  ------------------------DKAARILQRKYRDFVQKRERQKELATVERRREITVLVNEFHERLLL
ScInvs2   ------------------------RNAAVTIQSYWRGHLERCSYRTKLTEYNQQAYHQRLIHTLHDQLT-
AqInvs2   ------------------------LKSSIIIQALVRGFLARRSYQKLLQKRIEEQKKHQLIEEFGQMFIG
EmInvs2   ------------------------LEAAIKIQAFYRGHLQRIKFRSKLAEFKEQRCNDERVLNFAKWLLG
AvInvs2   ------------------------QTNAVIIQSFYRGFLQRKIFKIDLQKFRAYNLHNFYIDMMHQNYLN
OmInvs2   ------------------------HLYATKIQAFFRGFQQRKRFVQDLANFRAVRTHNNYIDCINRLYLK
SrInvs    ----------------------EDPSQRHQQQEPANKDDEERKRKEEEERRRRQQEEEERRERERKAKAE
```

```
                     850       860       870       880       890       900       910
              ....|....|....|....|....|....|....|....|....|....|....|....|....|....|
MmInvs        VQSPDPEHSRLPGRCPGRASQGDSSIDLQGTASRKPSETPIEHCRGP-----------------------
DrInvs        SAHNSQSRREKPSRAERRTREPETSDAPSIRSLPPVTPMSTKKCPVT-----------------------
HpInvsL       ----------------------NNSAVDASVN--LSEGFAP-----------------------------
SkInvs        ----------------------TSSDTEESVSSSRSDKYSV-----------------------------
AcInvs        PQLQREERGEDPVAVNGKTLTSASSDSDSGAVTGSSSTVSSQGRRKHTQLQQQQQPASQKDIVVEKKKSP
ChDgo         IEGN---------AANPFSLNRVARVKSPGTEMRVPNSSRSKSSTG------------------------
OcInvs1       ----------------------RQRQLDHEERLREESARQPDSPKN------------------------
OlInvs1       ----------------------RQRQLEQEERLR---ERQQGSPRRY-----------------------
OspInvs1      ----------------------RQRQLEQEERLR---LSE--SPKR------------------------
ScInvs1       ----------------------VKASKDKGKGVRLPPISQHGQLTS------------------------
AqInvs1       -------------------------ERQAKEWALR-----------------------------------
EmInvs1       ----------------------RLKETDQKLQSLS-----------------------------------
AvInvs1       ----------------------------------------------------------------------
OmInvs1       ----------------------------------------------------------------------
OcInvs2       DK-----------------AKAKVKSEKGNGMEEKKENVIPDQMKLN-----------------------
OlInvs2       DR-----------------ARVKAKPIVD---RKGGEDSDKEKAVSN-----------------------
OspInvs2      DK-----------------RLVKAKPTVE---ERRRDK--EEKARFN-----------------------
ScInvs2       --------------------------QKWRQEGLTRTPPKAQPPVQT-----------------------
AqInvs2       -------------------LQRIEPIVIDADELHMKAKDKDHEGAKK-----------------------
EmInvs2       VGAG-DLTALAESERAPPPQPSFMSGHELRGAGIQSESACTAQDVRV-----------------------
AvInvs2       EIANSKFKKLTIEDLDFENSLFISPWRSSLREKRQETLDDRSRRLEN-----------------------
OmInvs2       GIRENTIKTFTIKDLDFTDALYISPWRSSLREKRQETVEDRIRRLEN-----------------------
SrInvs        QE-----------------EKLRKKKQKQEQERRRREEARKKKEAE------------------------

                     920       930       940       950       960       970       980
              ....|....|....|....|....|....|....|....|....|....|....|....|....|....|
MmInvs        ----------------------------------------------------------------------
DrInvs        ----------------------------------------------------------------------
HpInvsL       ----------------------------------------------------------------------
SkInvs        ----------------------------------------------------------------------
AcInvs        DPLTVEVQKKSLMPAPAPVTTLPKQDEPTRRQQQTVGTPDVKPKQRSRSGGRLQERGHSAPPRRSSKPIA
ChDgo         ----------------------------------------------------------------------
OcInvs1       ----------------------------------------------------------------------
OlInvs1       ----------------------------------------------------------------------
OspInvs1      ----------------------------------------------------------------------
ScInvs1       ----------------------------------------------------------------------
AqInvs1       ----------------------------------------------------------------------
EmInvs1       ----------------------------------------------------------------------
AvInvs1       ----------------------------------------------------------------------
OmInvs1       ----------------------------------------------------------------------
OcInvs2       ----------------------------------------------------------------------
OlInvs2       ----------------------------------------------------------------------
OspInvs2      ----------------------------------------------------------------------
ScInvs2       ----------------------------------------------------------------------
AqInvs2       ----------------------------------------------------------------------
EmInvs2       ----------------------------------------------------------------------
AvInvs2       ----------------------------------------------------------------------
OmInvs2       ----------------------------------------------------------------------
SrInvs        ----------------------------------------------------------------------
```

```
                 990       1000      1010      1020      1030      1040      1050
          ....|....|....|....|....|....|....|....|....|....|....|....|....|....|
MmInvs    ----------------------------------------------------------------------
DrInvs    ----------------------------------------------------------------------
HpInvsL   ----------------------------------------------------------------------
SkInvs    ----------------------------------------------------------------------
AcInvs    LSYAQLLQQKDAMQRQQKPLQTKQLQSKQQPQRKSKQQQVTQQQLSKQQTHLPKQQTQPRLKQQLRQPKQ
ChDgo     ----------------------------------------------------------------------
OcInvs1   ----------------------------------------------------------------------
OlInvs1   ----------------------------------------------------------------------
OspInvs1  ----------------------------------------------------------------------
ScInvs1   ----------------------------------------------------------------------
AqInvs1   ----------------------------------------------------------------------
EmInvs1   ----------------------------------------------------------------------
AvInvs1   ----------------------------------------------------------------------
OmInvs1   ----------------------------------------------------------------------
OcInvs2   ----------------------------------------------------------------------
OlInvs2   ----------------------------------------------------------------------
OspInvs2  ----------------------------------------------------------------------
ScInvs2   ----------------------------------------------------------------------
AqInvs2   ----------------------------------------------------------------------
EmInvs2   ----------------------------------------------------------------------
AvInvs2   ----------------------------------------------------------------------
OmInvs2   ----------------------------------------------------------------------
SrInvs    ----------------------------------------------------------------------

                1060      1070      1080      1090      1100      1110      1120
          ....|....|....|....|....|....|....|....|....|....|....|....|....|....|
MmInvs    -----------------------SACVHPRSWEGGNSSKNQGTSSVEKRRGETNGKHRRCEEGPSSARQP
DrInvs    -----------------------REEVCVRETFGPDTGISLNCGSANERRS------------PAGSSRP
HpInvsL   -----------------------SSEMLSRDEDG------------EKMGVAM-----KIGPPLLGSSNP
SkInvs    -----------------------SSDRYSDNFED------------EEM---------------------
AcInvs    QLQQQQQTPPLRQQAQPPHLPPGSAEPRSRSRKGRKSSNTSALSVSVELSSSA-----SVDGTGASATPP
ChDgo     ----------------------------------------------------------------------
OcInvs1   ----------------------------------------------------Q-----------------
OlInvs1   ----------------------------------------------------Q-----------------
OspInvs1  ----------------------------------------------------Q-----------------
ScInvs1   ----------------------------------------------------S-----------------
AqInvs1   ----------------------------------------------------------------------
EmInvs1   ----------------------------------------------------------------------
AvInvs1   ----------------------------------------------------------------------
OmInvs1   ----------------------------------------------------------------------
OcInvs2   ----------------------------------------------------------------------
OlInvs2   ----------------------------------------------------------------------
OspInvs2  ----------------------------------------------------------------------
ScInvs2   ----------------------------------------------------------------------
AqInvs2   ----------------------------------------------------------------------
EmInvs2   ----------------------------------------------------------------------
AvInvs2   ----------------------------------------------------------------------
OmInvs2   ----------------------------------------------------------------------
SrInvs    ----------------------------------------------------------------------
```

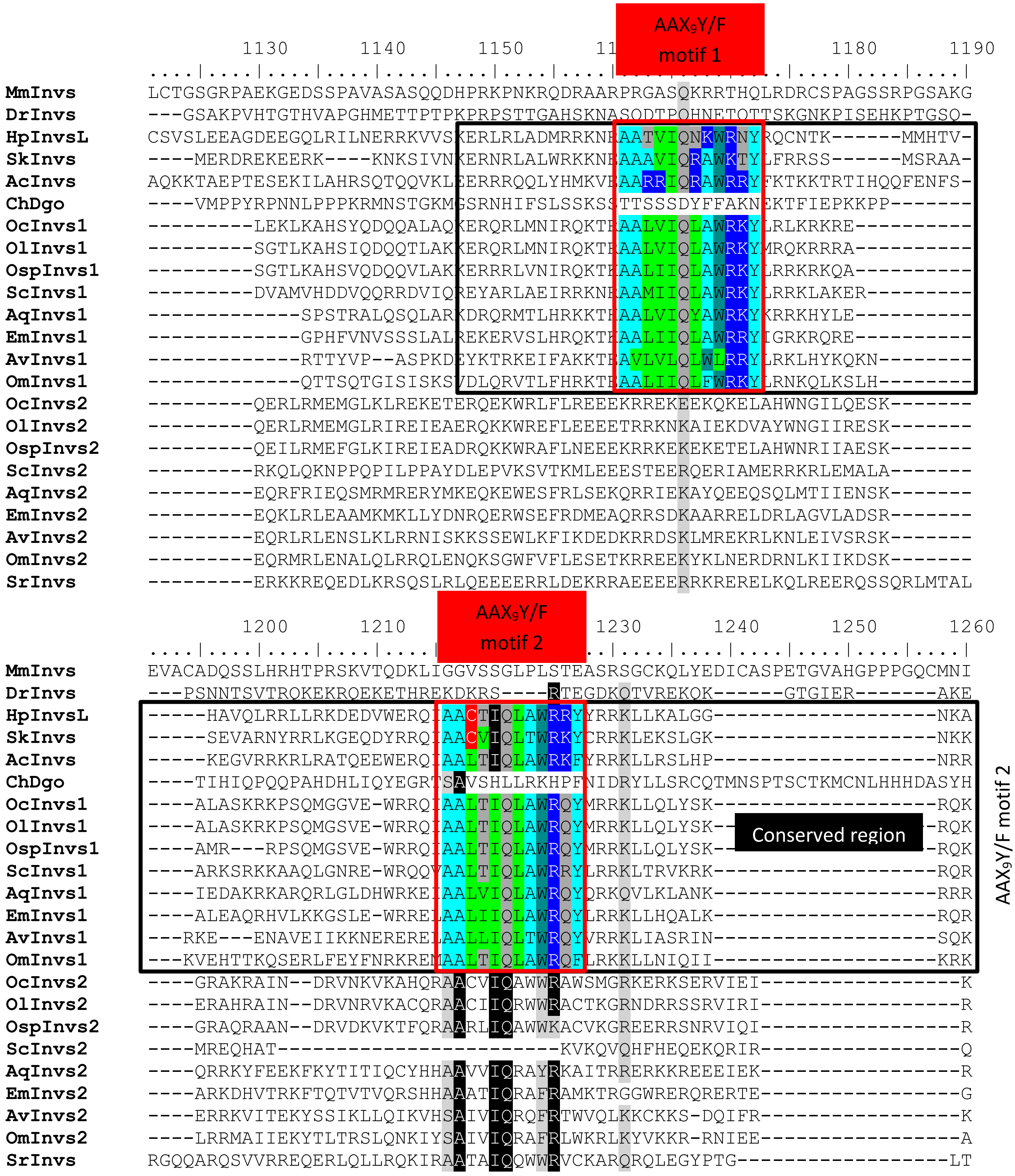

AAX9Y/F motif 1
1130 1140 1150 1180 1190
MmInvs LCTGSGRPAEKGEDSSPAVASASQQDHPRKPNKRQDRAARPRGASQKRRTHQLRDRCSPAGSSRPGSAKG
DrInvs ---GSAKPVHTGTHVAPGHMETTPTPKPRPSTTGAHSKNASQDTPQHNETQTTSKGNKPISEHKPTGSQ-
HpInvsL CSVSLEEAGDEEGQLRILNERRKVVSKERLRLADMRRKNRAATVIQNKWRNYRQCNTK------MMHTV-
SkInvs ----MERDREKEERK----KNKSIVNKERNRLALWRKKNRAAAVIQRAWKTYLFRRSS------MSRAA-
AcInvs AQKKTAEPTESEKILAHRSQTQQVKLEERRRQQLYHMKVRAARRIQRAWRRYFKTKKTRTIHQQFENFS-
ChDgo ----VMPPYRPNNLPPPKRMNSTGKMGSRNHIFSLSSKSSTTSSSDYFFAKNEKTFIEPKKPP-------
OcInvs1 ---------LEKLKAHSYQDQQALAQKERQRLMNIRQKTRAALVIQLAWRKYLRLKRKRE----------
OlInvs1 ---------SGTLKAHSIQDQQTLAKKERQRLMNIRQKTRAALVIQLAWRKYMRQKRRRA----------
OspInvs1 ---------SGTLKAHSVQDQQVLAKKERRRLVNIRQKTRAALIIQLAWRKYLRRKRKQA----------
ScInvs1 ---------DVAMVHDDVQQRRDVIQREYARLAEIRRKNRAAMIIQLAWRKYLRRKLAKER---------
AqInvs1 -------------SPSTRALQSQLARKDRQRMTLHRKKTRAALVIQYAWRKYKRRKHYLE----------
EmInvs1 -------------GPHFVNVSSSLALREKERVSLHRQKTRAALIIQLAWRRYIGRKRQRE----------
AvInvs1 -------------RTTYVP--ASPKDEYKTRKEIFAKKTRAVLVLQLWLRRYLRKLHYKQKN--------
OmInvs1 -------------QTTSQTGISISKSVDLQRVTLFHRKTRAALIIQLFWRKYLRNKQLKSLH--------
OcInvs2 ---------QERLRMEMGLKLREKETERQEKWRLFLREEEKRREKEEKQKELAHWNGILQESK-------
OlInvs2 ---------QERLRMEMGLRIREIEAERQKKWREFLEEEETRRKNKAIEKDVAYWNGIIRESK-------
OspInvs2 ---------QEILRMEFGLKIREIEADRQKKWRAFLNEEEKRRKEKEKETELAHWNRIIAESK-------
ScInvs2 ---------RKQLQKNPPQPILPPAYDLEPVKSVTKMLEEESTEERQERIAMERRKRLEMALA-------
AqInvs2 ---------EQRFRIEQSMRMRERYMKEQKEWESFRLSEKQRRIEKAYQEEQSQLMTIIENSK-------
EmInvs2 ---------EQKLRLEAAMKMKLLYDNRQERWSEFRDMEAQRRSDKAARRELDRLAGVLADSR-------
AvInvs2 ---------EQRLRLENSLKLRRNISKKSSEWLKFIKDEDKRRDSKLMREKRLKNLEIVSRSK-------
OmInvs2 ---------EQRMRLENALQLRRQLENQKSGWFVFLESETKRREEKYKLNERDRNLKIIKDSK-------
SrInvs ---------ERKKREQEDLKRSQSLRLQEEEERRLDEKRRAEEEERRKREKELKQLREERQSSQRLMTAL
AAX9Y/F motif 2
1200 1210 1230 1240 1250 1260
MmInvs EVACADQSSLHRHTPRSKVTQDKLIGGVSSGLPLSTEASRSGCKQLYEDICASPETGVAHGPPPGQCMNI
DrInvs ---PSNNTSVTRQKEKRQEKETHREKDKRS----RTEGDKQTVREKQK------GTGIER-------AKE
HpInvsL -----HAVQLRRLLRKDEDVWERQIAACTIQLAWRRYYRRKLLKALGG-------------------NKA
SkInvs -----SEVARNYRRLKGEQDYRRQIAACVIQLTWRKYCRRKLEKSLGK-------------------NKK
AcInvs -----KEGVRRKRLRATQEEWERQIAALTIQLAWRKFYRRKLLRSLHP-------------------NRR
ChDgo ----TIHIQPQQPAHDHLIQYEGRTSAVSHLLRKHPFNIDRYLLSRCQTMNSPTSCTKMCNLHHHDASYH
OcInvs1 ----ALASKRKPSQMGGVE-WRRQIAALTIQLAWRQYMRRKLLQLYSK-------------------RQK
OlInvs1 ----ALASKRKPSQMGSVE-WRRQIAALTIQLAWRQYMRRKLLQLYSK-------------------RQK
OspInvs1 ----AMR---RPSQMGSVE-WRRQIAALTIQLAWRQYMRRKLLQLYSK-------------------RQK
ScInvs1 ----ARKSRKKAAQLGNRE-WRQQYAALTIQLAWRRYLRRKLTRVKRK-------------------RQR
AqInvs1 ----IEDAKRKARQRLGLDHWRKEIAALVIQLAWRQYQRKQVLKLANK-------------------RRR
EmInvs1 ----ALEAQRHVLKKGSLE-WRRELAALIIQLAWRQYLRRKLLHQALK-------------------RQR
AvInvs1 ---RKE---ENAVEIIKKNERERELAALLIQLTWRQYVRRKLIASRIN-------------------SQK
OmInvs1 ---KVEHTTKQSERLFEYFNRKREMAALTIQLAWRQFLRKKLLNIQII-------------------KRK
OcInvs2 ----GRAKRAIN--DRVNKVKAHQRAACVIQAWWRAWSMGRKERKSERVIEI-----------------K
OlInvs2 ----ERAHRAIN--DRVNRVKACQRAACIIQRWWRACTKGRNDRRSSRVIRI-----------------R
OspInvs2 ----GRAQRAAN--DRVDKVKTFQRAARLIQAWWKACVKGREERRSNRVIQI-----------------R
ScInvs2 ----MREQHAT-----------------------KVKQVQHFHEQEKQRIR-----------------Q
AqInvs2 ----QRRKYFEEKFKYTITIQCYHHAAVVIQRAYRKAITRRERKKREEEIEK-----------------R
EmInvs2 ----ARKDHVTRKFTQTVTVQRSHHAAATIQRAFRAMKTRGGWRERQRERTE-----------------G
AvInvs2 ----ERRKVITEKYSSIKLLQIKVHSAIVIQRQFRTWVQLKKCKKS-DQIFR-----------------K
OmInvs2 ----LRRMAIIEKYTLTRSLQNKIYSAIVIQRAFRLWKRLKYVKKR-RNIEE-----------------A
SrInvs RGQQARQSVVRREQERLQLLRQKIRAATAIQQWWRVCKARQRQLEGYPTG-----------------LT
Conserved region
AAX9Y/F motif 2

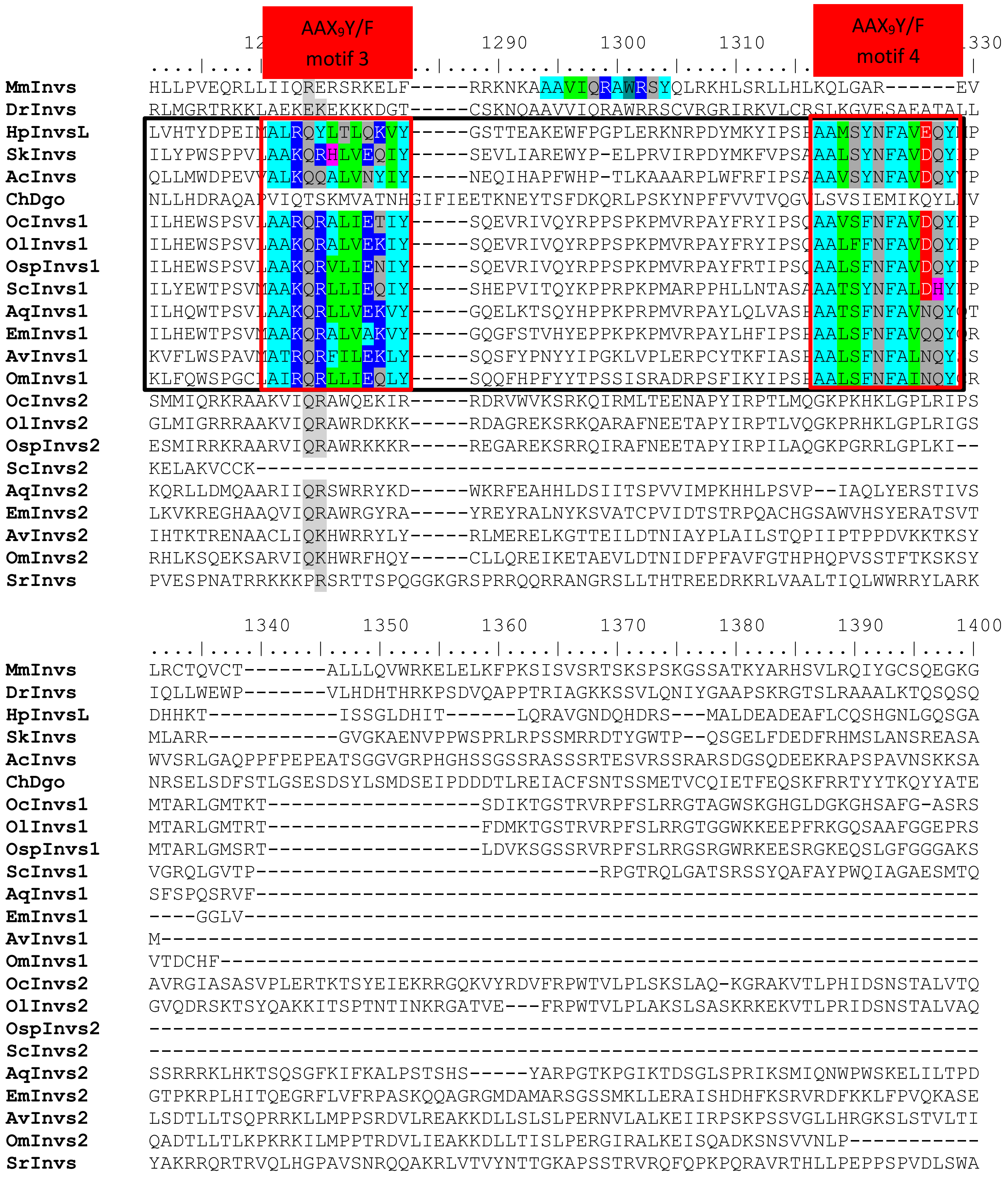

AAX9Y/F motif 3
AAX9Y/F motif 4
12 1290 1300 1310 1330
MmInvs HLLPVEQRLLIIQRERSRKELF-----RRKNKAAAVIQRAWRSYQLRKHLSRLLHLKQLGAR------EV
DrInvs RLMGRTRKKLAEKEKEKKKDGT-----CSKNQAAVVIQRAWRRSCVRGRIRKVLCRSLKGVESAEATALL
HpInvsL LVHTYDPEIMALRQYLTLQKVY-----GSTTEAKEWFPGPLERKNRPDYMKYIPSEAAMSYNFAVEQY[illegible]P
SkInvs ILYPWSPPVLAAKQRHLVEQIY-----SEVLIAREWYP-ELPRVIRPDYMKFVPSAAALSYNFAVDQY[illegible]P
AcInvs QLLMWDPEVVALKQQALVNYIY-----NEQIHAPFWHP-TLKAAARPLWFRFIPSAAAVSYNFAVDQY[illegible]P
ChDgo NLLHDRAQAPVIQTSKMVATNHGIFIEETKNEYTSFDKQRLPSKYNPFFVVTVQGVLSVSIEMIKQYL[illegible]V
OcInvs1 ILHEWSPSVLAARQRALIETIY-----SQEVRIVQYRPPSPKPMVRPAYFRYIPSQAAVSFNFAVDQY[illegible]P
OlInvs1 ILHEWSPSVLAAKQRALVEKIY-----SQEVRIVQYRPPSPKPMVRPAYFRYIPSQAALFFNFAVDQY[illegible]P
OspInvs1 ILHEWSPSVLAAKQRVLIENIY-----SQEVRIVQYRPPSPKPMVRPAYFRTIPSQAALSFNFAVDQY[illegible]P
ScInvs1 ILYEWTPSVMAAKQRLLIEQIY-----SHEPVITQYKPPRPKPMARPPHLLNTASAAATSYNFALDHY[illegible]P
AqInvs1 ILHQWTPSVLAAKQRLLVEKVY-----GQELKTSQYHPPKPRPMVRPAYLQLVASEAATSFNFAVNQY[illegible]T
EmInvs1 ILHEWTPSVMAAKQRALVAKVY-----GQGFSTVHYEPPKPKPMVRPAYLHFIPSEAALSFNFAVQQY[illegible]R
AvInvs1 KVFLWSPAVMATRQRFILEKLY-----SQSFYPNYYIPGKLVPLERPCYTKFIASEAALSFNFALNQY[illegible]S
OmInvs1 KLFQWSPGCLAIRQRLLIEQLY-----SQQFHPFYYTPSSISRADRPSFIKYIPSEAALSFNFAINQY[illegible]R
OcInvs2 SMMIQRKRAAKVIQRAWQEKIR-----RDRVWVKSRKQIRMLTEENAPYIRPTLMQGKPKHKLGPLRIPS
OlInvs2 GLMIGRRRAAKVIQRAWRDKKK-----RDAGREKSRKQARAFNEETAPYIRPTLVQGKPRHKLGPLRIGS
OspInvs2 ESMIRRKRAARVIQRAWRKKKR-----REGAREKSRRQIRAFNEETAPYIRPILAQGKPGRRLGPLKI--
ScInvs2 KELAKVCCK---
AqInvs2 KQRLLDMQAARIIQRSWRRYKD-----WKRFEAHHLDSIITSPVVIMPKHHLPSVP--IAQLYERSTIVS
EmInvs2 LKVKREGHAAQVIQRAWRGYRA-----YREYRALNYKSVATCPVIDTSTRPQACHGSAWVHSYERATSVT
AvInvs2 IHTKTRENAACLIQKHWRRYLY-----RLMERELKGTTEILDTNIAYPLAILSTQPIIPTPPDVKKTKSY
OmInvs2 RHLKSQEKSARVIQKHWRFHQY-----CLLQREIKETAEVLDTNIDFPFAVFGTHPHQPVSSTFTKSKSY
SrInvs PVESPNATRRKKKPRSRTTSPQGGKGRSPRRQQRRANGRSLLTHTREEDRKRLVAALTIQLWWRRYLARK
1340 1350 1360 1370 1380 1390 1400
MmInvs LRCTQVCT-------ALLLQVWRKELELKFPKSISVSRTSKSPSKGSSATKYARHSVLRQIYGCSQEGKG
DrInvs IQLLWEWP-------VLHDHTHRKPSDVQAPPTRIAGKKSSVLQNIYGAAPSKRGTSLRAAALKTQSQSQ
HpInvsL DHHKT-----------ISSGLDHIT------LQRAVGNDQHDRS---MALDEADEAFLCQSHGNLGQSGA
SkInvs MLARR-----------GVGKAENVPPWSPRLRPSSMRRDTYGWTP--QSGELFDEDFRHMSLANSREASA
AcInvs WVSRLGAQPPFPEPEATSGGVGRPHGHSSGSSRASSSRTESVRSSRARSDGSQDEEKRAPSPAVNSKKSA
ChDgo NRSELSDFSTLGSESDSYLSMDSEIPDDDTLREIACFSNTSSMETVCQIETFEQSKFRRTYYTKQYYATE
OcInvs1 MTARLGMTKT------------------SDIKTGSTRVRPFSLRRGTAGWSKGHGLDGKGHSAFG-ASRS
OlInvs1 MTARLGMTRT------------------FDMKTGSTRVRPFSLRRGTGGWKKEEPFRKGQSAAFGGEPRS
OspInvs1 MTARLGMSRT------------------LDVKSGSSRVRPFSLRRGSRGWRKEESRGKEQSLGFGGGAKS
ScInvs1 VGRQLGVTP-----------------------------RPGTRQLGATSRSSYQAFAYPWQIAGAESMTQ
AqInvs1 SFSPQSRVF---
EmInvs1 ----GGLV--
AvInvs1 M---
OmInvs1 VTDCHF--
OcInvs2 AVRGIASASVPLERTKTSYEIEKRRGQKVYRDVFRPWTVLPLSKSLAQ-KGRAKVTLPHIDSNSTALVTQ
OlInvs2 GVQDRSKTSYQAKKITSPTNTINKRGATVE---FRPWTVLPLAKSLSASKRKEKVTLPRIDSNSTALVAQ
OspInvs2 --
ScInvs2 --
AqInvs2 SSRRRKLHKTSQSGFKIFKALPSTSHS-----YARPGTKPGIKTDSGLSPRIKSMIQNWPWSKELILTPD
EmInvs2 GTPKRPLHITQEGRFLVFRPASKQQAGRGMDAMARSGSSMKLLERAISHDHFKSRVRDFKKLFPVQKASE
AvInvs2 LSDTLLTSQPRRKLLMPPSRDVLREAKKDLLSLSLPERNVLALKEIIRPSKPSSVGLLHRGKSLSTVLTI
OmInvs2 QADTLLTLKPKRKILMPPTRDVLIEAKKDLLTISLPERGIRALKEISQADKSNSVVNLP-----------
SrInvs YAKRRQRTRVQLHGPAVSNRQQAKRLVTVYNTTGKAPSSTRVRQFQPKPQRAVRTHLLPEPPSPVDLSWA

```
                    1410      1420      1430      1440      1450      1460      1470
           ....|....|....|....|....|....|....|....|....|....|....|....|....|....|
MmInvs     HHPIKSSKAPAVLHLSSVNSLQSIHLDNSGRSKKFSYNLQPSSQSKNKPKL-------------------
DrInvs     SQVLLDLSLRTHKQLSAVECVN--LVDSVSQAKQFSYHLRPSSGASQSSQN-------------------
HpInvsL    P-YSYNLND-------------------------------------------------------------
SkInvs     DHFSYNLHR-------------------------------------------------------------
AcInvs     LSSSNTKKKS------------------------------------------------------------
ChDgo      LFICLKTKTFSKSRKFYYFIEWKGSLEGRYENEKNNRISIYRLDFARCYWCGSEFTEEAEKSQTGSFIFL
OcInvs1    VAGSILF---------------------------------------------------------------
OlInvs1    LASSVLF---------------------------------------------------------------
OspInvs1   IASSVIF---------------------------------------------------------------
ScInvs1    QFQVLDFN--------------------------------------------------------------
AqInvs1    ----------------------------------------------------------------------
EmInvs1    ----------------------------------------------------------------------
AvInvs1    ----------------------------------------------------------------------
OmInvs1    ----------------------------------------------------------------------
OcInvs2    K---------------------------------------------------------------------
OlInvs2    K---------------------------------------------------------------------
OspInvs2   ----------------------------------------------------------------------
ScInvs2    ----------------------------------------------------------------------
AqInvs2    ETNEESLKLPNIMLRQHTGHRSNNPLAMTPIKLPLLK---------------------------------
EmInvs2    TTRGSRSSERRIAVEQDPSQRGPSQRGHSQQGLSQRGPLQRGPSQQSHSQQGPSQQSRSQRGLPQLLKAL
AvInvs2    PLDSKIDPFRNFKSQIAHVRSEFELPDNLPHRNPETNRKSYVMKRANSLQILKTDSSLPFVGSETKQIPI
OmInvs2    VLDAEIPIC-------------------------------------------------------------
SrInvs     SAASTYFKPPNPGRRRPRGLHATRRRQRQPAYANMQDPEHMLTLPIIHGTQGSRKNDARLNQAVSLPPV-

                    1480      1490      1500      1510      1520      1530      1540
           ....|....|....|....|....|....|....|....|....|....|....|....|....|....|
MmInvs     ----------------------------------------------------------------------
DrInvs     ----------------------------------------------------------------------
HpInvsL    ----------------------------------------------------------------------
SkInvs     ----------------------------------------------------------------------
AcInvs     ----------------------------------------------------------------------
ChDgo      PFDGKYLPKRASKTHKETIHEPFVTKTVHSNRDLQNEAALSQRRYTVQGRYRSIEKRKTKIIFKLERIDL
OcInvs1    ----------------------------------------------------------------------
OlInvs1    ----------------------------------------------------------------------
OspInvs1   ----------------------------------------------------------------------
ScInvs1    ----------------------------------------------------------------------
AqInvs1    ----------------------------------------------------------------------
EmInvs1    ----------------------------------------------------------------------
AvInvs1    ----------------------------------------------------------------------
OmInvs1    ----------------------------------------------------------------------
OcInvs2    ----------------------------------------------------------------------
OlInvs2    ----------------------------------------------------------------------
OspInvs2   ----------------------------------------------------------------------
ScInvs2    ----------------------------------------------------------------------
AqInvs2    ----------------------------------------------------------------------
EmInvs2    PTGIRKSYTAPVQLPLIVNHTQS-----------------------------------------------
AvInvs2    LTETKSSFTSLPPIVQK-----------------------------------------------------
OmInvs2    ----------------------------------------------------------------------
SrInvs     ----------------------------------------------------------------------
```

```
                1550
            ....|....|....
MmInvs      --------------
DrInvs      --------------
HpInvsL     --------------
SkInvs      --------------
AcInvs      --------------
ChDgo       YIYFVMNFSIDAFT
OcInvs1     --------------
OlInvs1     --------------
OspInvs1    --------------
ScInvs1     --------------
AqInvs1     --------------
EmInvs1     --------------
AvInvs1     --------------
OmInvs1     --------------
OcInvs2     --------------
OlInvs2     --------------
OspInvs2    --------------
ScInvs2     --------------
AqInvs2     --------------
EmInvs2     --------------
AvInvs2     --------------
OmInvs2     --------------
SrInvs      --------------
```

Abbreviations. Vertebrate: Dr, *Danio rerio* and Mm, *Mus musculus.* Hexapoda: Dm, *Drosophila melanogaster.* Mollusca: Ac, *Aplysia californica* and Cg, *Crassotrea gigantea*. Ambulacraria: Hp, *Hemicentrotus pulcherrimus*; Sk and *Saccoglossus kowalevskii*. Cnidaria: Ch, *Clytia hemisphaerica.* Porifera: Aq, *Amphimedon queenslandica*; Av, *Aphrocalistes vastus*; Em, *Ephydatia muelleri*; *Oc, Oscarella carmela*; *Ol, Oscarella lobularis*; *Om, Oopsacas minuta*; *Osp, Oscarella sp.*; Sc, *Sycon ciliatum*. Choanoflagellata: Sr, *Salpingoeca rosetta*.

Figure S2.5: PET family alignment. Threshold for shading has been fixed at 80% for both identity (black) and similarity (grey).

```
                         10        20        30        40        50        60        70
               ....|....|....|....|....|....|....|....|....|....|....|....|....|....|
MmPkL1         ----------------------------------------------------------------------
MmPkL2         ----------------------------------------------------------------------
MmPkL3         ----------------------------------------------------------------------
DmPk           MSSLSTGGGAGGSSGGPGGADAAAAPAAGQATVTATGNMEPAMVPRTANLLACKQWWRVCFLYGDQQKYY
DmEsn          ----------------------------------------------------------------------
ChPk           ----------------------------------------------------------------------
TaPk           ----------------------------------------------------------------------
MmTes          ----------------------------------------------------------------------
DmTes          ----------------------------------------------------------------------
ChTes          ----------------------------------------------------------------------
OcPkTesL       ----------------------------------------------------------------------
OlPkTesL       ----------------------------------------------------------------------
OspPkTesL      ----------------------------------------------------------------------
ScPkTesL       ----------------------------------------------------------------------
AqPkTesL       ----------------------------------------------------------------------
EmPkTesL       ----------------------------------------------------------------------
AvPkTesL       ----------------------------------------------------------------------
OmPkTesL       ----------------------------------------------------------------------
MlPkTesL       ----------------------------------------------------------------------
SrPkTesL       ----------------------------------------------------------------------

                         80        90       100       110       120       130       140
               ....|....|....|....|....|....|....|....|....|....|....|....|....|....|
MmPkL1         ----------------------------------------------------------------------
MmPkL2         ----------------------------------------------------------------------
MmPkL3         ----------------------------------------------------------------------
DmPk           RQLYSKAAAQRLADANQEPDNARDREYDTVDCDLIAGQLDAVEDADDGIDLGDHSSTPKGGATTAGRPLF
DmEsn          ----------------------------------------------------------------------
ChPk           ----------------------------------------------------------------------
TaPk           ----------------------------------------------------------------------
MmTes          ----------------------------------------------------------------------
DmTes          ----------------------------------------------------------------------
ChTes          ----------------------------------------------------------------------
OcPkTesL       ----------------------------------------------------------------------
OlPkTesL       ----------------------------------------------------------------------
OspPkTesL      ----------------------------------------------------------------------
ScPkTesL       ----------------------------------------------------------------------
AqPkTesL       ----------------------------------------------------------------------
EmPkTesL       ----------------------------------------------------------------------
AvPkTesL       ----------------------------------------------------------------------
OmPkTesL       ----------------------------------------------------------------------
MlPkTesL       ----------------------------------------------------------------------
SrPkTesL       ----------------------------------------------------------------------
```

```
                    150       160       170       180       190       200       210
            ....|....|....|....|....|....|....|....|....|....|....|....|....|....|
MmPkL1      ----------------------------------------------------------------------
MmPkL2      ----------------------------------------------------------------------
MmPkL3      ----------------------------------------------------------------------
DmPk        PHSSSPRRSKKLLRSLRAHVRGEKLPKNDTTTANESSEVTQRNARVTVLDDPFLFGIDADHLGDLVVRGK
DmEsn       ----------------------------------------------------------------------
ChPk        ----------------------------------------------------------------------
TaPk        ----------------------------------------------------------------------
MmTes       ----------------------------------------------------------------------
DmTes       ----------------------------------------------------------------------
ChTes       ----------------------------------------------------------------------
OcPkTesL    ----------------------------------------------------------------------
OlPkTesL    ----------------------------------------------------------------------
OspPkTesL   ----------------------------------------------------------------------
ScPkTesL    ----------------------------------------------------------------------
AqPkTesL    ----------------------------------------------------------------------
EmPkTesL    ----------------------------------------------------------------------
AvPkTesL    ----------------------------------------------------------------------
OmPkTesL    ----------------------------------------------------------------------
MlPkTesL    ----------------------------------------------------------------------
SrPkTesL    ----------------------------------------------------------------------

                    220       230       240       250       260       270       280
            ....|....|....|....|....|....|....|....|....|....|....|....|....|....|
MmPkL1      ----------------------------------------------------------------------
MmPkL2      ----------------------------------------------------------------------
MmPkL3      ----------------------------------------------------------------------
DmPk        RYSTLDATENMARFYAEQEATAQVLEIIEQEEESPEQEAPKPALPPKQKQQRPVPPLPPPPANRVTQDQG
DmEsn       -----------------------------------MQQAP-------QQQQHPHP----PSSSYYTQTES
ChPk        ----------------------------------------------------------------------
TaPk        ----------------------------------------------------------------------
MmTes       ----------------------------------------------------------------------
DmTes       ----------------------------------------------------------------------
ChTes       ----------------------------------------------------------------------
OcPkTesL    ----------------------------------------------------------------------
OlPkTesL    ----------------------------------------------------------------------
OspPkTesL   ----------------------------------------------------------------------
ScPkTesL    ----------------------------------------------------------------------
AqPkTesL    ----------------------------------------------------------------------
EmPkTesL    ----------------------------------------------------------------------
AvPkTesL    ----------------------------------------------------------------------
OmPkTesL    ----------------------------------------------------------------------
MlPkTesL    ----------------------------------------------------------------------
SrPkTesL    ----------------------------------------------------------------------
```

```
                  290       300       310       320       330       340       350
          ....|....|....|....|....|....|....|....|....|....|....|....|....|....|
MmPkL1    ----------------------------------------------------------------------
MmPkL2    ----------------------------------------------------------------------
MmPkL3    ----------------------------------------------------------------------
DmPk      TQPAAPQVPLQPLTAGDLQFLNLSLRQRSLPRSMKPFKDAHDISFTFNELDTSAEPEVATGAAQQESNEP
DmEsn     --------ELLQIEAGGTGLTFASHSQRPES--------------AISQVASTAHLDVPSAASS------
ChPk      ----------------------------------------------------------------------
TaPk      ----------------------------------------------------------------------
MmTes     ----------------------------------------------------------------------
DmTes     ----------------------------------------------------------------------
ChTes     ----------------------------------------------------------------------
OcPkTesL  ----------------------------------------------------------------------
OlPkTesL  ----------------------------------------------------------------------
OspPkTesL ----------------------------------------------------------------------
ScPkTesL  ----------------------------------------------------------------------
AqPkTesL  ----------------------------------------------------------------------
EmPkTesL  ----------------------------------------------------------------------
AvPkTesL  ----------------------------------------------------------------------
OmPkTesL  ----------------------------------------------------------------------
MlPkTesL  ----------------------------------------------------------------------
SrPkTesL  --------------------------------------------MEGSKKKKRVGVKFAAEAEVMVHEDP

                  360       370       380       390       400       410       420
          ....|....|....|....|....|....|....|....|....|....|....|....|....|....|
MmPkL1    ----------------------------------------------------------------------
MmPkL2    ----------------------------------------------------------------------
MmPkL3    -------------------------------------------------MFARGSRRRRSGRAPPEAEDP
DmPk      ISRTPLTQISYLQKIPTLPRHFSPSGQGLATPPALGSGGMGLPSSSSASALYAAQAAAGILPTSPLPLQR
DmEsn     -----------------------------------GSGGSAVSGGSGGAPESAGRFVS--------PLQR
ChPk      --------MLPDTSSNNHNNKYSRTKHQHHVTHNNNNNTMVSMTTEQSKEQRSMTSSSLNEDLYFSATED
TaPk      --------------------------------------------------------------PAEATRDS
MmTes     --------------------------------------------MSATHPTRLGTRTKESNACASQGLVR
DmTes     --------------------------------MSAVSPNSCVETPKAPEWLSKLESRREQLKRSKLGHEA
ChTes     ---------------------------------------------MAEEEAVEKEVIKATEITHKLGHER
OcPkTesL  --------------------------------------------MDALQRKKLQSVRIAAVKQGVLFHNV
OlPkTesL  --------------------------------------------MDPAQRKRLESVRKAAVKQGVLFHDV
OspPkTesL -------------------------------------------MADAAQRKKLQSVRKAAVKQGVLFHNV
ScPkTesL  ------------------------------------MSVRRGMPRANPKTSNHEIVKGTEFRTGRTRDPD
AqPkTesL  ------------MAE-------------------------------QKRVNRLPSIRSRAIETGVLVHEV
EmPkTesL  ------------MSDS---------------------NDKGLLKPTRKPTKVYASVRDVAIKKGLLAHEV
AvPkTesL  ------------MARYLPTKNSITLDVHQDDEFHQAFTDIKVRDDSTKTDRNLASVRAIADKRGILVHDV
OmPkTesL  ------------MARHSGQSQTFADKSMSGDEFHQAFTNFSKG-FASENSTNFASVREIAVKRGILVHDV
MlPkTesL  -----------------------------------------MAIRPENLTNKTNRETNFCFSKNRGARES
SrPkTesL  SDVPKPSAGKLRRVKVPVKAGDGSKPDADGAQEENEITRKRRGLRRKPTVKGANEVIDAGLKQGVLYHDV
```

```
                   430       440       450       460       470       480       490
             ....|....|....|....|....|....|....|....|....|....|....|....|....|....|
MmPkL1       -----------------------------------------------------------MPLEMEPKMSK
MmPkL2       -------------------------------------------------------MVTVMPLEMEKTISK
MmPkL3       ARGQPCNSCR-EQCPGFLLHGWRKICQHCKCP----REEH---------------AVRTVPVDLERIMCR
DmPk         HQQYLPPHHQ-QHPGAGMGPGPGSGAAAGPPL----GPQYSPGCSANPKY-----SNAQLPPPPHHHHQL
DmEsn        RHCQPPSH--------------------LPL-----NSVASPLRTASYKS-----AAAVAGHGFHHSHHQ
ChPk         DEEMLCSKC-------------RKICRHCRCS----REDH---------------CLLPPTIQEKTYSIS
TaPk         DTGRPCLKCR-QKCPGFCHHSWRKICRHCKCS----KSEH---------------DILADDHERFVEKMI
MmTes        KPPWANEG------EGFELHFWRKICRNCNVV----KKSMTVLL----------------SNEEDRKVGR
DmTes        GAGAPCAECK-DKCPGLDLHFWRKVCRNCKCP----KIQHVCPD----------------DDD-------
ChTes        GHGAECLKCG-PKCEGLDLHFWRKICKNCYCR----YDEHDVKS----------------EEEIHHGIVK
OcPkTesL     DAGAPCMKCG-DGCIGFELHYWRKICKNCRCK----REEH----------------------DIKVEEDA
OlPkTesL     DAGAPCLKCG-DACVGFELHYWRKICKNCRCK----REEH----------------------DIKAEENA
OspPkTesL    DAGAPCVKCG-DNCIGFELHFWRKICKNCHCK----REEH----------------------DIKAEEDA
ScPkTesL     EKGEPCKTCGPHICPGFQLHFWRKVCQYCRCP----ESEH----------------MSAKTLDNMEEATR
AqPkTesL     DEGSPCLRC--DNCEKAS-CFARKICANCQCP----REEH----------------------DIRPHEER
EmPkTesL     DEGTPCLVCG-DKCPGFSLHFWRKVCKNCLCP----REEH----------------------DVKEDNKE
AvPkTesL     DAGRPCIKCG-VKCPGFSLHFWRKICLHCKCP----RMDH----------------------DIQEYESH
OmPkTesL     DAGRPCMKCG-DKCPGFALHFWRKVCRHCKCP----WMEH----------------------DIHLIDEL
MlPkTesL     DYSE----------------------------DSSDSESH--------QQAAPPKPLRRNPGSFDDLTQG
SrPkTesL     DKGQPCFSCD-N-CPGFEMHFWRKTCTKCGCP----KSSHGFKELPTRSVGKLSGYDMYQQGTPNEDVRA

                   500       510       520       530       540       550       560
             ....|....|....|....|....|....|....|....|....|....|....|....|....|....|
MmPkL1       --------------------------------LVFGCQRSSTSDDDSGCALEEY----------------
MmPkL2       --------------------------------LMFDFQRSSTSDDDSGCALEEY----------------
MmPkL3       --------------------------------LISDFQRHSISDDDSGCASEEY----------------
DmPk         SPALSTPSPPSLLHHPAGGTSSASAHAPFLGGPHMDMQRQSHSDDDSGCALEEY----------------
DmEsn        ---------------------------------QLDFQRNSQSDDDSGCALEEY----------------
ChPk         -------------------------------KFADQHPSSMGNDGDSGCSTEEY----------------
TaPk         ---------------------------------FEYYKVNGLSDFNINPAIEEY----------------
MmTes        L------------------------------FEDTKYTTLIAKLSKDGIPMYKRNVMILT----------
DmTes        ---------------------------------ATGWAQFEILGQIRAKPAYIK----------------
ChTes        N------------------------------IFRKERGLSDEIGKLQIFDPEKQ----------------
OcPkTesL     --------------------------------ETKRMIKNLFSDNPSPAVKKKNE---------------
OlPkTesL     --------------------------------ETKRMIKNLFSDNPSPVVPRKTE---------------
OspPkTesL    --------------------------------ETKRMIRSLFSDNPSPIVQRKTE---------------
ScPkTesL     L-------------------------------TLNGLFKVDNYDEMSGSTQVDNVSKSTSGTQVLSTLD-
AqPkTesL     --------------------------------QAA-VGKLLFHSNSDAKAMVKDAASKSPQRPRATIPHI
EmPkTesL     --------------------------------GMS-VGKLLFAPTAELAKGSSIDISPSP-KLRRPLTKE
AvPkTesL     --------------------------------RTN-FEIIYFPQDLNPG---------------------
OmPkTesL     --------------------------------GTK-VQNYYFSLD-------------------------
MlPkTesL     -------------------------------DIVDYYTTVSPSGEYPGSATLQRK---------------
SrPkTesL     --------------------------------RVRRIREQRGDQDVDGEMTVEDVEEAVRQLNNENAVDD
```

```
                  570       580       590       600       610       620       630
          ....|....|....|....|....|....|....|....|....|....|....|....|....|....|
MmPkL1    -----------------AWVPPGLRPEQIQLYFACLPEEKVPYVNSPGEKHRIKQLLYQLPPHDNEVRYC
MmPkL2    -----------------AWVPPGLKPEQVHQYYSCLPEEKVPYVNSAGEKLRIKQLLHQLPPHDNEVRYC
MmPkL3    -----------------AWVPPGLKPEQVYQFFSCLPEDKVPYVNSPGEKYRIKQLLHQLPPHDSEAQYC
DmPk      -----------------TWVPPGLRPDQVRLYFSQIPDDKVPYVNSPGEQYRVRQLLHQLPPHDNEVRYC
DmEsn     -----------------TWVPPGLRPDQVRLYFSQLPDDKVPYVNSPGEKYRVKQLLHQLPPQDNEVRYC
ChPk      -----------------AWVPPGVSGELVNAYMNSLHEDKIPFVNSIGENYRAKQLLYQLPPHDSDSKHC
TaPk      -----------------AWTPPALSIRQIEAYFDCLPKENVPIIDSVGDRYRIQQLLYQHPAHDYDPKFC
MmTes     -NPVAAKKNVSINTVTYEWAPPVQNQALARQYMQMLPKEKQPVAGSEGAQYRKKQLAKQLPAHDQDPSKC
DmTes     ------IKALASQPVQLEWVPPNAAPDVVTDYMEKLGTAQIPVAGSDAALKRKMQLELQVPPHDLDAALC
ChTes     ------PAIAEQVKANFIKVPEVSSPFAMSKYLKQLPKEKDAFQGEAGQQYRNHQLQQQLPAHDFDPAFC
OcPkTesL  -------KDMMELNARYAWAP-DVEKDLAVKYMESLPEEKVPLKGTDGHKYRRDQLINQLPVHDNDASKC
OlPkTesL  -------QETIELDARFAWTP-NVGKDLAVKYMEALPEDKVPVKGTDGQKYRRDQLFNQLPVHDNDVTQC
OspPkTesL -------QEKIELDARFAWTP-DVEKDLAVKYMESLPEDKVPTKGTDGQKYRRDQLFNQLPVHDNDASRC
ScPkTesL  GSRKG-SSAQVSNCEQLKKIKTGTTKNLVAKFMENIPE----EEKEEGATYRQKQLLMQLPAHDIDGKAC
AqPkTesL  PKQESESPSILRRTENFTWTPRDTTVEAARSFFANLPQNKKPVAGMAGDRYWQKQKIRQLPAHDIDLIYC
EmPkTesL  PSQDS--PASVKKMNLYSWVVKGATPEAMEKFVESLPETKRPMKGTAGERYHHKQLIRQLPAHDIAITYC
AvPkTesL  -----------DKFDLLNWIPKGITKQLIDAYLSSLPKDKNPLE-PRGRIYRNQQFVYQLPKHDTFVIEH
OmPkTesL  -----------EKYDHMNWIPKGLTKQLIEVYLNSLPQDKNPSR-PCGINYRHRQLIYQLPEHDTFLYER
MlPkTesL  -------------GSPGVWVPPGIDHNLVKQYLAALPEDKRP-VNTLGVQWRIKQLQTQLPRYDHNINYC
SrPkTesL  GGDNN-DNDDGHDGGDDDDMETGELMGFEAPELTDLPE----MESFEDVSDDDDDDGDDGDDGDDDGDGD

                  640       650       660       670       680       690       700
          ....|....|....|....|....|....|....|....|....|....|....|....|....|....|
MmPkL1    Q--SLSEEEKKELQVFSA----------------------------------------------------
MmPkL2    N--SLDEEEKRELKLFSN----------------------------------------------------
MmPkL3    T--ALEEEEKKELRAFSQ----------------------------------------------------
DmPk      H--SLTDEERKELRLFST----------------------------------------------------
DmEsn     H--SLSDEERKELRIFSA----------------------------------------------------
ChPk      H--NLSEEEKRELRSFHG----------------------------------------------------
TaPk      Y--RLSEEEKKKHRQFSA----------------------------------------------------
MmTes     H--ELSPKEVKEMEQFVKKYKSEALG--------------------------------------------
DmTes     D--GLTETEAIQLQQYVQKLREQCVGQGVVVRLGDRLNHAQVEHVAPALMPTQEAQQTWQSLGLMPVADD
ChTes     N--KLSDQEKDRMQKFTD-VRDDDAG--------------------------------------------
OcPkTesL  D--NLTPAERELMEEFVE----------------------------------------------------
OlPkTesL  D--NLTDAEREQMHEFVL----------------------------------------------------
OspPkTesL D--NLNDAEREQMEEFVL----------------------------------------------------
ScPkTesL  K-TLMTTEEKQEMEEFR-----------------------------------------------------
AqPkTesL  N--ELTDEECKQMELFIK----------------------------------------------------
EmPkTesL  NEEDLSPEDCKQMEIFHK----------------------------------------------------
AvPkTesL  NKCNLSENEFEQVRAFIQ----------------------------------------------------
OmPkTesL  NREKLTDIEFQQVRAFIQ----------------------------------------------------
MlPkTesL  H--EMSPEEQEELFMFVE----------------------------------------------------
SrPkTesL  D-VQGQEGEEKEKEEEEEVKAEEPEPEPEPEPEPPKGPSDAILKKYTWVPDGLCDDDIERYFKAFPRDIV
```

```
                   710       720       730       740       750       760       770
           ....|....|....|....|....|....|....|....|....|....|....|....|....|....|
MmPkL1     ----------------------------------------------------------------------
MmPkL2     ----------------------------------------------------------------------
MmPkL3     ----------------------------------------------------------------------
DmPk       ----------------------------------------------------------------------
DmEsn      ----------------------------------------------------------------------
ChPk       ----------------------------------------------------------------------
TaPk       ----------------------------------------------------------------------
MmTes      ----------------------------------------------------------------------
DmTes      TLNELLANPKVAQALASPASAHPKLLVAFSEPLCESTAQFEENGALRAQTREKLLGISKPALLSLVTHGI
ChTes      ----------------------------------------------------------------------
OcPkTesL   ----------------------------------------------------------------------
OlPkTesL   ----------------------------------------------------------------------
OspPkTesL  ----------------------------------------------------------------------
ScPkTesL   ----------------------------------------------------------------------
AqPkTesL   ----------------------------------------------------------------------
EmPkTesL   ----------------------------------------------------------------------
AvPkTesL   ----------------------------------------------------------------------
OmPkTesL   ----------------------------------------------------------------------
MlPkTesL   ----------------------------------------------------------------------
SrPkTesL   PLRGSPGAGWRLRQLEI-----------------------------------------------------

                   780       790       800       810       820       830       840
           ....|....|....|....|....|....|....|....|....|....|....|....|....|....|
MmPkL1     ------------------------------QRKKEALGRGTIKLLS------------------------
MmPkL2     ------------------------------QRKRENLGRGNVRPFP------------------------
MmPkL3     ------------------------------QRKRENLGRATVRIFP------------------------
DmPk       ------------------------------QRKRDALGRGNVRQLM------------------------
DmEsn      ------------------------------QRKREALGRGAVRLLS------------------------
ChPk       ------------------------------RRKKDCLGRGNVRLFPNV----------------------
TaPk       ------------------------------TRKKDAFGQATVRTIG------------------------
MmTes      --------------------------------VGDVKFPSEMNAQGDK----------------------
DmTes      VYDKVLGILQEKKLNISRDPKLGPIAEFRKEYVNNPQFRAEINTICPQPPMTPIKSPPGTPFNSPLPLKN
ChTes      --------------------------------------QGAIKEKTAK----------------------
OcPkTesL   ------------------------------KRKKTAVGRASVKDKKATK---------------------
OlPkTesL   ------------------------------KRKKTAIGRASVKDRRPMM---------------------
OspPkTesL  ------------------------------KRKKTAIGRASVKDRSAAR---------------------
ScPkTesL   -----------------------------QHRQEHALGQGLMYTIGQKRDMTDEHKS-------------
AqPkTesL   ------------------------------IRREKFLGRGMIKASDSS----------------------
EmPkTesL   ------------------------------IRREKVAGRGEVKLREGSN---------------------
AvPkTesL   ------------------------------LRKERIS-RATIRANFTE----------------------
OmPkTesL   ------------------------------LRKDRIS-RAKIRANLSD----------------------
MlPkTesL   ------------------------------SRKEKAQGQGEVKSFK------------------------
SrPkTesL   --------------------------QLPPHDVDKALCSGLKHHDYPAYDAFNADKIAYAC---------
```

```
                  850       860       870       880       890       900       910
             ....|....|....|....|....|....|....|....|....|....|....|....|....|....|
MmPkL1       ----------------------------------------------------------------------
MmPkL2       ----------------------------------------------------------------------
MmPkL3       ----------------------------------------------------------------------
DmPk         ----------------------------------------------------------------------
DmEsn        ----------------------------------------------------------------------
ChPk         ----------------------------------------------------------------------
TaPk         ----------------------------------------------------------------------
MmTes        ---------------------------VHNCGN------------------------------RHAPAAV
DmTes        PVQIRMGAQMRQDTPMRRVKFGGVSTIVYDCGLPANTDYDRDPVFAQILQAEPLKHAFQEARAGRAPSSV
ChTes        ----------------------------------------------------------------------
OcPkTesL     ----------------------------------------------------------------------
OlPkTesL     ----------------------------------------------------------------------
OspPkTesL    ----------------------------------------------------------------------
ScPkTesL     ----------------------------------------------------------------------
AqPkTesL     ----------------------------------------------------------------------
EmPkTesL     ----------------------------------------------------------------------
AvPkTesL     ----------------------------------------------------------------------
OmPkTesL     ----------------------------------------------------------------------
MlPkTesL     ----------------------------------------------------------------------
SrPkTesL     ----------------------------------------------------------------------

                  920       930       940       950       960       970       980
             ....|....|....|....|....|....|....|....|....|....|....|....|....|....|
MmPkL1       ----------------------------------------------------------------------
MmPkL2       ----------------------------------------------------------------------
MmPkL3       ----------------------------------------------------------------------
DmPk         ----------------------------------------------------------------------
DmEsn        ----------------------------------------------------------------------
ChPk         ----------------------------------------------------------------------
TaPk         ----------------------------------------------------------------------
MmTes        ASK-------------------------------------------------------------------
DmTes        VISNIPAPVASLAELRGLNPATRAQLQSVGLDKNMLQSAVSNAPYYDRLFRSLHDKGISHDQCHLLQPMK
ChTes        ----------------------------------------------------------------------
OcPkTesL     ----------------------------------------------------------------------
OlPkTesL     ----------------------------------------------------------------------
OspPkTesL    ----------------------------------------------------------------------
ScPkTesL     ----------------------------------------------------------------------
AqPkTesL     ----------------------------------------------------------------------
EmPkTesL     ----------------------------------------------------------------------
AvPkTesL     ----------------------------------------------------------------------
OmPkTesL     ----------------------------------------------------------------------
MlPkTesL     ----------------------------------------------------------------------
SrPkTesL     ----------------------------------------------------------------------
```

```
                    990       1000      1010      1020      1030      1040      1050
              ....|....|....|....|....|....|....|....|....|....|....|....|....|....|
MmPkL1        ----------------------------------------------------------------------
MmPkL2        ----------------------------------------------------------------------
MmPkL3        ----------------------------------------------------------------------
DmPk          ----------------------------------------------------------------------
DmEsn         ----------------------------------------------------------------------
ChPk          ----------------------------------------------------------------------
TaPk          ----------------------------------------------------------------------
MmTes         ----------------------------------------------------------------------
DmTes         QVHDWLLDDDQLLDEIDKVFADMANGCKDISYPKPSLGELPTSQSSDSGFHSKPPTPGYGSEDLTAGQGR
ChTes         ----------------------------------------------------------------------
OcPkTesL      ----------------------------------------------------------------------
OlPkTesL      ----------------------------------------------------------------------
OspPkTesL     ----------------------------------------------------------------------
ScPkTesL      ----------------------------------------------------------------------
AqPkTesL      ----------------------------------------------------------------------
EmPkTesL      ----------------------------------------------------------------------
AvPkTesL      ----------------------------------------------------------------------
OmPkTesL      ----------------------------------------------------------------------
MlPkTesL      ----------------------------------------------------------------------
SrPkTesL      ----------------------------------------------------------------------

                    1060      1070      1080      1090      1100      1110      1120
              ....|....|....|....|....|....|....|....|....|....|....|....|....|....|
MmPkL1        ------------------------------------RAVMHAVCEQCGLQMNGGEVAVFASRAG-----P
MmPkL2        ------------------------------------VTMTGAICEQCGGQIKGGDIAVFASRAG-----H
MmPkL3        ------------------------------------VTITGAICEECGKQIGGGDIAVFASRAG-----L
DmPk          ------------------------------------SARP---CDGCDDLISTGDIAVFATRLG-----P
DmEsn         ------------------------------------DERP---CKGCEEPLSGGDIVVFAQRLG-----A
ChPk          ------------------------------------AEGSSGVCQQCSKRIVPGEVVVHAWRAG-----K
TaPk          ------------------------------------SRMNGHECAECGEQLNHGELGLFASHAVP----E
MmTes         -------------------------------DKSAESKKTQYSCYCCKHTTNEGEPAIYAERAGY-----
DmTes         FASIPGIEDMNMYPSCAGMPEQFQQLRLHGDEASGKNSTRTILCADCNQPIAMGEVAVKADRAGK-----
ChTes         -------------------------------------EKTHWKCEKCGENLMIGEVAIFAEKAGK-----
OcPkTesL      -------------------------------------ESK-WKCSECNETIESGSCAVFADKAAD-----
OlPkTesL      -------------------------------------ESDGWTCAECRKGLESGSVAVFADRAGDD----
OspPkTesL     -------------------------------------ESSGWVCEECRKPLESGSCAVFADRAGEN----
ScPkTesL      -------------------------------------VEEVWDCQECTDTLKKGETAIFTDKTGGER---
AqPkTesL      -----------------------------------------CVCKRCCVTFKKGESAVIADKLSGSD---
EmPkTesL      -------------------------------------LSTQWVCVSCKIPMKTGDVAVFAERAKG-----
AvPkTesL      ---------------------------------------LAWNCSGCTKPLKIGDAAIMLDESAR-S---
OmPkTesL      ---------------------------------------LALNCSCCDHSLKIGDAAIMVDEIP--P---
MlPkTesL      ------------------------------------KKSKKK-CDKCGGLVDCAVIADKIP---------
SrPkTesL      ------------------------------DSGVAVVIDSPRICFRCGQGLFPGDLAVTTERIVDETGQT
```

```
              1130      1140      1150      1160      1170      1180      1190
          ....|....|....|....|....|....|....|....|....|....|....|....|....|....|
MmPkL1     GVCWHPSCFVCFTCNELLVDLIYFYQDG--KIHCGRHHAE-LLKPRCSACDEIIFADECTEAEGRHWHMK
MmPkL2     GICWHPPCFVCTVCNELLVDLIYFYQDG--KIYCGRHHAE-CLKPRCAACDEIIFADECTEAEGRHWHMR
MmPkL3     GACWHPQCFVCTTCQELLVDLIYFYHAG--KVYCGRHHAE-CLRPRCQACDEIIFSPECTEAEGRHWHMG
DmPk       NASWHPACFACSVCRELLVDLIYFHRDG--RMYCGRHHAE-TLKPRCSACDEIILADECTEAEGRAWHMN
DmEsn      QLCWHPGCFVCSVCKELLVDLIYFQRDG--NLYCGRHHAE-TQKPRCSACDEIIFSDECTEAEGRTWHMK
ChPk       EACWHPACFQCTTCQELLVDLVYFYQEG--RVYCGRHHAE-LLKPRCSACDEIIFSDECTEAEGRFWHLG
TaPk       NLVWHPECFICCVCENGLVDLIYYYKDG--EVYCGRHHAD-SVKPRCNACDEIIFTEECIQAHGRTWHTD
MmTes      DKLWHPACFICSTCGELLVDMIYFWKNG--KLYCGRHYCD-SEKPRCAGCDELIFSNEYTQAENQNWHLK
DmTes      EIAWHPGCFKCITCRELLADLVYFFHQG--QVFCGRDLAIRLKIPRCRACDELIFTKEYTAAEEATFHIK
ChTes      DKCWHPNCFVCDTCQELLVDLIYVFKDG--KIFCGRHYGE-ITRVRCAACDELIFTKEYTQAEDQNWHLQ
OcPkTesL   -MCWHPKCFVCCKCKELLVDLIYFWKDE--HLYCGRHHAE-LVKPRCAACDELIFSKEYTRAEDKNWHLR
OlPkTesL   -RCWHPGCFVCSKCKELLVDLIYFWHGD--KLYCGRHHAE-LVKPRCAACDELIFSKEYTRAEDKNWHLR
OspPkTesL  -ACWHPGCFVCSQCKELLVDLIYFWRDG--KLYCGRHHAE-LVKPRCAACDELIFSKEYTHAEDKNWHLR
ScPkTesL   --CWHAKCFVCFKCKELLVDLIYFHKDD--HVYCGRHHAE-LVKPRCKACDELIFTGEYTHAEDADWHIV
AqPkTesL   -NMYHPACFTCTDCNELLIELIYFVYED--KLYCGRHHSE-KMKPRCAACDEMIFCEEYTRAEDQNWHVN
EmPkTesL   -MCWHPACFTCSDCNELLAQLIYFWDEKEHKIYCGRHHAE-IMKPRCAACDELIVCPEFTRAEEQNWHLN
AvPkTesL   -IFFHAHCFICSDCKELLVDLIYFYSNG--KLYCGRHHAE-TIKPRCAACDELIFSKEFTKAEGLNWHLQ
OmPkTesL   -IFFHPHCFICNICKELLVDLIYFISNN--KLYCGRHHAE-TLKPRCAACDELIFSKEFTRAEGLNWHLH
MlPkTesL   GRVWHPGCFSCTTCEEVLVDLIYFQYDG--RLYCGRHHAE-LLRPRCHACDELIFGQEFITADKHEYHKE
SrPkTesL   AVYHHDTCFVCEACDSPLADLFCFVTPEE-QLVCGRHYAD-LYRPRCHACDETIFDQDYAFAEEHNWHRE

              1200      1210      1220      1230      1240      1250      1260
          ....|....|....|....|....|....|....|....|....|....|....|....|....|....|
MmPkL1     HFCCLECETVLGGQ-RYIMKD--GRPFCCGCFESLYAEYCETCGEHIGVDHAQMT---YDGQHWHATEAC
MmPkL2     HFCCFECETVLGGQ-RYIMKE--GRPYCCHCFESLYAEYCDTCAQHIGIDQGQMT---YDGQHWHATETC
MmPkL3     HFCCFECEASLGGQ-RYVMRQ--SRPHCCACYEARHAEYCDGCGEHIGLDQGQMA---YEGQHWHASDRC
DmPk       HFACHECDKQLGGQ-RYIMRE--GKPYCLHCFDAMFAEYCDYCGEAIGVDQGQMS---HDGQHWHATDEC
DmEsn      HFACQECEHQLGGQ-RYIMRE--GKPYCLACFDTMFAEYCDYCGEVIGVDQGQMS---HDGQHWHATDQC
ChPk       HFACYECDSSLGGQ-RYVMRD--NHPICCVCFEKMFAEFCDSCGEPIGIDVGQMA---HGSQHWHANEKC
TaPk       HFVCYECECRLGSRNQYIMRD--GQPYCCRCFESLYAVYCESCGEMIELNDGHMA---HNDMHWHASDDC
MmTes      HFCCFDCDHILAGK-IYVMVT--DKPVCKPCYVKNHAVVCQGCHNAIDPEVQRVT---YNNFSWHASTEC
DmTes      HFCCYQCDEPLAGQ-QYIADEKSNMPLCLLCYDRLFAVRCQRCKVAIGPADQGVA---WGDVHWHASCFV
ChTes      HFCCLKCDKMLGGQ-KYVARD--GKPYCMGCYDTTFAKTCQTCKQRIAADAKRVS---YKDANWHASEEC
OcPkTesL   HFCCFECDAQLGGK-RYISQE--SHPYCLECFDKRFSKKCQSCGKPIPADTPHLT---HGKFSWHGMDDC
OlPkTesL   HFCCFECDVQLGGK-RYVSHE--NHPYCLDCFDRRFSKVCQSCGNAIPADAPHLT---HGEQNWHGTEQC
OspPkTesL  HFCCFECDTQLGGK-RYVSHD--GHPYCLECFDRRFSKLCQRCGKPIPADKPHLT---HGNYSWHGTEEC
ScPkTesL   HFCCWRCDELLGGK-RYISRE--GHPFCLGCFDKEFGKKCNTCNKAIAADESHLT---HKDMTWHGNDEC
AqPkTesL   HFCCHRCDSYLGGQ-TYMAKE--TQPYCLKCYELLFANICTACGSTISLDEPVLK---HEGKYWHARAEC
EmPkTesL   HFCCLRCDAGLAGK-EYRPQD--GKPYCIQCYDLVFSTVCETCGMTISLDQPRLV---HNNVTWHGDSKC
AvPkTesL   HFCCIDCDTLLGGK-KYMVADN-GHPYCLICFANLYSKGCATCGNFIRVDDPRLS---HGEFDWHGSPLC
OmPkTesL   HFCCIECDSLLGGQ-KYMVADN-GHPYCLLCFSRLFSKGCSTCGSFIGVDDPRLS---HGDFDWHGTSVC
MlPkTesL   HFCCWECDTGLGGA-KYVSHK--GQPYCQGCYEKLFCTRCVTCREPIGAGAPLFK---HGALRWHGNPQC
SrPkTesL   HFCCYRCDAHLIGK-DYIALS--GEPVCLDCFGDEFAERCAACHQPIGVNEQKVTDGKRRGKVWHRS--C
```

```
                   1270      1280      1290      1300      1310      1320      1330
           ....|....|....|....|....|....|....|....|....|....|....|....|....|....|
MmPkL1     FSCAQCKASLLGCPFLPKQGQIYCSKTCSLGEDIHASDSSDSAFQSARSRDSRRSVRMGRSSRSADQCRQ
MmPkL2     FCCAHCKKSLLGRPFLPKQGQIFCSRACSAGEDPNGSDSSDSAFQNARAKESRRSAKIGKNKGKTEEA--
MmPkL3     FCCSRCSRPLLGRPFLPRRGLIFCSRACSLGSETTAPGPG------------RRSWSAG-----------
DmPk       FSCNTCRCSLLGRAFLPRRGAIYCSIACSKGEPPTPSDSSGTGMYTTPTPPTQRVRPHPQAPLPARIPSS
DmEsn      FSCCTCRCSLLGRPFLPRRGTIYCSIACSKGEPPTPSDTSSG----PQLRPTHRASTSSQIAKSPRRGGE
ChPk       FSCFNCGQTLLGQPFLPKNGEIFCSSGCSRGIPPPNPVNPKYPPRSASRNRSYRPSSESSNNNSRDGYVS
TaPk       FSCSECNQSLLGKTFLPKHGKLYCSVACFKIGRMPSEYGGSEESLVEYSDCDINHPQPPTSSVKDIGVGI
MmTes      FLCSCCSKCLIGQKFMPVEGMVFCSVECKRMMS-------------------------------------
DmTes      CAGVQCSKPLIGGRFCVKENMPFCSPTCVRSLIN------------------------------------
ChTes      FRCLACSEPMLGKQFIYKNKDVYCSGACARK---------------------------------------
OcPkTesL   FNCSQCSKPLVGQKFLPREEKIFCSKDCARKAKKNEA---------------------------------
OlPkTesL   FRCSQCSKSLVGQKFLPKNGKIFCSKACWKKSNDR-----------------------------------
OspPkTesL  FKCSQCSKSLVGQKFLPKNDKIFCSKACARKSIGQ-----------------------------------
ScPkTesL   FTCHTCKKSLVGKPFLPRLDHIFCCRACYKVYKEAN-MELAGTPEVK-----------------------
AqPkTesL   FKCRTCKKVLVDQPFLPKSSKIFCSKECFREYRDNSSQRPFISRDQSAPPSF------------------
EmPkTesL   FKCSHCGKSLVGKPFLPKNRKVYCSKTCAREDKDSSK---------------------------------
AvPkTesL   FKCHYCSTSLLGSPFLPKGSSLYCSKICYKS---RKN---------------------------------
OmPkTesL   FRCNFCCVSLLGSPFIPKGNYLYCSKDCYKSDKFRKTL--------------------------------
MlPkTesL   YACSFCKTSLVNRTFMPTERYVYCSKNCYRCHESLRKTKGK-----------------------------
SrPkTesL   FVCAACHQELHGRVCVPRDGRLYCREDYDRIFKKIARKSMRSKPRVRAALGESDIWSMAQDF--------

                   1340      1350      1360      1370      1380      1390      1400
           ....|....|....|....|....|....|....|....|....|....|....|....|....|....|
MmPkL1     SLLLSPALNYKFPGLSGNADDTLSRKLDDVSLASRQGAGFANEEFWKARVEQEASEDPEEWAEHEDYMTQ
MmPkL2     MLNQHSQLQVSSNRLSADVDP-LSVQMDLLSLSS-QTPSLNRDPIWRSREEPFHYGNKMEQNQSQSPL-Q
MmPkL3     ----------------------------------------------------------------------
DmPk       HASSSPPMSPQQQQQHQATFNQAMYQMQSQQMEAAGGLVDQSKSYAASDSDAGVVKDLEHGGHMGGGD-L
DmEsn      RERDPGRKAHHGHPKATGSAGDLLERQERQRMEAAG------------------VADLLLGGGVPG----
ChPk       SSTMSPEPVRKIVEIRSKASYRSSLDKYGLAAAEKIGDIVRN----------------------------
TaPk       RGQLNVIQNSSERKLKNSQTNRKYWNRHEQDHIGSSE---------------------------------
MmTes      ----------------------------------------------------------------------
DmTes      ----------------------------------------------------------------------
ChTes      ----------------------------------------------------------------------
OcPkTesL   ----------------------------------------------------------------------
OlPkTesL   ----------------------------------------------------------------------
OspPkTesL  ----------------------------------------------------------------------
ScPkTesL   ----------------------------------------------------------------------
AqPkTesL   ----------------------------------------------------------------------
EmPkTesL   ----------------------------------------------------------------------
AvPkTesL   ----------------------------------------------------------------------
OmPkTesL   ----------------------------------------------------------------------
MlPkTesL   ----------------------------------------------------------------------
SrPkTesL   ----------------------------------------------------------------------
```

```
                  1410      1420      1430      1440      1450      1460      1470
          ....|....|....|....|....|....|....|....|....|....|....|....|....|....|
MmPkL1    LLLKFGDKNLFQQ--QSSEVDPRASEHWIPDNMVTNKPEVKPN----HQGLASKKYQSDMYWAQSQDGLG
MmPkL2    LLSQCNIRTSYSPGGQGAGAQPDMWAKHFSNPKRSSSMALKGHGGSFIQECREDYYPGRLMSQESYSDMS
MmPkL3    ---------------------------TVTTPLTTS----------------------------------
DmPk      TDFSGGRASSTSQNLSPLNSPGDFQPHFLPKPMELQRDGV----------------------------YN
DmEsn     ----------------------------MPRPAHPPP-------------------------------ID
ChPk      ----------------------------MPNGGVIKEESD----------------------------EK
TaPk      -------------------------------------------------------------------VIP
MmTes     ----------------------------------------------------------------------
DmTes     ----------------------------------------------------------------------
ChTes     ----------------------------------------------------------------------
OcPkTesL  ----------------------------------------------------------------------
OlPkTesL  ----------------------------------------------------------------------
OspPkTesL ----------------------------------------------------------------------
ScPkTesL  ----------------------------------------------------------------------
AqPkTesL  ----------------------------------------------------------------------
EmPkTesL  ----------------------------------------------------------------------
AvPkTesL  ----------------------------------------------------------------------
OmPkTesL  ----------------------------------------------------------------------
MlPkTesL  ----------------------------------------------------------------------
SrPkTesL  ----------------------------------------------------------------------

                  1480      1490      1500      1510      1520      1530      1540
          ....|....|....|....|....|....|....|....|....|....|....|....|....|....|
MmPkL1    DSAYGSHPGPASSRRLQELDLD-HGAAGYTHDQSQWYEDSLECLSDLKP-EQSIRDSMDSLALSNITGAS
MmPkL2    SQSFNETRGSIPVPKYEEEEEEEEGGISTQQCRPRRPLSSLKYTEDMTPTEQTPRGSMESLALSNATGLS
MmPkL3    TASFSATEG-------------------------------------------------TSETASKGTCTK
DmPk      FNEMSSNLDAAWSAKPTNSYHLQRQLLENPHTASMPELAGKLVAPPAHMQHLSQLHAVSSHQFQQHEYAD
DmEsn     LTELGISLDNICAGD--KSIFGDTQTLTN----SMPDML--------------LSKADDSHSYQSIDKIN
ChPk      DETSSTCSSVASSRAKELPSFPPSNRHSKKPILRVPPKQRPKPTGPEIWIDMVPPKEATTHRKQEEDWEK
TaPk      ESFTGASSGLAYSRS----------------------------------------QKLNSYRSRDNNAEF
MmTes     ----------------------------------------------------------------------
DmTes     ----------------------------------------------------------------------
ChTes     ----------------------------------------------------------------------
OcPkTesL  ----------------------------------------------------------------------
OlPkTesL  ----------------------------------------------------------------------
OspPkTesL ----------------------------------------------------------------------
ScPkTesL  ----------------------------------------------------------------------
AqPkTesL  ----------------------------------------------------------------------
EmPkTesL  ----------------------------------------------------------------------
AvPkTesL  ----------------------------------------------------------------------
OmPkTesL  ----------------------------------------------------------------------
MlPkTesL  ----------------------------------------------------------------------
SrPkTesL  ----------------------------------------------------------------------
```

```
                   1550      1560      1570      1580      1590      1600      1610
           ....|....|....|....|....|....|....|....|....|....|....|....|....|....|
MmPkL1     VDGESKPR--PSLYSLQNFEEIEAEDC-EKMSNMGTLNSSMLHRSAESLQSLNSGLCPEKILPEEKPAHL
MmPkL2     AEGGAKRQEHLSRFSMPDLSKDSGMNVSEKLSNMGTLNSSMQFRSAESVRSLLSAQQYQEMEGNLHQLSN
MmPkL3     AEPAAGPE-----------------------------EPSHFLRGAPHRHSMPELGLRSAPEPPTESPGH
DmPk       ILHPPPPP--PGEIPELPTPNLSVASTALPPELMGSPTHSAGDRSLNTPMSTQSASHAPPHPVSILSGAS
DmEsn      LNSPS-------------NSDLTQSTQELANELELD-NEPVRELPHDGYEQLFANNRNQEHPAEQYD---
ChPk       QSVRSMRS------------NRSTTSTKKYGDAMSHLNGNDTIERLVQGERQINRRHRRRKVDDSYFSDY
TaPk       SKNNSNIR-----------------------------RSRSRSNGHDSAQSNPNNQIEVETASANDHYNY
MmTes      ----------------------------------------------------------------------
DmTes      ----------------------------------------------------------------------
ChTes      ----------------------------------------------------------------------
OcPkTesL   ----------------------------------------------------------------------
OlPkTesL   ----------------------------------------------------------------------
OspPkTesL  ----------------------------------------------------------------------
ScPkTesL   ----------------------------------------------------------------------
AqPkTesL   ----------------------------------------------------------------------
EmPkTesL   ----------------------------------------------------------------------
AvPkTesL   ----------------------------------------------------------------------
OmPkTesL   ----------------------------------------------------------------------
MlPkTesL   ----------------------------------------------------------------------
SrPkTesL   ----------------------------------------------------------------------

                   1620      1630      1640      1650      1660      1670      1680
           ....|....|....|....|....|....|....|....|....|....|....|....|....|....|
MmPkL1     PV-LRRSKSQSRPQQ--VKFSDDVIDNGSYDIE-IRQPPMSERTRRRAYHFEERGSRPHHHRHRRSRKSR
MmPkL2     PLGYRDLQSHGRMHQS-FDFDGGIASSKLPGQEGVHIQPMSERTRRRTTSRDD-NRRFRPHRSRRSRRSR
MmPkL3     PAPHPDDNAFGRQSTPRVSFRDPLVSEGGPRRT-LSAPPAQRRRPRSPPPRTPSCHHHHHHRRRRQRHRR
DmPk       SSSPMSGEPAKKKGVRFEGIPDTLPRSRSYSGNGAGTSGGGERERDRDKDKEGGGRHGHGHSSRRRRRRK
DmEsn      -DEQLDNRPMK--EVRFHSVQDTMSRSKSYTDNSNARR-------------------------RRRRRNQ
ChPk       EVERRKRTQRRKQTPLVYQMDDEKPNIPLMTVKSRSTESLDAGKARVAGRRDE----------RDGKKKR
TaPk       NNPTKNPQHRKSPKQKKYTKNETMALEGPSSELKFKKYSSIDRIGEGIAYAQP-----------LDRIRE
MmTes      ----------------------------------------------------------------------
DmTes      ----------------------------------------------------------------------
ChTes      ----------------------------------------------------------------------
OcPkTesL   ----------------------------------------------------------------------
OlPkTesL   ----------------------------------------------------------------------
OspPkTesL  ----------------------------------------------------------------------
ScPkTesL   ----------------------------------------------------------------------
AqPkTesL   ----------------------------------------------------------------------
EmPkTesL   ----------------------------------------------------------------------
AvPkTesL   ----------------------------------------------------------------------
OmPkTesL   ----------------------------------------------------------------------
MlPkTesL   ----------------------------------------------------------------------
SrPkTesL   ----------------------------------------------------------------------
```

```
                      1690      1700      1710      1720      1730      1740      1750
              ....|....|....|....|....|....|....|....|....|....|....|....|....|....|
MmPkL1        SDNALNLVTERKYSA--KDRLRLYTPDNYEKFIQNKSARELQAYMQNANLYSQYAHATSDYALQNPGMNR
MmPkL2        SDNALHLASEREVIARLKERPPLRAREDYDQFMRQRSFQESLGQGSRRDLYSQCPRTVSDLALQNAFGER
MmPkL3        RG----------------------SHHHHHHPGRHGHHRCDLGSGSDSGSCSSSP---------------
DmPk          SSSSSSHHRSGSGHR----------SHSTTRADTYAPAQPLSSSYQGPPSVLQAANLVHESPSRQQRERE
DmEsn         SRSSSEMQINQTNLR----------LHN---AQTQVGTTPLNLLNN------------------------
ChPk          TKSETNLTNSKNYKS---------KNDAVANLRLNIDVNSPKKQSYN-----------------------
TaPk          ADQSILVAKGYKKSE-------------------------------------------------------
MmTes         ----------------------------------------------------------------------
DmTes         ----------------------------------------------------------------------
ChTes         ----------------------------------------------------------------------
OcPkTesL      ----------------------------------------------------------------------
OlPkTesL      ----------------------------------------------------------------------
OspPkTesL     ----------------------------------------------------------------------
ScPkTesL      ----------------------------------------------------------------------
AqPkTesL      ----------------------------------------------------------------------
EmPkTesL      ----------------------------------------------------------------------
AvPkTesL      ----------------------------------------------------------------------
OmPkTesL      ----------------------------------------------------------------------
MlPkTesL      ----------------------------------------------------------------------
SrPkTesL      ----------------------------------------------------------------------

                      1760      1770      1780      1790      1800      1810      1820
              ....|....|....|....|....|....|....|....|....|....|....|....|....|....|
MmPkL1        FLGLCGEDDDSWCSSSTSSSDSEEEGYFLGQPIPQPRPQRFTYYTDDLSSPAS-ALPTPQFTQRTT----
MmPkL2        WGPYFTEYD--WCSTCSSSSESDNEGYFLGEPIPQPARLRYVTSDELLHKYSSYGVPKSSTLGGRG----
MmPkL3        --------------SSPSSESSEDDGFFLGERIPLPPHLCRPRTTQDTSTETFNSPAQP--LVQES----
DmPk          RERERHEESEESDVCSTCSSSSSSSSEDYMMMYQLPQRRHYGGVRVSYVPNDALAYDRKRKPSE--------
DmEsn         ----LDNCDVASICSTCSSSSSSDMD-DYVYRLPARKHYGGVRVAYVPNDALAYERKKKMAQDSSLAPGA
ChPk          ------KMELSKKATNKKTESNDSRKTARSFGMFSAEDQQMTRINYVTQDDMAMDHRRNSKSPKKS----
TaPk          -------------CITGSSSDDSQPDTNVTFNLVGKLDIQRGKSPQKVSTANSFNESRKEKKVRIVD---
MmTes         ----------------------------------------------------------------------
DmTes         ----------------------------------------------------------------------
ChTes         ----------------------------------------------------------------------
OcPkTesL      ----------------------------------------------------------------------
OlPkTesL      ----------------------------------------------------------------------
OspPkTesL     ----------------------------------------------------------------------
ScPkTesL      ----------------------------------------------------------------------
AqPkTesL      ----------------------------------------------------------------------
EmPkTesL      ----------------------------------------------------------------------
AvPkTesL      ----------------------------------------------------------------------
OmPkTesL      ----------------------------------------------------------------------
MlPkTesL      ----------------------------------------------------------------------
SrPkTesL      ----------------------------------------------------------------------
```

```
                   1830      1840
            ....|....|....|....|.
MmPkL1      -----KSKKKKGHKGKNCIIS
MmPkL2      -----QLHSRKRQKSKNCIIS
MmPkL3      -----HPVMPRQTRDKNCIVA
DmPk        ----LGGD-----KDKNCIIS
DmEsn       GNASVGGAPAIMHESKNCTIS
ChPk        --------KRKSKNQGQCVIS
TaPk        ----------EAVDKSKCIVM
MmTes       ---------------------
DmTes       ---------------------
ChTes       ---------------------
OcPkTesL    ---------------------
OlPkTesL    ---------------------
OspPkTesL   ---------------------
ScPkTesL    ---------------------
AqPkTesL    ---------------------
EmPkTesL    ---------------------
AvPkTesL    ---------------------
OmPkTesL    ---------------------
MlPkTesL    ---------------------
SrPkTesL    ---------------------
```

Abbreviations. Vertebrate: Mm, *Mus musculus.* Hexapoda: Dm, *Drosophila melanogaster.* Cnidaria: Ch, *Clytia hemisphaerica.* Placozoa: Ta, *Trichoplax adhaerens*. Porifera: Aq, *Amphimedon queenslandica*; Av, *Aphrocalistes vastus*; Em, *Ephydatia muelleri*; *Oc, Oscarella carmela*; *Ol, Oscarella lobularis*; *Om, Oopsacas minuta*; *Osp, Oscarella sp.*; Sc, *Sycon ciliatum*. Ctenophora: Ml, *Mnemiopsis leidyi*. Choanoflagellata: Sr, *Salpingoeca rosetta*.

Figure S2.6: Strabismus/Van Gogh alignment. Threshold for shading has been fixed at 80% for both identity (black) and similarity (grey). The red square indicate the final PDZ binding motif.

```
                   10        20        30        40        50        60        70
           ....|....|....|....|....|....|....|....|....|....|....|....|....|....|
MmVangL1   MDTESTYSGYSYYSSHSKKSHRQGERT----------------RERHKSPRNKDGRGSEKSVT-IQAPAG
DrVangL1   MDTDSIHSGYSHHSNRSRGSNKQSERSS---------------RDRHKPHSRDSSSRSDKNVT-ISATSV
MmVangL2   MDTESQYSGYSYKSGHSRSSRKHRDR-----------------RDRHRSKSRDGS-RGDKSVT-IQAP-G
DrVangL2   MDNESQYSGYSYKSSHSRSSRKHRDR-----------------RDRHRSKSRDSSSRGDKSVT-IQAP-G
DmVang     MENESVKSEHSGRSRRSRNHNNNGGGGGGGGGGGGGSVNNGYHRERDRSRHSHRSTHSSKSAKGFQRGDM
ChStbm     ---------------------------------------------------MDDFDDGVIEVQVIP----
TaVang     --------------------------------------------------MSSQDGEDVVEVHVIR----
OcVang     ------------------------------------------------------MDEEVVEVEVIR----
OlVang     ------------------------------------------------------MEEEVVEVEVIR----
OspVang    ----------------------------------------------------------------------

                   80        90       100       110       120       130       140
           ....|....|....|....|....|....|....|....|....|....|....|....|....|....|
MmVangL1   EPLLANDSARTGA-----------EEVQDDNWGETTT--AITG----------TSEHSISQEDIARISKD
DrVangL1   QPQQPDQSSAP-------------TDAQDDNWGETTT--AVTG----------TSDHSLSQEDLAGFGKD
MmVangL2   EPLLDNESTRG--------------DERDDNWGETTT--VVTG----------TSEHSISHDDLTRIAKD
DrVangL2   EPLLDAESTRG--------------DDRDDNWGETTT--VVTG----------TSEHSVSNEDLTRASKE
DmVang     APYQTSVNMTGDGSHDGQEVIEVQILPQDENWGENTT--AVTGN---------TSEQSISMEDINNMWHR
ChStbm     ---------------------------QDDNWGETAT--TITET---------ATSVYSDLDDTDPFGEE
TaVang     ---------------------------QDENWGVGPSGRATSIA---------TSDRSAGMGNVTFRRDH
OcVang     ---------------------------QADDWGVASTISATTASNARHG---DSAS--VGGGGVYFSNDD
OlVang     ---------------------------QSDDWGVASTISATTASKSHAGGGGDSASVVAGGGGVYFANED
OspVang    ----------------------------------------------------------------------

                  150       160       170       180       190       200       210
           ....|....|....|....|....|....|....|....|....|....|....|....|....|....|
MmVangL1   ME-DSVG-LDCKRYLGLTVASFLGLLVFLTPIAFILLP--------------QILWREELKPCGAICEGL
DrVangL1   TE-GPVEKLNCIRFLPLALTLLLGLLVLVTPLSFLILP--------------QLMWPERLQTCGTACEGL
MmVangL2   ME-DSVP-LDCSRHLGVAAGAILALLSFLTPLAFLLLP--------------PLLWREELEPCGTACEGL
DrVangL2   LE-DSSP-LECRRFAGPIVSGVLGLFALLTPLAFLLLP--------------QLLWRDSLEPCGTPCEGL
DmVang     ES-DKGFSFACRRYVESSFYFLLGCGAFFSPVAMVVMPYVGFFPSAFDHPEITQTVRTQLLACSEQCKGQ
ChStbm     IEKHRFQCPNSKQALNILLSVILALMAVVSPIAFVIIPN------------VSSSLKVTGEECGSVCEGL
TaVang     DDDDDENPAPWNSLLSIGVPIAVGTLALLTPLIFILLP-------------FMMGTTWQLSNCGNYCNGS
OcVang     DDDLAKAAKPRACDSQQCWFILLGCFALVSPIVIALAPLHP----------EWGVRTNNTDGCNYECATR
OlVang     DDDLTKASRPRACDRRQCTLVFVAFFALVSPVVIVLAPLHP----------QWGVRTNGTDGCNDECVTR
OspVang    ----------------------------------------------------------------------

                  220       230       240       250       260       270       280
           ....|....|....|....|....|....|....|....|....|....|....|....|....|....|
MmVangL1   LISVSFKLLILLIGTWALFFRKQ-RADVPRVFVFRALLLVLIFLFVVSYWLFYGVRILD---------SR
DrVangL1   FLSLAFKLLILLLAGWALFMRPT-RASLPRIAVFRALLGLLTLLLLLSYWLFFGVRILD---------SQ
MmVangL2   FISVAFKLLILLLGSWALFFRRP-KASLPRVFVLRALLMVLVFLLVISYWLFYGVRILD---------AR
DrVangL2   YVSLAFKLLVLLISSWALFLRPS-RSTLPRFFVFRCLLMALVFLFVASYWLFYGVRVLE---------PR
DmVang     LVSLAARLLLLAIGLWAVFMRRT-SATMPRIFLYRALVLLLVTICTFAYWLFYIVQVTNGAKIVVETGGD
ChStbm     YITIAIKEIVLIFGLWALYIRPN-KVDLPRLNVFKVGMMVLVYVSIICFWLFYSIRMIG----------N
TaVang     YLTLAIKEIILLIGIWALYSRHHSHYTLPRLISYKLFVLSLVLMVTICYWIYYVVAVIIP---------K
OcVang     FVGFGTRLVLLAVAIFALFFRRA-RASGPAVYRLKVVALLLSVAVLTAYWVVFGLVLARK---------T
OlVang     CVGFGARLVLLAVALVALFLRTA-RASGTRVDRVRALSLLLAFLVLVAYWSIFGVVLVRR---------T
OspVang    --------------------RRT-RASGPAIERVRFFAVLLSFVVLVAYWVVFGLVLARN---------T
```

Abbreviations. Vertebrate: Dr, *Danio rerio* and Mm, *Mus musculus.* Hexapoda: Dm, *Drosophila melanogaster.* Cnidaria: Ch, *Clytia hemisphaerica.* Placozoa: Ta, *Trichoplax adhaerens*. Porifera: *Oc, Oscarella carmela*; *Ol, Oscarella lobularis*; *Om, Oopsacas minuta*; *Osp, Oscarella sp*.

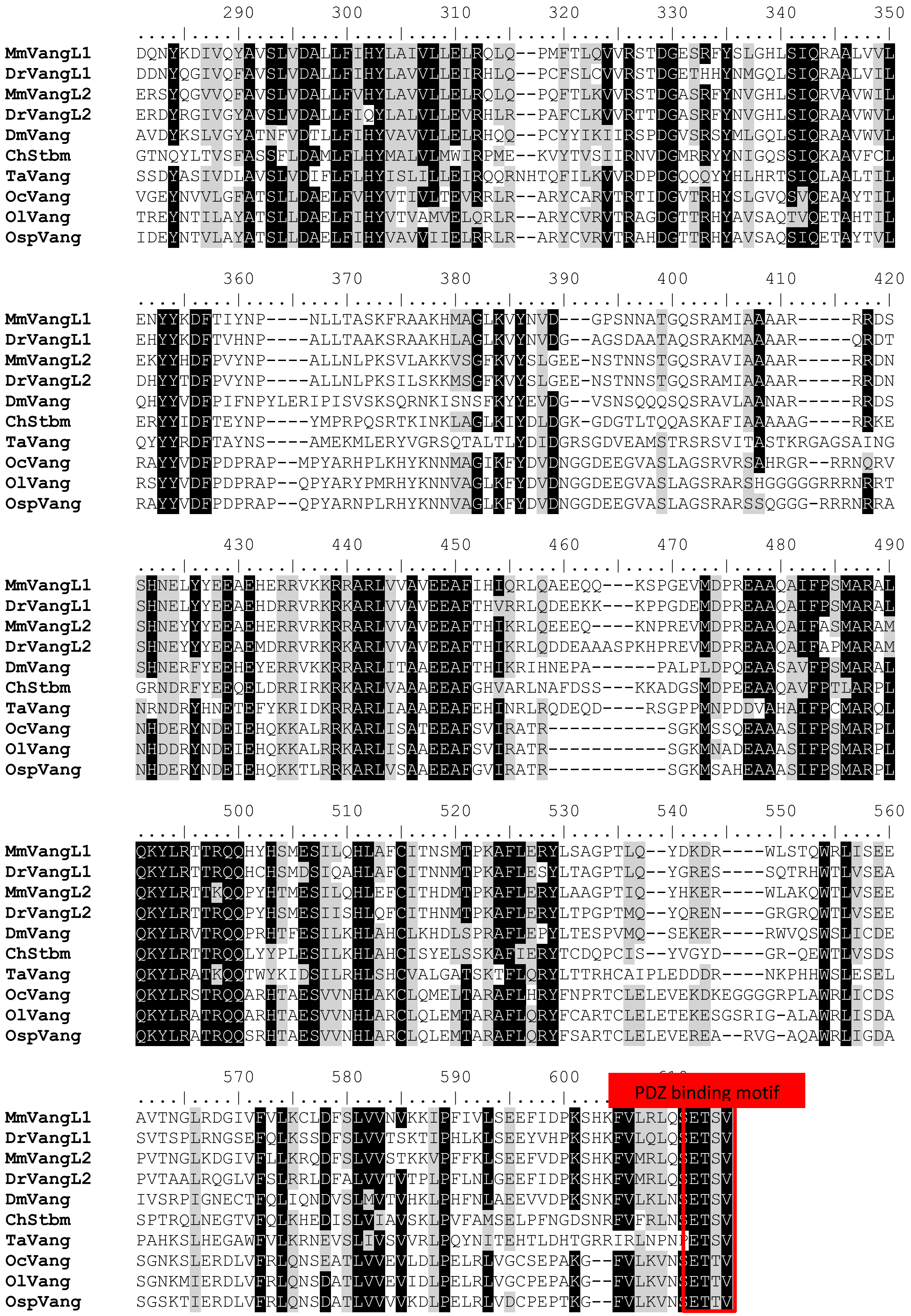
290 300 310 320 330 340 350
MmVangL1 DQNYKDIVQYAVSLVDALLFIHYLAIVLLELRQLQ--PMFTLQVVRSTDGESRFYSLGHLSIQRAALVVL
DrVangL1 DDNYQGIVQFAVSLVDALLFIHYLAVVLLEIRHLQ--PCFSLCVVRSTDGETHHYNMGQLSIQRAALVIL
MmVangL2 ERSYQGVVQFAVSLVDALLFVHYLAVVLLELRQLQ--PQFTLKVVRSTDGASRFYNVGHLSIQRVAVWIL
DrVangL2 ERDYRGIVGYAVSLVDALLFIQYLALVLLEVRHLR--PAFCLKVVRTTDGASRFYNVGHLSIQRAAVWVL
DmVang AVDYKSLVGYATNFVDTLLFIHYVAVVLLELRHQQ--PCYYIKIIRSPDGVSRSYMLGQLSIQRAAVWVL
ChStbm GTNQYLTVSFASSFLDAMLFLHYMALVLMWIRPME--KVYTVSIIRNVDGMRRYYNIGQSSIQKAAVFCL
TaVang SSDYASIVDLAVSLVDIFLFLHYISLILLEIRQQRNHTQFILKVVRDPDGQQQYYHLHRTSIQLAALTIL
OcVang VGEYNVVLGFATSLLDAELFVHYVTIVLTEVRRLR--ARYCARVTRTIDGVTRHYSLGVQSVQEAAYTIL
OlVang TREYNTILAYATSLLDAELFIHYVTVAMVELQRLR--ARYCVRVTRAGDGTTRHYAVSAQTVQETAHTIL
OspVang IDEYNTVLAYATSLLDAELFIHYVAVVIIELRRLR--ARYCVRVTRAHDGTTRHYAVSAQSIQETAYTVL
360 370 380 390 400 410 420
MmVangL1 ENYYKDFTIYNP----NLLTASKFRAAKHMAGLKVYNVD---GPSNNATGQSRAMIAAAAR-----RRDS
DrVangL1 EHYYKDFTVHNP----ALLTAAKSRAAKHLAGLKVYNVDG--AGSDAATAQSRAKMAAAAR-----QRDT
MmVangL2 EKYYHDFPVYNP----ALLNLPKSVLAKKVSGFKVYSLGEE-NSTNNSTGQSRAVIAAAAR-----RRDN
DrVangL2 DHYYTDFPVYNP----ALLNLPKSILSKKMSGFKVYSLGEE-NSTNNSTGQSRAMIAAAAR-----RRDN
DmVang QHYYVDFPIFNPYLERIPISVSKSQRNKISNSFKYYEVDG--VSNSQQQSQSRAVLAANAR-----RRDS
ChStbm ERYYIDFTEYNP----YMPRPQSRTKINKLAGLKIYDLDGK-GDGTLTQQASKAFIAAAAAG----RRKE
TaVang QYYYRDFTAYNS----AMEKMLERYVGRSQTALTLYDIDGRSGDVEAMSTRSRSVITASTKRGAGSAING
OcVang RAYYVDFPDPRAP--MPYARHPLKHYKNNMAGIKFYDVDNGGDEEGVASLAGSRVRSAHRGR--RRNQRV
OlVang RSYYVDFPDPRAP--QPYARYPMRHYKNNVAGLKFYDVDNGGDEEGVASLAGSRARSHGGGGGRRRNRRT
OspVang RAYYVDFPDPRAP--QPYARNPLRHYKNNVAGLKFYDVDNGGDEEGVASLAGSRARSSQGGG-RRRNRRA
430 440 450 460 470 480 490
MmVangL1 SHNELYYEEAEHERRVKKRRARLVVAVEEAFIHIQRLQAEEQQ---KSPGEVMDPREAAQAIFPSMARAL
DrVangL1 SHNELYYEEAEHDRRVRKRKARLVVAVEEAFTHVRRLQDEEKK---KPPGDEMDPREAAQAIFPSMARAL
MmVangL2 SHNEYYYEEAEHERRVRKRRARLVVAVEEAFTHIKRLQEEEQ----KNPREVMDPREAAQAIFASMARAM
DrVangL2 SHNEYYYEEAEMDRRVRKRKARLVVAVEEAFTHIKRLQDDEAAASPKHPREVMDPREAAQAIFAPMARAM
DmVang SHNERFYEEHEYERRVKKRRARLITAAEEAFTHIKRIHNEPA------PALPLDPQEAASAVFPSMARAL
ChStbm GRNDRFYEEQELDRRIRKRKARLVAAAEEAFGHVARLNAFDSS---KKADGSMDPEEAAQAVFPTLARPL
TaVang NRNDRYHNETEFYKRIDKRRARLIAAAEEAFEHINRLRQDEQD----RSGPPMNPDDVAHAIFPCMARQL
OcVang NHDERYNDEIEHQKKALRRKARLISATEEAFSVIRATR-----------SGKMSSQEAAASIFPSMARPL
OlVang NHDDRYNDEIEHQKKALRRKARLISAAEEAFSVIRATR-----------SGKMNADEAAASIFPSMARPL
OspVang NHDERYNDEIEHQKKTLRRKARLVSAAEEAFGVIRATR-----------SGKMSAHEAAASIFPSMARPL
500 510 520 530 540 550 560
MmVangL1 QKYLRTTRQQHYHSMESILQHLAFCITNSMTPKAFLERYLSAGPTLQ--YDKDR----WLSTQWRLISEE
DrVangL1 QKYLRTTRQQHCHSMDSIQAHLAFCITNNMTPKAFLESYLTAGPTLQ--YGRES----SQTRHWTLVSEA
MmVangL2 QKYLRTTKQQPYHTMESILQHLEFCITHDMTPKAFLERYLAAGPTIQ--YHKER----WLAKQWTLVSEE
DrVangL2 QKYLRTTRQQPYHSMESIISHLQFCITHNMTPKAFLERYLTPGPTMQ--YQREN----GRGRQWTLVSEE
DmVang QKYLRVTRQQPRHTFESILKHLAHCLKHDLSPRAFLEPYLTESPVMQ--SEKER----RWVQSWSLICDE
ChStbm QKYLRTTRQQLYYPLESILKHLAHCISYELSSKAFIERYTCDQPCIS--YVGYD----GR-QEWTLVSDS
TaVang QKYLRATKQQTWYKIDSILRHLSHCVALGATSKTFLQRYLTTRHCAIPLEDDDR----NKPHHWSLESEL
OcVang QKYLRSTRQQARHTAESVVNHLAKCLQMELTARAFLHRYFNPRTCLELEVEKDKEGGGGRPLAWRLICDS
OlVang QKYLRATRQQARHTAESVVNHLARCLQLEMTARAFLQRYFCARTCLELETEKESGSRIG-ALAWRLISDA
OspVang QKYLRATRQQSRHTAESVVNHLARCLQLEMTARAFLQRYFSARTCLELEVEREA--RVG-AQAWRLIGDA
570 580 590 600
PDZ binding motif
MmVangL1 AVTNGLRDGIVFVLKCLDFSLVVNVKKIPFIVLSEEFIDPKSHKFVLRLQSETSV
DrVangL1 SVTSPLRNGSEFQLKSSDFSLVVTSKTIPHLKLSEEYVHPKSHKFVLQLQSETSV
MmVangL2 PVTNGLKDGIVFLLKRQDFSLVVSTKKVPFFKLSEEFVDPKSHKFVMRLQSETSV
DrVangL2 PVTAALRQGLVFSLRRLDFALVVTVTPLPFLNLGEEFIDPKSHKFVMRLQSETSV
DmVang IVSRPIGNECTFQLIQNDVSLMVTVHKLPHFNLAEEVVDPKSNKFVLKLNSETSV
ChStbm SPTRQLNEGTVFQLKHEDISLVIAVSKLPVFAMSELPFNGDSNRFVFRLNSETSV
TaVang PAHKSLHEGAWFVLKRNEVSLIVSVVRLPQYNITEHTLDHTGRRIRLNPNPETSV
OcVang SGNKSLERDLVFRLQNSEATLVVEVLDLPELRLVGCSEPAKG--FVLKVNSETTV
OlVang SGNKMIERDLVFRLQNSDATLVVEVIDLPELRLVGCPEPAKG--FVLKVNSETTV
OspVang SGSKTIERDLVFRLQNSDATLVVVVKDLPELRLVDCPEPTKG--FVLKVNSETTV

Figure S2.7: Maximum likelihood unrooted tree of PET family.

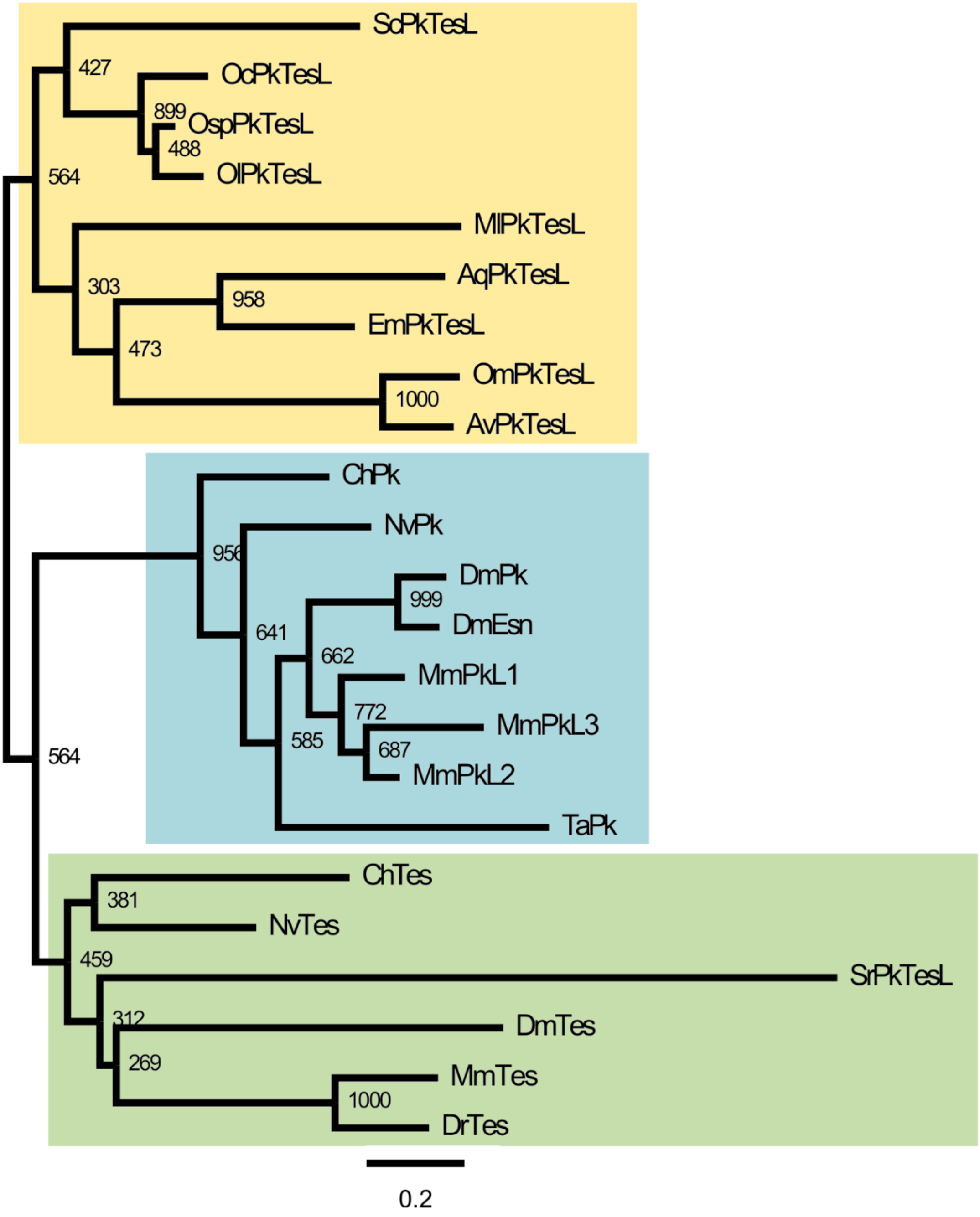

Percentage bootstrap support obtained in maximum likelihood analysis is indicated each node. Abbreviations. Vertebrate: Dr, *Danio rerio*; Gg, *Gallus gallus*; Hs, *Homo sapiens* and Mm, *Mus musculus.* Hexapoda: Dm, *Drosophila melanogaster.* Mollusca: Ac, *Aplysia californica* and Cg, *Crassotrea gigantea*. Ambulacraria: Hp, *Hemicentrotus pulcherrimus*; Sk, *Saccoglossus kowalevskii* and Sp, *Strongylocentrotus purpuratus*. Cnidaria: Ch, *Clytia hemisphaerica;* Nv, *Nematostella vectensis.* Placozoa: Ta, *Trichoplax adhaerens*. Porifera: Aq, *Amphimedon queenslandica*; Av, *Aphrocalistes vastus*; Em, *Ephydatia muelleri*; *Oc, Oscarella carmela*; *Ol, Oscarella lobularis*; *Om, Oopsacas minuta*; *Osp, Oscarella sp.*; Sc, *Sycon ciliatum*. Ctenophora: Ml, *Mnemiopsis leidyi*. Choanoflagellata: Sr, *Salpingoeca rosetta*.

Figure S2.8: Bayesian unrooted tree of PET family.

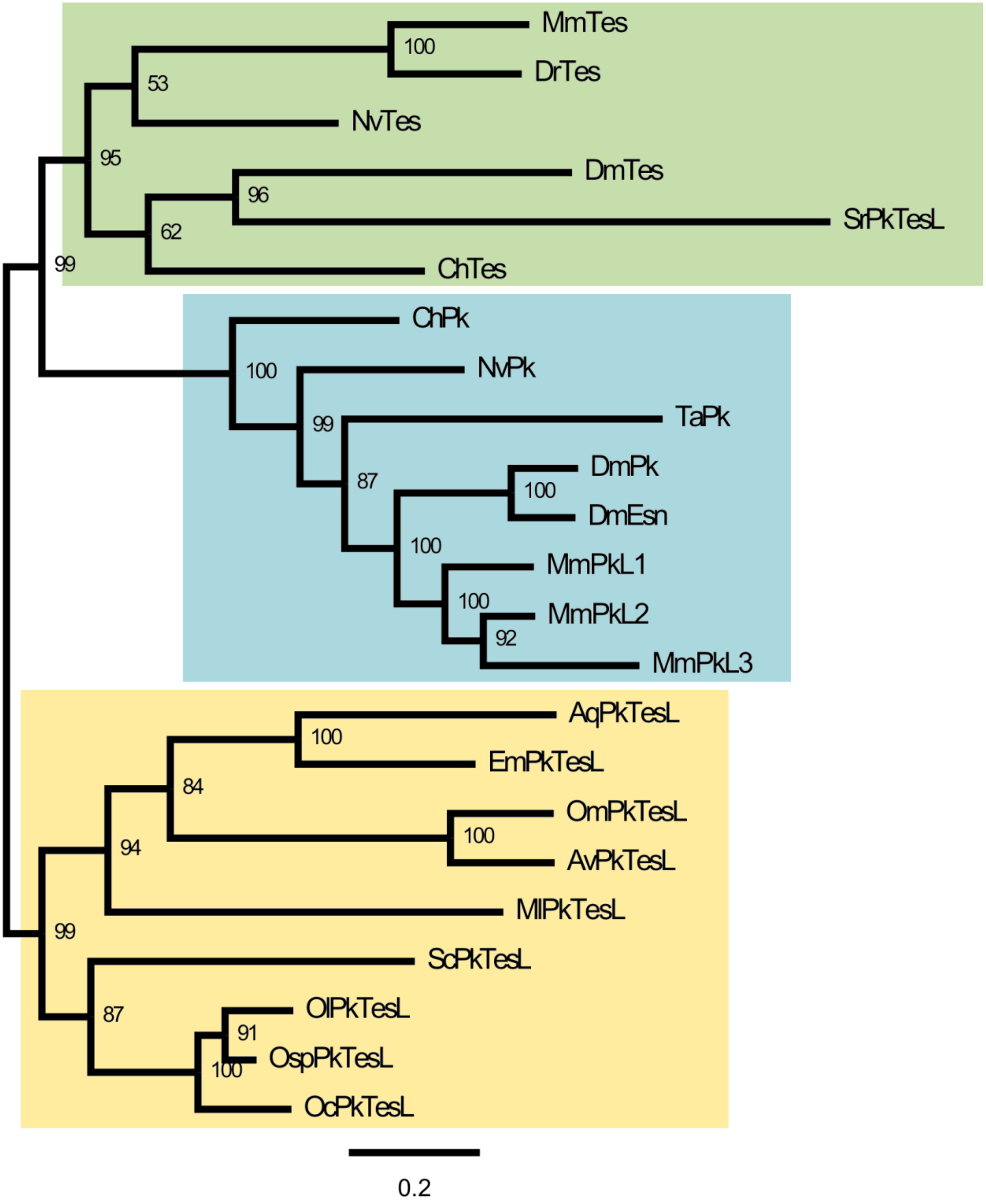

Posterior probability obtained in Bayesian analysis is indicated above each node. Abbreviations. Vertebrate: Dr, *Danio rerio*; Gg, *Gallus gallus*; Hs, *Homo sapiens* and Mm, *Mus musculus.* Hexapoda: Dm, *Drosophila melanogaster.* Mollusca: Ac, *Aplysia californica* and Cg, *Crassotrea gigantea*. Ambulacraria: Hp, *Hemicentrotus pulcherrimus*; Sk, *Saccoglossus kowalevskii* and Sp, *Strongylocentrotus purpuratus*. Cnidaria: Ch, *Clytia hemisphaerica;* Nv, *Nematostella vectensis.* Placozoa: Ta, *Trichoplax adhaerens*. Porifera: Aq, *Amphimedon queenslandica*; Av, *Aphrocalistes vastus*; Em, *Ephydatia muelleri*; *Oc, Oscarella carmela*; *Ol, Oscarella lobularis*; *Om, Oopsacas minuta*; *Osp, Oscarella sp.*; Sc, *Sycon ciliatum*. Ctenophora: Ml, *Mnemiopsis leidyi*. Choanoflagellata: Sr, *Salpingoeca rosetta*.

Figure S2.9: Intro/exon composition of *flamingo* in *Sycon ciliatum*.

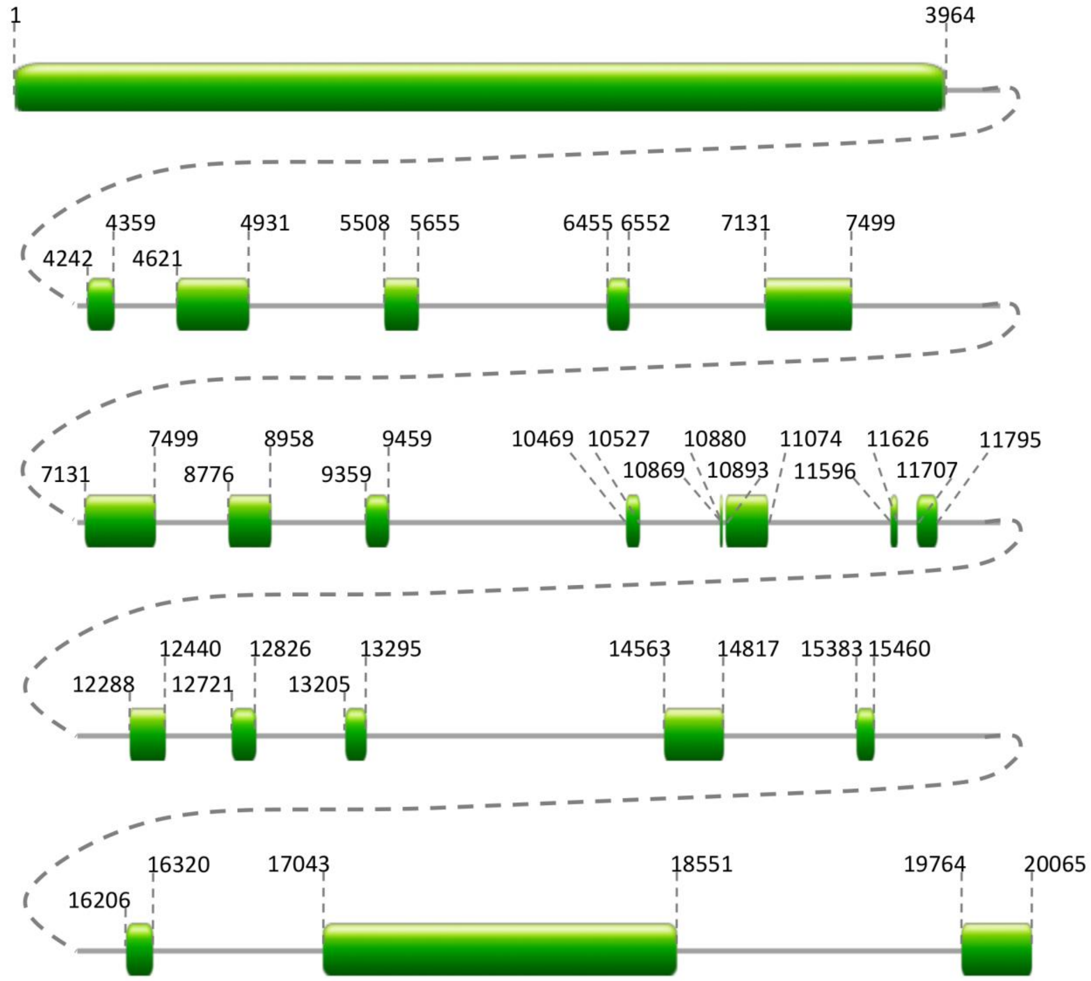

Location of exons is indicated in base pairs along the gene.

Figure S2.10: Alternative scenario 1 of the PCP players during animal evolution.

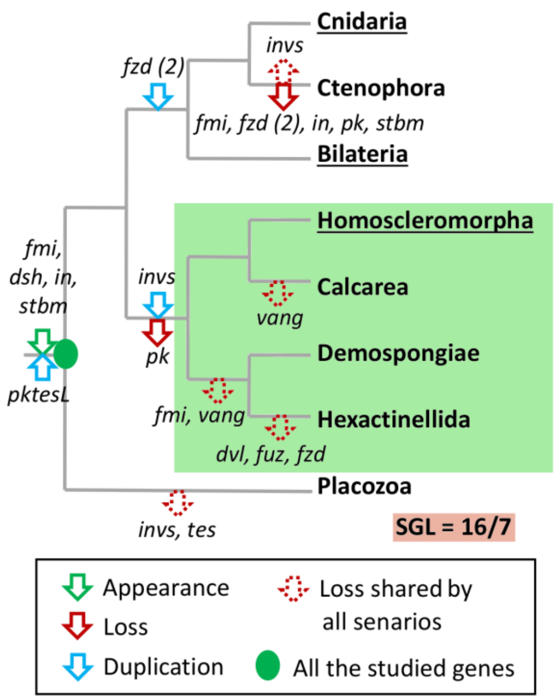

The emergence (green arrows), duplications (blue arrows) and secondarily losses (red arrows) of PCP genes are indicated (16 secondarily gene losses, SGL), based on genome analyses. Abbreviations. *dsh*, *dishevelled*; *fmi*, *flamingo*; *fuz*, *fuzzy*; *fzd*, *frizzled*; *intu*, *inturned*; *invs*, *inversin*; *pktesL*, *prickle-testin like*; *pk*, *prickle*; *tes*, *testin* and *vang*, *van gogh*.

Figure S2.11: Alternative scenario 2 of the PCP players during animal evolution.

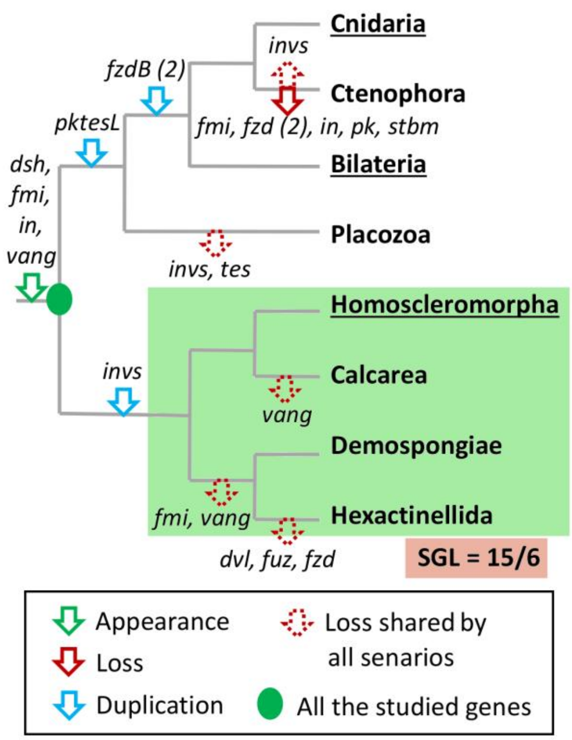

The emergence (green arrows), duplications (blue arrows) and secondarily losses (red arrows) of PCP genes are indicated (15 secondarily gene losses, SGL), based on genome analyses. Abbreviations. *dsh*, *dishevelled*; *fmi*, *flamingo*; *fuz*, *fuzzy*; *fzd*, *frizzled*; *intu*, *inturned*; *invs*, *inversin*; *pktesL*, *prickle-testin like*; *pk*, *prickle*; *tes*, *testin* and *vang*, *van gogh*.

Figure S2.12: Alternative scenario 3 of the PCP players during animal evolution.

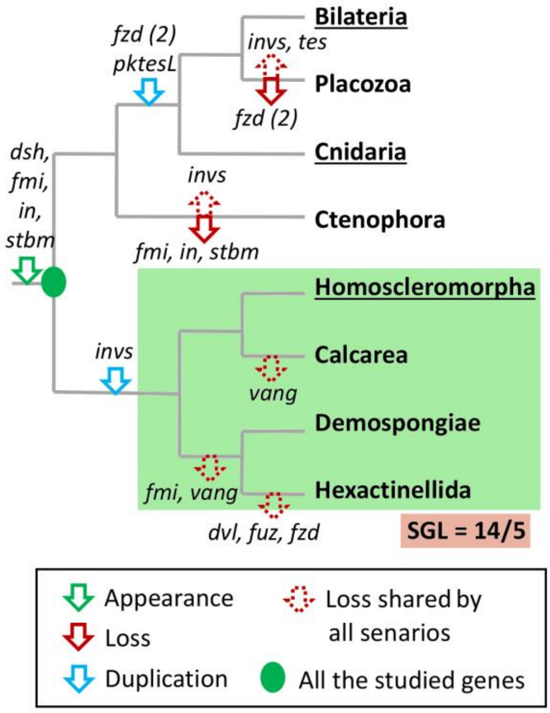

The emergence (green arrows), duplications (blue arrows) and secondarily losses (red arrows) of PCP genes are indicated (14 secondarily gene losses, SGL), based on genome analyses. Abbreviations. *dsh*, *dishevelled*; *fmi*, *flamingo*; *fuz*, *fuzzy*; *fzd*, *frizzled*; *intu*, *inturned*; *invs*, *inversin*; *pktesL*, *prickle-testin like*; *pk*, *prickle*; *tes*, *testin* and *vang*, *van gogh*.
